# Relationship Between Improvements in Glycemic Control and Risk of Pregnancy Complications in Patients With Diabetes Mellitus: Metaregression Analysis of Randomized Controlled Trials of Intensive Glucose Management

**DOI:** 10.1155/jdr/3490884

**Published:** 2025-06-23

**Authors:** Satoru Kodama, Kazuya Fujihara, Noriko Yagyuda, Yoko Yachi, Chika Horikawa, Yasunaga Takeda, Sakiko Yoshizawa Morikawa, Takaho Yamada, Kiminori Kato, Yoshimi Nakagawa, Shiro Tanaka, Hitoshi Shimano, Hirohito Sone

**Affiliations:** ^1^Department of Prevention of Noncommunicable Diseases and Promotion of Health Checkup, Niigata University Graduate School of Medical and Dental Sciences, Niigata, Japan; ^2^Department of Hematology, Endocrinology and Metabolism, Niigata University Graduate School of Medical and Dental Sciences, Niigata, Japan; ^3^Department of Nutrition, Faculty of Health Care Science, Chiba Prefectural University of Health Sciences, Chiba, Japan; ^4^Department of Health and Nutrition, Faculty of Human Life Studies, University of Niigata Prefecture, Niigata, Japan; ^5^Department of Food and Nutrition, Tokushima Bunri University Faculty of Human Life Science, Tokushima, Japan; ^6^Department of Complex Biosystem Research, Institute of Natural Medicine, University of Toyama, Toyama, Japan; ^7^Department of Pharmacoepidemiology, Kyoto University School of Public Health, Kyoto, Japan; ^8^Department of Internal Medicine (Endocrinology and Metabolism), Faculty of Medicine, University of Tsukuba, Tsukuba, Ibaraki, Japan

**Keywords:** diabetes mellitus, glycemic control, pregnancy complications, metaregression

## Abstract

**Background:** It remains unknown whether improvements in prognosis of pregnancy in patients with diabetes mellitus are dependent on glycemic control (GC). This metaregression analysis was aimed at exploring the relationships between the improvements in GC.

**Methods:** Using Embase and MEDLINE (from Jan. 1, 1950, to Apr. 29, 2024), we searched for randomized controlled trials of intensive glucose management in pregnant women with gestational, pregestational, or overt diabetes mellitus, in which the blood glucose level using any GC indicator and the incidence of any adverse maternal and/or fetal outcome were compared between two groups with different intensities of glucose management. Relative risks (RRs) of individual adverse outcomes were regressed on the reductions in individual GC indicators.

**Results:** We examined the dose–response relationship between reductions in four GC indicators (hemoglobin A1c (A1C), fasting plasma glucose (FPG), 2-h postprandial glucose, and mean blood glucose) and 14 adverse pregnancy outcomes in 62 eligible trials. Reductions in FPG were associated with the reduced risk of 10/14 adverse outcomes, with the exceptions being cesarean section, small for gestational age, premature rupture of membranes, and congenital malformation. Reductions in A1C were strongly associated with the reduced risk of cesarean section (*r* = 0.67, *p* < 0.001), indicating that the RR (95% confidence interval) for a 1% incremental decrease in A1C was 0.63 (0.49–0.80).

**Conclusions:** Risk reductions in the majority of pregnancy complications in those with diabetes depend on the improvement of GC induced by intensive glucose management.

**Summary**

• Whether reduced risk of pregnancy complications in diabetes is dependent on the improvement of glycemic control (GC) is unknown.
• Metaregression analysis in previous trials including intensive glucose management indicated positive dose–response associations between reductions in at least one GC indicator and risk of 11 main adverse outcomes.
• Improvement in pregnancy prognosis in patients with diabetes depends on improvement of GC during pregnancy.

## 1. Introduction

Prevalence of diabetes in pregnancy has increased in parallel with the worldwide epidemic of obesity. The increase includes not only the prevalence of Type 1 diabetes (T1D) and Type 2 diabetes (T2D) (pregestational diabetes) among those of reproductive age but also gestational diabetes mellitus (GDM) [1]. Diabetes confers significant maternal and fetal risks such as miscarriage, preeclampsia, macrosomia, neonatal hypoglycemia, hyperbilirubinemia, and neonatal respiratory distress syndrome (RDS) [1]. In particular, poor GC has been associated with a high risk of adverse maternal and fetal outcomes such as cesarean section (CS), shoulder dystocia, and macrosomia [2].

Interventions mainly aimed at improving GC in pregnant women with pregestational diabetes [3] and GDM [4–7] are effective in reducing the risk of some maternal and fetal/neonatal complications. However, it is an unsolved issue of whether the risk reduction is dependent on GC or could otherwise be attributed to an education program regardless of the improvement in GC. To address this issue, we performed a meta-analysis of previous trials of intensive glucose management among patients with diabetes focusing on the impact of GC during pregnancy on the risk of adverse pregnancy outcomes.

## 2. Methods

### 2.1. Protocol

Before performing this meta-analysis, the protocol was registered in the international prospective register of systematic reviews (PROSPERO) (CRD42022356069).

### 2.2. Literature Search

Electronic literature search was conducted for randomized controlled trials (RCTs) published from Jan. 1, 1950, to Apr. 29, 2024 using MEDLINE and Embase. Search strategy is shown in Table 1. We listed any possible terms related to the following four elements: (1) pregnant women with diabetes mellitus, (2) intensive glucose management, (3) adverse pregnancy outcomes, and (4) RCT. These were combined using the Boolean operator “AND.”

### 2.3. Inclusion and Exclusion Criteria

Study inclusion criteria were (1) RCT with a parallel design; (2) all participants had diabetes including T1D, T2D, pregestational diabetes, overt diabetes, or GDM; (3) interventions during pregnancy that included two groups consisting of an intervention and a control group; (4) assessment of the incidence of at least one adverse pregnancy outcome as a study outcome; and (5) assessment of improvement of GC as the exposure.

As to Criteria 3, the intervention had to include intensive glucose management by at least one of the following: (a) frequent monitoring of blood glucose, (b) addition of a glucose-lowering drug or increasing dosage of an already prescribed glucose-lowering drug, and (c) establishing a strict glycemic target and adjusting drug dosages to achieve the glycemic target. We excluded articles having an abstract only or were presented at a conference because study quality could not be assessed. We also excluded trials in which the intensity of glucose management was not differentiated (e.g., Web-based vs. in-person education, metformin vs. insulin).

### 2.4. Definition of Study Outcomes and Exposures

We considered all maternal or fetal/neonatal adverse outcomes that occurred from the beginning of pregnancy until the perinatal period. Exceptionally, maternal hypoglycemia was not considered because it is not specific to pregnant women with diabetes mellitus. The corresponding effect measure was relative risk (RR) in the intervention group versus the control group. To calculate RR, included trials had to provide data on the number of cases and noncases for each adverse pregnancy outcome. If there was no event in either the intervention or control group, we added 0.5 to the number of patients and events in each group [8].

The GC indicators included A1C, fasting plasma glucose (FPG), postprandial glucose, and mean blood glucose (MBG). As to study exposures, included studies had to provide information to calculate the mean difference between intervention and control groups in the blood glucose level after the intervention involving at least one of the four GC indicators. If we could not determine the number of cases and noncases or the mean difference in GC indicators between the intervention and control groups, we queried the study author for data clarification.

### 2.5. Data Extraction

Two authors (S.K. and K.F.) extracted the data. Disagreements were solved by the third author (H.So.). Extracted data were as follows: first author, year, country of the study, number of patients, type of diabetes, mean age, mean body mass index (BMI) at entry or pregestational BMI, methods for glucose management, whether the methods included drug interventions, blood glucose levels after the intervention, and adverse pregnancy outcomes.

Study quality was assessed using the revised Cochrane risk-of-bias tool for randomized trials (RoB 2) [9]. This tool is structured into five domains through which bias might be introduced into the results: R, bias arising from the randomization process; D, bias due to deviations from intended interventions; Mi, bias due to missing outcome data; Me, bias in measurement of the outcome; and S, bias in selection of the reported results. For each domain, we allocated one of three categories: “high,” “some concerns,” and “low.” Overall risk of bias was considered “high” if there was a high risk of bias in at least one domain and/or some concerns about risk of bias in three or more domains. It was considered low if risk of bias was considered low in any domain. Otherwise, there were some concerns about the overall risk of bias (i.e., neither low nor high risk of bias).

### 2.6. Data Synthesis

Primarily, the overall RR of each adverse pregnancy outcome was estimated using the Mantel–Haenszel method [10]. Between-study heterogeneity was assessed using *I*-squared statistics [11]. If significant heterogeneity using Cochrane's *Q* test [12] was statistically detected, a random-effects model was adapted; otherwise, a fixed-effects model was chosen. Analysis was stratified by the following trials' characteristics: methods for intensive glucose management, whether a drug intervention was included or not, type of diabetes (i.e., GDM or others), and geographic region (i.e., Asia/Middle East or others). The influence of these characteristics on the pooled RR was examined by metaregression using the STATA command “metareg.” Publication bias was visually assessed by funnel plots and formally assessed by two statistical tests, Begg's rank correlation test [13] and Egger's regression asymmetry test [14]. If a statistically significant publication bias was detected by at least one of the above two tests, we used a trim–fill method to adjust the pooled estimate for publication bias [15]. This method includes the assumption that the funnel plots of the results (i.e., effect sizes and their corresponding standard errors) of published studies are symmetrical without publication bias. If the funnel plots are asymmetrical, then the results from hypothetically unpublished studies that caused the asymmetrical funnel plots were trimmed to restore the symmetry, and the pooled estimate was recalculated including the results from both the published and hypothetically unpublished studies.

Secondarily, to examine the dose–response relationship between improved GC and the reduced risk of adverse pregnancy complications, the RRs for each adverse outcome were regressed on the differences in each GC indicator between intervention and control groups, where the RRs were weighted by their corresponding inverse of logarithms of RR. The correlation coefficient (*r*) in each metaregression analysis was calculated as follows: $r=\pm \sqrt{1-\left({\text{SS}}_{\text{after}}/{\text{SS}}_{\text{before}}\right)}$, where SS_before_ and SS_after_ were the sum of the square before and after entering the difference as the explanatory variable and *r* was minus if reductions in a GC indicator were negatively associated with risk reductions in an adverse pregnancy outcome. *p* < 0.05 was considered to be statistically significant except that *p* < 0.10 was used to detect a significant publication bias [16]. All statistical analyses were conducted using STATA statistical software (Version 16, Stata Corporation, College Station, Texas, United States).

## 3. Results

### 3.1. Literature Searches

**Figure 1:**
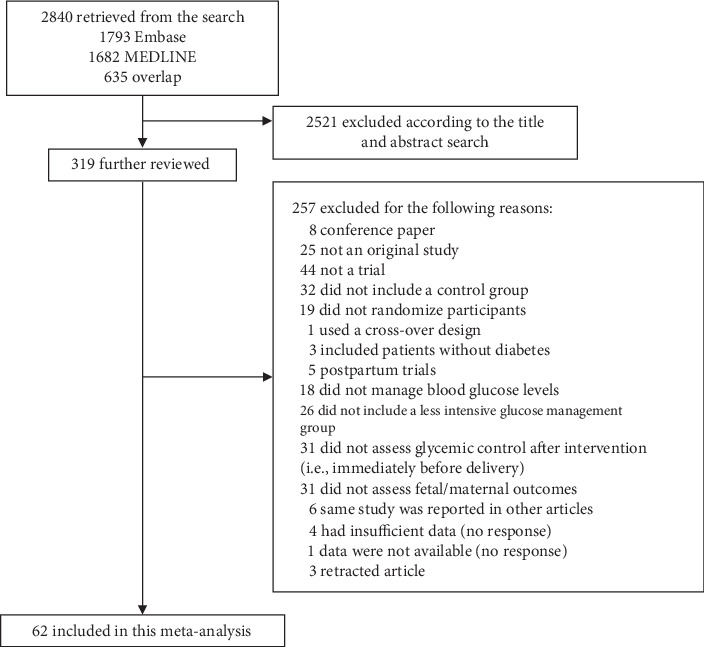
Study flowchart for identifying eligible trials.

Figure 1 is a flowchart showing the selection of eligible studies. Of 2840 articles retrieved from electronic literature searches, 319 articles were kept for full-paper review. In addition to 252 articles that were found to be ineligible, we excluded five trials because the authors did not respond to our queries. In one of the five articles [17], the full paper was unavailable despite a request to obtain it. The other four of the five trials had insufficient data to perform this meta-analysis. It was impossible to estimate the number of cases and noncases in one study [18] and impossible to calculate the mean difference in blood glucose levels between groups in three studies [19–21]. Finally, 62 ( = 319 − 252 − 5) eligible trials [19, 22–82] consisting of 11,989 patients (6030 in the intervention group and 5959 patients in the control group) were identified and included in this meta-analysis.

### 3.2. Study Characteristics

Table 2 summarizes the characteristics of trials included in this meta-analysis. Thirty-six trials were conducted in Asia and the Middle East. Twenty-four trials were conducted in Europe and North America, and the other two trials were multicenter studies conducted in two or more countries. Most of the included trials (50/62 trials) targeted patients with GDM while four trials and one trial targeted patients with T1D and T2D, respectively. Another four trials targeted those with pregestational or overt diabetes. The type of diabetes was not specified in the remaining three trials.  Table 3 summarizes blood glucose levels after the intervention in both the intervention and control groups. As the GC indicator, A1C, FPG, 2-h postprandial glucose (2hPG), or MBG was used in 41 trials, 39 trials, 30 trials, and 17 trials, respectively.

Methods for glucose management were categorized into four types: (1) glucose-monitoring only (22 trials), (2) introducing new glucose-lowering drugs such as sulfonylurea and insulin (11 trials), (3) setting a strict glycemic goal (5 trials), and (4) multifocal (i.e., combining 1–3) (24 trials). Except for two trials, mean age was homogeneous among studies and ranged from 25 to 35 years. Obesity indicator was assessed by prepregnancy BMI/body weight and/or BMI/body weight at study entry. Adverse pregnancy outcomes examined in the included trials are summarized in Table 4. There were 36 types of adverse (19 maternal and 17 fetal/neonatal) outcomes consisting of 15,430 events that were identified by two or more trials.  Table 5 shows maternal and fetal/neonatal adverse outcomes identified in each trial. The number of examined outcomes ranged from 1 to 22 (median, 9 outcomes).

### 3.3. Study Quality

Results of risk of bias using RoB 2 are shown in Table 6. Reasons for a high risk of bias were that “randomization” was not stated (R domain), per-protocol analysis was used although there were patients lost to follow-up (D domain), and the arbitrary use of medical records or description of outcomes in the Results section although they were not mentioned in the Methods section (S domain). Other reasons for concerns about the risk of bias were not describing methods for randomization (R domain), not specifying outcomes in the Methods section (S domain), and neither mentioning nor having robust or missing data although these were unlikely to influence study results (Mi domain). All trials had low risk of bias for the Me domain. Overall, we finally judged 17, 20, and 25 trials as having low, unknown, and high risk of bias, respectively.

### 3.4. Overall Effect of Intensive Glucose Management on the Risk of Adverse Pregnancy Outcomes

Table 7 shows the overall effect of intensive glucose management on the risk of maternal and fetal adverse pregnancy outcomes. Risks of 17 of 36 specified outcomes were significantly reduced through intensive glucose management. Intensive glucose management did not elevate the risk of any of the adverse outcomes. Corresponding forest plots showing RRs with 95% CI from included trials by individual adverse outcomes are shown in Supporting Information 1.

The statistically significant publication bias that was detected in the 14 overall estimates and the corresponding funnel plots (Supporting Information 2) suggested that all risk reductions were exaggerated due to the existence of hypothetically unpublished studies. However, adjustment for publication bias using the trim–fill method did not change the general conclusions except for the pooled RR for pregnancy-induced hypertension (PIH), neonatal hypoglycemia, and RDS, for which significantly low RRs were changed to insignificant.

Table 8 shows stratified analyses of the effect of intensive glucose management on adverse pregnancy outcomes; these analyses were limited to 14 outcomes for which there were 15 or more pieces of data including nonzero events. A larger effectiveness of intensive glucose management was observed in trials conducted in Asia and the Middle East compared with those in countries other than in Asia and the Middle East (*p* = 0.01 for preterm delivery; *p* = 0.01 for macrosomia; *p* = 0.002 for neonatal hypoglycemia; *p* = 0.004 for admission to neonatal intensive care units (NICUs); *p* = 0.01 for RDS). In general, the content of the intensive glucose management did not affect the magnitude of the risk of adverse pregnancy outcomes except for neonatal hypoglycemia (*p* = 0.01) and admission to NICU (*p* = 0.02). In these outcomes, multifocal interventions yielded significantly larger risk reductions (*p* < 0.001 for neonatal hypoglycemia; *p* = 0.045 for admission to NICU) while using only drug interventions was significantly less efficacious in reducing the risk of admission to NICU (*p* = 0.003) compared with the use of other methods of intensive glucose management.

Including drug interventions for GC did not contribute to risk reductions and was less efficacious than not including drug interventions in reducing risk of admission to NICU (*p* = 0.004) and RDS (*p* = 0.04). As to small for gestational age (SGA), risk was significantly elevated by setting a strict GC goal (RR [95% CI], 5.40 [1.44–20.23]; *p* = 0.04 for difference from other methods of glucose management) and including a drug intervention (RR [95% CI], 1.36 [1.05–1.75]). Intensive glucose management was more efficacious in trials of patients with GDM than in those with patients not having GDM in reducing the risk of macrosomia (*p* = 0.03) and fetal distress (*p* = 0.03). The risks were significantly reduced in trials for patients with GDM (RR [95% CI], 0.63 [0.56–0.71] for macrosomia; 0.38 [0.28–0.52] for fetal distress) but not significantly reduced in trials for patients not having GDM (RR [95% CI], 0.93 [0.69–1.25] for macrosomia; 0.92 [0.55–1.54] for fetal distress). The risk of SGA was significantly elevated in trials for patients with other types of diabetes (RR [95% CI], 1.84 [1.11–3.04]) but not in trials for patients having GDM (RR [95% CI], 1.09 [0.84–1.41]).

### 3.5. Metaregression Analysis to Explore the Dose–Response Relationship Between Reductions in GC Indicators and Risk of Adverse Pregnancy Outcomes

Table 9 shows the RR for incremental reductions in the four GC indicators in the same 14 adverse pregnancy outcomes as described in Table 8. Supporting Information 3 shows the corresponding scatter plots where the RRs for each of the 14 outcomes were plotted on the reductions in each of the four GC indicators. There were no significant relationships between reductions in any GC indicator and RRs for SGA, premature rupture of membranes (PROMs), and congenital malformation. However, as to the other 11 outcomes, the RRs for incremental reductions in at least one GC indicator were significantly lowered (i.e., < 1).

Among the four GC indicators, for FPG, there was the largest number of significantly lowered RRs (10 of 14 outcomes) although the RR for 10 mg/dL reductions was not significant for CS (RR [95% CI], 0.86 [0.72–1.01] [*p* = 0.07]) as well as for SGA, PROM, and congenital malformation as previously mentioned. Significantly lowered RR for 1% reductions in A1C was observed in six adverse outcomes. In particular, regarding reduced risk of CS, there was a strong association between A1C reductions and reduced risk of CS (*r* = 0.59; RR [95% CI] for a 1% reduction in A1C, 0.66 [0.53–0.83]; *p* = 0.001). However, there was no relationship between reduced A1C and the RRs for postpartum hemorrhage (*r* = 0.37; *p* = 0.49), admission to NICU (*r* = 0.26; *p* = 0.36), RDS (*r* = 0.19; *p* = 0.55), and fetal distress (*r* = 0.48; *p* = 0.16) although significant associations were observed between FPG reductions and risk reductions in these outcomes (RR [95% CI] for 10 mg/dL reductions in FPG, 0.74 [0.57–0.97] [*r* = 0.62, *p* = 0.03] for postpartum hemorrhage; 0.54 [0.34–0.87] [*r* = 0.66, *p* = 0.02] for admission to NICU; 0.56 [0.38–0.85] [*r* = 0.68, *p* = 0.01] for RDS; and 0.49 [0.29–0.84] [*r* = 0.72, *p* = 0.01] for fetal distress).

Significantly lowered RR for 10 mg/dL reductions in 2hPG was observed in four adverse outcomes. In particular, the strength of association of 2hPG reductions expressed as a correlation coefficient (*r*) was prominent for preterm delivery (*r* = 0.73, *p* < 0.001), PIH (*r* = 0.72, *p* = 0.01), and hyperbilirubinemia (*r* = 0.84, *p* < 0.001) compared with that of reductions in the other GC indicators. However, in these four outcomes, the RR for 10 mg/dL reductions in FPG was also significantly lowered. The significantly lowered RR for 10 mg/dL reductions in MBG was observed only in two outcomes (RR [95% CI], 0.71 [0.55–0.91] [*r* = 0.65, *p* = 0.01] for macrosomia; 0.72 [0.55–0.94] [*r* = 0.69, *p* = 0.02] for RDS) partly because the number of data was too small to detect statistical significance.

### 3.6. Sensitivity Analyses of Metaregression

Limiting analyses to trials of patients with GDM did not change the general conclusions partly because the majority of included trials targeted patients with GDM (Table 10). Corresponding scatter plots are presented in Supporting Information 3. We stratified analyses by the geographic regions where trials were conducted (i.e., Asia and Middle East or countries other than in those regions), limiting exposures to reductions in three GC indicators (i.e., A1C, FPG, and 2hPG) and outcomes to six complications (i.e., CS, macrosomia, preterm delivery, PIH, neonatal hypoglycemia, and hyperbilirubinemia) so that there were sufficient data for at least 20 RRs to be regressed for reductions in at least one of the three GC indicators (Table 11 and Supporting Information 4). In the analysis that included trials conducted in Asia and the Middle East, reductions in at least one GC indicator among A1C, FPG, and 2hPG were considered to be positively related to risk reductions in any of the six adverse outcomes. However, no such associations were noted in the analysis of trials conducted in countries other than in Asia or the Middle East. In particular, in Asia and the Middle East, reductions in A1C were positively associated with risk reductions in PIH (RR [95% CI] for 1% of A1C reduction, 0.38 [0.19–0.78] [*r* = 0.70, *p* = 0.01]) but there was a negative association between reductions in A1C and risk reductions in PIH in countries other than in Asia or the Middle East (RR [95% CI] for 1% of A1C reduction, 14.76 [1.36–160.65] [*r* = −0.67, *p* = 0.03]).

## 4. Discussion

The current metaregression analyses showed that reduced blood glucose levels were associated with a risk reduction in 11 of 14 main adverse pregnancy outcomes in patients with diabetes in a dose–response fashion. It can be considered that the improved GC induced by intensive glucose management is associated with an improved prognosis of pregnant women with diabetes. According to Hill's criteria, which is a valid tool for establishing causation, a dose–response gradient suggests that the observed association is causal [84, 85]. It has been suggested that improved GC causes the improved prognosis in pregnant women with diabetes. To support this suggestion, further meta-analyses are needed to examine the dose–response relationship by expanding the included trials to those with interventions such as exercise and supplements (e.g., probiotics [86]) regardless of whether the intervention included intensive glucose management, which was the only intervention examined in the present meta-analysis.

A positive relationship between reductions in blood glucose levels and risk of adverse pregnancy outcomes is pathophysiologically plausible. The hyperglycemia–hyperinsulinemia hypothesis (also known as the Pedersen hypothesis [87, 88]) traditionally proposed that maternal hyperglycemia leads to fetal hyperglycemia, which stimulates maturation and hypertrophy of the fetal pancreas. This results in fetal hyperinsulinemia and neonatal hypoglycemia. Since insulin is a dominant fetal growth hormone, fetal hyperinsulinemia accelerates fetal growth and, therefore, macrosomia is more likely to occur [89]. Blood insulin levels in pregnant women with hyperglycemia are usually high, which can promote increased renal sodium reabsorption and increased blood volume. It can also enhance the response of small blood vessels in the whole body to sympathetic nerve excitation, which results in PIH [90]. In addition, fetal hyperglycemia increases osmotic diuresis which subsequently leads to polyuria and polyhydramnios [91]. Fetal hyperglycemia and hyperinsulinemia can independently cause fetal hypoxia [92]. Polycythemia and hyperbilirubinemia are understood to be counterregulatory mechanisms for this state of hypoxia as it triggers erythropoietin secretion and increased red cell production [93]. In addition to the hyperglycemia–hyperinsulinemia hypothesis, a nonpathophysiological explanation is also applicable to the relationship between GC and risk of preterm delivery. Low socioeconomic status (SES) is generally associated with elevated risk of preterm delivery [94], and individuals with low SES also have worse GC than individuals with a higher SES [95, 96]. The socioeconomic factor could be a mediator between blood glucose levels and risk of preterm delivery.

The American Diabetes Association (ADA) recommends monitoring both FPG and postprandial glucose levels [1]. However, our meta-analysis does not seem to support this recommendation. Our analysis showed that the reduction in FPG could explain risk reductions of any of the pregnancy complications that could be explained by the reduction in 2hPG, suggesting that the major adverse pregnancy outcomes could be covered by monitoring only FPG levels even if 2hPG was not monitored. This suggestion favors the management of glucose levels considering that assessing FPG is simpler and less time-consuming than assessing postprandial glucose. Concerns also have been raised about the reproducibility of postprandial glucose levels [97]. However, monitoring postprandial glucose may be essential for patients with normal FPG but abnormal postprandial glucose levels. Effective monitoring of such patients to prevent pregnancy complications should be further investigated.

In addition, our meta-analysis also did not necessarily support the following ADA statement: “as A1C represents an integrated measure of glucose, it may not fully capture postprandial hyperglycemia, which drives macrosomia. Thus, although A1C may be useful, it should be used as a secondary measure of glycemic control in pregnancy, after blood glucose monitoring” [1]. The current metaregression analysis showed that A1C reduction was more greatly associated with reduction of risks of CS than reductions in FPG, 2hPG, and MBG. Previous cohort studies reported that pre-existing diabetes or GDM itself was associated with CS, irrespective of other medical indications such as macrosomia [98, 99]. The risk for CS could be mediated by clinical practice patterns whereby physicians consider patients with poor GC, which is generally represented by high A1C values rather than high FPG or postprandial plasma glucose values, as having a high-risk pregnancy and refer them to surgeons for CS. However, our results suggest that FPG is superior to A1C for assessing risk of other pregnancy complications. The metaregression analysis (Table 9) indicated that although reductions in FPG were significantly associated with the reduced risk of postpartum hemorrhage, RDS, admission to NICU, and fetal distress, A1C reductions were not significantly associated with any of these outcomes. Similarity of the associations of FPG with RDS, admission to NICU and fetal distress could be explained by the finding that RDS was among the most common causes of NICU admissions and infants with RDS had lower Apgar scores than those without RDS [100]

Interestingly, A1C reductions were associated with the reduced risk of hyperbilirubinemia (*r* = 0.47, *p* = 0.03) (Table 9) but not RDS (*r* = 0.19, *p* = 0.55) (see Results). Conversely, MBG reductions were associated with the reduced risk of RDS (*r* = 0.69, *p* = 0.02) (see Results) but not hyperbilirubinemia (*r* = 0.20, *p* = 0.67) (Table 9). Although both A1C and MBG reflect mean 24-h blood glucose values, A1C reflects average glucose levels over several preceding weeks. It is speculated that the risk of hyperbilirubinemia is influenced by glycemia earlier in pregnancy, whereas that of RDS is more strongly associated with glycemia later in pregnancy. Further research should clarify the stage of pregnancy when GC is critical according to the incidence of pregnancy outcomes.

The risks of some adverse outcomes were not reduced by intensive glucose management. A previous meta-analysis indicated that preconception care was associated with reduced risk of congenital malformation (RR = 0.29) and SGA (RR = 0.52) [101]. However, the current meta-analysis did not show that intensive glucose management significantly reduced the risk of these adverse outcomes. The focus of the interventions in our meta-analysis was during pregnancy, suggesting that even earlier (i.e., before pregnancy) glucose management is required for prevention of congenital malformations and SGA. However, it is possible that intensive glucose management during pregnancy could have been effective but was not statistically proved because the incidence of congenital malformations could have been too low for the detection of statistical significance.

Previous data showed that low blood glucose during pregnancy was associated with an increased risk of having a SGA infant [102, 103]. However, our meta-analysis did not show evidence that intensive glucose management was harmful in terms of bearing a SGA infant. Furthermore, the dose–response data (see Table 9) show that the reduction in 2hPG was borderline associated with reduction of risks for SGA (*p* = 0.050), suggesting that the improvement of GC is favorable for preventing SGA. Not only low blood glucose values but also various risk factors such as gestational hypertension, eclampsia, and preeclampsia were linked to the risk of SGA [104]. Considering that our meta-analysis indicated that reductions in A1C, FPG, and 2hPG were associated with the risk reduction of PIH (Table 9), intensive glucose management possibly prevents SGA via lowering the risk of PIH. However, according to the stratified analysis (Table 9), setting a strict glycemic target elevated the risk of SGA. Excessive GC, which can cause hypoglycemia, may elevate the risk of SGA. However, when GC management is adequate but not excessive, it will not elevate the risk of SGA.

The lack of a relationship between reductions in blood glucose levels and risk of PROM should be addressed although the current meta-analysis indicated that intensive glucose management per se lowered the risk of PROM. Etiology of PROM is multifocal, including demographic and clinical factors such as smoking, previous preterm delivery, and cervical surgery as well as choriodecidual infection [105]. It has been speculated that various risk factors for PROM cannot be ameliorated by improving GC.

Of note, this meta-analysis provides evidence of the relatively low efficacy of intensive glucose management and reduction in blood glucose levels in terms of preventing pregnancy complications for patients with diabetes living in Europe and North America compared with those in Asia and the Middle East. The influence of race or ethnicity on the benefit of glucose management cannot be discussed because, to our knowledge, previous studies have not explored this issue. Nevertheless, the reason for the large differences in results between trials conducted in Asia/Middle East and Europe/North America should be discussed. Reviewing the study characteristics of participants in the included trials (Table 2) showed that the mean BMI was significantly higher in trials conducted in countries other than in Asia and the Middle East compared with those in Asia and the Middle East (mean [SD] (kilogram/square meter unit), 31.6 [3.9] vs. 28.0 [3.7] for BMI at entry (*p* = 0.02 and 26.3 [2.0] vs. 23.9 [1.6] for prepregnancy BMI (*p* = 0.02))). One possible explanation is that, in general, patients living in Europe and North America were more obese and thus had higher risks of adverse pregnancy outcomes than those in Asian countries, irrespective of GC [106]. The large-scale prospective study utilizing the UK Biobank showed that obesity indicators such as BMI, waist circumference, and body fat percentage were associated with higher odds ratios for preeclampsia and gestational hypertension than elevated blood glucose [107]. It is probable that GC has a relatively low priority for preventing adverse pregnancy outcomes in pregnant women living in Europe or North America, where in general obesity is more prevalent than in Asia and the Middle East.

Several limitations should be addressed. First, a large part of included trials targeted patients with GDM. We could not perform a sensitivity metaregression analysis wherein analyses were limited to trials for T1D or T2D patients. Second, the number of data was insufficient to detect statistical significance for some outcomes. Third, criteria for adverse outcomes such as macrosomia and fetal distress varied among trials, and failure to standardize these criteria is of concern. Fourth, there was statistically significant publication bias for some of the adverse pregnancy outcomes. However, its impact could not discussed because, unfortunately, there is no method to adjust for publication bias in a metaregression analysis.

In conclusion, the current results indicated that risk reductions of the majority of pregnancy complications in diabetes depend on improved GC induced by intensive glucose management. To support that such improvement in GC improves the prognosis of pregnant women with diabetes, further meta-analyses are needed expand to any intervention whether or not it includes intensive glucose management.

## Figures and Tables

**Table 1:** Search strategy.

S1	(gestational NEAR/15 hyperglycemia) OR “gestational diabete∗” OR (gestational NEAR/15 glucose) OR (pregnan∗ NEAR/15 hyperglycemia) OR (pregnan∗ NEAR/15 diabete∗) OR (pregnan∗ NEAR/15 glucose) OR “maternal hyperglycemia” OR “maternal diabete∗” OR (maternal NEAR/15 glucose) OR IADPSG OR DIPSI OR ADIPS OR NDDG OR “carpenter Coustan”

S2	(EMB.EXACT.EXPLODE(“Glucose Blood Level”) OR EMB.EXACT.EXPLODE(“Hyperglycemia”) OR EMB.EXACT.EXPLODE(“Glycosylated Hemoglobin”) OR MESH.EXACT.EXPLODE(“Blood Glucose”) OR MESH.EXACT.EXPLODE(“Hyperglycemia”) OR MESH.EXACT.EXPLODE(“Hemoglobin A, Glycosylated”)

S3	“blood glucos∗” OR hyperglycemi∗ OR “hemoglobin∗ A” OR HbA1C OR “Hb A” OR “HbA 1c” OR HbA OR A1Cs OR (glucose PRE/3 test) OR OGTT OR GTT OR (fasting PRE/3 glucose) OR FPG OR FBG OR ((postprandial OR post-prandial) PRE/3 (glucose OR sugar)) OR “1hBG” OR “2hBG” OR “1hPG” OR “2hPG” OR (glycosylated PRE/6 hemoglobin∗) OR “blood glucos∗” OR “blood sugar” OR hyperglycaemi∗ OR “haemoglobin∗ A” OR HbA1C OR “Hb A” OR “HbA 1c” OR HbA OR A1Cs

S4	((intensi∗ OR conventional∗ OR regular OR strict∗ OR tight OR usual OR routin∗ OR standard) NEAR/6 (control∗ OR therap∗ OR treatment OR intervention∗ OR management∗ OR care)) OR “intervention group” OR “control group” OR (intervention AND control) OR monitor∗

S5	(S2 OR S3) AND S4

S6	(pregnan∗ NEAR/5 outcome∗) OR (obstetric∗ NEAR/5 complicat∗) OR (pregnan∗ NEAR/5 disorder∗) OR (obstetric∗ NEAR/5 outcome∗) OR “spontaneous miscarriage” OR “spontaneous abortion” OR “pregnancy loss” OR “antepartum hemorrhage” OR “post-partum hemorrhage” OR “postpartum hemorrhage” OR (Induc∗ NEAR/3 (labor OR labour)) OR “Instrumental birth” OR “caesarean section” OR “cesarean section” OR “shoulder dystocia” OR “pre-eclampsia” OR “preeclampsia” OR eclampsia OR “placenta previa” OR “placental abruption” OR (“premature rupture” PRE/3 membrane∗) OR PROM OR infection OR “amniotic embolism” OR “hyperemesis gravidarum”

S7	mortality OR death OR stillbirth OR congenital OR malformation OR anomaly OR impair∗ OR disabilit∗ OR prematur∗ OR preter∗ OR macrosomia OR “low birth weight” OR lbw OR “large for gestational age” OR LGA OR “small for gestational age” OR SGA OR (neonat∗ NEAR/3 hypoglyc∗) OR (neonat∗ NEAR/3 hyperbili∗) OR jaundice OR “respiratory distress syndrome” OR RDS OR “hyaline membrane disease” OR HMD OR Apgar OR asphyxia OR apnoea OR apnea OR “neonatal intensive care unit” OR NICU

S8	EMB.EXACT.EXPLODE(“newborn death”) OR EMB.EXACT.EXPLODE(“fetus death”) OR EMB.EXACT.EXPLODE(“perinatal death”) OR EMB.EXACT.EXPLODE(“embryo death”) OR EMB.EXACT.EXPLODE(“maternal death”)) OR (EMB.EXACT.EXPLODE(“pregnancy disorder”)) OR (EMB.EXACT.EXPLODE(“newborn disease”)) OR (MESH.EXACT.EXPLODE(“Pregnancy Complications”)) OR (MESH.EXACT.EXPLODE(“Congenital Abnormalities”) OR MESH.EXACT.EXPLODE(“Fetal Diseases”) OR MESH.EXACT.EXPLODE(“Infant, Newborn, Diseases”))

S9	S8 OR S7 OR S6

S10	rtype.exact(“Randomized Controlled Trial” OR “Controlled Clinical Trial”) OR subt.exact(“randomized controlled trial” OR “controlled clinical trial” OR “randomized controlled trials as topic”)

S11	ab(placebo∗ OR randomly OR randomized OR randomised OR trial∗ OR group∗) OR ti(placebo∗ OR randomly OR randomized OR randomised OR trial∗ OR group∗)

S12	S11 OR S10

S13	subt.exact(“retrospective study” OR “cohort analysis” OR “case control study” OR “case-control studies” OR “animal experiment” OR “conference abstract” OR “review” OR “animal model” OR “rat” OR “retrospective studies” OR “epidemiology” OR “animals” OR “systematic review” OR “observational study” OR “cross-sectional study” OR “meta analysis” OR “animal tissue” OR “rats” OR “cohort studies” OR “wistar rat” OR “rats, wistar” OR “cross-sectional studies” OR “conference paper” OR “animal cell” OR “animal” OR “animals, newborn” OR “rats, sprague-dawley” OR “case report”))

S14	ti(protocol OR relationship OR relation OR predict∗ OR predictor∗ OR non-randomized OR association OR associated OR “systematic review∗” OR meta-analysis OR cohort OR observational OR rat OR rats OR animal)

S15	S13 OR S14

S16	S1 AND S5 AND S9 AND S12 NOT S15

*Note:* “ti” and “ab” signify that the descriptor terms in parentheses exist in the title and abstract, respectively. [∗] indicate allowing inflections within 10 characters. T1 NEAR/# T2 allows # or less words between the term T1 and the term T2, with the term T1 and the term T2 in any order. T1 PRE/# T2 allows # or less words between the term T1 and the term T2, with the term T1 and the term T2. “EXPLODE” means searching a subject term and all its associated narrower terms while “EXACT” means searching for the specified term or phrase only.

Abbreviations: EMB, thesaurus terms for Embase; MESH, thesaurus terms for MEDLINE; rtype, publication type; subt, subjects.

**Table 2:** Summary of trials included in this meta-analysis.

**Study source**	**Country**	**Type of diabetes**	**Contents of glucose management**	**Drug intervention adjustment of dose**	**No. of patients**		**Age**		**BMI (kg/m** ^**2**^ **)** ^**b**^	
					**I**	**C**	**I**	**C**	**I**	**C**
Feldman (2024) [23]	United States	GDM	Monitor only	No	98	99	34.9	35.0	[26.9]	[27.0]

Xu (2024) [22]	China	GDM	Multifocal	No	248	256	28.6	29.1	[22.5]	[22.1]

Boggess (2023) [24]	United States	Any	Drug (metformin)	Yes	397	397	32.8	33.1	36.4	36.3

Dunne (2023) [26]	Ireland	GDM	Drug (metformin)	Yes	268	267	34.3	34.3	29.9	30.0

Lai (2023) [27]	China	GDM	Monitor only	No	62	62	31.8	31.8	[22.2]	[23.1]

Majewska (2023) [28]	Poland	GDM	Monitor only	No	49	50	33.0	32.0	[24.7]	[23.0]

Thye (2023) [29]	Malaysia	GDM	Monitor only	No	52	52	33.5	33.2	29.6 [27.8]	28.0 [26.0]

Wang (2023) [25]	China	GDM	Multifocal	No	60	60	30.3	30.5	64.5 kg	64.3 kg

Zhou (2023) [30]	China	GDM	Multifocal	No	182	168	30.3	29.9	[23.1]	[23.1]

El-Gamasy (2022) [31]	Egypt	GDM	Multifocal	No	75	75	31.2	31.6	35.2	34.2

Hantrakun (2022) [32]	Thailand	GDM	Drug (metformin)	Yes	40	40	33.1	32.2	[23.3]	[24.1]

He (2022) [33]	China	GDM	Multifocal	No	85	85	27.3	27.6	22.1	22.0

Hong (2022) [34]	Malaysia	GDM	Monitor only	No	52	54	31.6	33.5	28.7	29.1

Jiang (2022) [35]	China	GDM	Multifocal	No	65	65	28.5	28.6	28.5	28.5

Jiao (2022) [36]	China	GDM	Drug (metformin)	Yes	68	68	30.6	30.7	—	—

Lu (2022) [37]	China	GDM	Monitor only	No	44	44	^a^		—	—

Qi (2022) [38]	China	GDM	Multifocal	No	45	45	28.5	28.2	[23.9]	[24.0]

Cao (2021) [39]	China	GDM	Multifocal	No	541	454	29.9	30.0	27.7	27.3

Guo (2021) [40]	China	GDM	Multifocal	No	70	70	29.5	29.8	—	—

Meng (2021) [41]	China	GDM	Multifocal	No	45	48	26.3	27.0	—	—

Tumminia (2021) [42]	Italy	T1D/T2D	Monitor only	No	21	19	31.2	32.4	[24.7]	[26.6]

Wang (2021) [43]	China	GDM	Drug (metformin)	Yes	56	56	31.1	30.6	23.8	23.9

Yew (2021) [44]	Singapore	GDM	Multifocal	No	170	170	32.0	32.2	[25.6]	[25.5]

Feig (2020) [45]	Multi	T2D	Drug (metformin)	Yes	253	249	35.0	34.7	35.0	35.2

Ji (2020) [46]	China	GDM	Multifocal	No	84	85	30.1	30.0	23.5	23.3

Tang (2020) [47]	China	GDM	Multifocal	No	100	80	28.3	29.6	—	—

Wang (2020) [48]	China	GDM	Multifocal	No	42	42	36.8	36.7	31.2	31.4

Zheng (2020) [49]	China	GDM	Drug (metformin)	Yes	42	38	^b^		—	—

Guo (2019) [50]	China	GDM	Multifocal	No	64	60	31.2	30.6	[25.7]	[25.6]

Lane (2019) [51]	United States	GDM	Monitor only	No	11	12	29.9	30.8	33.9	33.7

Scifres (2019) [52]	United States	GDM	Strict target	Yes	30	30	32.8	31.6	36.5	36.1

Batta (2018) [53]	Jordan	GDM	Multifocal	No	51	34	33.8	34.6	—	—

Mackillop (2018) [19]	China	GDM	Monitor only	No	101	102	33.9	33.0	31.1	31.6

Miremberg (2018) [54]	Israel	GDM	Multifocal	No	60	60	31.7	32.2	27.1	27.1

Paramasivam (2018) [55]	Malaysia	GDM	Monitor only	No	25	25	32.6	32.8	31.4	30.3

Voormolen (2018) [56]	Netherlands	Any	Monitor only	No	147	153	33.0	32.0	28.0	29.0
		GDM			54	54				
		T1D			50	56				
		T2D			40	41				

Yang (2018) [57]	China	GDM	Multifocal	No	57	50	31.6	32.2	—	—

Feig (2017) [58]	Multi	T1D	Monitor only	No	108	107	31.4	31.5	26.1	25.3

Liao (2017) [59]	China	GDM	Monitor only	No	200	200	30.9	30.8	[21.8]	[22.3]

Liu (2017) [60]	China	GDM	Multifocal	Yes	60	60	30.6	32.8	—	—

Alfadhli (2016) [61]	KSA	GDM	Monitor only	No	60	62	32.9	34.2	31.1	32.1

Wei (2016) [62]	China	GDM	Monitor only	No	51	55	30.3	30.0	^c^	

Casey (2015) [63]	United States	GDM	Drug (SU)	Yes	189	186	31.3	31.2	[29.0]	[28.9]

Given (2015) [64]	United Kingdom	GDM	Multifocal	No	21	26	33.5	30.1	33.9	32.4

Yang (2014) [65]	China	GDM	Multifocal	Yes	339	361	29.9	29.7	[22.9]	[23.4]

Cordua (2013) [66]	Denmark	T1D	Monitor only	No	27	59	31.0	30.0	[25.1]	[23.5]

Secher (2013) [67]	Denmark	T1D/T2D	Monitor only	No	79	75	32.0	31.0	[25.1]	[24.7]

Cao (2012) [68]	China	GDM	Monitor only	No	127	148	32.2	32.0	22.6	22.6

Homko (2012) [69]	United States	GDM	Multifocal	No	40	40	30.0	30.3	34.1	34.1

Perez-Ferre (2010) [70]	Spain	GDM	Multifocal	No	49	48	33.3	34.2	[28.0]	[29.0]

Murphy (2008) [71]	United States	T1D/T2D	Monitor only	No	38	33	30.9	32.5	27.9	28.4

Homko (2007) [72]	United States	GDM	Multifocal	No	32	25	29.8	29.2	33.4	32.5

Homko (2002) [73]	United States	GDM	Monitor only	No	31	27	29.0	30.3	[27.4]	[27.3]

Nachum (1999) [74]	Israel	Any	Drug (insulin)	Yes	196	196	32.7	32.4	27.8	27.6
		GDM			138	136				
		T1D/T2D			58	60				

Bonomo (1998) [75]	Italy	GDM	Strict target	Yes	71	71	31.5	31.3	25.4	25.8

Garner (1997) [76]	Canada	GDM	Strict target	Yes	149	150	30.7	30.7	[68.9 kg]	[71.2 kg]

Demarini (1994) [77]	United States	T1D	Strict target	Yes	68	69	25.3	26.6	—	—

Thompson (1990) [78]	United States	GDM	Drug (insulin)	Yes	50	45	27.0	26.0	192 I.b.	200 I.b.

Farrag (1987) [79]	KSA	Overt	Strict target	Yes	16	15	—	—	—	—

Goldberg (1986) [80]	United States	GDM	Monitor only	No	58	58	30.4	30.1	—	—

Persson (1985) [81]	Sweden	GDM	Drug (insulin)	Yes	97	105	30.5	29.0	64.7 kg	60.0 kg

Varner (1983) [82]	United States	T1D	Monitor only	No	14	14	24.0	23.3	—	—

*Note:* —, no data.

Abbreviations: BMI, body mass index; BW, body weight; C, control group; GDM, gestational diabetes mellitus; I, intervention group; KSA, Kingdom of Saudi Arabia; MBG, mean blood glucose; multi, multicenter study; overt, overt diabetes; SU, sulfonylurea; T1D, Type 1 diabetes; T2D, Type 2 diabetes.

^a^Thirty-one percent of patients were > 26 years old.

^b^Square parentheses indicate mean BMI or BW of study participants at prepregnancy; otherwise, they indicate BMI or BW at entry.

^c^Twenty-five percent of patients were overweight (prepregnancy BMI ≥ 25 kg/m^2^).

**Table 3:** Summary of glycemic control in the intervention (I) and control (C) groups.

**Study source**	**Mean glucose after intervention**							
	**A1C, %**		**FPG, mg/dL**		**2hPG, mg/dL**		**MBG, mg/dL**	
	**I**	**C**	**I**	**C**	**I**	**C**	**I**	**C**
Feldman (2024) [23]			85.2	87.1	104.1	105.1		

Xu (2024) [22]			84.7	85.2	116.8	121.4		

Boggess (2023) [24]	6.0	6.2						

Dunne (2023) [26]			81.1	84.7				

Lai (2023) [27]	5.3	5.4					96.2	96.2

Majewska (2023) [28]	5.1	5.1						

Thye (2023) [29]	5.4	5.3						

Wang (2023) [25]	8.4	6.9	99.8	112.3	124.0	133.9		

Zhou (2023) [30]	5.3	6.3	87.0	101.1	113.7	128.3		

El-Gamasy (2022) [31]			95.7	113.9			132.4	169.7

Hantrakun (2022) [32]			87.0	86.0	101.0	102.0		

He (2022) [33]			85.8	98.6	104.1	122.0		

Hong (2022) [34]	5.4	5.4						

Jiang (2022) [35]	7.0	8.5	119.8	129.7	130.3	159.6		

Jiao (2022) [36]	5.3	6.3	72.6	99.8	102.5	148.8		

Lu (2022) [37]	5.3	6.4	73.4	96.6	98.5	140.7		

Qi (2022) [38]	5.1	5.8	101.4	116.4	126.3	143.1		

Cao (2021) [39]	5.3	5.4	86.8	89.7				

Guo (2021) [40]			90.5	113.0	128.3	142.9		

Meng (2021) [41]	7.1	7.8	78.6	102.3	120.2	146.3		

Tumminia (2021) [42]	6.2	6.3					111.0	115.0

Wang (2021) [43]	6.1	6.6	106.8	118.4	142.7	158.9		

Yew (2021) [44]							97.3	99.8

Feig (2020) [45]	5.9	6.1	95.5	97.8	113.3	114.6	109.0	113.0

Ji (2020) [46]	6.0	6.6	94.1	109.0	120.0	174.6		

Tang (2020) [47]			78.3	141.0	114.9	156.7		

Wang (2020) [48]	5.8	6.1	91.0	96.2	117.2	122.0		

Zheng (2020) [49]			92.4	102.2	94.4	110.3		

Guo (2019) [50]	4.7	5.3	75.7	77.5	126.1	127.9		

Lane (2019) [51]	5.3	5.3						

Scifres (2019) [52]	5.6	5.5	91.0	95.0	105.0	115.0	101.0	107.0

Batta (2018) [53]	5.2	5.6	83.2	89.9				

Mackillop (2018) [19]	5.5	5.4	94.3	94.4				

Miremberg (2018) [54]							112.6	105.1

Paramasivam (2018) [55]	5.2	5.6	82.9	85.8	106.3	104.5		

Voormolen (2018) [56]	6.8	7.0						

Yang (2018) [57]			77.7	95.7	103.8	125.0		

Feig (2017) [58]	6.4	6.5					120.7	126.1

Liao (2017) [59]	5.3	5.7	94.9	100.9	110.8	140.2		

Liu (2017) [60]			105.8	132.3	127.6	158.4		

Alfadhli (2016) [61]	5.7	6.1	84.9	89.9	103.1	113.0		

Wei (2016) [62]	5.5	5.6						

Casey (2015) [63]							84.0	87.0

Given (2015) [64]	5.3	5.3					111.7	111.7

Yang (2014) [65]	5.2	5.2						

Cordua (2013) [66]	6.0	6.2					100.9	97.3

Secher (2013) [67]	6.0	6.1					111.7	111.7

Cao (2012) [68]			89.5	94.5	120.0	127.6		

Homko (2012) [69]			91.5	94.3			107.4	109.7

Perez-Ferre (2010) [70]	5.3	5.4						

Murphy (2008) [71]	5.8	6.4						

Homko (2007) [72]	6.1	6.2	90.8	88.6	108.4	110.9	106.6	104.5

Homko (2002) [73]			85.5	89.4				

Nachum (1999) [74]	5.7	6.1					97.9	102.6
	5.5	5.8					97.7	100.9
	6.2	6.7					98.6	106.3

Bonomo (1998) [75]	4.4	4.4	82.4	91.5	101.1	110.0		

Garner (1997) [76]			80.5	84.6				

Demarini (1994) [77]	5.4^a^	5.5	125.9	132.1				

Thompson (1990) [78]			81.3	79.7	94.1	93.8		

Farrag (1987) [79]							90.1	151.4

Goldberg (1986) [80]			98.0	104.0	182.0	177.0		

Persson (1985) [81]	6.8	7.0					105.8	111.8

Varner (1983) [82]	7.2	7.6						

*Note:* —, no data. Another six trials [26, 54, 57, 73, 76, 80] and one trial [77], respectively, used 1-h and 1.5-h postprandial glucose as the GC indicator; values were excluded in this meta-analysis because of insufficient number of data.

Abbreviations: 2hPG, 2-h postprandial glucose; A1C, hemoglobin A1c; FPG, fasting plasma glucose; MBG, mean blood glucose.

^a^Hemoglobin A1 (A1) was used and it was converted into A1C using the following formula with 0.98 correlation coefficient [83]: A1C value = (A1 value − 1.01)/1.20.

**Table 4:** Adverse pregnancy outcomes identified in this meta-analysis.

	**No. of observations**	**No. of events**
(a) Maternal complications		
Cesarean section (53 trials) [19, 22–24, 26–36, 38, 39, 41, 42, 44–46, 48–51, 53–59, 61–76, 78–80, 82]	10,752	4802
Preterm delivery (46 trials) [19, 22–27, 29–42, 44–49, 51, 54–61, 64–72, 79]	9417	1344
Polyhydramnios (18 trials) [22, 25, 30, 33, 35–38, 40, 41, 46, 48, 49, 51, 54, 59–61]	2908	153
Oligohydramnios (4 trials) [22, 24, 30, 37]	1736	23
Pregnancy-induced hypertension (37 trials) [19, 22–27, 29–32, 34, 36, 37, 39, 41, 45, 47, 48, 51, 54, 56–59, 63–65, 67–72, 74, 77, 79]	8855	1057
Premature rupture of membranes (16 trials) [22, 24, 30, 31, 37–39, 41, 43, 49, 57, 59, 61, 65, 69, 72]	4693	480
Placental abruption (4 trials) [30, 39, 70, 72]	1499	11
Maternal infection (13 trials) [24–26, 30, 31, 35, 41, 43, 45, 47, 59, 60, 79]	3503	269
Chorioamnionitis (4 trials) [23, 30, 63, 69]	996	125
Postpartum hemorrhage (15 trials) [22–26, 29–31, 34, 40, 41, 47, 49, 59, 60]	3856	266
Shoulder dystocia (18 trials) [19, 23, 24, 26, 29, 34, 45, 50, 51, 54, 56, 58, 61, 63–65, 70, 78]	4491	40
Perineal trauma (8 trials) [19, 29, 31, 34, 51, 54, 61, 63]	1121	15
Anemia (2 trials) [32, 37]	168	14
Death (4 trials) [23, 24, 29, 56]	1383	8
Antepartum hemorrhage (2 trials) [26, 31]	674	54
Fetal growth restriction (5 trials) [22, 24, 31, 32, 59]	1928	23
Placenta previa (4 trials) [22, 24, 34, 41]	1496	5
Amniotic fluid turbidity (2 trials) [22, 32]	854	31
ICU (3 trials) [19, 23, 61]	485	9

(b) Fetal/neonatal complications		
Macrosomia (55 trials) [19, 22–28, 30–32, 34–52, 55–59, 61–76, 78–81]	11,021	1546
Neonatal hypoglycemia (47 trials) [19, 22–24, 26–28, 30–32, 35–39, 41, 42, 44, 45, 48–50, 52, 55–74, 76, 78, 81, 82]	10,009	1276
Admission to neonatal intensive care unit (27 trials) [19, 23, 24, 26, 27, 29–32, 34, 44, 45, 51, 52, 55–59, 61, 63, 64, 68, 69, 71–73]	5530	1055
Respiratory distress syndrome (26 trials) [23, 24, 26, 27, 31, 32, 36, 39, 40, 42–45, 51, 52, 58, 59, 61, 64, 68, 69, 72–74, 79, 81]	5935	457
Fetal distress (22 trials) [22, 26, 30–33, 35, 37–41, 44, 48, 59–61, 65, 70, 74, 75, 77]	5793	247
Hyperbilirubinemia (35 trials) [19, 22, 24, 26, 27, 30, 32, 35–37, 39, 41–45, 47, 49, 52, 55, 57, 58, 60, 61, 63, 64, 68, 69, 72–74, 76, 78, 81, 82]	7674	1151
Miscarriage (12 trials) [22, 24, 31, 37, 45, 58, 59, 61, 67, 69, 71, 82]	3050	43
Stillbirth (15 trials) [22, 24, 26, 29, 31, 39, 45, 46, 58, 61, 63, 64, 68, 73, 76]	5074	41
Neonatal death (24 trials) [22–24, 26, 30, 31, 44, 45, 55, 56, 59, 61, 63, 68, 69, 71, 72, 74–76, 78, 79, 81, 82]	6201	33
Small for gestational age (27 trials) [22, 24, 26–31, 34, 42, 45, 51, 52, 55, 56, 58, 59, 61–65, 70, 71, 74, 75, 81]	6320	343
Congenital malformation (22 trials) [22, 25, 26, 30, 35, 41, 45–47, 49, 55, 56, 58, 61, 64, 65, 68, 69, 71, 72, 74, 76]	5146	109
Birth injury (13 trials) [24, 26, 27, 29, 34, 44, 45, 58, 61, 63, 73, 74, 76]	3828	92
Hypocalcemia (8 trials) [24, 70, 74, 76–78, 81, 82]	2017	162
Polycythemia (5 trials) [32, 70, 74, 81, 82]	801	32
Pneumonia (6 trials) [22, 30, 41, 47, 59, 60]	1647	111
Encephalopathy (2 trials) [23, 41]	290	1
Anemia (2 trials) [22, 32]	584	2

**Table 5:** Descriptions of maternal and fetal/neonatal complications.

**Study source**	**Maternal complications**	**Fetal/neonatal complications**
Feldman (2024) [23]	8 outcomes: CS, preterm delivery, PIH, chorioamnionitis, postpartum hemorrhage, shoulder dystocia, maternal death, admission to ICU	6 outcomes: Macrosomia, neonatal hypoglycemia, admission to NICU, RDS, neonatal death, encephalopathy
Xu (2024) [22]	10 outcomes: CS, preterm delivery, polyhydramnios, oligohydramnios, PIH, PROM, postpartum hemorrhage, IUGR, placenta previa, amniotic fluid turbidity	11 outcomes: Macrosomia, neonatal hypoglycemia, fetal distress, hyperbilirubinemia, miscarriage, stillbirth, neonatal death, SGA, congenital malformation, pneumonia, anemia
Boggess (2023) [24]	11 outcomes: CS, preterm delivery, oligohydramnios, PIH, PROM, maternal infection, postpartum hemorrhage, shoulder dystocia, maternal death, IUGR, placenta previa	11 outcomes: Macrosomia, neonatal hypoglycemia, admission to NICU, RDS, hyperbilirubinemia, miscarriage, stillbirth, neonatal death, SGA, birth injury, hypocalcemia
Dunne (2023) [26]	7 outcomes: CS, preterm delivery, PIH, maternal infection, postpartum hemorrhage, shoulder dystocia, antepartum hemorrhage	11 outcomes: Macrosomia, neonatal hypoglycemia, admission to NICU, RDS, fetal distress, hyperbilirubinemia, stillbirth, neonatal death, SGA, congenital malformation, birth injury
Lai (2023) [27]	3 outcomes: CS, preterm delivery, PIH	7 outcomes: Macrosomia, neonatal hypoglycemia, admission to NICU, RDS, hyperbilirubinemia, SGA, birth injury
Majewska (2023) [28]	1 outcome: CS	3 outcomes: Macrosomia, neonatal hypoglycemia, SGA
Thye (2023) [29]	7 outcomes: CS, preterm delivery, PIH, postpartum hemorrhage, shoulder dystocia, perineal trauma, maternal death	4 outcomes: Admission to NICU, stillbirth, SGA, birth injury
Wang (2023) [25]	5 outcomes: Preterm delivery, polyhydramnios, PIH, maternal infection, postpartum hemorrhage	2 outcomes: Macrosomia, congenital malformation
Zhou (2023) [30]	11 outcomes: CS, preterm delivery, polyhydramnios, oligohydramnios, PIH, PROM, placental abruption, maternal infection, chorioamnionitis, postpartum hemorrhage, amniotic fluid turbidity	9 outcomes: Macrosomia, neonatal hypoglycemia, admission to NICU, fetal distress, hyperbilirubinemia, neonatal death, SGA, congenital malformation, pneumonia
El-Gamasy (2022) [31]	9 outcomes: CS, preterm delivery, PIH, PROM, maternal infection, postpartum hemorrhage, perineal trauma, antepartum hemorrhage, IUGR	9 outcomes: Macrosomia, neonatal hypoglycemia, admission to NICU, RDS, fetal distress, miscarriage, stillbirth, neonatal death, SGA
Hantrakun (2022) [32]	5 outcomes: CS, preterm delivery, PIH, maternal anemia, IUGR	8 outcomes: Macrosomia, neonatal hypoglycemia, admission to NICU, RDS, fetal distress, hyperbilirubinemia, polycythemia, anemia
He (2022) [33]	3 outcomes: CS, preterm delivery, polyhydramnios	1 outcome: Fetal distress
Hong (2022) [34]	7 outcomes: CS, preterm delivery, PIH, postpartum hemorrhage, shoulder dystocia, perineal trauma, placenta previa	4 outcomes: Macrosomia, admission to NICU, SGA, birth injury
Jiang (2022) [35]	4 outcomes: CS, preterm delivery, polyhydramnios, maternal infection	5 outcomes: Macrosomia, neonatal hypoglycemia, fetal distress, hyperbilirubinemia, congenital malformation
Jiao (2022) [36]	4 outcomes: CS, preterm delivery, polyhydramnios, PIH	4 outcomes: Macrosomia, neonatal hypoglycemia, RDS, hyperbilirubinemia
Lu (2022) [37]	6 outcomes: Preterm delivery, polyhydramnios, oligohydramnios, PIH, PROM, maternal anemia	5 outcomes: Macrosomia, neonatal hypoglycemia, fetal distress, hyperbilirubinemia, miscarriage
Qi (2022) [38]	4 outcomes: CS, preterm delivery, polyhydramnios, PROM	3 outcomes: Macrosomia, neonatal hypoglycemia, fetal distress
Cao (2021) [39]	5 outcomes: CS, preterm delivery, PIH, PROM, placental abruption	6 outcomes: Macrosomia, neonatal hypoglycemia, RDS, fetal distress, hyperbilirubinemia, stillbirth
Guo (2021) [40]	3 outcomes: Preterm delivery, polyhydramnios, postpartum hemorrhage	3 outcomes: Macrosomia, RDS, fetal distress
Meng (2021) [41]	8 outcomes: CS, preterm delivery, polyhydramnios, PIH, PROM, maternal infection, postpartum hemorrhage, placenta previa	7 outcomes: Macrosomia, neonatal hypoglycemia, fetal distress, hyperbilirubinemia, congenital malformation, pneumonia, encephalopathy
Tumminia (2021) [42]	2 outcomes: CS, preterm delivery	5 outcomes: Macrosomia, neonatal hypoglycemia, RDS, hyperbilirubinemia, SGA
Wang (2021) [43]	2 outcomes: PROM, maternal infection	3 outcomes: Macrosomia, RDS, hyperbilirubinemia
Yew (2021) [44]	2 outcomes: CS, preterm delivery	8 outcomes: Macrosomia, neonatal hypoglycemia, admission to NICU, RDS, fetal distress, hyperbilirubinemia, neonatal death, birth injury
Feig (2020) [45]	5 outcomes: CS, preterm delivery, PIH, maternal infection, shoulder dystocia	11 outcomes: Macrosomia, neonatal hypoglycemia, admission to NICU, RDS, hyperbilirubinemia, miscarriage, stillbirth, neonatal death, SGA, congenital malformation, birth injury
Ji (2020) [46]	3 outcomes: CS, preterm delivery, polyhydramnios	3 outcomes: Macrosomia, stillbirth, congenital malformation
Tang (2020) [47]	4 outcomes: Preterm delivery, PIH, maternal infection, postpartum hemorrhage	4 outcomes: Macrosomia, hyperbilirubinemia, congenital malformation, pneumonia
Wang (2020) [48]	4 outcomes: CS, preterm delivery, polyhydramnios, PIH	3 outcomes: Macrosomia, neonatal hypoglycemia, fetal distress
Zheng (2020) [49]	5 outcomes: CS, preterm delivery, polyhydramnios, PROM, postpartum hemorrhage	4 outcomes: Macrosomia, neonatal hypoglycemia, hyperbilirubinemia, congenital malformation
Guo (2019) [50]	2 outcomes: CS, shoulder dystocia	2 outcomes: Macrosomia, neonatal hypoglycemia
Lane (2019) [51]	6 outcomes: CS, preterm delivery, polyhydramnios, PIH, shoulder dystocia, perineal trauma	4 outcomes: Macrosomia, admission to NICU, RDS, SGA
Scifres (2019) [52]	None	6 outcomes: Macrosomia, neonatal hypoglycemia, admission to NICU, RDS, hyperbilirubinemia, SGA
Batta (2018) [53]	1 outcome: CS	None
Mackillop (2018) [19]	6 outcomes: CS, preterm delivery, PIH, shoulder dystocia, perineal trauma, admission to ICU	4 outcomes: Macrosomia, neonatal hypoglycemia, admission to NICU, hyperbilirubinemia
Miremberg (2018) [54]	6 outcomes: CS, preterm delivery, polyhydramnios, PIH, shoulder dystocia, perineal trauma	None
Paramasivam (2018) [55]	2 outcomes: CS, preterm delivery	7 outcomes: Macrosomia, neonatal hypoglycemia, admission to NICU, hyperbilirubinemia, neonatal death, SGA, congenital malformation
Voormolen (2018) [56]	5 outcomes: CS, preterm delivery, PIH, shoulder dystocia, maternal death	6 outcomes: Macrosomia, neonatal hypoglycemia, admission to NICU, neonatal death, SGA, congenital malformation
Yang (2018) [57]	4 outcomes: CS, preterm delivery, PIH, PROM	4 outcomes: Macrosomia, neonatal hypoglycemia, admission to NICU, hyperbilirubinemia
Feig (2017) [58]	4 outcomes: CS, preterm delivery, PIH, shoulder dystocia	10 outcomes: Macrosomia, neonatal hypoglycemia, admission to NICU, RDS, hyperbilirubinemia, miscarriage, stillbirth, SGA, congenital malformation, birth injury
Liao (2017) [59]	8 outcomes: CS, preterm delivery, polyhydramnios, PIH, PROM, maternal infection, postpartum hemorrhage, IUGR	9 outcomes: Macrosomia, neonatal hypoglycemia, admission to NICU, RDS, fetal distress, miscarriage, neonatal death, SGA, pneumonia
Liu (2017) [60]	4 outcomes: Preterm delivery, polyhydramnios, maternal infection, postpartum hemorrhage	4 outcomes: Neonatal hypoglycemia, fetal distress, hyperbilirubinemia, pneumonia
Alfadhli (2016) [61]	7 outcomes: CS, preterm delivery, polyhydramnios, PROM, shoulder dystocia, perineal trauma, admission to ICU	12 outcomes: Macrosomia, neonatal hypoglycemia, admission to NICU, RDS, fetal distress, hyperbilirubinemia, miscarriage, stillbirth, neonatal death, SGA, congenital malformation, birth injury
Wei (2016) [62]	1 outcome: CS	3 outcomes: Macrosomia, neonatal hypoglycemia, SGA
Casey (2015) [63]	5 outcomes: CS, PIH, chorioamnionitis, shoulder dystocia, perineal trauma	8 outcomes: Macrosomia, neonatal hypoglycemia, admission to NICU, hyperbilirubinemia, stillbirth, neonatal death, SGA, birth injury
Given (2015) [64]	4 outcomes: CS, preterm delivery, PIH, shoulder dystocia	8 outcomes: Macrosomia, neonatal hypoglycemia, admission to NICU, RDS, hyperbilirubinemia, stillbirth, SGA, congenital malformation
Yang (2014) [65]	5 outcomes: CS, preterm delivery, PIH, PROM, shoulder dystocia	5 outcomes: Macrosomia, neonatal hypoglycemia, fetal distress, SGA, congenital malformation
Cordua (2013) [66]	2 outcomes: CS, preterm delivery	2 outcomes: Macrosomia, neonatal hypoglycemia
Secher (2013) [67]	3 outcomes: CS, preterm delivery, PIH	3 outcomes: Macrosomia, neonatal hypoglycemia, miscarriage
Cao (2012) [68]	3 outcomes: CS, preterm delivery, PIH	8 outcomes: Macrosomia, neonatal hypoglycemia, admission to NICU, RDS, hyperbilirubinemia, stillbirth, neonatal death, congenital malformation
Homko (2012) [69]	5 outcomes: CS, preterm delivery, PIH, PROM, chorioamnionitis	8 outcomes: Macrosomia, neonatal hypoglycemia, admission to NICU, RDS, hyperbilirubinemia, miscarriage, neonatal death, congenital malformation
Perez-Ferre (2010) [70]	5 outcomes: CS, preterm delivery, PIH, placental abruption, shoulder dystocia	6 outcomes: Macrosomia, neonatal hypoglycemia, fetal distress, SGA, hypocalcemia, polycythemia
Murphy (2008) [71]	3 outcomes: CS, preterm delivery, PIH	7 outcomes: Macrosomia, neonatal hypoglycemia, admission to NICU, miscarriage, neonatal death, SGA, congenital malformation
Homko (2007) [72]	5 outcomes: CS, preterm delivery, PIH, PROM, placental abruption	7 outcomes: Macrosomia, neonatal hypoglycemia, admission to NICU, RDS, hyperbilirubinemia, neonatal death, congenital malformation
Homko (2002) [73]	1 outcome: CS	7 outcomes: Macrosomia, neonatal hypoglycemia, admission to NICU, RDS, hyperbilirubinemia, stillbirth, birth injury
Nachum (1999) [74]	2 outcomes: CS, PIH	11 outcomes: Macrosomia, neonatal hypoglycemia, RDS, fetal distress, hyperbilirubinemia, neonatal death, SGA, congenital malformation, birth injury, hypocalcemia, polycythemia
Bonomo (1998) [75]	1 outcome: CS	4 outcomes: Macrosomia, fetal distress, neonatal death, SGA
Garner (1997) [76]	1 outcome: CS	8 outcomes: Macrosomia, neonatal hypoglycemia, hyperbilirubinemia, stillbirth, neonatal death, congenital malformation, birth injury, hypocalcemia
Demarini (1994) [77]	1 outcome: PIH	2 outcomes: Fetal distress, hypocalcemia
Thompson (1990) [78]	2 outcomes: CS, shoulder dystocia	5 outcomes: Macrosomia, neonatal hypoglycemia, hyperbilirubinemia, neonatal death, hypocalcemia
Farrag (1987) [79]	4 outcomes: CS, preterm delivery, PIH, maternal infection	3 outcomes: Macrosomia, RDS, neonatal death
Goldberg (1986) [80]	1 outcome: CS	1 outcome: Macrosomia
Persson (1985) [81]	None	8 outcomes: Macrosomia, neonatal hypoglycemia, RDS, hyperbilirubinemia, neonatal death, SGA, hypocalcemia, polycythemia
Varner (1983) [82]	1 outcome: CS	6 outcomes: Neonatal hypoglycemia, hyperbilirubinemia, miscarriage, neonatal death, hypocalcemia, polycythemia

Abbreviations: CS, cesarean section; ICU, intensive care unit; IUGR, intrauterine growth restriction; NICU, neonatal intensive care unit; PIH, pregnancy-induced hypertension; PROM, premature rapture of membranes; RDS, respiratory distress syndrome; SGA, small for gestational age.

**Table 6:** Assessment of study quality using the revised Cochrane risk-of-bias tool for randomized trials (RoB 2).

**Study source**	**R**	**D**	**Mi**	**Me**	**S**	**Overall**
Feldman (2024) [23]	?	L	?	L	L	?
Xu (2024) [22]	L	L	L	L	L	L
Boggess (2023) [24]	L	L	H	L	L	H
Dunne (2023) [26]	L	L	L	L	L	L
Lai (2023) [27]	H	H	L	L	L	H
Majewska (2023) [28]	L	L	H^a^	L	L	H
Thye (2023) [29]	L	L	?	L	L	L
Wang (2023) [25]	H	L	L	L	L	H
Zhou (2023) [30]	L	H	L	L	L	L
El-Gamasy (2022) [31]	?	L	L	L	L	?
Hantrakun (2022) [32]	L	L	L	L	L	L
He (2022) [33]	?	L	L	L	?	?
Hong (2022) [34]	L	L	L	L	L	L
Jiang (2022) [35]	?	L	L	L	L	?
Jiao (2022) [36]	?	L	L	L	L	?
Lu (2022) [37]	?	L	L	L	L	?
Qi (2022) [38]	H	L	L	L	L	H
Cao (2021) [39]	?	L	L	L	?	?
Guo (2021) [40]	?	L	L	L	?	?
Meng (2021) [41]	?	L	L	L	L	?
Tumminia (2021) [42]	L	L	L	L	L	L
Wang (2021) [43]	L	L	L	L	L	L
Yew (2021) [44]	L	L	L	L	H	H
Feig (2020) [45]	L	L	L	L	L	L
Ji (2020) [46]	L	L	L	L	L	L
Tang (2020) [47]	H	L	L	L	?	H
Wang (2020) [48]	L	L	L	L	?	?
Zheng (2020) [49]	H	L	L	L	L	H
Guo (2019) [50]	L	L	L	L	?	?
Lane (2019) [51]	L	H	L	L	L	H
Scifres (2019) [52]	L	L	L	L	?	?
Batta (2018) [53]	L	L	L	L	H	H
Mackillop (2018) [19]	L	L	L	L	L	L
Miremberg (2018) [54]	L	L	L	L	L	L
Paramasivam (2018) [55]	L	H	L	L	L	H
Voormolen (2018) [56]	L	L	L	L	L	L
Yang (2018) [57]	H	L	L	L	?	H
Feig (2017) [58]	L	L	L	L	L	L
Liao (2017) [59]	H	L	L	L	?	H
Liu (2017) [60]	?	L	L	L	L	?
Alfadhli (2016) [61]	L	H	H^a^	L	H	H
Wei (2016) [62]	?	H	L	L	L	H
Casey (2015) [63]	L	L	L	L	H	H
Given (2015) [64]	L	L	L	L	H	H
Yang (2014) [65]	?	L	L	L	H	H
Cordua (2013) [66]	L	L	L	L	L	L
Secher (2013) [67]	L	L	L	L	L	L
Cao (2012) [68]	?	L	**?**	L	L	?
Homko (2012) [69]	?	L	L	L	L	?
Perez-Ferre (2010) [70]	?	H	L	L	?	H
Murphy (2008) [71]	L	L	L	L	H	H
Homko (2007) [72]	?	L	L	L	L	?
Homko (2002) [73]	?	L	L	L	?	?
Nachum (1999) [74]	L	L	L	L	?	?
Bonomo (1998) [75]	H^a^	L	L	L	L	H
Garner (1997) [76]	?	L	L	L	H	H
Demarini (1994) [77]	?	H	?	L	?	H
Thompson (1990) [78]	L	H	L	L	L	H
Farrag (1987) [79]	?	L	L	L	?	?
Goldberg (1986) [80]	H	L	L	L	H	H
Persson (1985) [81]	?	L	L	L	?	?
Varner (1983) [82]	L	L	L	L	L	L

*Note:* Five domains for assessing risk of bias are as follows: R: bias arising from the randomization process; D, bias due to deviations from intended interventions; Mi, bias due to missing outcome data; Me, bias in measurement of the outcome; S, bias in selection of the reported result. O indicates overall risk of bias. “L” and “H**”** indicate low and high risk of bias, respectively. “?” indicates some concerns about risk of bias.

^a^Reasons for the determination of a high risk of bias were the use of only age and BMI as the limiting factors in the restricted randomization (R domain) [75] and a failure to refer to missing data that would influence the study results (Mi domain) [28, 61].

**Table 7:** Overall relative risk (RR) with 95% confidence interval (CI) and test of publication bias for each adverse outcome.

**Maternal outcomes**	**No.**	**RR (95% CI)** ^**b**^	**χ** ^2^	**p** ** for ** **χ** ^2^	**I** ^2^	**Tau**	**Z**	**p** ** for ** **Z**	**Begg**	**Egger**	**Trim**–**fill**^**c**^		
											**Fill**	**Unadjusted**	**Adjusted**

Cesarean section	53	**0.91 (0.85–0.97)**	**104.3**	**< 0.001**	50.1%	0.021	**2.84**	**0.004**	0.63	**0.08**	**2**	**0.94 (0.90–0.98)**	**0.94 (0.91–0.98)**
Preterm delivery	46	**0.76 (0.65–0.88)**	**66.7**	**0.02**	**32.5%**	0.055	**3.65**	**< 0.001**	**0.09**	**0.009**	**10**	**0.78 (0.68–0.89)**	**0.84 (0.72–0.99)**
Polyhydramnios	16 (18)	**0.25 (0.17–0.37)**	8.3	0.92	0.0%	—	**7.16**	**< 0.001**	0.26	**0.006**	**6**	**0.28 (0.19–0.41)**	**0.35 (0.25–0.50)**
Oligohydramnios	3 (4)	0.61 (0.27–1.38)	**0.4**	**0.81**	**0.0%**	—	1.20	0.23	1.00	0.90			
PIH (preeclampsia/GH)^a^	37	**0.82 (0.67–0.99)**	**68.9**	**0.01**	**47.7%**	0.122	**2.03**	**0.04**	0.27	**0.02**	**5**	**0.81 (0.67–0.99)**	0.86 (0.71–1.05)
PROM	15 (16)	**0.65 (0.55–0.77)**	18.4	0.19	23.9%	—	**5.11**	**< 0.001**	1.00	0.40	**2**	**0.66 (0.55–0.79)**	**0.68 (0.57–0.80)**
Placental abruption	4	0.74 (0.25–2.22)	1.0	0.81	0.0%	—	0.53	0.60	0.73	0.95			
Maternal infection	13	**0.51 (0.40–0.64)**	19.8	0.07	39.4%	—	**5.58**	**< 0.001**	**0.08**	**0.09**	**4**	**0.53 (0.41–0.67)**	**0.58 (0.46–0.73)**
Chorioamnionitis	4	**0.63 (0.46–0.86)**	0.4	0.93	0.0%	—	**2.91**	**0.004**	1.00	0.88			
Postpartum hemorrhage	15	**0.62 (0.50–0.78)**	20.6	0.11	31.9%	—	**4.09**	**< 0.001**	0.20	**0.08**	**4**	**0.67 (0.53–0.85)**	**0.74 (0.59–0.93)**
Shoulder dystocia	8 (18)	1.10 (0.61–1.97)	3.0	0.89	0.0%	—	−0.31	0.76	0.71	0.60			
Perineal trauma	4 (8)	1.08 (0.41–2.88)	4.2	0.24	28.5%	—	−0.16	0.87	0.73	0.60			
Maternal anemia	2	0.56 (0.19–1.59)	1.0	0.32	0.0%	—	1.09	0.27	1.00	—^d^			
Maternal death	2 (4)	0.60 (0.15–2.51)	1.6	0.21	36.6%	—	0.69	0.49	1.00	—^d^			
Antepartum hemorrhage	2	0.59 (0.35–1.001)	0.2	0.70	0.0%	—	1.96	0.050	1.00	—^d^			
Fetal growth restriction	4 (5)	0.53 (0.23–1.24)	1.9	0.39	0.0%	—	1.43	0.15	0.31	0.11			
Placenta previa	3 (4)	2.54 (0.49–13.07)	0.6	0.74	0.0%	—	−1.11	0.26	1.00	0.44			
Amniotic fluid turbidity	1 (2)	**0.38 (0.18–0.80)**	—^e^	—^e^	—^e^	—	**2.56**	**0.01**	—^e^	—^d^			
Admission to ICU	3	0.50 (0.14–0.79)	0.3	0.86	0.0%	—	1.06	0.29	1.00	0.84			

**Fetal/neonatal outcomes**	**N**	**RR (95% CI)**	**χ** ^2^	**p** ** for ** **χ** ^2^	**I** ^2^	**Tau**	**Z**	**p** ** for ** **Z**	**Begg**	**Egger**		**Trim–fill**	
											**Fill**	**Unadjusted**	**Adjusted**

Macrosomia [LGA]^a^	55	**0.70 (0.61–0.81)**	**95.0**	**< 0.001**	**43.1%**	0.097	**4.71**	**< 0.001**	**0.07**	**0.03**	**1**	**0.70 (0.61–0.81)**	**0.71 (0.61–0.82)**
Neonatal hypoglycemia	46	**0.75 (0.64–0.88)**	**71.6**	**0.01**	**37.1%**	0.074	**3.51**	**< 0.001**	0.29	**0.004**	**9**	**0.74 (0.63–0.88)**	0.84 (0.70–1.02)
Admission to NICU	27	**0.82 (0.74–0.90)**	32.0	0.19	18.8%	—	**3.77**	**< 0.001**	0.80	0.19			
RDS	26	**0.82 (0.69–0.97)**	30.0	0.23	16.5%	—	**2.28**	**0.02**	**0.06**	**0.04**	**8**	0.86 (0.72–1.03)	0.99 (0.84–1.17)
Fetal [neonatal] distress^a^	21 (22)	**0.46 (0.31–0.67)**	**32.6**	**0.04**	**38.6%**	0.268	**3.96**	**< 0.001**	0.29	**0.004**	**8**	**0.45 (0.31–0.67)**	**0.62 (0.43–0.90)**
Hyperbilirubinemia	34 (35)	**0.82 (0.70–0.95)**	**51.8**	**0.02**	**36.3%**	0.056	**2.61**	**0.01**	0.95	0.26			
Miscarriage	11 (12)	1.09 (0.61–1.96)	3.9	0.95	0.0%	—	−0.29	0.77	**0.04**	**0.03**	**4**	1.09 (0.61–1.96)	1.35 (0.78–2.33)
Stillbirth [IUFD]^a^	12 (15)	**0.36 (0.19–0.68)**	4.5	0.96	0.0%	—	**3.16**	**0.002**	0.30	0.70			
Neonatal [perinatal] death^a^	14 (24)	0.95 (0.51–1.75)	8.2	0.83	0.0%	—	0.15	0.86	1.00	0.59			
Small for gestational age	25 (27)	1.18 (0.96–1.45)	28.6	0.24	16.0%	—	−1.57	0.12	0.34	0.38			
Congenital malformation	17 (22)	0.75 (0.52–1.08)	8.5	0.93	0.0%	—	1.55	0.12	**0.01**	**0.002**	**6**	**0.84 (0.57**–**1.22)**	**0.98 (0.69**–**1.41)**
Birth injury	10 (13)	0.84 (0.56–1.25)	2.9	0.97	0.0%	—	0.87	0.38	0.59	0.28			
Hypocalcemia	6 (8)	1.14 (0.88–1.49)	8.3	0.14	39.9%	—	−1.00	0.32	0.45	0.71			
Polycythemia	4 (5)	1.16 (0.60–2.26)	0.7	0.88	0.0%	—	−0.43	0.66	0.31	0.19			
Pneumonia	6	**0.40 (0.27**–**0.59)**	4.7	0.56	0.0%	—	**4.63**	**< 0.001**	0.45	0.34			
Encephalopathy	1 (2)	0.36 (0.02–8.50)	—^e^	—^e^	—^e^	—	0.64	0.52	—^e^	—^d^			
Anemia (fetal)	2	1.02 (0.15–7.08)	0.9	0.33	0.0%	—	−0.02	0.99	1.00	—^d^			

*Note:* —, not applicable. Values in bold indicate statistical significance (*p* < 0.05).

Abbreviations: fill, number of filled studies; GH, gestational hypertension; ICU, intensive care unit; IUFD, intrauterine fetal death; LGA, large for gestational age; NICU, neonatal intensive care unit; PIH, pregnancy-induced hypertension; PROM, premature rupture of membranes; RDS, respiratory distress syndrome.

^a^If data on the former outcome did not exist, it was substituted for the latter outcome. If both the former and latter outcomes existed, data on the former outcome were chosen.

^b^Based on the Mantel–Haenszel model.

^c^RR with 95% CI was estimated based on inverse-variance methods.

^d^At least three data are required for the test.

^e^At least 2 data are required.

**Table 8:** Results of stratified analyses for the **14** main adverse pregnancy outcomes.

**Outcome**	**No.** ^**a**^	**RR (95% CI)**	**χ** ^2^	**p** ** for ** **χ** ^2^	**I** ^2^	**Tau**	**Z**	**p** ** for ** **Z**	**Metaregression**
Cesarean section									
Methods for management									0.75^e^
(a) Multifocal	20	**0.87 (0.76**–**0.999)**	**66.5**	**< 0.001**	71.4%	0.053	**1.97**	**0.049**	0.38
(b) Intensive monitoring only	21	0.94 (0.88–1.01)	25.6	0.18	22.0%	—	1.61	0.11	0.28
(c) Glucose-lowering drug^b^	9	**0.91 (0.84**–**0.99)**	95.1	0.33	12.2%	—	**2.30**	**0.02**	0.81
(d) Strict glycemic goal	3	0.91 (0.66–1.26)	2.7	0.26	25.0%	—	0.55	0.58	0.98
Drug intervention									0.73
(a) Included	13	0.96 (0.90–1.02)	18.7	0.10	35.9%	—	1.43	0.15	
(b) Not included	40	**0.91 (0.83**–**0.98)**	**82.7**	**< 0.001**	**52.9%**	0.030	**2.36**	**0.02**	
Type of diabetes									0.63
(a) Gestational diabetes	43	**0.91 (0.84**–**0.99)**	**93.5**	**< 0.001**	**55.1%**	0.041	**2.30**	**0.02**	
(b) Others^c^	9	**0.85 (0.76**–**0.94)**	8.1	0.43	0.7%	—	**3.18**	**0.001**	
Geographic region									0.12
(a) Asia and Middle East	30	**0.86 (0.78**–**0.95)**	**81.9**	**< 0.001**	**64.6%**	0.037	**3.01**	**0.003**	
(b) Others^d^	23	0.96 (0.90–1.02)	22.4	0.44	1.9%	—	1.37	0.17	

Preterm delivery									
Methods for management									0.15^e^
(a) Multifocal	22	**0.61 (0.50**–**0.74)**	32.2	0.06	34.9%	—	**4.82**	**<0.001**	**0.03**
(b) Intensive monitoring only	17	0.84 (0.69–1.01)	18.3	0.31	12.5%	—	1.90	0.06	0.28
(c) Glucose-lowering drug^b^	6	0.90 (0.80–1.02)	8.3	0.14	39.6%	—	1.67	0.10	0.15
(d) Strict glycemic goal	1	0.47 (0.05–4.65)	—	—	—	—	0.65	0.52	0.71
Drug intervention									0.45
(a) Included	9	**0.86 (0.76**–**0.97)**	13.7	0.09	41.5%	—	**2.50**	**0.02**	
(b) Not included	37	**0.73 (0.63**–**0.84)**	51.0	0.050	29.4%	—	**4.34**	**<0.001**	
Type of diabetes									0.16
(a) Gestational diabetes	37	**0.63 (0.50**–**0.81)**	**58.2**	**0.01**	**38.1%**	0.174	**3.74**	**< 0.001**	
(b) Others^c^	7	0.88 (0.76–1.004)	2.1	0.92	0.0%	—	1.90	0.06	
Geographic region									**0.01**
(a) Asia and Middle East	31	**0.58 (0.45**–**0.76)**	**50.2**	**0.01**	**40.2%**	0.189	**3.99**	**<0.001**	
(b) Others^d^	15	0.91 (0.82–1.02)	6.5	0.95	0.0%	—	1.68	0.09	

Polyhydramnios									
Methods for management									0.90^e^
(a) Multifocal	11 (12)	**0.24 (0.16**–**0.38)**	6.1	0.81	0.0%	—	**6.41**	**< 0.001**	0.91
(b) Intensive monitoring only	3 (4)	0.33 (0.09–1.21)	1.0	0.60	0.0%	—	1.67	0.09	0.67
(c) Glucose-lowering drug^b^	2	**0.23 (0.08**–**0.66)**	1.0	0.33	0.0%	—	**2.73**	**0.006**	0.81
(d) Strict glycemic goal	0	—							
Drug intervention									0.51
(a) Included	3	**0.20 (0.09**–**0.48)**	1.1	0.57	0.0%	—	**3.65**	**< 0.001**	
(b) Not included	13 (15)	**0.26 (0.17**–**0.40)**	6.7	0.88	0.0%	—	**6.15**	**< 0.001**	
Type of diabetes									—
(a) gestational diabetes	16 (18)	**0.25 (0.17**–**0.37)**	8.3	0.91	0.0%		**7.16**	**< 0.001**	
(b) Others^c^	0								
Geographic region									0.65
(a) Asia and Middle East	15 (17)	**0.25 (0.17**–**0.37)**	8.1	0.89	0.0%		**7.03**	**< 0.001**	
(b) Others^d^	1	0.14 (0.01–2.50)	—	—	—		1.33	0.18	

Pregnancy-induced hypertension									
Methods for management									0.09^e^
(a) Multifocal	15	0.79 (0.52–1.22)	**32.1**	**0.004**	**56.4%**	0.311	1.06	0.29	0.85
(b) Intensive monitoring only	13	**0.59 (0.47**–**0.75)**	12.2	0.43	1.9%	—	**4.46**	**< 0.001**	**0.01**
(c) Glucose-lowering drug^b^	7	1.09 (0.94–1.28)	4.6	0.60	0.0%	—	−1.12	0.26	0.12
(d) Strict glycemic goal	2	1.10 (0.52–2.37)	1.5	0.22	32.8%	—	−0.25	0.80	0.61
Drug intervention									0.01
(a) Included	10	1.14 (0.98–1.32)	8.4	0.49	0.0%	—	−1.68	0.09	
(b) Not included	27	**0.67 (0.52**–**0.86)**	**43.0**	**0.02**	**38.1%**	0.138	**3.09**	**0.002**	
Type of diabetes									0.81
(a) Gestational diabetes	30	**0.76 (0.59**–**0.97)**	**53.7**	**0.004**	**46.0%**	0.175	**2.21**	**0.03**	
(b) Others^c^	9	0.85 (0.67–1.07)	13.3	0.10	40.0%	—	1.36	0.18	
Geographic region									0.06
(a) Asia and Middle East	22	**0.63 (0.45**–**0.88)**	**43.8**	**0.002**	**52.1%**	0.265	**2.69**	**0.01**	
(b) Others^d^	15	1.01 (0.88–1.15)	13.3	0.13	29.9%		−0.08	0.94	

Premature rapture of membrane									
Methods for management									0.27^e^
(a) Multifocal	9 (10)	**0.51 (0.27**–**0.95)**	5.7	0.58	0.0%	—	**2.11**	**0.04**	0.51
(b) Intensive monitoring only	3	0.84 (0.61–1.14)	3.0	0.22	33.5%	—	1.13	0.26	0.16
(c) Glucose-lowering drug^b^	3	**0.42 (0.19**–**0.92)**	0.1	0.96	0.0%	—	**2.18**	**0.03**	0.33
(d) Strict glycemic goal	0								
Drug intervention									0.99
(a) Included	4	**0.68 (0.50**–**0.93)**	2.0	0.58	0.0%	—	**2.36**	**0.02**	
(b) Not included	11 (12)	**0.63 (0.52**–**0.77)**	16.3	0.09	38.8%	—	**4.50**	**< 0.001**	
Type of diabetes									—
(a) Gestational diabetes	14 (15)	**0.65 (0.55**–**0.77)**	18.3	0.15	28.9%		**5.05**	**< 0.001**	
(b) Others^c^	0	**—**							
Geographic region									0.96
(a) Asia and Middle East	12 (13)	**0.65 (0.55**–**0.77)**	15.8	0.15	30.3%	—	**5.07**	**< 0.001**	
(b) Others^d^	3	0.69 (0.24–2.01)	2.7	0.27	24.7%	—	0.68	0.50	
Postpartum hemorrhage									
Methods for management									0.39^e^
(a) Multifocal	8	**0.42 (0.27**–**0.68)**	12.1	0.10	42.2%	—	**3.86**	**< 0.001**	0.12
(b) Intensive monitoring only	4	**0.60 (0.37**–**0.97)**	2.5	0.47	0.0%	—	**2.08**	**0.04**	0.86
(c) Glucose-lowering drug^b^	3	0.83 (0.60–1.14)	1.8	0.44	0.0%	—	1.14	0.25	0.24
(d) Strict glycemic goal	0	—							
Drug intervention									0.51
(a) Included	4	0.76 (0.56–1.05)	3.9	0.27	22.8%	—	1.67	0.10	
(b) Not included	11	**0.51 (0.37**–**0.71)**	14.9	0.14	48.6%	—	**3.99**	**< 0.001**	
Type of diabetes									0.19
(a) Gestational diabetes	14	**0.61 (0.48**–**0.77)**	19.0	0.12	31.5%	—	**4.25**	**< 0.001**	
(b) Others^c^	0	—	—	—	—				
Geographic region									0.24
(a) Asia and Middle East	12	**0.48 (0.34**–**0.67)**	16.0	0.14	31.0%	—	**4.25**	**< 0.001**	
(b) Others^d^	3	0.80 (0.59–1.09)	1.9	0.39	0.0%	—	1.42	0.16	

Macrosomia									
Methods for management									0.21^e^
(a) Multifocal	20	**0.57 (0.48**–**0.68)**	24.8	0.17	23.4%	—	**6.16**	**< 0.001**	0.11
(b) Intensive monitoring only	20	0.82 (0.64–1.05)	**35.7**	**0.01**	**46.8%**	0.120	1.55	0.12	0.07
(c) Glucose-lowering drug^b^	11	**0.67 (0.58**–**0.78)**	13.3	0.21	24.7%	—	**4.92**	**< 0.001**	0.53
(d) Strict glycemic goal	4	0.70 (0.48–1.01)	6.5	0.09	53.7%	—	1.90	0.06	0.58
Drug intervention									0.72
(a) Included	16	**0.67 (0.59**–**0.77)**	20.2	0.16	25.8%	—	**5.76**	**< 0.001**	
(b) Not included	39	**0.73 (0.59**–**0.92)**	**74.0**	**< 0.001**	**48.6%**	0.158	**3.34**	**0.001**	
Type of diabetes									**0.03**
(a) Gestational diabetes	47	**0.63 (0.56**–**0.71)**	62.8	0.050	26.8%	—	**7.48**	**< 0.001**	
(b) Others^c^	10	0.93 (0.69–1.25)	**21.6**	**0.01**	**58.4%**	0.119	0.49	0.62	
Geographic region									**0.01**
(a) Asia and Middle East	31	**0.56 (0.48**–**0.65)**	39.6	0.11	24.3%	—	**7.66**	**< 0.001**	
(b) Others^d^	24	0.85 (0.70–1.02)	**42.8**	**0.01**	**46.3%**	0.080	1.74	0.08	

Neonatal hypoglycemia									
Methods for management									**0.01** ^e^
(a) Multifocal	16	**0.47 (0.36**–**0.60)**	19.4	0.20	22.5%	—	**5.85**	**< 0.001**	**< 0.001**
(b) Intensive monitoring only	18	0.86 (0.73–1.003)	12.7	0.76	0.0%	—	1.92	0.06	0.40
(c) Glucose-lowering drug^b^	10	0.85 (0.61–1.18)	**17.9**	**0.04**	**49.7%**	0.098	0.98	0.33	0.37
(d) Strict glycemic goal	2	1.32 (0.80–2.20)	1.3	0.25	24.1%	—	−1.09	0.28	0.12
Drug intervention									0.27
(a) Included	14	0.83 (0.62–1.12)	**27.0**	**0.01**	**51.9%**	0.119	1.19	0.24	
(b) Not included	32	**0.72 (0.63**–**0.81)**	41.3	0.10	25.1%	—	**4.78**	**< 0.001**	
Type of diabetes									0.73
(a) Gestational diabetes	37	**0.71 (0.56**–**0.89)**	**57.2**	**0.01**	**37.1%**	0.143	**2.97**	**0.003**	
(b) Others^c^	8	**0.68 (0.54**–**0.85)**	6.7	0.46	0.0%	—	**3.29**	**0.001**	
Geographic region									**0.002**
(a) Asia and Middle East	24	**0.50 (0.36**–**0.68)**	**38.7**	**0.02**	**40.6%**	0.188	**4.32**	**< 0.001**	
(b) Others^d^	22	0.94 (0.85–1.05)	18.3	0.63	0.0%	—	1.17	0.24	

Admission to neonatal intensive care unit									
Methods for management									**0.02** ^e^
(a) Multifocal	7	**0.55 (0.40**–**0.76)**	5.3	0.51	0.0%	—	**3.67**	**< 0.001**	**0.045**
(b) Intensive monitoring only	14	**0.71 (0.59**–**0.85)**	9.7	0.72	0.0%	—	**3.73**	**< 0.001**	0.23
(c) *Glucose-lowering drug*^b^	5	1.01 (0.87–1.16)	3.8	0.43	0.0%	—	−0.09	0.93	***0.003***
(d) Strict glycemic goal	1	0.75 (0.30–1.90)	—	—	—	—	0.61	0.54	0.92
Drug intervention									**0.004**
(a) Included	6	1.00 (0.87–1.15)	4.1	0.53	0.0%	—	0.02	0.98	
(b) Not included	21	**0.66 (0.57**–**0.78)**	16.5	0.68	0.0%		**4.42**	**< 0.001**	
Type of diabetes									0.39
(a) Gestational diabetes	22	**0.72 (0.62**–**0.85)**	23.4	0.32	10.2%	—	**3.88**	**< 0.001**	
(b) Others^c^	3	0.89 (0.70–1.15)	4.8	0.09	58.2%	—	0.88	0.38	
Geographic region									**0.004**
(a) Asia and Middle East	13	**0.61 (0.50**–**0.75)**	11.9	0.45	0.0%	—	**4.71**	**< 0.001**	
(b) Others^d^	14	0.93 (0.82–1.05)	10.1	0.68	0.0%	—	1.21	0.23	

Respiratory distress syndrome									
Methods for management									0.11^e^
(a) Multifocal	7	**0.53 (0.37**–**0.76)**	8.1	0.23	26.2%	—	**3.44**	**0.001**	**0.02**
(b) Intensive monitoring only	9	0.77 (0.50–1.17)	4.3	0.78	0.0%	—	1.22	0.22	0.96
(c) *Glucose-lowering drug*^b^	8	1.04 (0.82–1.32)	6.9	0.45	0.0%	—	−0.33	0.74	***0.02***
(d) Strict glycemic goal	2	0.63 (0.30–1.32)	3.0	0.08	67.0%	—	1.22	0.22	0.76
Drug intervention									**0.04**
(a) Included	10	1.00 (0.80–1.25)	10.3	0.33	12.5%	—	0.03	0.98	
(b) Not included	16	**0.62 (0.47**–**0.82)**	14.5	0.49	0.0%	—	**3.41**	**0.001**	
Type of diabetes									0.89
(a) Gestational diabetes	21	**0.77 (0.62**–**0.96)**	25.7	0.18	22.2%	—	**2.28**	**0.02**	
(b) Others^c^	5	0.81 (0.48–1.38)	5.2	0.27	23.00%	—	0.78	0.44	
Geographic region									**0.01**
(a) Asia and Middle East	13	**0.55 (0.41**–**0.73)**	14.4	0.28	16.5%	—	**4.07**	**< 0.001**	
(b) Others^d^	13	1.05 (0.84–1.30)	6.7	0.88	0.0%	—	−0.41	0.68	

Fetal distress									
Methods for management									0.09^e^
(a) Multifocal	14	**0.34 (0.23**–**0.49)**	16.1	0.25	19.1%	—	**5.74**	**< 0.001**	0.12
(b) Intensive monitoring only	3	**0.36 (0.20**–**0.97)**	1.3	0.54	0.0%	—	**3.23**	**0.001**	0.67
(c) Glucose-lowering drug^b^	3	1.19 (0.53–2.66)	1.7	0.43	0.0%	—	−0.42	0.67	0.11
(d) Strict glycemic goal	1 (2)	1.02 (0.58–1.78)	—	—	—	—	−0.05	0.96	0.16
Drug intervention									0.10
(a) Included	6 (7)	0.56 (0.21–1.49)	**11.9**	**0.04**	**58.0%**	0.685	1.16	0.25	
(b) Not included	15	**0.38 (0.28**–**0.53)**	13.5	0.49	0.0%	—	**5.77**	**< 0.001**	
Type of diabetes									**0.03**
(a) Gestational diabetes	20 (21)	**0.38 (0.28**–**0.52)**	23.9	0.20	20.4%	—	**6.29**	**< 0.001**	
(b) Others^c^	2	1.02 (0.61–1.70)	0.0	0.98	0.0%	—	−0.07	0.94	
Geographic region									0.14
(a) Asia and Middle East	18	**0.39 (0.29**–**0.53)**	25.3	0.09	32.8%	—	**6.16**	**< 0.001**	
(b) Others^d^	3 (4)	0.92 (0.55–1.54)	0.5	0.77	0.0%	—	0.30	0.76	

Hyperbilirubinemia									
Methods for management									0.34^e^
(a) Multifocal	12	**0.70 (0.59**–**0.84)**	13.2	0.28	16.8%	—	**4.02**	**< 0.001**	0.11
(b) Intensive monitoring only	10	0.99 (0.80–1.23)	9.5	0.39	5.2%	—	0.05	0.96	0.34
(c) Glucose-lowering drug^b^	10 (11)	0.92 (0.66–1.29)	**20.1**	**0.02**	**55.1%**	0.113	0.49	0.62	0.31
(d) Strict glycemic goal	2	0.69 (0.42–1.16)	0.2	0.64	0.0%	—	1.39	0.64	0.58
Drug intervention									0.64
(a) Included	13 (14)	0.85 (0.65–1.11)	**22.7**	**0.03**	**47.1%**	0.086	1.21	0.23	
(b) Not included	21	**0.80 (0.70**–**0.92)**	28.7	0.09	30.3%		**3.12**	**0.002**	
Type of diabetes									0.31
(a) Gestational diabetes	29 (30)	**0.77 (0.64**–**0.93)**	**44.7**	**0.02**	**37.4%**	0.080	**2.75**	**0.006**	
(b) Others^c^	5	1.03 (0.82–1.29)	6.3	0.18	36.5%	—	−0.26	0.80	
Geographic region									0.15
(a) Asia and Middle East	20	**0.74 (0.61**–**0.91)**	**33.8**	**0.02**	**43.8%**	0.077	**2.81**	**0.01**	
(b) Others^d^	14 (15)	0.98 (0.83–1.15)	14.3	0.36	8.9%	—	0.26	0.80	

Small for gestational age									
Methods for management									0.12^e^
(a) Multifocal	5 (6)	0.92 (0.55–1.54)	1.7	0.79	0.0%	—	0.32	0.75	0.41
(b) Intensive monitoring only	12 (13)	0.96 (0.65–1.41)	11.9	0.37	7.2%	—	0.21	0.84	0.36
(c) Glucose-lowering drug^b^	6	1.28 (0.96–1.70)	8.0	0.16	37.6%	—	0.24	0.81	0.45
(d) *Strict glycemic goal*	2	***5.40 (1.44***–***20.23)***	0.04	0.84	0.0%	—	***−2.50***	***0.01***	***0.03***
Drug intervention									0.16
(a) Included	9	***1.36 (1.05***–***1.75)***	12.7	0.12	37.1%	—	***−2.32***	***0.02***	
(b) Not included	16 (18)	0.92 (0.65–1.29)	13.5	0.57	0.0%	—	0.48	0.63	
Type of diabetes									0.16
(a) Gestational diabetes	19 (21)	1.09 (0.84–1.41)	22.8	0.20	21.2%	—	−0.65	0.52	
(b) Others^c^	5	***1.84 (1.11***–***3.04)***	2.1	0.72	0.0%	—	***−2.35***	***0.02***	
Geographic region									0.24
(a) Asia and Middle East	11 (12)	0.96 (0.70–1.33)	9.3	0.51	0.0%	—	0.24	0.81	
(b) Others^d^	14 (15)	***1.35 (1.03***–***1.76)***	17.5	0.18	25.9%	—	***−2.21***	***0.03***	

Congenital malformation									
Methods for management									0.61^e^
(a) Multifocal	8 (11)	**0.51 (0.27**–**0.95)**	5.7	0.58	0.0%	—	**2.11**	**0.04**	0.43
(b) Intensive monitoring only	5 (6)	0.78 (0.38–1.56)	1.0	0.79	0.0%	—	0.69	0.49	0.84
(c) Glucose-lowering drug^b^	4	1.04 (0.58–1.87)	1.1	0.79	0.0%	—	−0.13	0.89	0.36
(d) Strict glycemic goal	0 (1)	**—**							
Drug intervention									0.36
(a) Included	4 (6)	1.04 (0.58–1.87)	1.1	0.79	0.0%	—	−0.13	0.89	
(b) Not included	13 (16)	**0.61 (0.38**–**0.98)**	6.6	0.88	0.0%	—	**2.04**	**0.04**	
Type of diabetes									0.67
(a) Gestational diabetes	13 (18)	0.66 (0.42–1.02)	8.0	0.78	0.0%	—	1.86	0.06	
(b) Others^c^	4	0.92 (0.44–1.94)	0.2	0.98	0.0%	—	0.21	0.83	
Geographic region									0.22
(a) Asia and Middle East	10 (13)	**0.56 (0.34**–**0.92)**	5.7	0.77	0.0%	—	**2.23**	**0.02**	
(b) Others^d^	7 (9)	1.07 (0.62–1.84)	1.3	0.97	0.0%	—	−0.25	0.80	

*Note:* —, not applicable. If data on both the diabetes type and combination of diabetes types existed, we chose data for the latter in the sensitivity analysis according to the focus and drug intervention as in the overall analysis (Table 7) and chose data for the former in the sensitivity analysis according to the diabetes types. Values in bold indicate statistical significance (*p* < 0.05).

Abbreviations: CI, confidence interval; RR, relative risk.

^a^Values in parentheses indicate including data without events in both intervention and control groups.

^b^Means starting or adding glucose-lowering drugs (does not mean change of drug dose).

^c^Including Type 1, Type 2, Type 1 or Type 2, and pregestational diabetes, data with nonspecified diabetes (i.e., GDM and the other types of diabetes were excluded.

^d^Multicenter study was included.

^e^Multivariate metaregression was used. In the other analysis, univariate metaregression was used.

**Table 9:** Estimated relative risk (95% confidence interval) of adverse pregnancy outcomes for the increments of each glycemic control (GC) indicator.

**Indicator outcome**	**A1C**				**FPG**				**2hPG**				**MBG**			
	**No.**	**r**	**Per −1%**	**p**	**No.**	**r**	**Per −10** mg**/dL**	**p**	**No.**	**r**	**Per −10 mg/dL**	**P**	**No.**	**r**	**Per −10 mg/dL**	**p**
Cesarean section	35	**0.59**	**0.66 (0.53**–**0.83)**	**0.001**	31	0.38	0.86 (0.72–1.01)	0.07	23	0.32	0.94 (0.87–1.03)	0.17	15	0.43	0.95 (0.89–1.01)	0.11

Preterm delivery	30	**0.49**	**0.52 (0.32**–**0.84)**	**0.01**	29	**0.60**	**0.76 (0.64**–**0.91)**	**0.004**	24	**0.73**	**0.76 (0.66**–**0.86)**	**< 0.001**	13	0.57	0.79 (0.60–1.03)	0.07

Polyhydramnios	11	0.20	1.00 (0.26–3.94)	0.99	14	0.63	0.61 (0.32–1.16)	0.12	14	0.59	0.77 (0.53–1.12)	0.15	1	—		

PIH	25	**0.51**	**0.42 (0.23**–**0.77)**	**0.01**	21	**0.62**	**0.64 (0.48**–**0.85)**	**0.004**	15	**0.72**	**0.74 (0.61**–**0.90)**	**0.01**	12	0.43	0.91 (0.76–1.09)	0.28

PROM	11	0.34	0.67 (0.29–1.54)	0.30	13	0.35	0.80 (0.49–1.30)	0.33	10	−0.39	1.20 (0.73–1.95)	0.42	3	0.39	0.82 (< 0.001–2.27 × 10^3^)	0.80

Postpartum hemorrhage	7	0.37	0.65 (0.15–2.90)	0.49	**12**	**0.62**	**0.74 (0.57**–**0.97)**	**0.03**	10	0.48	0.73 (0.45–1.18)	0.17	1	—		

Macrosomia	37	**0.50**	**0.38 (0.22**–**0.65)**	**0.001**	35	**0.44**	**0.77 (0.63**–**0.95)**	**0.02**	28	0.74	0.89 (0.77–1.02)	0.09	16	**0.65**	**0.71 (0.55**–**0.91)**	**0.01**

Hypoglycemia^a^	32	**0.62**	**0.38 (0.23**–**0.63)**	**< 0.001**	28	**0.78**	**0.51 (0.39**–**0.67)**	**< 0.001**	21	**0.62**	**0.75 (0.61**–**0.93)**	**0.01**	15	0.48	0.71 (0.50–1.01)	0.06

Admission to NICU	17	0.26	0.70 (0.31–1.57)	0.36	16	**0.66**	**0.54 (0.34**–**0.87)**	**0.02**	11	0.66	0.76 (0.57–1.01)	0.054	10	0.59	0.79 (0.58–1.07)	0.11

RDS	16	0.19	0.66 (0.16–2.79)	0.55	16	**0.68**	**0.56 (0.38**–**0.85)**	**0.01**	11	0.54	0.75 (0.52–1.08)	0.11	13	**0.69**	**0.72 (0.55**–**0.94)**	**0.02**

Fetal distress	13	0.48	0.45 (0.14–1.46)	0.16	17	**0.72**	**0.49 (0.29**–**0.84)**	**0.01**	13	0.42	0.81 (0.50–1.29)	0.33	3	1.00	0.56 (0.05–6.63)	0.21

Hyperbilirubinemia	21	**0.47**	**0.57 (0.35**–**0.93)**	**0.03**	24	**0.64**	**0.74** (0.62–0.88)	**0.002**	18	**0.84**	**0.69 (0.60**–**0.80)**	**< 0.001**	12	0.20	0.77 (0.21–2.79)	0.67

SGA	21	0.09	0.76 (0.18–3.29)	0.70	9	0.39	0.57 (0.15–2.10)	0.34	7	0.76	0.53 (0.28–1.00)	0.050	10	0.35	0.79 (0.46–1.36)	0.34

Congenital malformation	12	0.31	0.75 (0.21–2.69)	0.63	13	0.70	0.66 (0.40–1.07)	0.09	11	−0.25	1.01 (0.75–1.37)	0.93	5	−0.15	1.15 (0.002–8.13 × 10^2^)	0.95

*Note:* —, impossible to analyze because of insufficient number of data. Values in bold indicate statistical significance (*p* < 0.05).

Abbreviations: 2hPG, 2-h postprandial glucose; A1C, hemoglobin A1c; FPG, fasting plasma glucose; MBG, mean blood glucose; NICU, neonatal intensive care unit; PIH, pregnancy-induced hypertension; PROM, premature rupture of membrane; RDS, respiratory distress syndrome; SGA, small for gestational age.

^a^Neonatal hypoglycemia.

**Table 10:** Estimated relative risk (95% confidence interval) of adverse pregnancy outcomes for increments of each glycemic control (GC) indicator limiting analyses to trials for gestational diabetes.

**Outcome**	**GC indicator**															
	**A1C**				**FPG**				**2hPG**				**MBG**			
	**No.**	**r**	**Per −1%**	**p**	**No.**	**r**	**Per −10** mg**/dL**	**p**	**No.**	**r**	**Per −10 mg/dL**	**p**	**No.**	**r**	**Per −10 mg/dL**	**p**
Cesarean section	26	**0.67**	**0.63 (0.49**–**0.80)**	**0.001**	30	0.41	0.85 (0.71–1.01)	0.07	22	0.36	0.94 (0.85–1.03)	0.17	9	0.59	0.94 (0.86–1.02)	0.11

Preterm delivery	22	**0.47**	**0.54 (0.31**–**0.95)**	**0.03**	28	**0.52**	**0.77 (0.64**–**0.94)**	**0.01**	22	**0.50**	**0.78 (0.64**–**0.95)**	**0.02**	7	0.66	0.72 (0.47–1.11)	0.11

Polyhydramnios	11	−0.20	1.00 (0.26–3.94)	0.99	14	0.63	0.61 (0.32–1.16)	0.12	14	0.59	0.77 (0.53–1.12)	0.15	—			

PIH	19	**0.65**	**0.39 (0.21**–**0.74)**	**0.01**	19	**0.59**	**0.66 (0.48**–**0.89)**	**0.01**	14	**0.60**	**0.79 (0.64**–**0.98)**	**0.03**	8	0.59	0.91 (0.72–1.14)	0.35

PROM	10	0.34	0.66 (0.28–1.57)	0.31	13	0.35	0.80 (0.49–1.30)	0.33	10	−0.39	1.20 (0.73–1.95)	0.42	3	0.39	0.82 (< 0.001–2.27 × 10^3^)	0.80

Postpartum hemorrhage	6	0.43	0.66 (0.11–3.81)	0.55	**12**	**0.62**	**0.74 (0.57**–**0.97)**	**0.03**	10	0.48	0.73 (0.45–1.18)	0.17	1	—		

Macrosomia	30	**0.49**	**0.40 (0.22**–**0.72)**	**0.003**	34	**0.44**	**0.77 (0.63**–**0.95)**	**0.02**	27	0.32	0.89 (0.77–1.04)	0.15	10	0.50	0.80 (0.57–1.13)	0.17

Hypoglycemia^a^	23	**0.65**	**0.40 (0.21**–**0.73)**	**0.005**	27	**0.79**	**0.50 (0.38**–**0.66)**	**< 0.001**	20	**0.59**	**0.75 (0.59**–**0.941)**	**0.02**	10	0.57	0.67 (0.43–1.04)	0.07

Admission to NICU	12	0.42	0.63 (0.26–1.55)	0.28	15	**0.61**	**0.57 (0.35**–**0.95)**	**0.03**	10	0.51	0.79 (0.55–1.13)	0.16	8	0.71	0.81 (0.60–1.09)	0.14

RDS	12	0.19	0.73 (0.14–3.90)	0.68	15	**0.67**	**0.58 (0.38**–**0.88)**	**0.01**	10	0.49	0.78 (0.53–1.16)	0.19	9	0.61	0.71 (0.47–1.06)	0.08

Fetal distress	12	0.30	0.56 (0.15–2.03)	0.34	16	**0.70**	**0.57 (0.34**–**0.96)**	**0.04**	13	0.43	0.81 (0.50–1.29)	0.33	3	0.97	0.54 (0.04–7.78)	0.21

Hyperbilirubinemia	16	0.45	0.63 (0.38–1.05)	0.07	23	**0.61**	**0.76 (0.63**–**0.91)**	**0.005**	17	**0.80**	**0.70 (0.59**–**0.84)**	**0.001**	9	0.23	0.77 (0.15–3.93)	0.71

SGA	15	0.21	0.55 (0.10–3.16)	0.47	8	0.32	0.58 (0.12–2.82)	0.43	6	**0.82**	**0.41 (0.17**–**0.98)**	**0.047**	7	0.30	0.84 (0.45–1.59)	0.52

Congenital malformation	8	−0.07	1.09 (0.15–8.05)	0.92	12	0.69	0.96 (0.91–1.01)	0.10	10	−0.36	1.09 (0.76–1.56)	0.59	3	0.82	0.35 (< 0.001–1.38 × 10^18^)	0.81

*Note:* —, impossible to analyze because of insufficient number of data. Values in bold indicate statistical significance (*p* < 0.05).

Abbreviations: 2hPG, 2-h postprandial glucose; A1C, hemoglobin A1c; FPG, fasting plasma glucose; MBG, mean blood glucose; NICU, neonatal intensive care unit; PIH, pregnancy-induced hypertension; PROM, premature rapture of membrane; RDS, respiratory distress syndrome; SGA, small for gestational age.

^a^Neonatal hypoglycemia.

**Table 11:** Estimated relative risk (95% confidence interval) of adverse pregnancy outcomes for the increment of each glycemic control (GC) indicator stratifying geographic regions into Asian and non-Asian areas.

**Outcome**		**Indicator of GC**											
		**A1C**				**FPG**				**2hPG**			
		**No.**	**r**	**Per −1%**	**p**	**No.**	**r**	**Per −10 mg/dL**	**p**	**No.**	**r**	**Per −10 mg/dL**	**p**
Cesarean section	Asian and Middle East	20	**0.69**	**0.65 (0.49**–**0.85)**	**0.003**	21	0.42	0.89 (0.71–1.11)	0.28	17	0.34	0.97 (0.87–1.08)	0.52
	Others	15	−0.06	1.03 (0.50–2.14)	0.93	10	0.24	0.78 (0.37–1.64)	0.47	6	0.22	0.84 (0.34–2.07)	0.62

Preterm delivery	Asian and Middle East	18	0.48	0.54 (0.29–1.01)	0.06	24	**0.49**	**0.81 (0.66**–**0.99)**	**0.04**	21	0.41	0.81 (0.64–1.02)	0.07
	Others	12	0.13	0.87 (0.15–5.08)	0.86	5	−0.32	1.56 (0.06–43.90)	0.70	3	−0.16	1.00 (< 0.001–1.46 × 10^22^)	1.00

PIH	Asian and Middle East	14	**0.70**	**0.38 (0.18**–**0.78)**	**0.01**	15	0.51	0.71 (0.48–1.04)	0.07	12	0.37	0.87 (0.66–1.15)	0.28
	Others	11	***−0.67***	***14.76 (1.36***–***1.61 × 10***^***2***^**)**	***0.03***	6	−0.22	1.28 (0.17–9.57)	0.75	3	−0.77	37.2 (< 0.001–3.25 × 10^22^)	0.52

Macrosomia	Asian and Middle East	21	**0.48**	**0.47 (0.24**–**0.90)**	**0.03**	24	0.49	0.80 (0.64–1.00)	0.051	21	0.40	0.91 (0.76–1.08)	0.26
	Others	16	0.51	0.31 (0.08–1.20)	0.09	11	0.06	0.93 (0.20–4.26)	0.91	7	−0.62	2.11 (0.71–6.33)	0.14

Neonatal hypoglycemia	Asian and Middle East	17	**0.71**	**0.38** (0.19–0.78)	**0.01**	19	**0.83**	**0.51 (0.38**–**0.69)**	**< 0.001**	16	0.50	0.79 (0.59–1.06)	0.10
	Others	15	0.13	0.77 (0.21–2.79)	0.67	9	−0.47	2.67 (0.29–24.19)	0.33	5	−0.22	1.16 (0.23–5.85)	0.79

Hyperbilirubinemia	Asian and Middle East	12	0.51	0.66 (0.41–1.08)	0.09	17	**0.68**	**0.73 (0.60**–**0.89)**	**0.004**	15	**0.87**	**0.67 (0.56**–**0.80)**	**< 0.001**
	Others	9	0.06	0.97 (0.10–9.31)	0.97	7	0.28	0.87 (0.02–31.16)	0.92	3	0.90	0.42 (1.49 × 10^2^)	0.31

*Note:* Values in bold indicate statistical significance (*p* < 0.05).

Abbreviations: 2hPG, 2-h postprandial glucose; A1C, hemoglobin A1c; FPG, fasting plasma glucose; PIH, pregnancy-induced hypertension.

## Data Availability

The data that support the findings of this study are available in the supporting information of this article.

## Supporting Information

Supporting Information 1 Forest plots of relative risks (RRs) with 95% confidence interval (CI) from individual trials. Size of squares is proportional to study weight (i.e., inverse of variance of logarithm of RR). The RR was not calculated for data with no event in both intervention and control groups, as indicated by “Excluded.”

Supporting Information 2 Funnel plots of the logarithm of relative risk (RR) and its corresponding standard error (se) for each adverse pregnancy outcome. Visually, asymmetry of the funnel plot suggests bias which was statistically detected (see Table 7).

Supporting Information 3 Scatterplots of reductions in each glycemic indicator and relative risks (RRs) for 14 main adverse pregnancy outcomes. Solid regression lines correspond to all scatterplots and dashed lines correspond to scatterplots from trials of patients with gestational diabetes. The size of circles is proportional to study weight (i.e., inverse of variance of logarithm of RR). Gray circles show trials for gestational diabetes, and white circles show trials for other type of diabetes. The bold regression line indicates that the association was statistically significant, and the thin regression line indicates that the association was not significant. The upper regression formula was determined by analyzing all trials; the lower regression formula was determined limiting the metaregression to trials for gestational diabetes. Panels showing only one regression line and formula indicate that all trials targeted only patients with gestational diabetes. Abbreviations: 2hPG, 2-h postprandial glucose; A1C, hemoglobin A1c; FPG, fasting plasma glucose; MBG, mean blood glucose.

Supporting Information 4 Scatterplots of reductions in each glycemic indicator and relative risks (RRs) for six adverse pregnancy outcomes. Solid regression lines correspond to scatterplots for trials conducted in Asia or the Middle East, and dashed regression lines correspond to scatterplots for trials conducted in countries other than in Asia and the Middle East. Size of circles is proportional to study weight (i.e., inverse of variance of logarithm of RR). White circles indicate trials performed in Asia or the Middle East, and gray circles indicate trials performed in countries other than in Asia and the Middle East. Upper and lower regression formulas are determined when trials conducted for Asian and non-Asian countries, respectively, were analyzed. Panels that show only one regression line and formula are those for which regression lines could not be drawn for trials conducted in non-Asian countries because of an insufficient number of data (at least three data are necessary). Abbreviations: 2hPG, 2-h postprandial glucose; A1C, hemoglobin A1c; FPG, fasting plasma glucose; MBG, mean blood glucose.
