# Supplementary material for: Relationship Between Improvements in Glycemic Control and Risk of Pregnancy Complications in Patients With Diabetes Mellitus: Metaregression Analysis of Randomized Controlled Trials of Intensive Glucose Management
Source: J Diabetes Res. 2025 Jun 23;2025:3490884. doi: 10.1155/jdr/3490884 (PMC12208766; doi:10.1155/jdr/3490884)
Supplement: Supporting Information 1 — Forest plots of relative risks (RRs) with 95% confidence interval (CI) from individual trials. Size of squares is proportional to study weight (i.e., inverse of variance of logarithm of RR). The RR was not calculated for data with no event in both intervention and control groups, as indicated by “Excluded.” [file 3490884.f1.pdf]

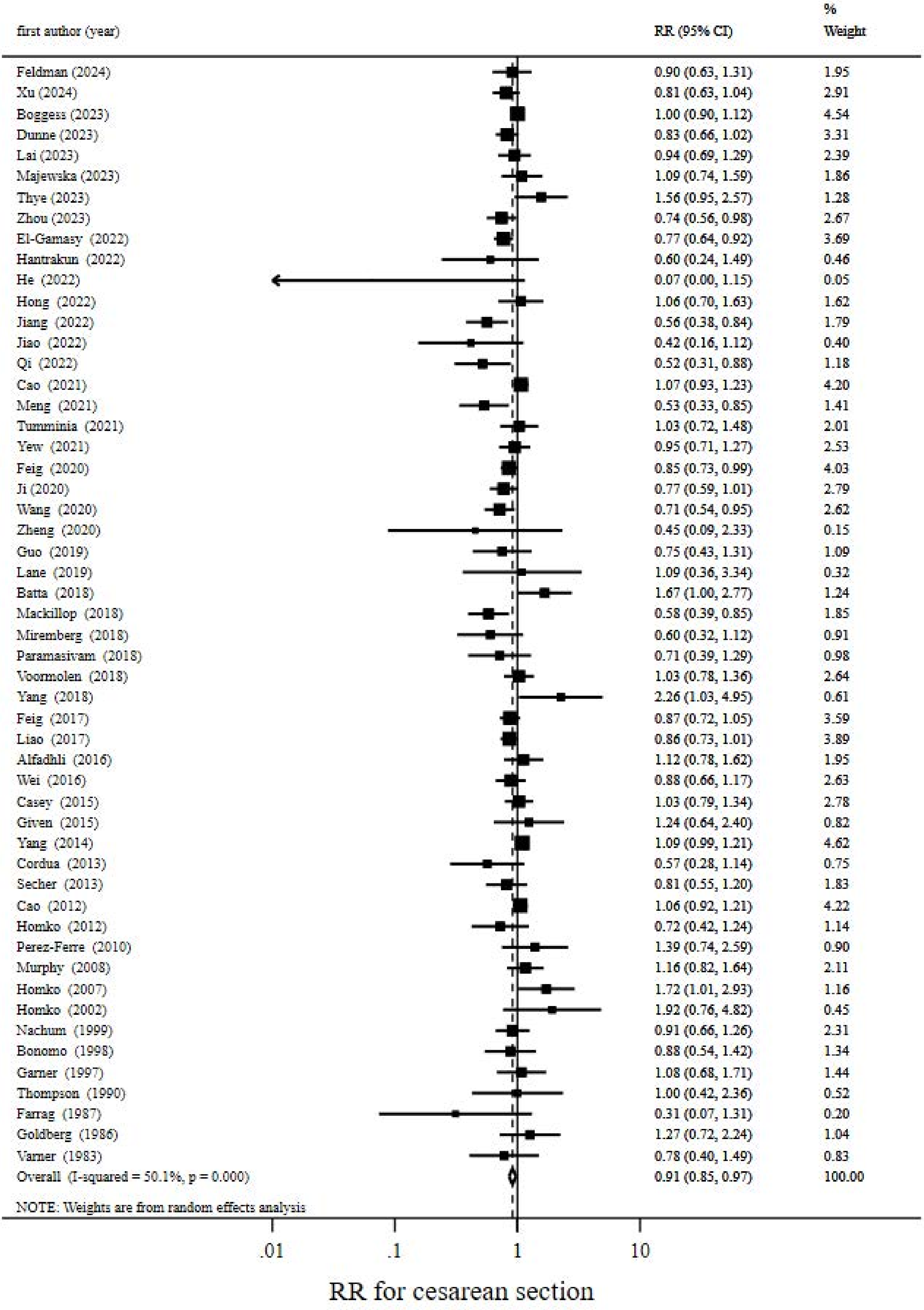
first author (year)
RR (95% CI)
% Weight
Feldman (2024) 0.90 (0.63, 1.31) 1.95
Xu (2024) 0.81 (0.63, 1.04) 2.91
Boggess (2023) 1.00 (0.90, 1.12) 4.54
Dunne (2023) 0.83 (0.66, 1.02) 3.31
Lai (2023) 0.94 (0.69, 1.29) 2.39
Majewska (2023) 1.09 (0.74, 1.59) 1.86
Thye (2023) 1.56 (0.95, 2.57) 1.28
Zhou (2023) 0.74 (0.56, 0.98) 2.67
El-Gamasy (2022) 0.77 (0.64, 0.92) 3.69
Hantrakun (2022) 0.60 (0.24, 1.49) 0.46
He (2022) 0.07 (0.00, 1.15) 0.05
Hong (2022) 1.06 (0.70, 1.63) 1.62
Jiang (2022) 0.56 (0.38, 0.84) 1.79
Jiao (2022) 0.42 (0.16, 1.12) 0.40
Qi (2022) 0.52 (0.31, 0.88) 1.18
Cao (2021) 1.07 (0.93, 1.23) 4.20
Meng (2021) 0.53 (0.33, 0.85) 1.41
Tumminia (2021) 1.03 (0.72, 1.48) 2.01
Yew (2021) 0.95 (0.71, 1.27) 2.53
Feig (2020) 0.85 (0.73, 0.99) 4.03
Ji (2020) 0.77 (0.59, 1.01) 2.79
Wang (2020) 0.71 (0.54, 0.95) 2.62
Zheng (2020) 0.45 (0.09, 2.33) 0.15
Guo (2019) 0.75 (0.43, 1.31) 1.09
Lane (2019) 1.09 (0.36, 3.34) 0.32
Batta (2018) 1.67 (1.00, 2.77) 1.24
Mackillop (2018) 0.58 (0.39, 0.85) 1.85
Miremberg (2018) 0.60 (0.32, 1.12) 0.91
Paramasivam (2018) 0.71 (0.39, 1.29) 0.98
Voormolen (2018) 1.03 (0.78, 1.36) 2.64
Yang (2018) 2.26 (1.03, 4.95) 0.61
Feig (2017) 0.87 (0.72, 1.05) 3.59
Liao (2017) 0.86 (0.73, 1.01) 3.89
Alfadhli (2016) 1.12 (0.78, 1.62) 1.95
Wei (2016) 0.88 (0.66, 1.17) 2.63
Casey (2015) 1.03 (0.79, 1.34) 2.78
Given (2015) 1.24 (0.64, 2.40) 0.82
Yang (2014) 1.09 (0.99, 1.21) 4.62
Cordua (2013) 0.57 (0.28, 1.14) 0.75
Secher (2013) 0.81 (0.55, 1.20) 1.83
Cao (2012) 1.06 (0.92, 1.21) 4.22
Homko (2012) 0.72 (0.42, 1.24) 1.14
Perez-Ferre (2010) 1.39 (0.74, 2.59) 0.90
Murphy (2008) 1.16 (0.82, 1.64) 2.11
Homko (2007) 1.72 (1.01, 2.93) 1.16
Homko (2002) 1.92 (0.76, 4.82) 0.45
Nachum (1999) 0.91 (0.66, 1.26) 2.31
Bonomo (1998) 0.88 (0.54, 1.42) 1.34
Garner (1997) 1.08 (0.68, 1.71) 1.44
Thompson (1990) 1.00 (0.42, 2.36) 0.52
Farrag (1987) 0.31 (0.07, 1.31) 0.20
Goldberg (1986) 1.27 (0.72, 2.24) 1.04
Varner (1983) 0.78 (0.40, 1.49) 0.83
Overall (I-squared = 50.1%, p = 0.000) 0.91 (0.85, 0.97) 100.00
NOTE: Weights are from random effects analysis
.01
.1
1
10
RR for cesarean section

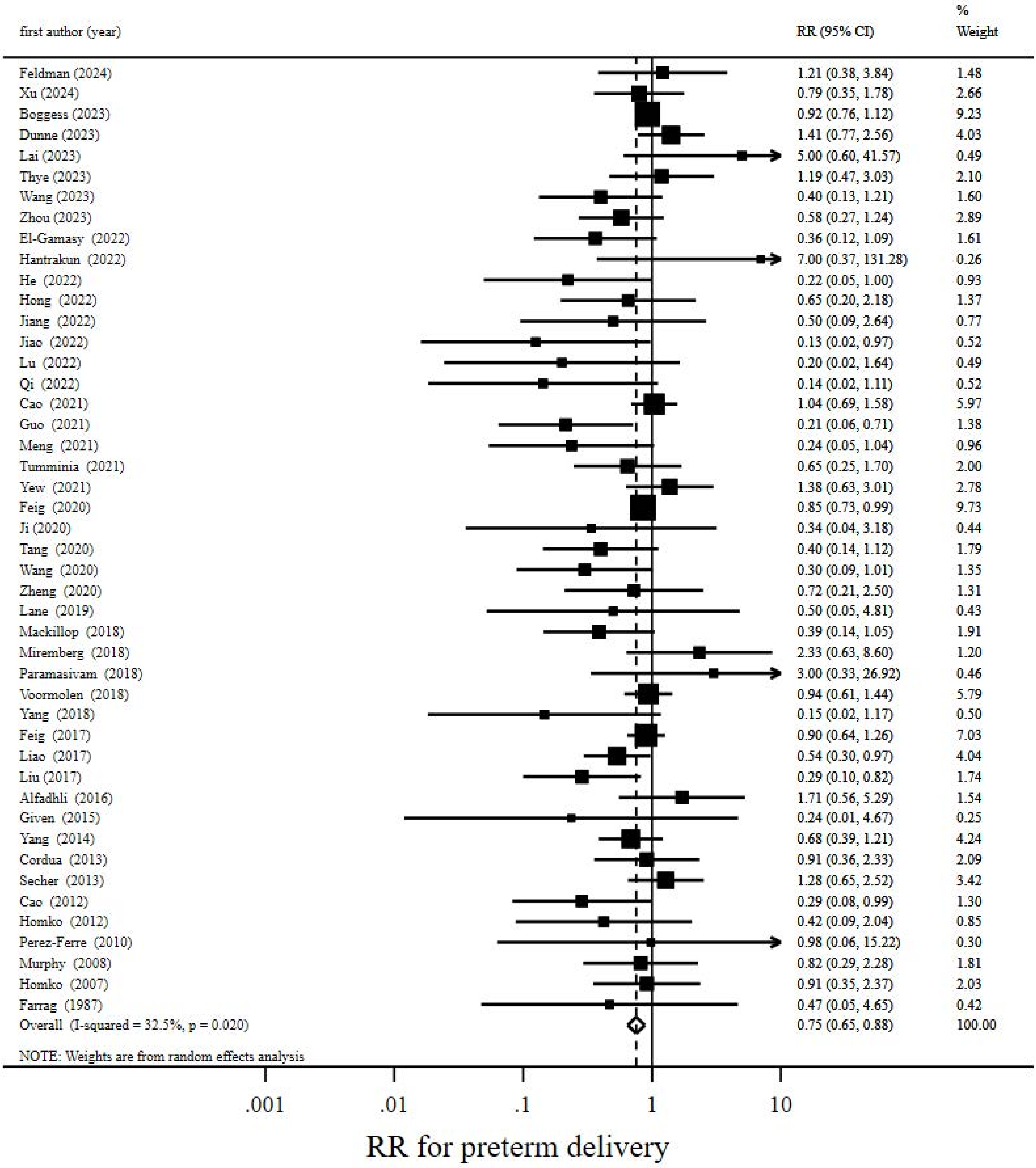
first author (year)
RR (95% CI)
% Weight
Feldman (2024) 1.21 (0.38, 3.84) 1.48
Xu (2024) 0.79 (0.35, 1.78) 2.66
Boggess (2023) 0.92 (0.76, 1.12) 9.23
Dunne (2023) 1.41 (0.77, 2.56) 4.03
Lai (2023) 5.00 (0.60, 41.57) 0.49
Thye (2023) 1.19 (0.47, 3.03) 2.10
Wang (2023) 0.40 (0.13, 1.21) 1.60
Zhou (2023) 0.58 (0.27, 1.24) 2.89
El-Gamasy (2022) 0.36 (0.12, 1.09) 1.61
Hantrakun (2022) 7.00 (0.37, 131.28) 0.26
He (2022) 0.22 (0.05, 1.00) 0.93
Hong (2022) 0.65 (0.20, 2.18) 1.37
Jiang (2022) 0.50 (0.09, 2.64) 0.77
Jiao (2022) 0.13 (0.02, 0.97) 0.52
Lu (2022) 0.20 (0.02, 1.64) 0.49
Qi (2022) 0.14 (0.02, 1.11) 0.52
Cao (2021) 1.04 (0.69, 1.58) 5.97
Guo (2021) 0.21 (0.06, 0.71) 1.38
Meng (2021) 0.24 (0.05, 1.04) 0.96
Tumminia (2021) 0.65 (0.25, 1.70) 2.00
Yew (2021) 1.38 (0.63, 3.01) 2.78
Feig (2020) 0.85 (0.73, 0.99) 9.73
Ji (2020) 0.34 (0.04, 3.18) 0.44
Tang (2020) 0.40 (0.14, 1.12) 1.79
Wang (2020) 0.30 (0.09, 1.01) 1.35
Zheng (2020) 0.72 (0.21, 2.50) 1.31
Lane (2019) 0.50 (0.05, 4.81) 0.43
Mackillop (2018) 0.39 (0.14, 1.05) 1.91
Miremberg (2018) 2.33 (0.63, 8.60) 1.20
Paramasivam (2018) 3.00 (0.33, 26.92) 0.46
Voormolen (2018) 0.94 (0.61, 1.44) 5.79
Yang (2018) 0.15 (0.02, 1.17) 0.50
Feig (2017) 0.90 (0.64, 1.26) 7.03
Liao (2017) 0.54 (0.30, 0.97) 4.04
Liu (2017) 0.29 (0.10, 0.82) 1.74
Alfadhli (2016) 1.71 (0.56, 5.29) 1.54
Given (2015) 0.24 (0.01, 4.67) 0.25
Yang (2014) 0.68 (0.39, 1.21) 4.24
Cordua (2013) 0.91 (0.36, 2.33) 2.09
Secher (2013) 1.28 (0.65, 2.52) 3.42
Cao (2012) 0.29 (0.08, 0.99) 1.30
Homko (2012) 0.42 (0.09, 2.04) 0.85
Perez-Ferre (2010) 0.98 (0.06, 15.22) 0.30
Murphy (2008) 0.82 (0.29, 2.28) 1.81
Homko (2007) 0.91 (0.35, 2.37) 2.03
Farrag (1987) 0.47 (0.05, 4.65) 0.42
Overall (I-squared = 32.5%, p = 0.020) 0.75 (0.65, 0.88) 100.00
NOTE: Weights are from random effects analysis
.001
.01
.1
1
10
RR for preterm delivery

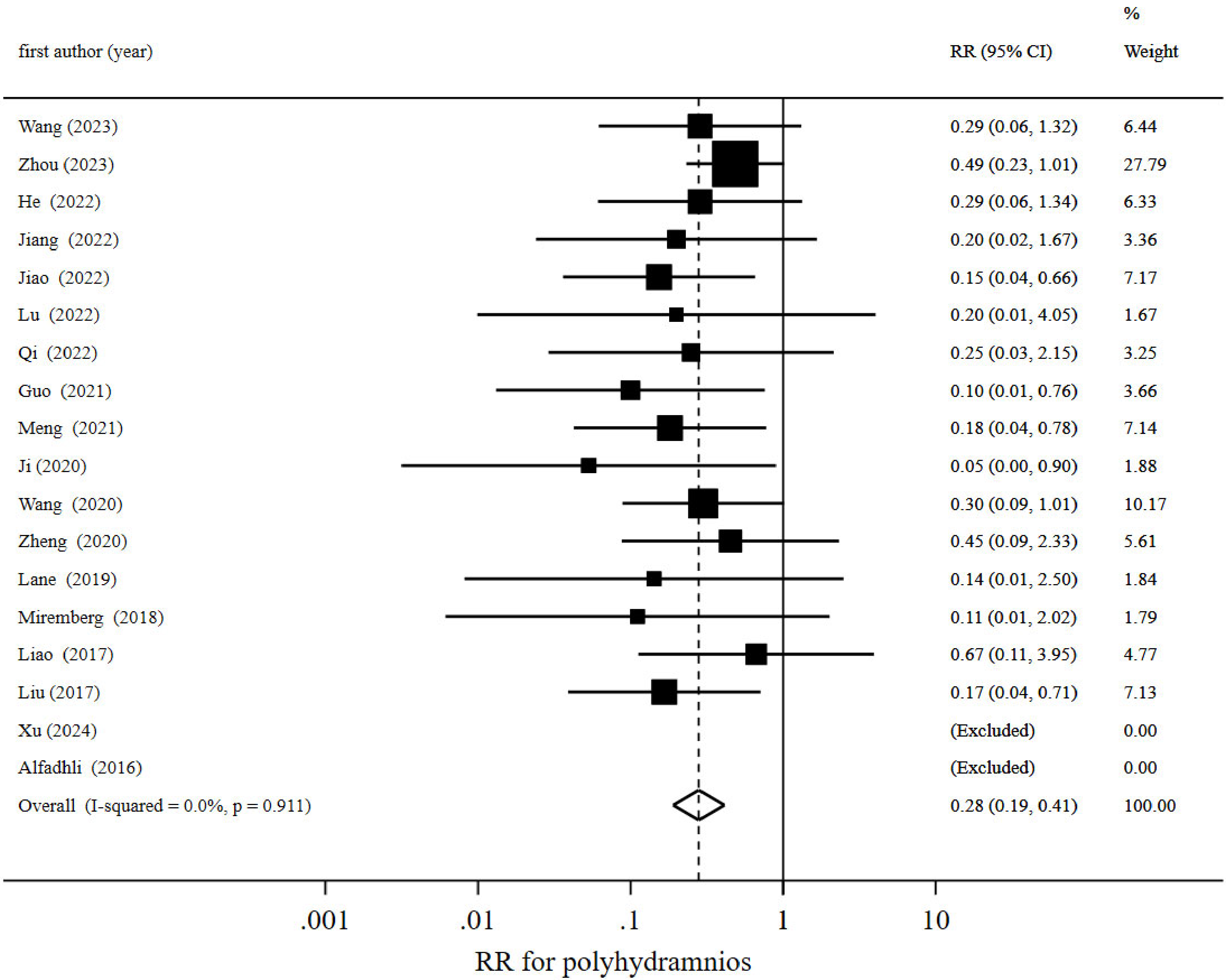

%
Weight
first author (year)
RR (95% CI)
Wang (2023)
0.29 (0.06, 1.32)
6.44
Zhou (2023)
0.49 (0.23, 1.01)
27.79
He (2022)
0.29 (0.06, 1.34)
6.33
Jiang (2022)
0.20 (0.02, 1.67)
3.36
Jiao (2022)
0.15 (0.04, 0.66)
7.17
Lu (2022)
0.20 (0.01, 4.05)
1.67
Qi (2022)
0.25 (0.03, 2.15)
3.25
Guo (2021)
0.10 (0.01, 0.76)
3.66
Meng (2021)
0.18 (0.04, 0.78)
7.14
Ji (2020)
0.05 (0.00, 0.90)
1.88
Wang (2020)
0.30 (0.09, 1.01)
10.17
Zheng (2020)
0.45 (0.09, 2.33)
5.61
Lane (2019)
0.14 (0.01, 2.50)
1.84
Miremberg (2018)
0.11 (0.01, 2.02)
1.79
Liao (2017)
0.67 (0.11, 3.95)
4.77
Liu (2017)
0.17 (0.04, 0.71)
7.13
Xu (2024)
(Excluded)
0.00
Alfadhli (2016)
(Excluded)
0.00
Overall (I-squared = 0.0%, p = 0.911)
0.28 (0.19, 0.41)
100.00
.001
.01
.1
1
10
RR for polyhydramnios

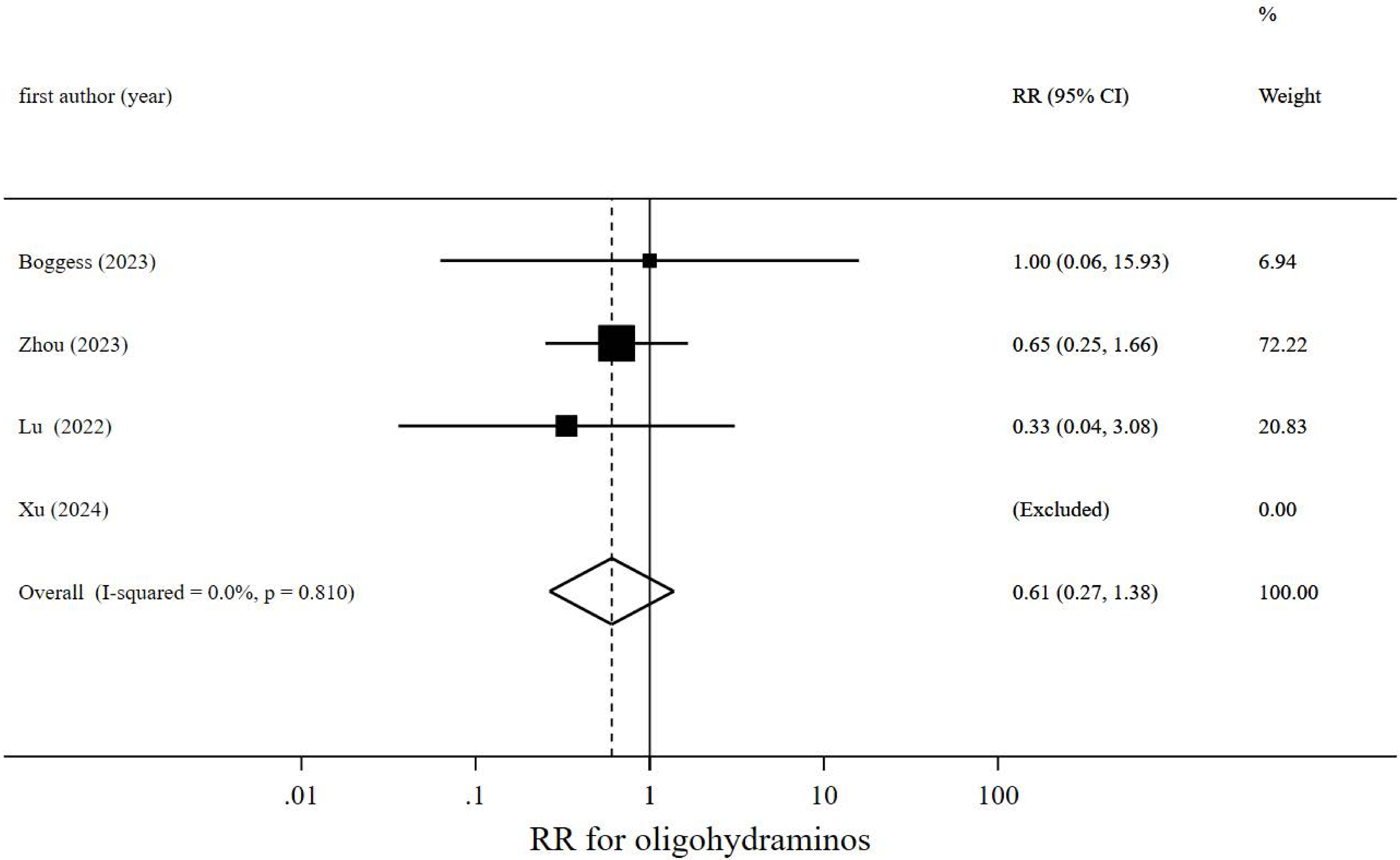

%
first author (year)
RR (95% CI)
Weight
Boggess (2023)
1.00 (0.06, 15.93)
6.94
Zhou (2023)
0.65 (0.25, 1.66)
72.22
Lu (2022)
0.33 (0.04, 3.08)
20.83
Xu (2024)
(Excluded)
0.00
Overall (I-squared = 0.0%, p = 0.810)
0.61 (0.27, 1.38)
100.00
.01
.1
1
10
100
RR for oligohydraminos

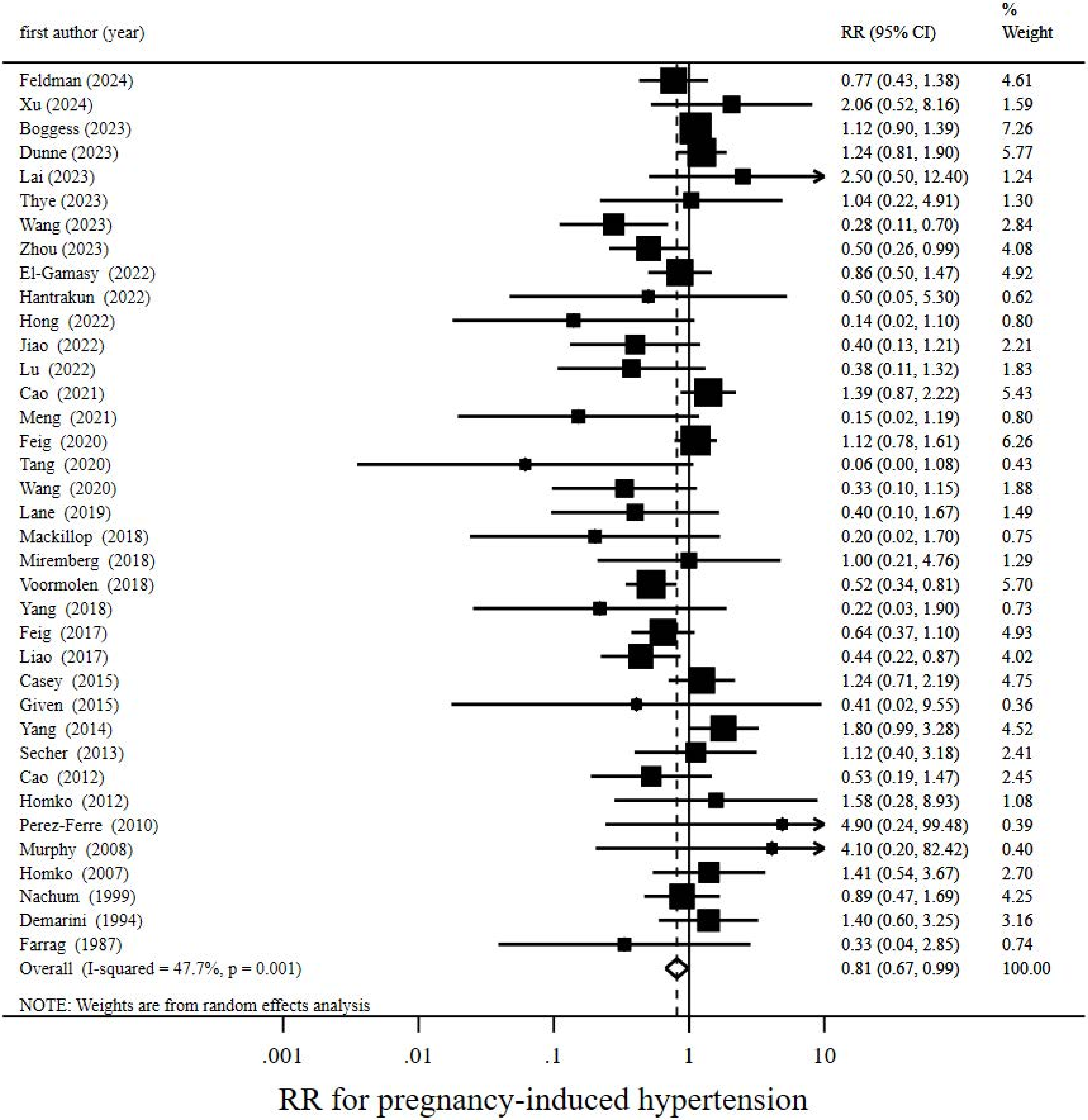
first author (year)
RR (95% CI)
% Weight
Feldman (2024) 0.77 (0.43, 1.38) 4.61
Xu (2024) 2.06 (0.52, 8.16) 1.59
Boggess (2023) 1.12 (0.90, 1.39) 7.26
Dunne (2023) 1.24 (0.81, 1.90) 5.77
Lai (2023) 2.50 (0.50, 12.40) 1.24
Thye (2023) 1.04 (0.22, 4.91) 1.30
Wang (2023) 0.28 (0.11, 0.70) 2.84
Zhou (2023) 0.50 (0.26, 0.99) 4.08
El-Gamasy (2022) 0.86 (0.50, 1.47) 4.92
Hantrakun (2022) 0.50 (0.05, 5.30) 0.62
Hong (2022) 0.14 (0.02, 1.10) 0.80
Jiao (2022) 0.40 (0.13, 1.21) 2.21
Lu (2022) 0.38 (0.11, 1.32) 1.83
Cao (2021) 1.39 (0.87, 2.22) 5.43
Meng (2021) 0.15 (0.02, 1.19) 0.80
Feig (2020) 1.12 (0.78, 1.61) 6.26
Tang (2020) 0.06 (0.00, 1.08) 0.43
Wang (2020) 0.33 (0.10, 1.15) 1.88
Lane (2019) 0.40 (0.10, 1.67) 1.49
Mackillop (2018) 0.20 (0.02, 1.70) 0.75
Miremberg (2018) 1.00 (0.21, 4.76) 1.29
Voormolen (2018) 0.52 (0.34, 0.81) 5.70
Yang (2018) 0.22 (0.03, 1.90) 0.73
Feig (2017) 0.64 (0.37, 1.10) 4.93
Liao (2017) 0.44 (0.22, 0.87) 4.02
Casey (2015) 1.24 (0.71, 2.19) 4.75
Given (2015) 0.41 (0.02, 9.55) 0.36
Yang (2014) 1.80 (0.99, 3.28) 4.52
Secher (2013) 1.12 (0.40, 3.18) 2.41
Cao (2012) 0.53 (0.19, 1.47) 2.45
Homko (2012) 1.58 (0.28, 8.93) 1.08
Perez-Ferre (2010) 4.90 (0.24, 99.48) 0.39
Murphy (2008) 4.10 (0.20, 82.42) 0.40
Homko (2007) 1.41 (0.54, 3.67) 2.70
Nachum (1999) 0.89 (0.47, 1.69) 4.25
Demarini (1994) 1.40 (0.60, 3.25) 3.16
Farrag (1987) 0.33 (0.04, 2.85) 0.74
Overall (I-squared = 47.7%, p = 0.001) 0.81 (0.67, 0.99) 100.00
NOTE: Weights are from random effects analysis
.001
.01
.1
1
10
RR for pregnancy-induced hypertension

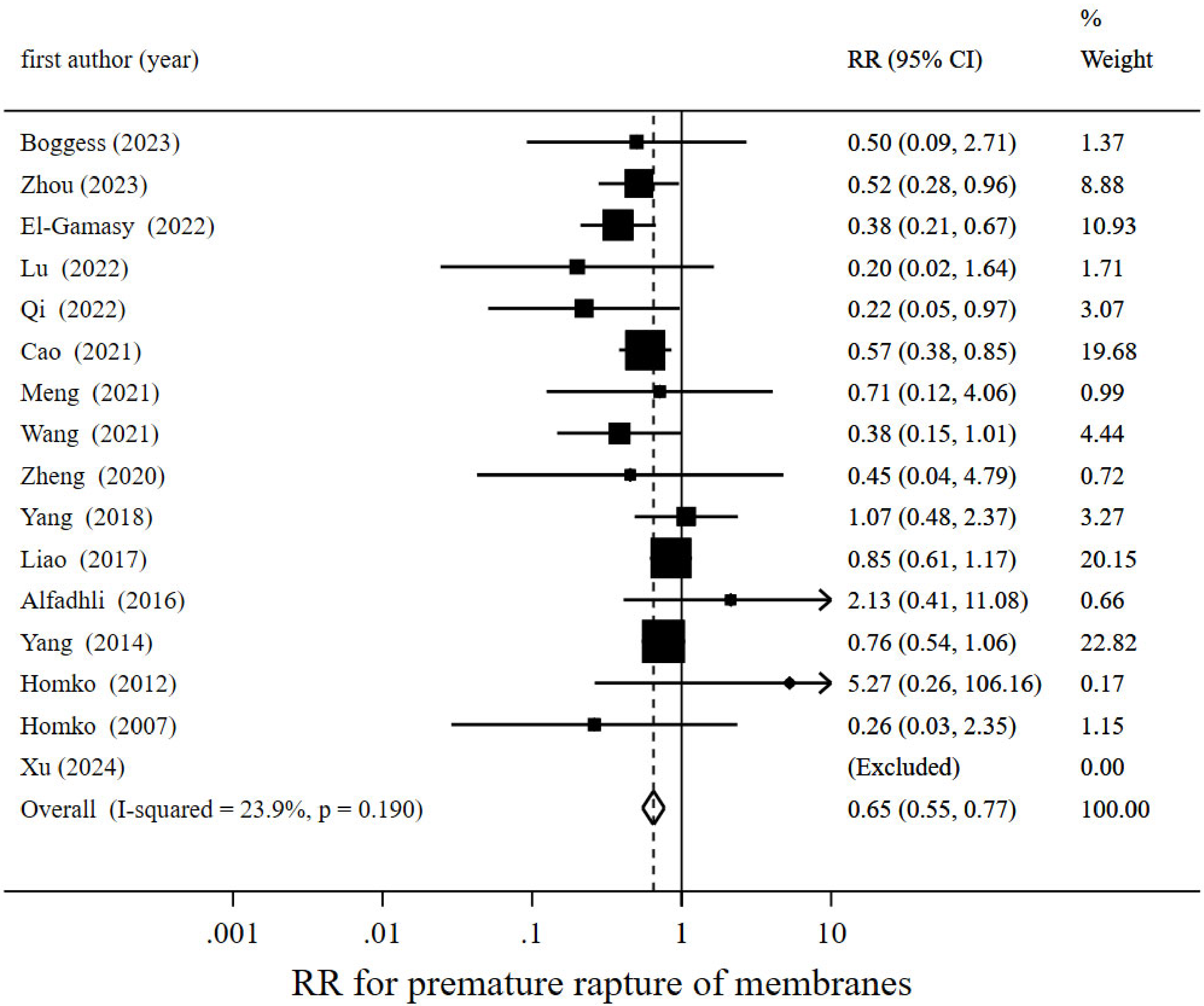
first author (year)
RR (95% CI)
% Weight
Boggess (2023) 0.50 (0.09, 2.71) 1.37
Zhou (2023) 0.52 (0.28, 0.96) 8.88
El-Gamasy (2022) 0.38 (0.21, 0.67) 10.93
Lu (2022) 0.20 (0.02, 1.64) 1.71
Qi (2022) 0.22 (0.05, 0.97) 3.07
Cao (2021) 0.57 (0.38, 0.85) 19.68
Meng (2021) 0.71 (0.12, 4.06) 0.99
Wang (2021) 0.38 (0.15, 1.01) 4.44
Zheng (2020) 0.45 (0.04, 4.79) 0.72
Yang (2018) 1.07 (0.48, 2.37) 3.27
Liao (2017) 0.85 (0.61, 1.17) 20.15
Alfadhli (2016) 2.13 (0.41, 11.08) 0.66
Yang (2014) 0.76 (0.54, 1.06) 22.82
Homko (2012) 5.27 (0.26, 106.16) 0.17
Homko (2007) 0.26 (0.03, 2.35) 1.15
Xu (2024) (Excluded) 0.00
Overall (I-squared = 23.9%, p = 0.190) 0.65 (0.55, 0.77) 100.00
.001
.01
.1
1
10
RR for premature rapture of membranes

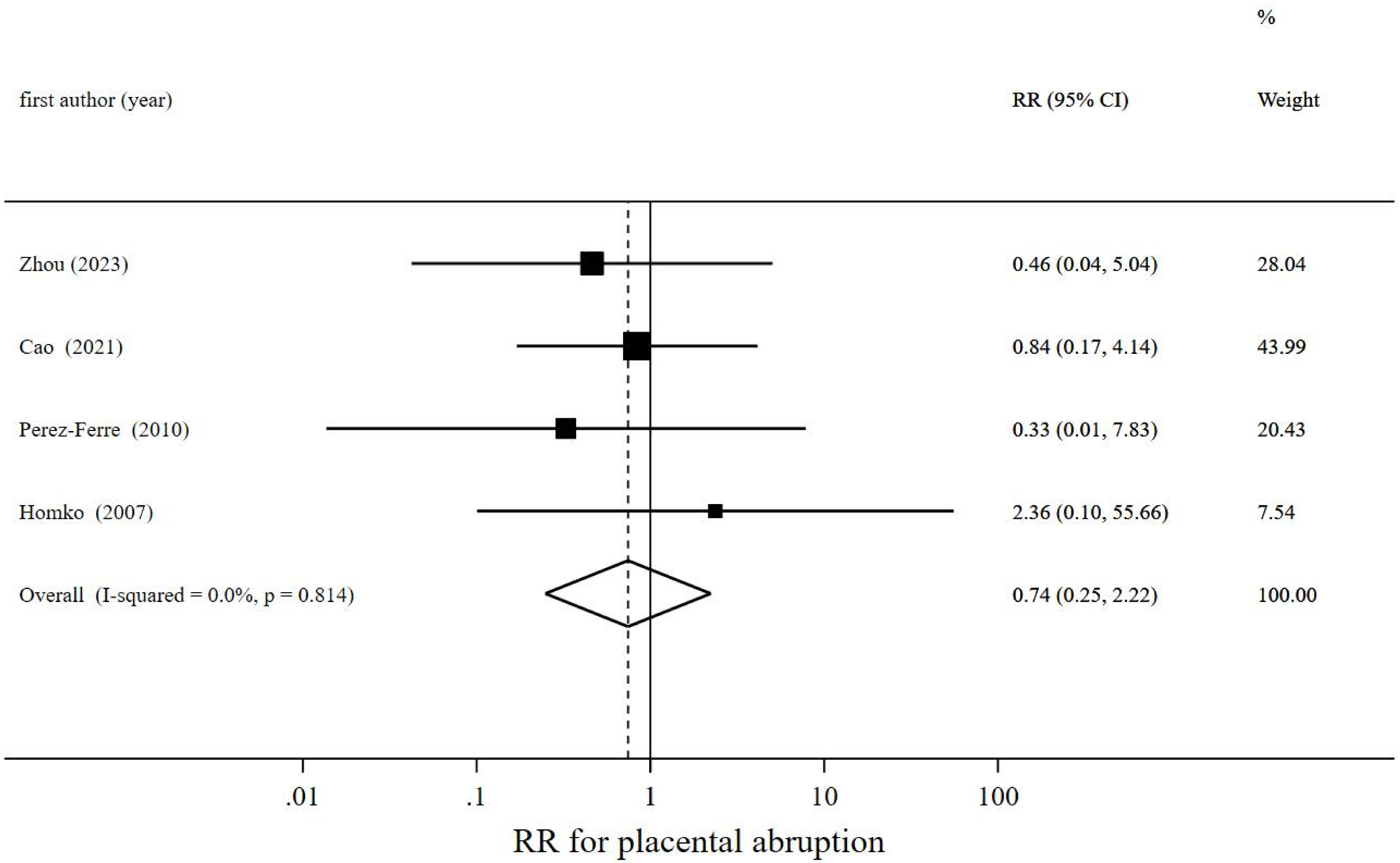
%
first author (year)
RR (95% CI)
Weight
Zhou (2023)
0.46 (0.04, 5.04)
28.04
Cao (2021)
0.84 (0.17, 4.14)
43.99
Perez-Ferre (2010)
0.33 (0.01, 7.83)
20.43
Homko (2007)
2.36 (0.10, 55.66)
7.54
Overall (I-squared = 0.0%, p = 0.814)
0.74 (0.25, 2.22)
100.00
.01
.1
1
10
100
RR for placental abruption

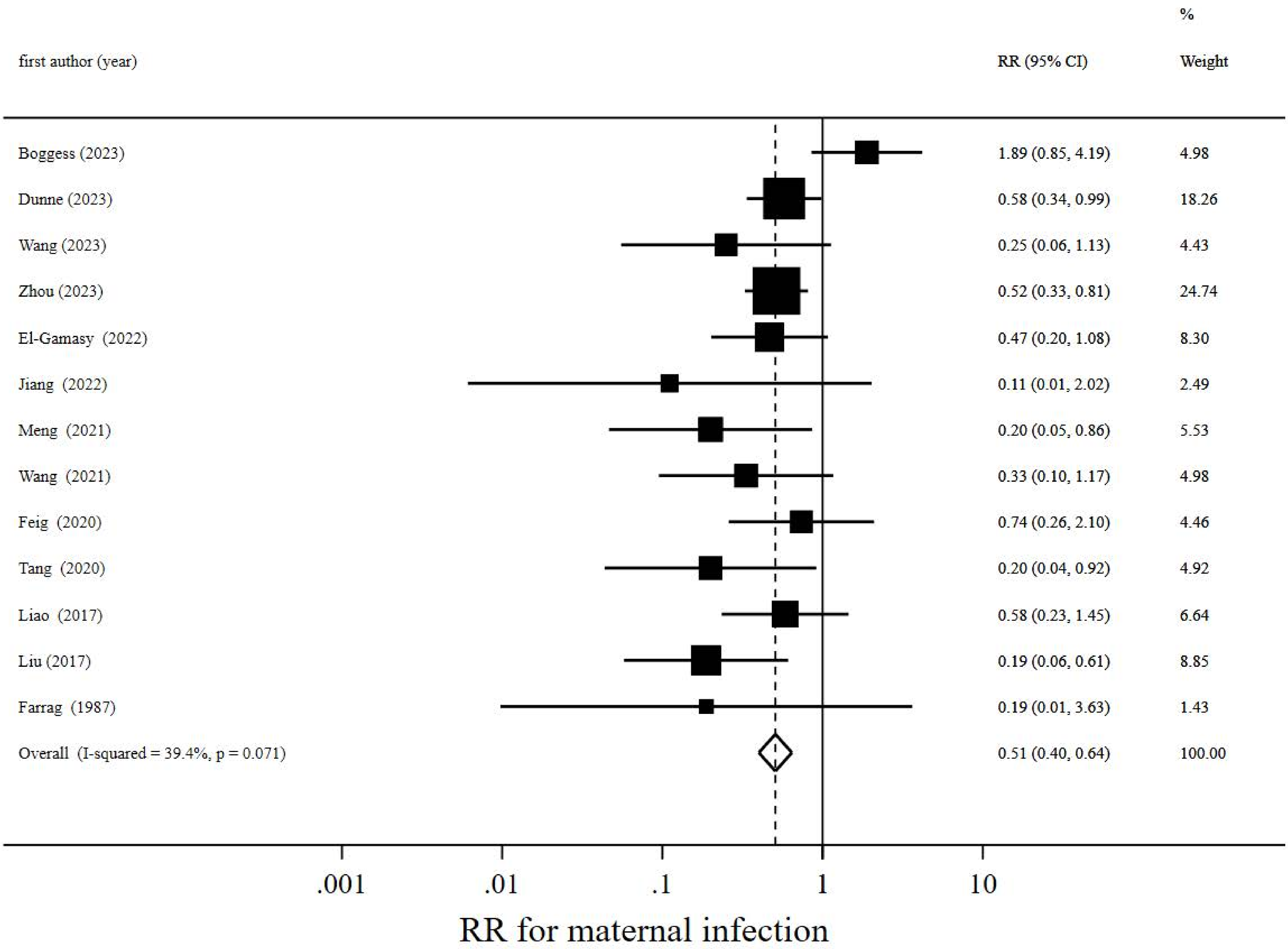

first author (year)
RR (95% CI)
%
Weight
Boggess (2023)
1.89 (0.85, 4.19)
4.98
Dunne (2023)
0.58 (0.34, 0.99)
18.26
Wang (2023)
0.25 (0.06, 1.13)
4.43
Zhou (2023)
0.52 (0.33, 0.81)
24.74
El-Gamasy (2022)
0.47 (0.20, 1.08)
8.30
Jiang (2022)
0.11 (0.01, 2.02)
2.49
Meng (2021)
0.20 (0.05, 0.86)
5.53
Wang (2021)
0.33 (0.10, 1.17)
4.98
Feig (2020)
0.74 (0.26, 2.10)
4.46
Tang (2020)
0.20 (0.04, 0.92)
4.92
Liao (2017)
0.58 (0.23, 1.45)
6.64
Liu (2017)
0.19 (0.06, 0.61)
8.85
Farrag (1987)
0.19 (0.01, 3.63)
1.43
Overall (I-squared = 39.4%, p = 0.071)
0.51 (0.40, 0.64)
100.00
.001
.01
.1
1
10
RR for maternal infection

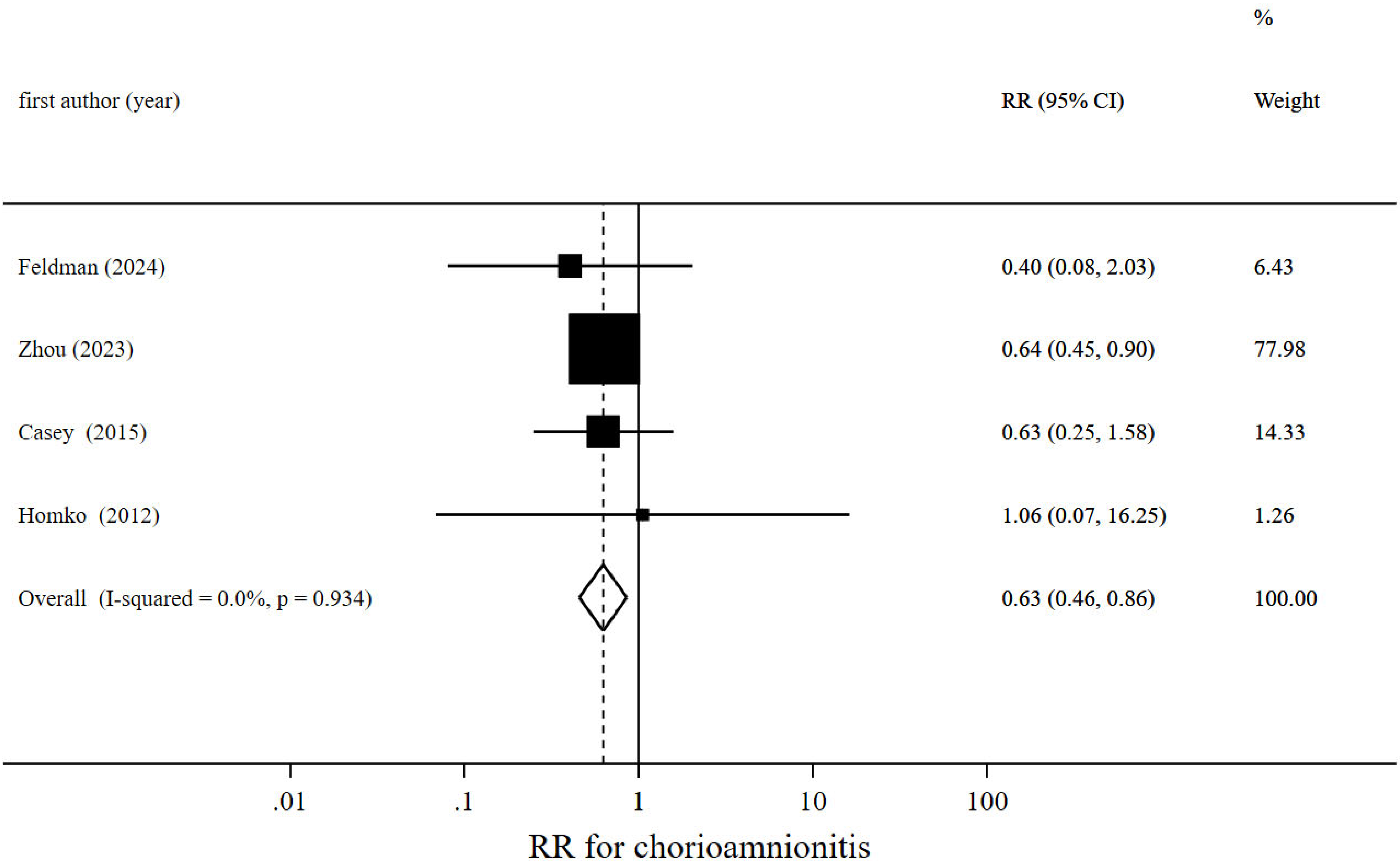

%
first author (year)
RR (95% CI)
Weight
Feldman (2024)
0.40 (0.08, 2.03)
6.43
Zhou (2023)
0.64 (0.45, 0.90)
77.98
Casey (2015)
0.63 (0.25, 1.58)
14.33
Homko (2012)
1.06 (0.07, 16.25)
1.26
Overall (I-squared = 0.0%, p = 0.934)
0.63 (0.46, 0.86)
100.00
.01
.1
1
10
100
RR for chorioamnionitis

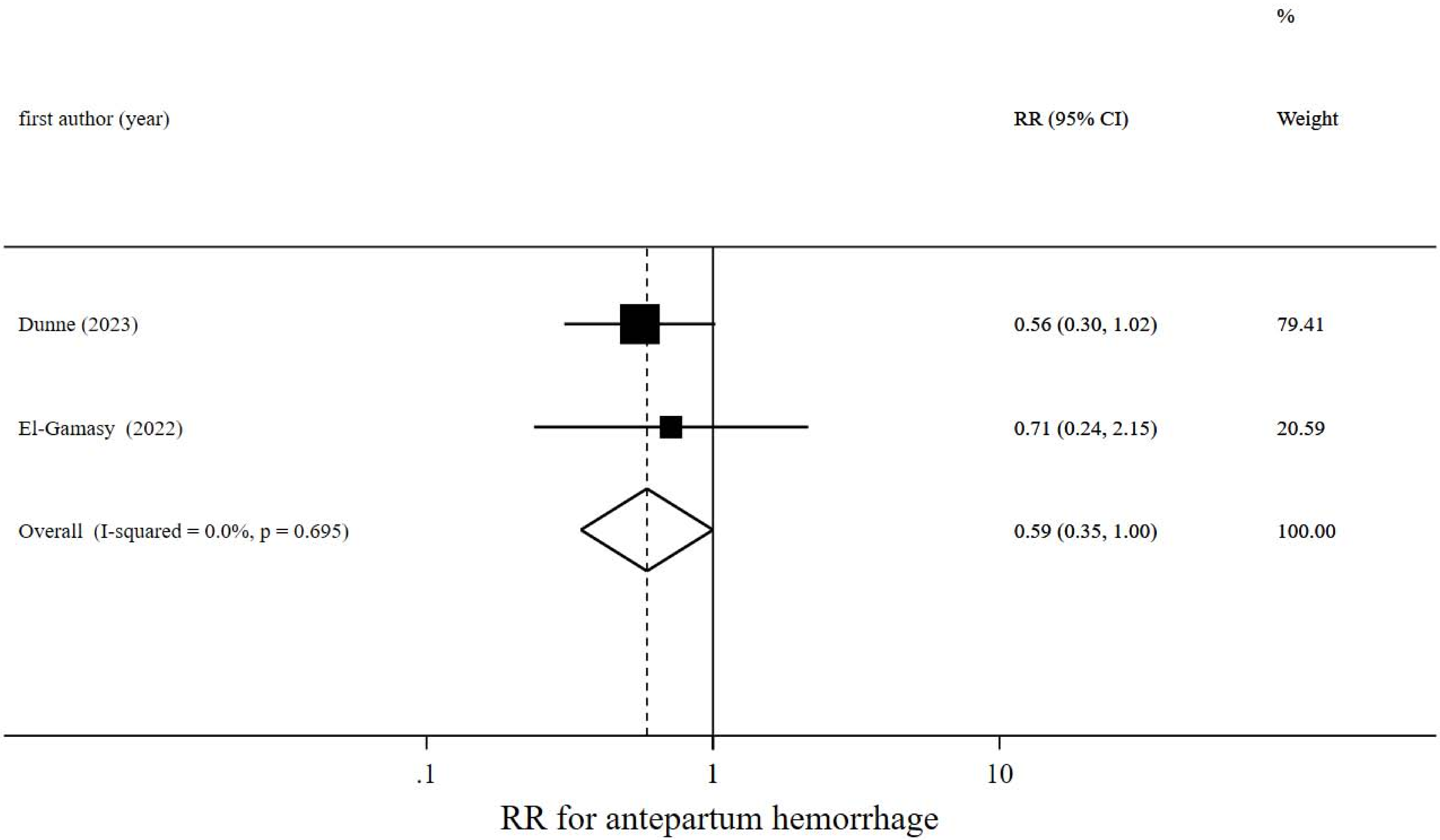
%
first author (year)
RR (95% CI)
Weight
Dunne (2023)
0.56 (0.30, 1.02)
79.41
El-Gamasy (2022)
0.71 (0.24, 2.15)
20.59
Overall (I-squared = 0.0%, p = 0.695)
0.59 (0.35, 1.00)
100.00
.1
1
10
RR for antepartum hemorrhage

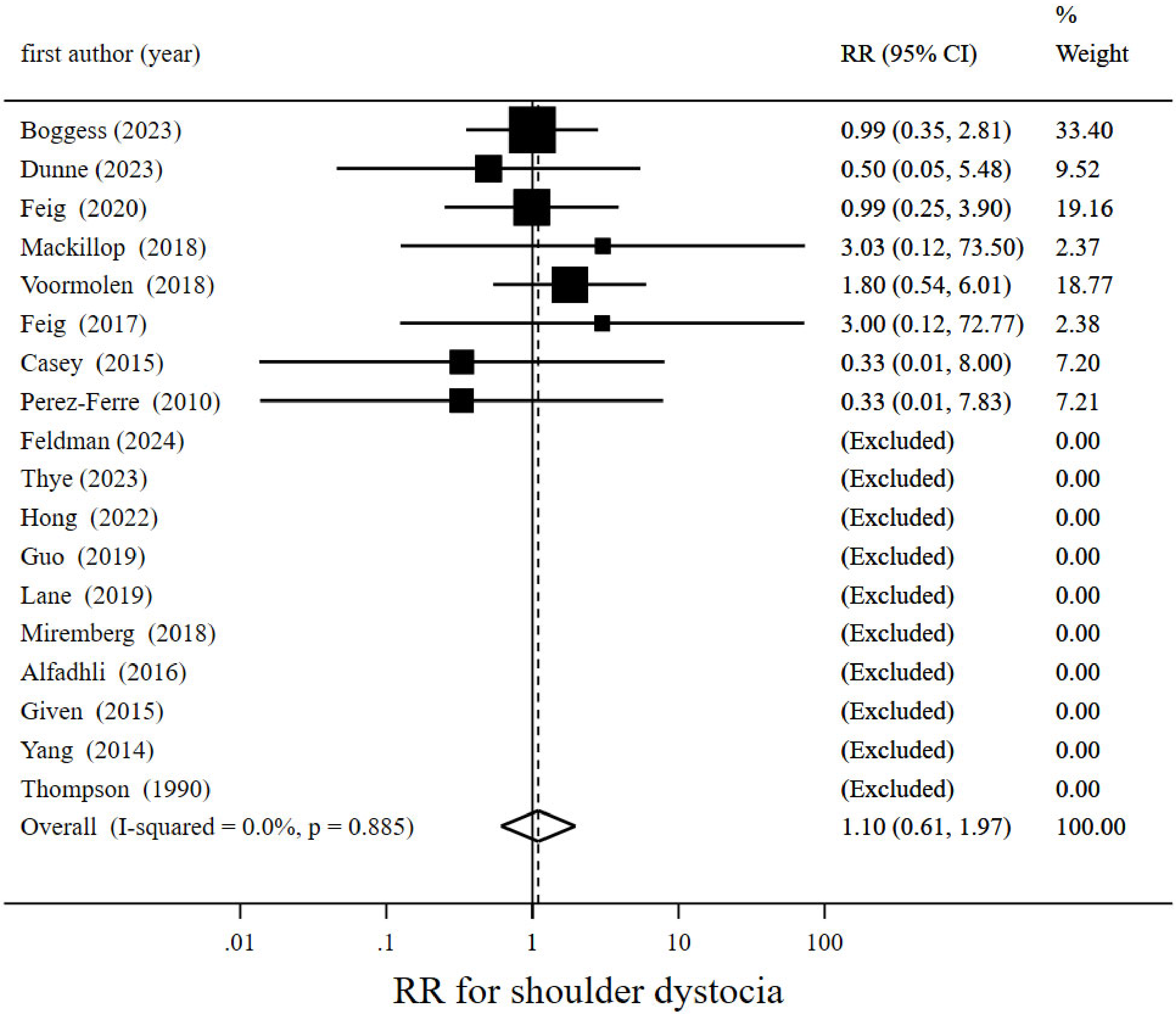
first author (year)
RR (95% CI)
% Weight
Boggess (2023) 0.99 (0.35, 2.81) 33.40
Dunne (2023) 0.50 (0.05, 5.48) 9.52
Feig (2020) 0.99 (0.25, 3.90) 19.16
Mackillop (2018) 3.03 (0.12, 73.50) 2.37
Voormolen (2018) 1.80 (0.54, 6.01) 18.77
Feig (2017) 3.00 (0.12, 72.77) 2.38
Casey (2015) 0.33 (0.01, 8.00) 7.20
Perez-Ferre (2010) 0.33 (0.01, 7.83) 7.21
Feldman (2024) (Excluded) 0.00
Thye (2023) (Excluded) 0.00
Hong (2022) (Excluded) 0.00
Guo (2019) (Excluded) 0.00
Lane (2019) (Excluded) 0.00
Miremberg (2018) (Excluded) 0.00
Alfadhli (2016) (Excluded) 0.00
Given (2015) (Excluded) 0.00
Yang (2014) (Excluded) 0.00
Thompson (1990) (Excluded) 0.00
Overall (I-squared = 0.0%, p = 0.885) 1.10 (0.61, 1.97) 100.00
.01
.1
1
10
100
RR for shoulder dystocia

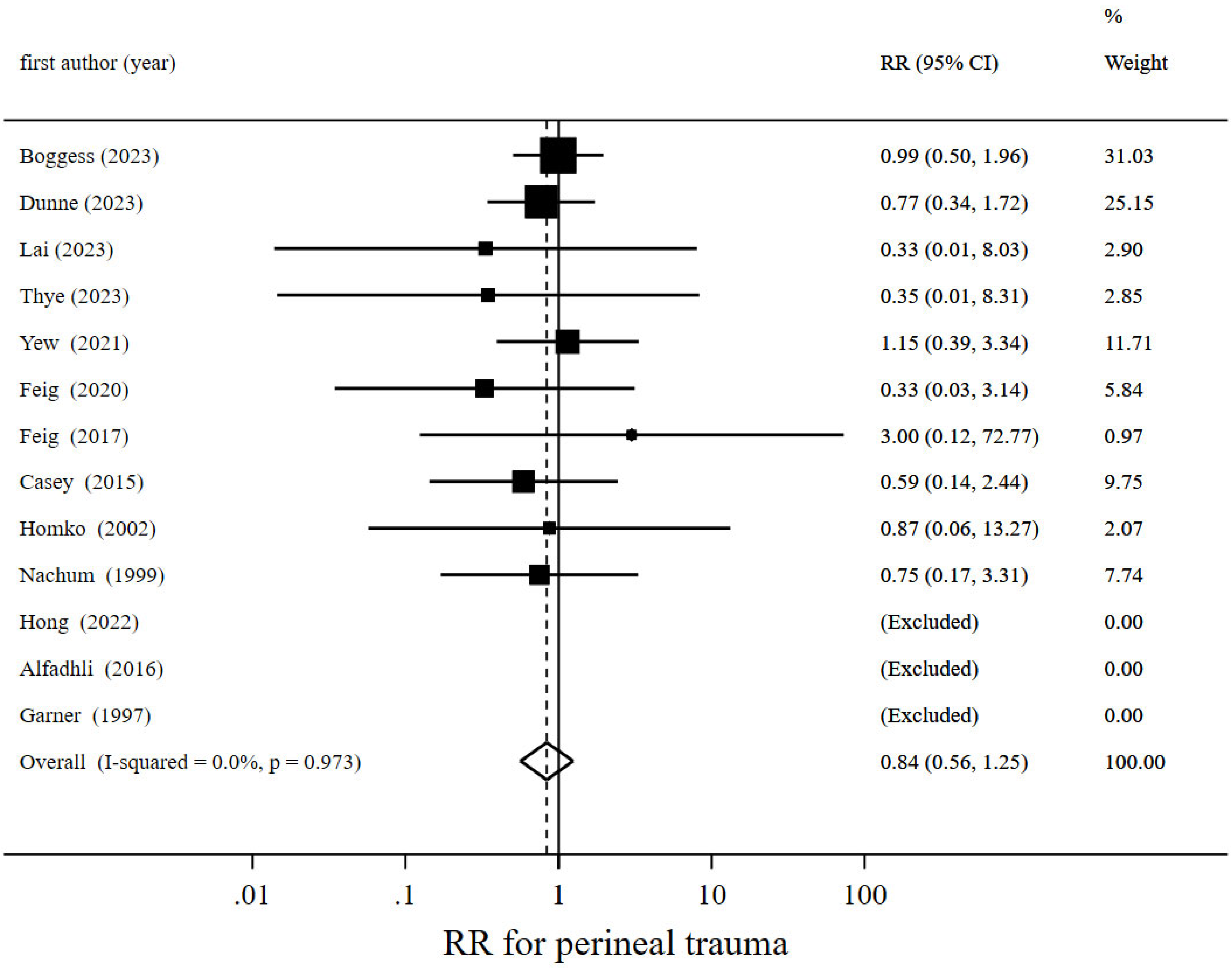
first author (year)
RR (95% CI)
%
Weight
Boggess (2023) 0.99 (0.50, 1.96) 31.03
Dunne (2023) 0.77 (0.34, 1.72) 25.15
Lai (2023) 0.33 (0.01, 8.03) 2.90
Thye (2023) 0.35 (0.01, 8.31) 2.85
Yew (2021) 1.15 (0.39, 3.34) 11.71
Feig (2020) 0.33 (0.03, 3.14) 5.84
Feig (2017) 3.00 (0.12, 72.77) 0.97
Casey (2015) 0.59 (0.14, 2.44) 9.75
Homko (2002) 0.87 (0.06, 13.27) 2.07
Nachum (1999) 0.75 (0.17, 3.31) 7.74
Hong (2022) (Excluded) 0.00
Alfadhli (2016) (Excluded) 0.00
Garner (1997) (Excluded) 0.00
Overall (I-squared = 0.0%, p = 0.973) 0.84 (0.56, 1.25) 100.00
.01
.1
1
10
100
RR for perineal trauma

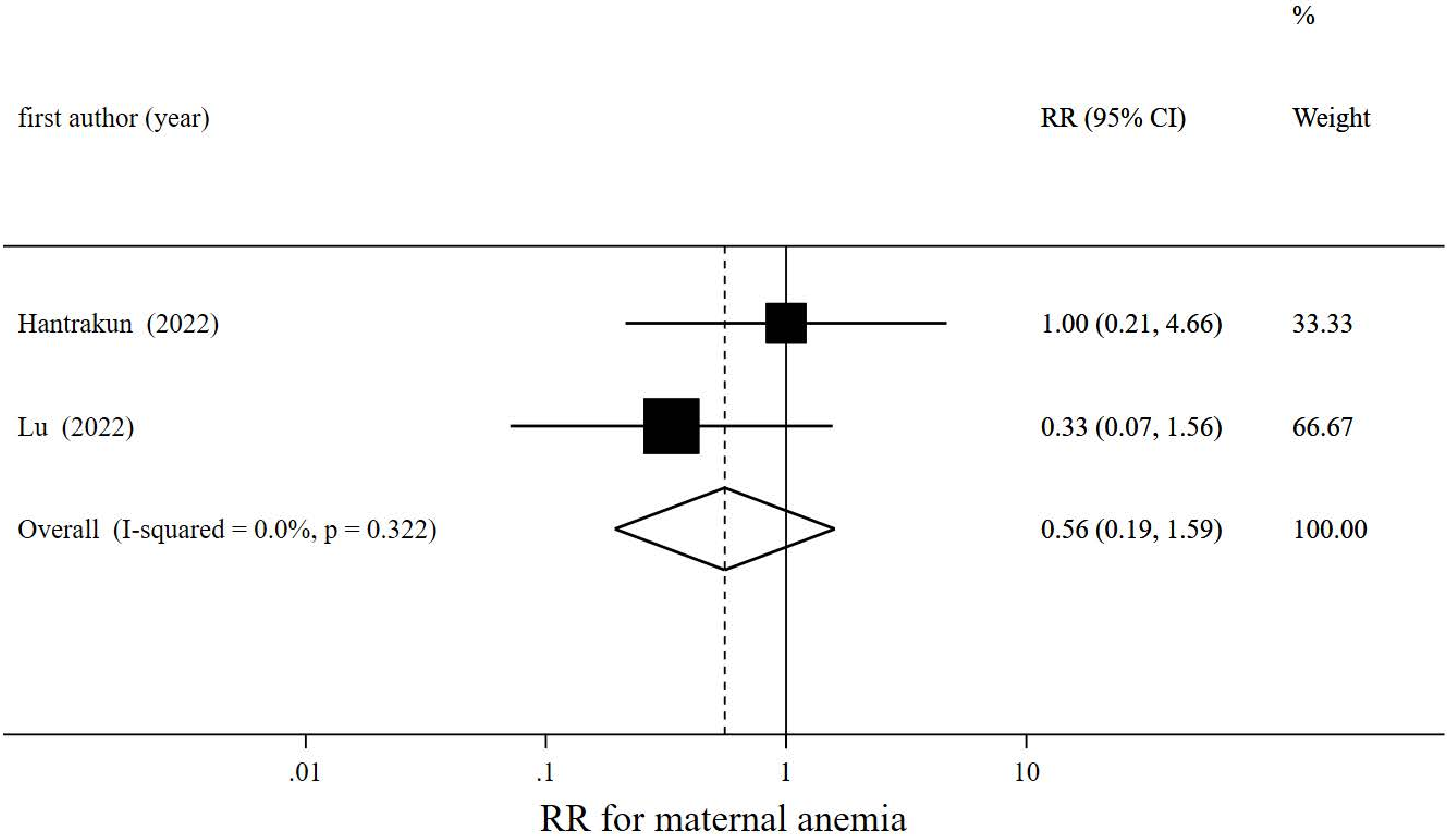
%
first author (year)
RR (95% CI)
Weight
Hantrakun (2022)
1.00 (0.21, 4.66)
33.33
Lu (2022)
0.33 (0.07, 1.56)
66.67
Overall (I-squared = 0.0%, p = 0.322)
0.56 (0.19, 1.59)
100.00
.01
.1
1
10
RR for maternal anemia

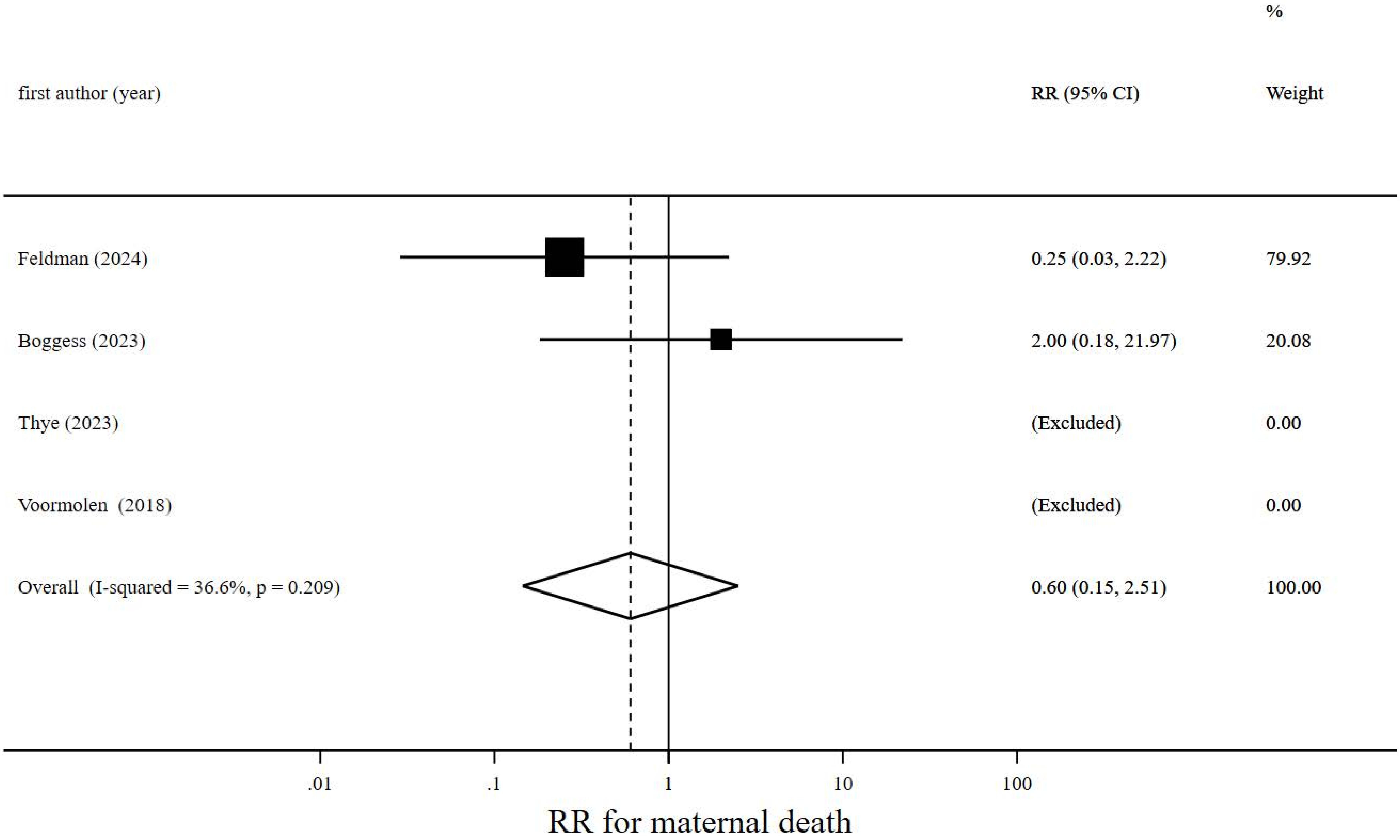
%
first author (year)
RR (95% CI)
Weight
Feldman (2024)
0.25 (0.03, 2.22)
79.92
Boggess (2023)
2.00 (0.18, 21.97)
20.08
Thye (2023)
(Excluded)
0.00
Voormolen (2018)
(Excluded)
0.00
Overall (I-squared = 36.6%, p = 0.209)
0.60 (0.15, 2.51)
100.00
.01
.1
1
10
100
RR for maternal death

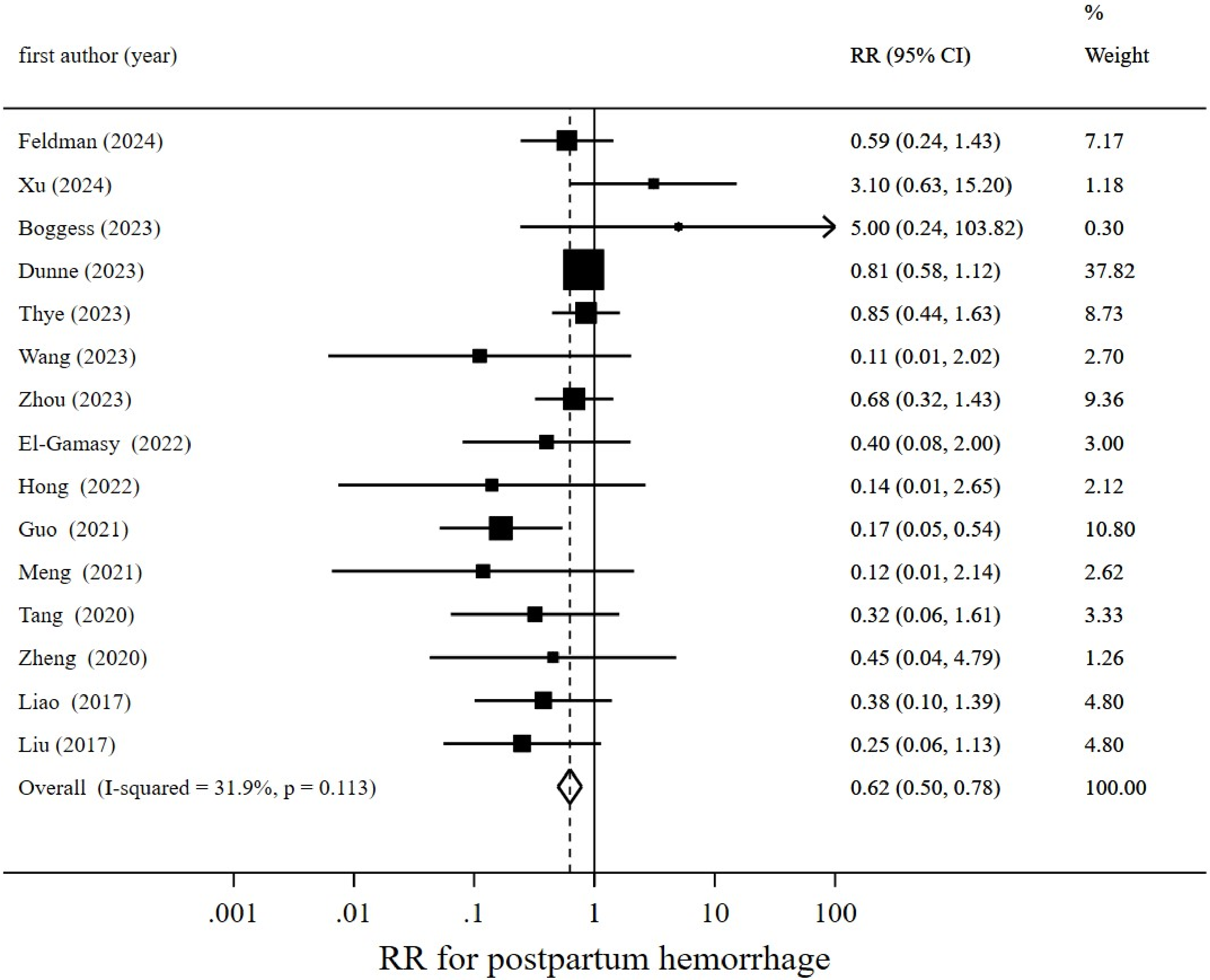

first author (year)
RR (95% CI)
% Weight
Feldman (2024) 0.59 (0.24, 1.43) 7.17
Xu (2024) 3.10 (0.63, 15.20) 1.18
Boggess (2023) 5.00 (0.24, 103.82) 0.30
Dunne (2023) 0.81 (0.58, 1.12) 37.82
Thye (2023) 0.85 (0.44, 1.63) 8.73
Wang (2023) 0.11 (0.01, 2.02) 2.70
Zhou (2023) 0.68 (0.32, 1.43) 9.36
El-Gamasy (2022) 0.40 (0.08, 2.00) 3.00
Hong (2022) 0.14 (0.01, 2.65) 2.12
Guo (2021) 0.17 (0.05, 0.54) 10.80
Meng (2021) 0.12 (0.01, 2.14) 2.62
Tang (2020) 0.32 (0.06, 1.61) 3.33
Zheng (2020) 0.45 (0.04, 4.79) 1.26
Liao (2017) 0.38 (0.10, 1.39) 4.80
Liu (2017) 0.25 (0.06, 1.13) 4.80
Overall (I-squared = 31.9%, p = 0.113) 0.62 (0.50, 0.78) 100.00
.001
.01
.1
1
10
100
RR for postpartum hemorrhage

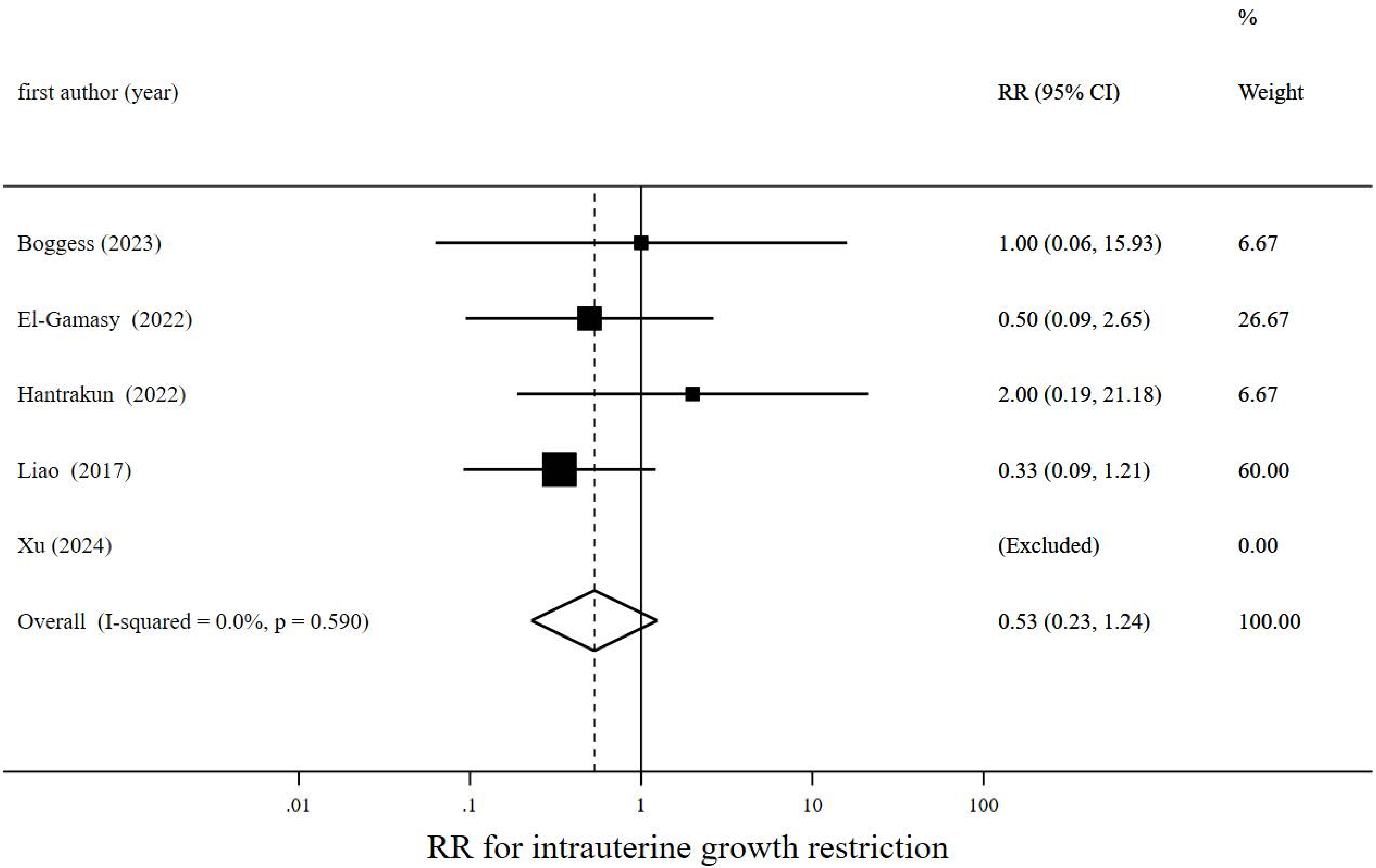

%
first author (year)
RR (95% CI)
Weight
Boggess (2023)
1.00 (0.06, 15.93)
6.67
El-Gamasy (2022)
0.50 (0.09, 2.65)
26.67
Hantrakun (2022)
2.00 (0.19, 21.18)
6.67
Liao (2017)
0.33 (0.09, 1.21)
60.00
Xu (2024)
(Excluded)
0.00
Overall (I-squared = 0.0%, p = 0.590)
0.53 (0.23, 1.24)
100.00
.01
.1
1
10
100
RR for intrauterine growth restriction

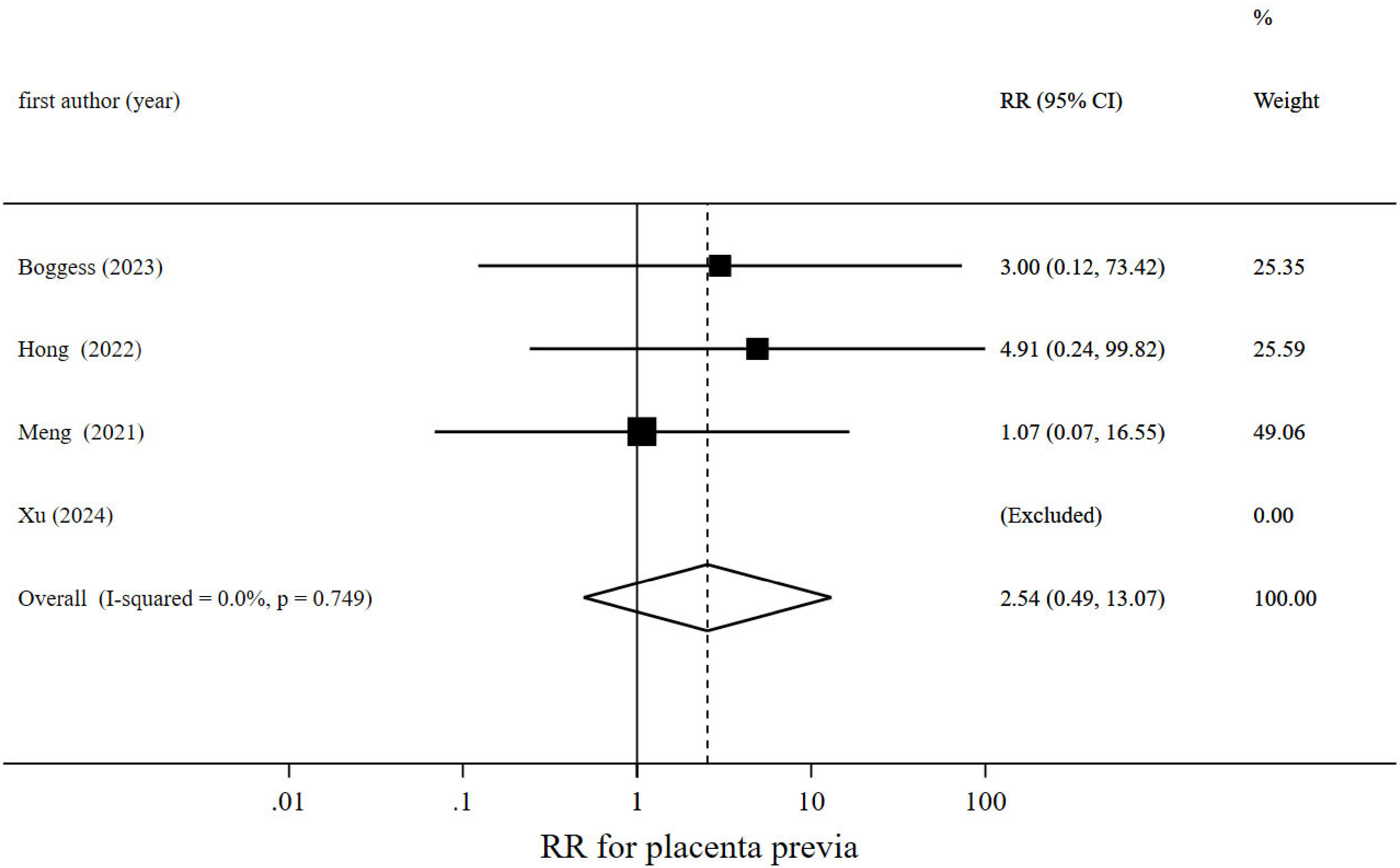
%
first author (year)
RR (95% CI)
Weight
Boggess (2023)
3.00 (0.12, 73.42)
25.35
Hong (2022)
4.91 (0.24, 99.82)
25.59
Meng (2021)
1.07 (0.07, 16.55)
49.06
Xu (2024)
(Excluded)
0.00
Overall (I-squared = 0.0%, p = 0.749)
2.54 (0.49, 13.07)
100.00
.01
.1
1
10
100
RR for placenta previa

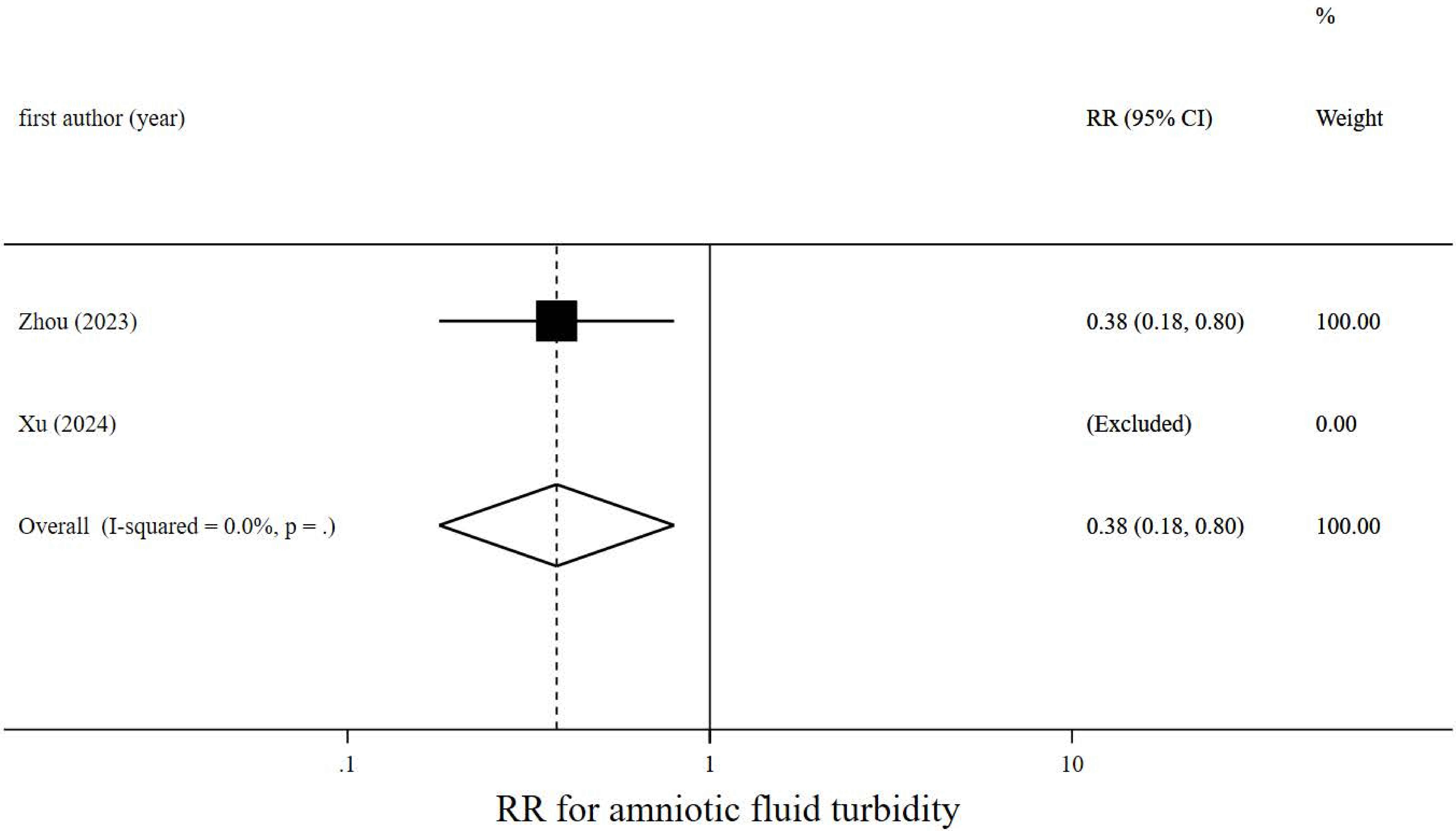
%
first author (year)
RR (95% CI)
Weight
Zhou (2023)
0.38 (0.18, 0.80)
100.00
Xu (2024)
(Excluded)
0.00
Overall (I-squared = 0.0%, p = .)
0.38 (0.18, 0.80)
100.00
.1
1
10
RR for amniotic fluid turbidity

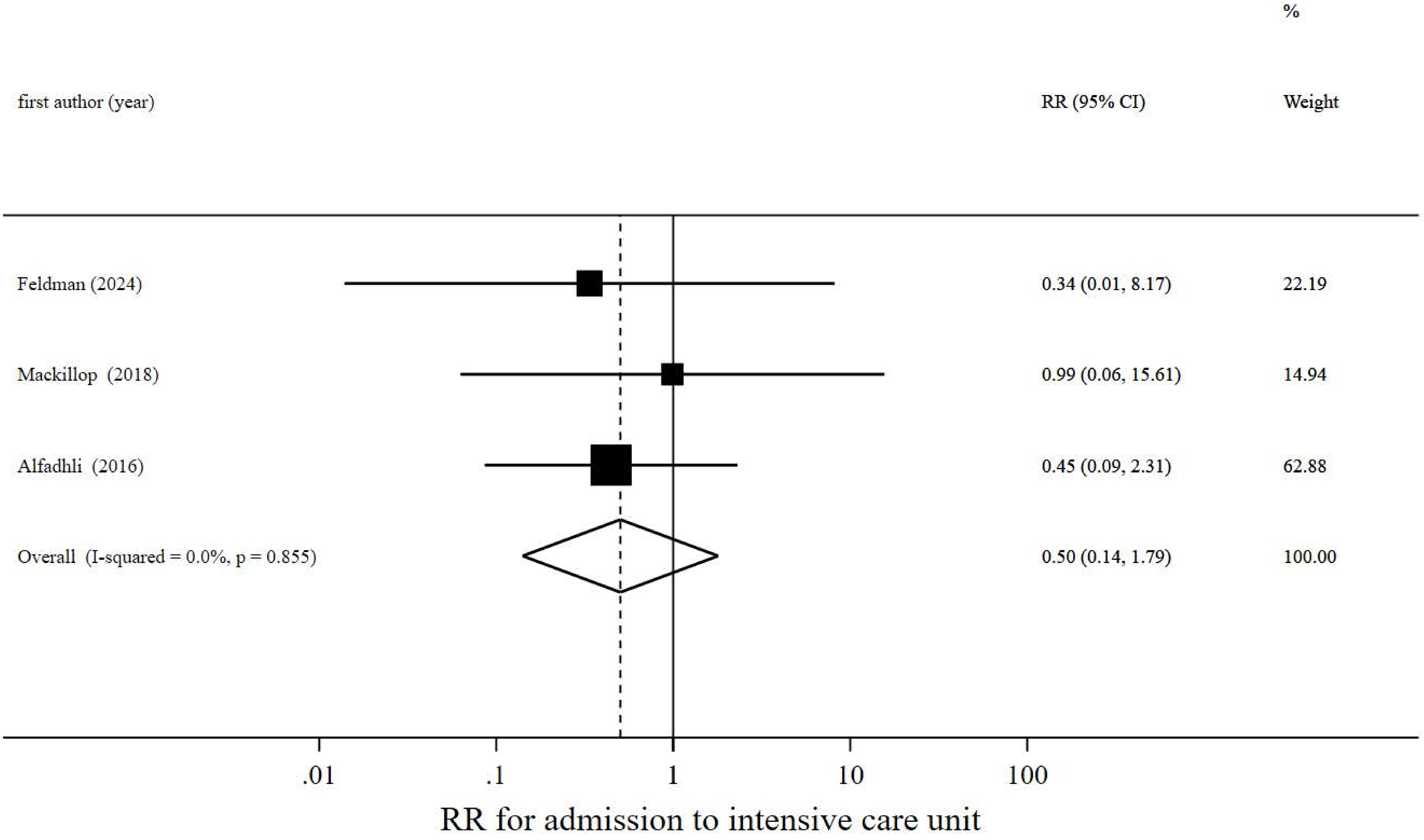
%
first author (year)
RR (95% CI)
Weight
Feldman (2024)
0.34 (0.01, 8.17)
22.19
Mackillop (2018)
0.99 (0.06, 15.61)
14.94
Alfadhli (2016)
0.45 (0.09, 2.31)
62.88
Overall (I-squared = 0.0%, p = 0.855)
0.50 (0.14, 1.79)
100.00
.01
.1
1
10
100
RR for admission to intensive care unit

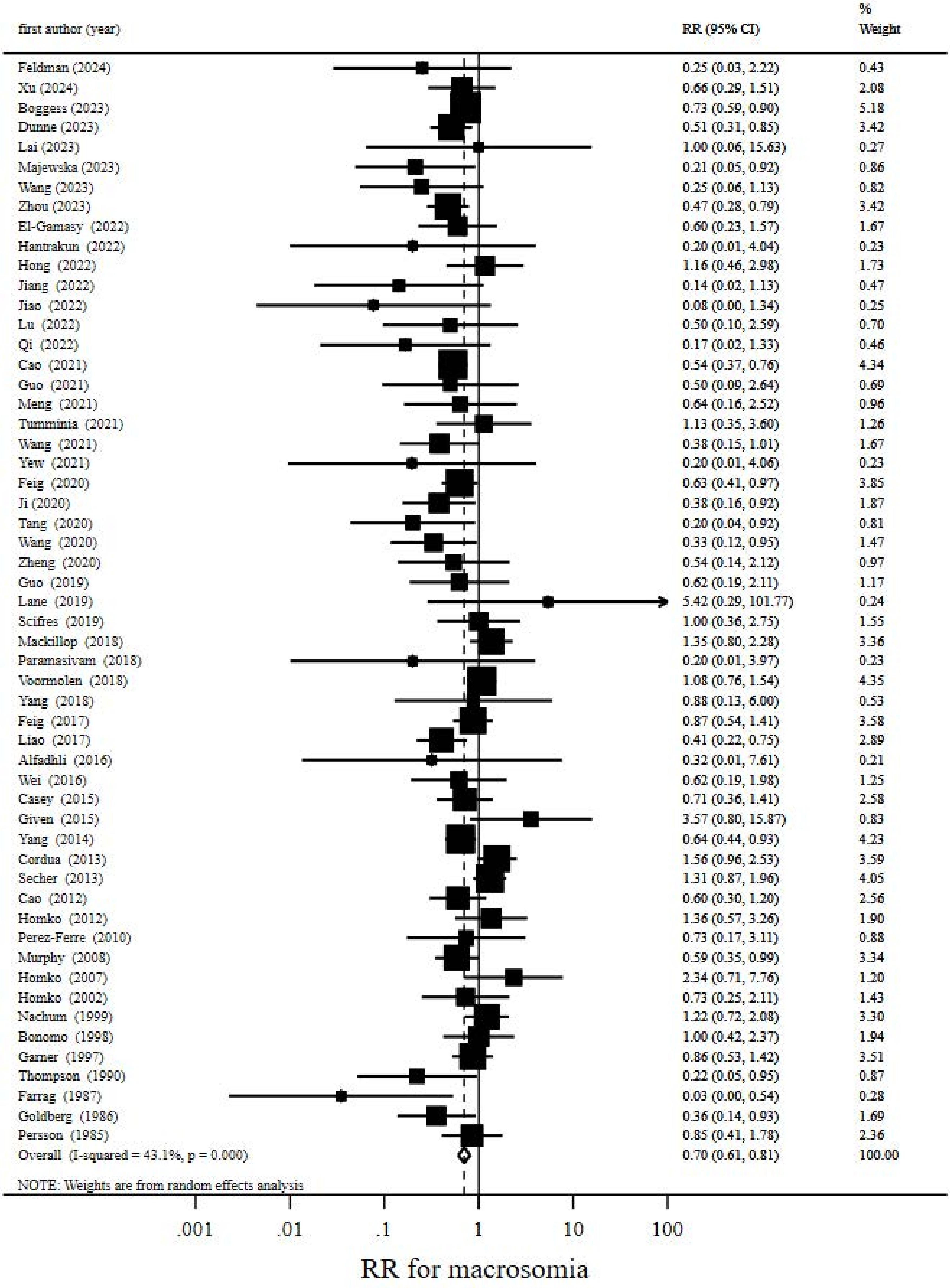
first author (year)
RR (95% CI)
% Weight
Feldman (2024) 0.25 (0.03, 2.22) 0.43
Xu (2024) 0.66 (0.29, 1.51) 2.08
Boggess (2023) 0.73 (0.59, 0.90) 5.18
Dunne (2023) 0.51 (0.31, 0.85) 3.42
Lai (2023) 1.00 (0.06, 15.63) 0.27
Majewska (2023) 0.21 (0.05, 0.92) 0.86
Wang (2023) 0.25 (0.06, 1.13) 0.82
Zhou (2023) 0.47 (0.28, 0.79) 3.42
El-Gamasy (2022) 0.60 (0.23, 1.57) 1.67
Hantrakun (2022) 0.20 (0.01, 4.04) 0.23
Hong (2022) 1.16 (0.46, 2.98) 1.73
Jiang (2022) 0.14 (0.02, 1.13) 0.47
Jiao (2022) 0.08 (0.00, 1.34) 0.25
Lu (2022) 0.50 (0.10, 2.59) 0.70
Qi (2022) 0.17 (0.02, 1.33) 0.46
Cao (2021) 0.54 (0.37, 0.76) 4.34
Guo (2021) 0.50 (0.09, 2.64) 0.69
Meng (2021) 0.64 (0.16, 2.52) 0.96
Tumminia (2021) 1.13 (0.35, 3.60) 1.26
Wang (2021) 0.38 (0.15, 1.01) 1.67
Yew (2021) 0.20 (0.01, 4.06) 0.23
Feig (2020) 0.63 (0.41, 0.97) 3.85
Ji (2020) 0.38 (0.16, 0.92) 1.87
Tang (2020) 0.20 (0.04, 0.92) 0.81
Wang (2020) 0.33 (0.12, 0.95) 1.47
Zheng (2020) 0.54 (0.14, 2.12) 0.97
Guo (2019) 0.62 (0.19, 2.11) 1.17
Lane (2019) 5.42 (0.29, 101.77) 0.24
Scifres (2019) 1.00 (0.36, 2.75) 1.55
Mackillop (2018) 1.35 (0.80, 2.28) 3.36
Paramasivam (2018) 0.20 (0.01, 3.97) 0.23
Voormolen (2018) 1.08 (0.76, 1.54) 4.35
Yang (2018) 0.88 (0.13, 6.00) 0.53
Feig (2017) 0.87 (0.54, 1.41) 3.58
Liao (2017) 0.41 (0.22, 0.75) 2.89
Alfadhli (2016) 0.32 (0.01, 7.61) 0.21
Wei (2016) 0.62 (0.19, 1.98) 1.25
Casey (2015) 0.71 (0.36, 1.41) 2.58
Given (2015) 3.57 (0.80, 15.87) 0.83
Yang (2014) 0.64 (0.44, 0.93) 4.23
Cordua (2013) 1.56 (0.96, 2.53) 3.59
Secher (2013) 1.31 (0.87, 1.96) 4.05
Cao (2012) 0.60 (0.30, 1.20) 2.56
Homko (2012) 1.36 (0.57, 3.26) 1.90
Perez-Ferre (2010) 0.73 (0.17, 3.11) 0.88
Murphy (2008) 0.59 (0.35, 0.99) 3.34
Homko (2007) 2.34 (0.71, 7.76) 1.20
Homko (2002) 0.73 (0.25, 2.11) 1.43
Nachum (1999) 1.22 (0.72, 2.08) 3.30
Bonomo (1998) 1.00 (0.42, 2.37) 1.94
Garner (1997) 0.86 (0.53, 1.42) 3.51
Thompson (1990) 0.22 (0.05, 0.95) 0.87
Farrag (1987) 0.03 (0.00, 0.54) 0.28
Goldberg (1986) 0.36 (0.14, 0.93) 1.69
Persson (1985) 0.85 (0.41, 1.78) 2.36
Overall (I-squared = 43.1%, p = 0.000) 0.70 (0.61, 0.81) 100.00
NOTE: Weights are from random effects analysis
.001
.01
.1
1
10
100
RR for macrosomia

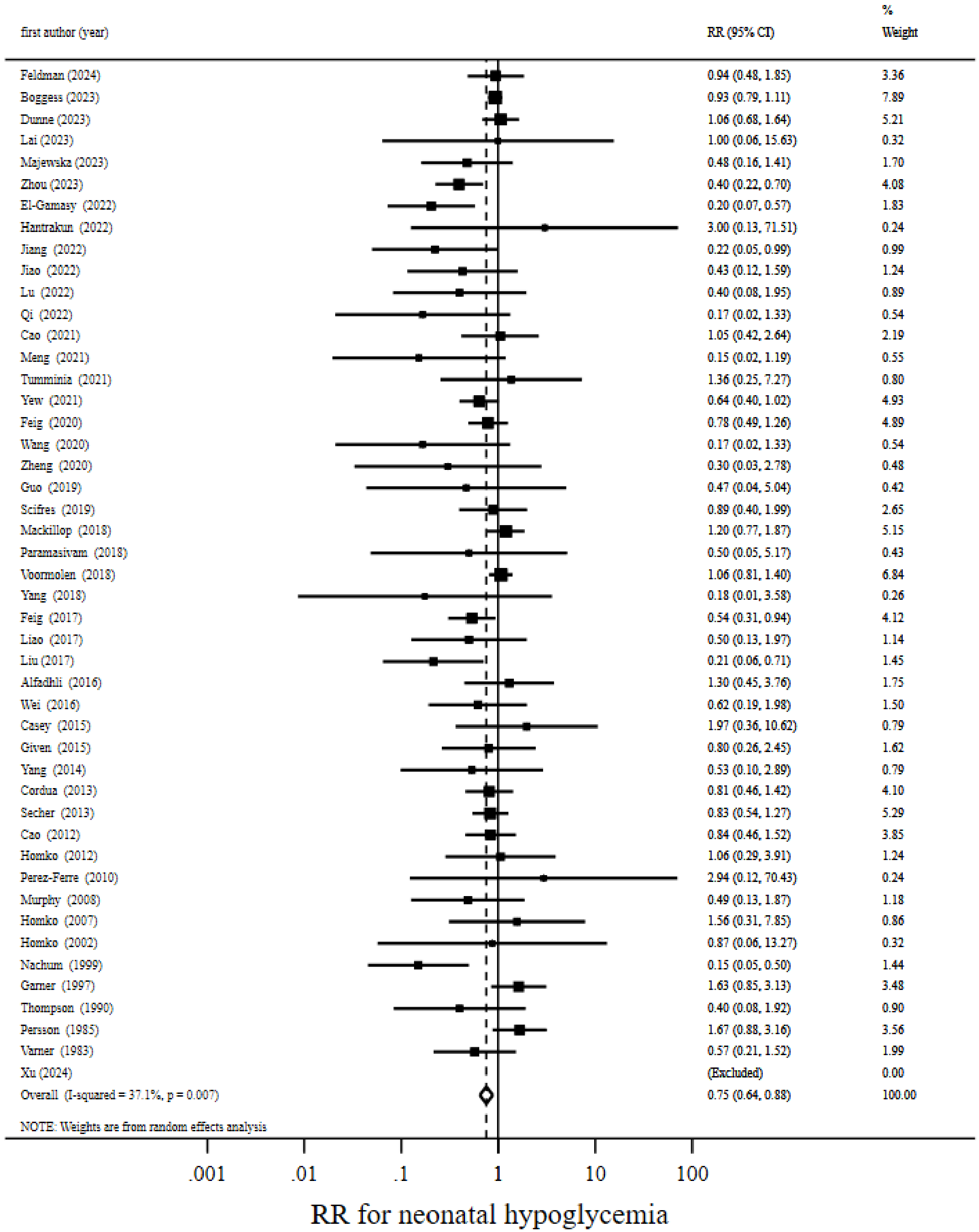
first author (year)
RR (95% CI)
%
Weight
Feldman (2024) 0.94 (0.48, 1.85) 3.36
Boggess (2023) 0.93 (0.79, 1.11) 7.89
Dunne (2023) 1.06 (0.68, 1.64) 5.21
Lai (2023) 1.00 (0.06, 15.63) 0.32
Majewska (2023) 0.48 (0.16, 1.41) 1.70
Zhou (2023) 0.40 (0.22, 0.70) 4.08
El-Gamasy (2022) 0.20 (0.07, 0.57) 1.83
Hantrakun (2022) 3.00 (0.13, 71.51) 0.24
Jiang (2022) 0.22 (0.05, 0.99) 0.99
Jiao (2022) 0.43 (0.12, 1.59) 1.24
Lu (2022) 0.40 (0.08, 1.95) 0.89
Qi (2022) 0.17 (0.02, 1.33) 0.54
Cao (2021) 1.05 (0.42, 2.64) 2.19
Meng (2021) 0.15 (0.02, 1.19) 0.55
Tumminia (2021) 1.36 (0.25, 7.27) 0.80
Yew (2021) 0.64 (0.40, 1.02) 4.93
Feig (2020) 0.78 (0.49, 1.26) 4.89
Wang (2020) 0.17 (0.02, 1.33) 0.54
Zheng (2020) 0.30 (0.03, 2.78) 0.48
Guo (2019) 0.47 (0.04, 5.04) 0.42
Scifres (2019) 0.89 (0.40, 1.99) 2.65
Mackillop (2018) 1.20 (0.77, 1.87) 5.15
Paramasivam (2018) 0.50 (0.05, 5.17) 0.43
Voormolen (2018) 1.06 (0.81, 1.40) 6.84
Yang (2018) 0.18 (0.01, 3.58) 0.26
Feig (2017) 0.54 (0.31, 0.94) 4.12
Liao (2017) 0.50 (0.13, 1.97) 1.14
Liu (2017) 0.21 (0.06, 0.71) 1.45
Alfadhli (2016) 1.30 (0.45, 3.76) 1.75
Wei (2016) 0.62 (0.19, 1.98) 1.50
Casey (2015) 1.97 (0.36, 10.62) 0.79
Given (2015) 0.80 (0.26, 2.45) 1.62
Yang (2014) 0.53 (0.10, 2.89) 0.79
Cordua (2013) 0.81 (0.46, 1.42) 4.10
Secher (2013) 0.83 (0.54, 1.27) 5.29
Cao (2012) 0.84 (0.46, 1.52) 3.85
Homko (2012) 1.06 (0.29, 3.91) 1.24
Perez-Ferre (2010) 2.94 (0.12, 70.43) 0.24
Murphy (2008) 0.49 (0.13, 1.87) 1.18
Homko (2007) 1.56 (0.31, 7.85) 0.86
Homko (2002) 0.87 (0.06, 13.27) 0.32
Nachum (1999) 0.15 (0.05, 0.50) 1.44
Garner (1997) 1.63 (0.85, 3.13) 3.48
Thompson (1990) 0.40 (0.08, 1.92) 0.90
Persson (1985) 1.67 (0.88, 3.16) 3.56
Varner (1983) 0.57 (0.21, 1.52) 1.99
Xu (2024) (Excluded) 0.00
Overall (I-squared = 37.1%, p = 0.007) 0.75 (0.64, 0.88) 100.00
NOTE: Weights are from random effects analysis
.001
.01
.1
1
10
100
RR for neonatal hypoglycemia

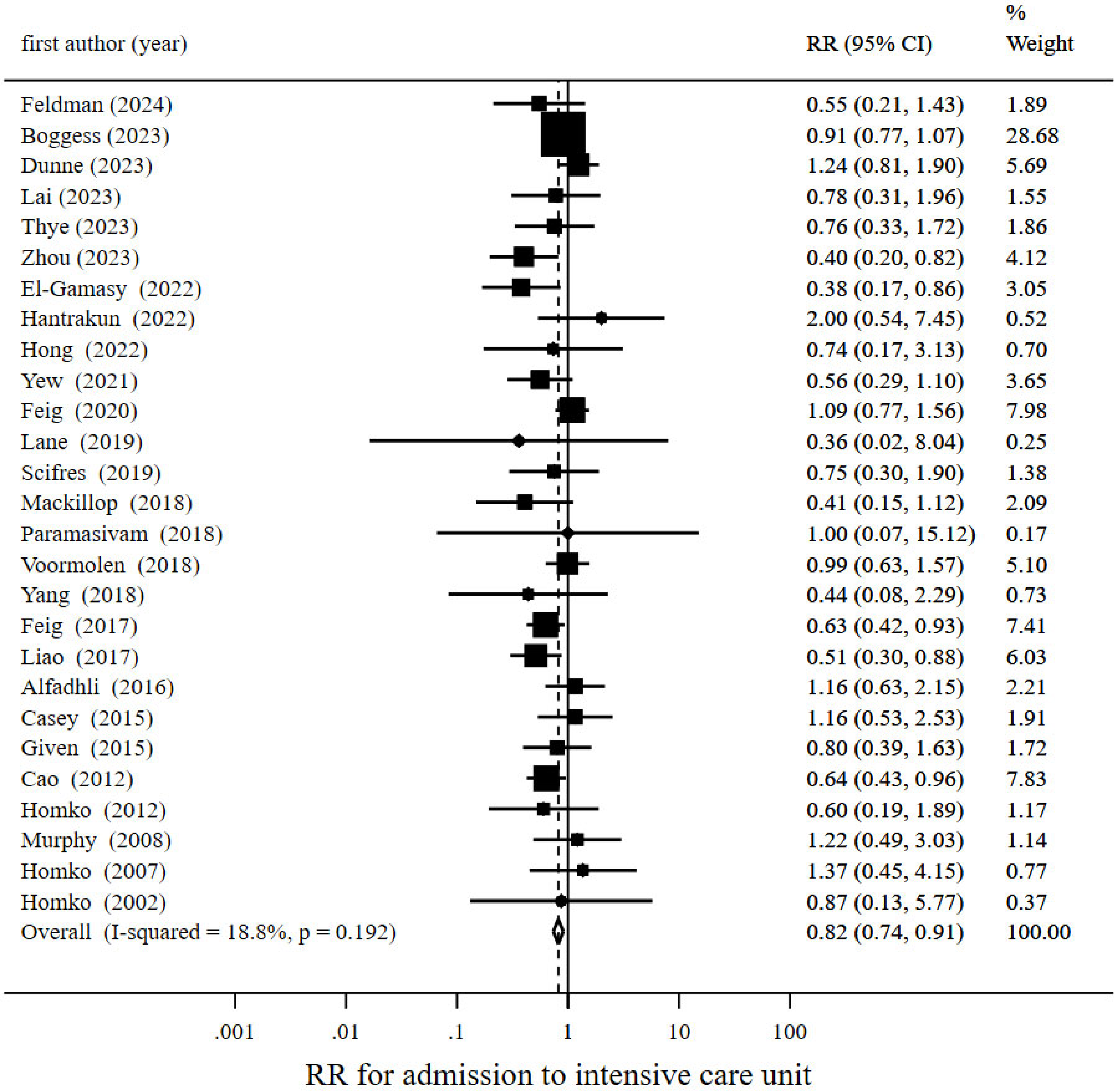
first author (year)
RR (95% CI)
% Weight
Feldman (2024) 0.55 (0.21, 1.43) 1.89
Boggess (2023) 0.91 (0.77, 1.07) 28.68
Dunne (2023) 1.24 (0.81, 1.90) 5.69
Lai (2023) 0.78 (0.31, 1.96) 1.55
Thye (2023) 0.76 (0.33, 1.72) 1.86
Zhou (2023) 0.40 (0.20, 0.82) 4.12
El-Gamasy (2022) 0.38 (0.17, 0.86) 3.05
Hantrakun (2022) 2.00 (0.54, 7.45) 0.52
Hong (2022) 0.74 (0.17, 3.13) 0.70
Yew (2021) 0.56 (0.29, 1.10) 3.65
Feig (2020) 1.09 (0.77, 1.56) 7.98
Lane (2019) 0.36 (0.02, 8.04) 0.25
Scifres (2019) 0.75 (0.30, 1.90) 1.38
Mackillop (2018) 0.41 (0.15, 1.12) 2.09
Paramasivam (2018) 1.00 (0.07, 15.12) 0.17
Voormolen (2018) 0.99 (0.63, 1.57) 5.10
Yang (2018) 0.44 (0.08, 2.29) 0.73
Feig (2017) 0.63 (0.42, 0.93) 7.41
Liao (2017) 0.51 (0.30, 0.88) 6.03
Alfadhli (2016) 1.16 (0.63, 2.15) 2.21
Casey (2015) 1.16 (0.53, 2.53) 1.91
Given (2015) 0.80 (0.39, 1.63) 1.72
Cao (2012) 0.64 (0.43, 0.96) 7.83
Homko (2012) 0.60 (0.19, 1.89) 1.17
Murphy (2008) 1.22 (0.49, 3.03) 1.14
Homko (2007) 1.37 (0.45, 4.15) 0.77
Homko (2002) 0.87 (0.13, 5.77) 0.37
Overall (I-squared = 18.8%, p = 0.192) 0.82 (0.74, 0.91) 100.00
.001
.01
.1
1
10
100
RR for admission to intensive care unit

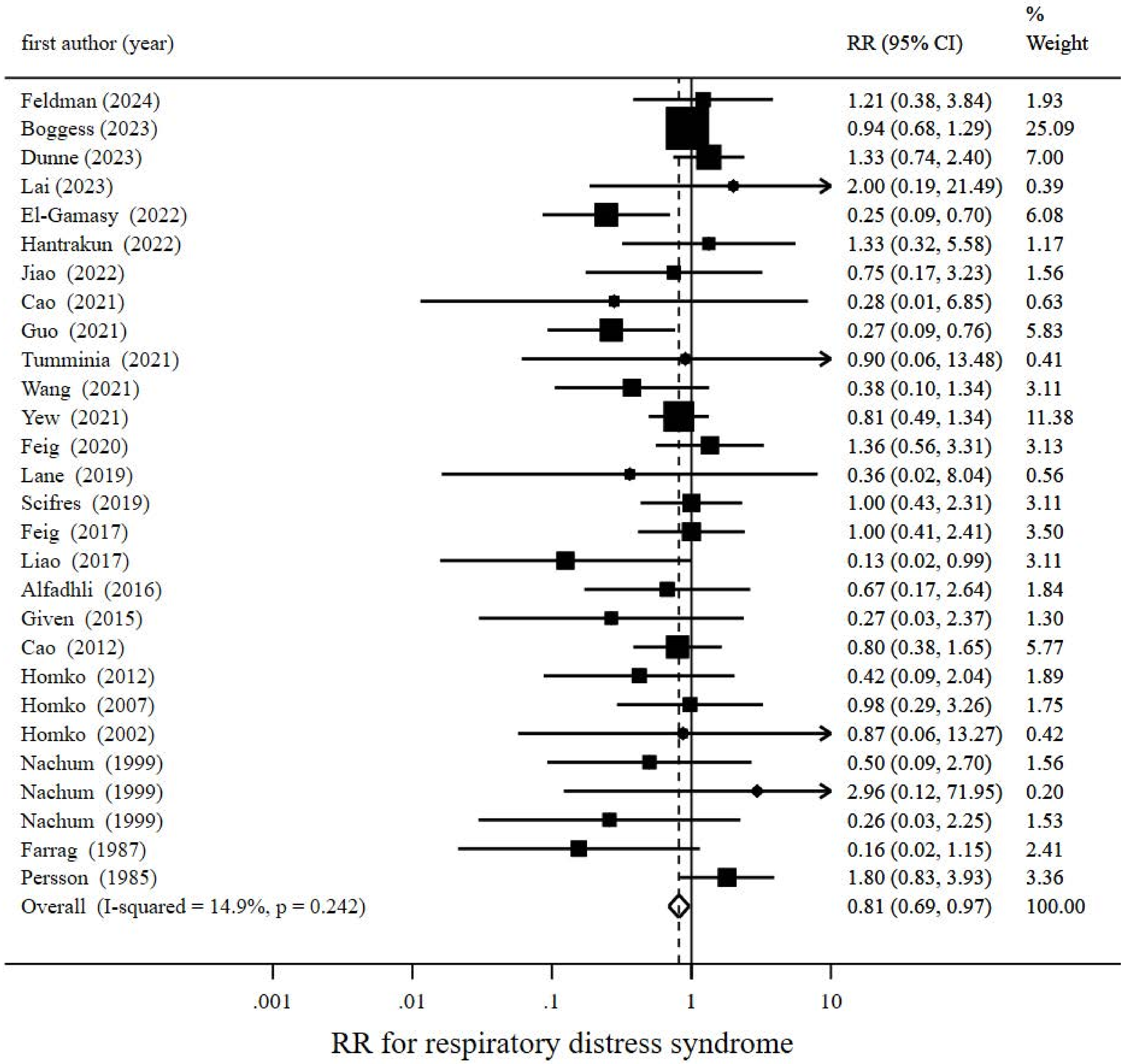
first author (year)
RR (95% CI)
% Weight
Feldman (2024) 1.21 (0.38, 3.84) 1.93
Boggess (2023) 0.94 (0.68, 1.29) 25.09
Dunne (2023) 1.33 (0.74, 2.40) 7.00
Lai (2023) 2.00 (0.19, 21.49) 0.39
El-Gamasy (2022) 0.25 (0.09, 0.70) 6.08
Hantrakun (2022) 1.33 (0.32, 5.58) 1.17
Jiao (2022) 0.75 (0.17, 3.23) 1.56
Cao (2021) 0.28 (0.01, 6.85) 0.63
Guo (2021) 0.27 (0.09, 0.76) 5.83
Tumminia (2021) 0.90 (0.06, 13.48) 0.41
Wang (2021) 0.38 (0.10, 1.34) 3.11
Yew (2021) 0.81 (0.49, 1.34) 11.38
Feig (2020) 1.36 (0.56, 3.31) 3.13
Lane (2019) 0.36 (0.02, 8.04) 0.56
Scifres (2019) 1.00 (0.43, 2.31) 3.11
Feig (2017) 1.00 (0.41, 2.41) 3.50
Liao (2017) 0.13 (0.02, 0.99) 3.11
Alfadhli (2016) 0.67 (0.17, 2.64) 1.84
Given (2015) 0.27 (0.03, 2.37) 1.30
Cao (2012) 0.80 (0.38, 1.65) 5.77
Homko (2012) 0.42 (0.09, 2.04) 1.89
Homko (2007) 0.98 (0.29, 3.26) 1.75
Homko (2002) 0.87 (0.06, 13.27) 0.42
Nachum (1999) 0.50 (0.09, 2.70) 1.56
Nachum (1999) 2.96 (0.12, 71.95) 0.20
Nachum (1999) 0.26 (0.03, 2.25) 1.53
Farrag (1987) 0.16 (0.02, 1.15) 2.41
Persson (1985) 1.80 (0.83, 3.93) 3.36
Overall (I-squared = 14.9%, p = 0.242) 0.81 (0.69, 0.97) 100.00
.001
.01
.1
1
10
RR for respiratory distress syndrome

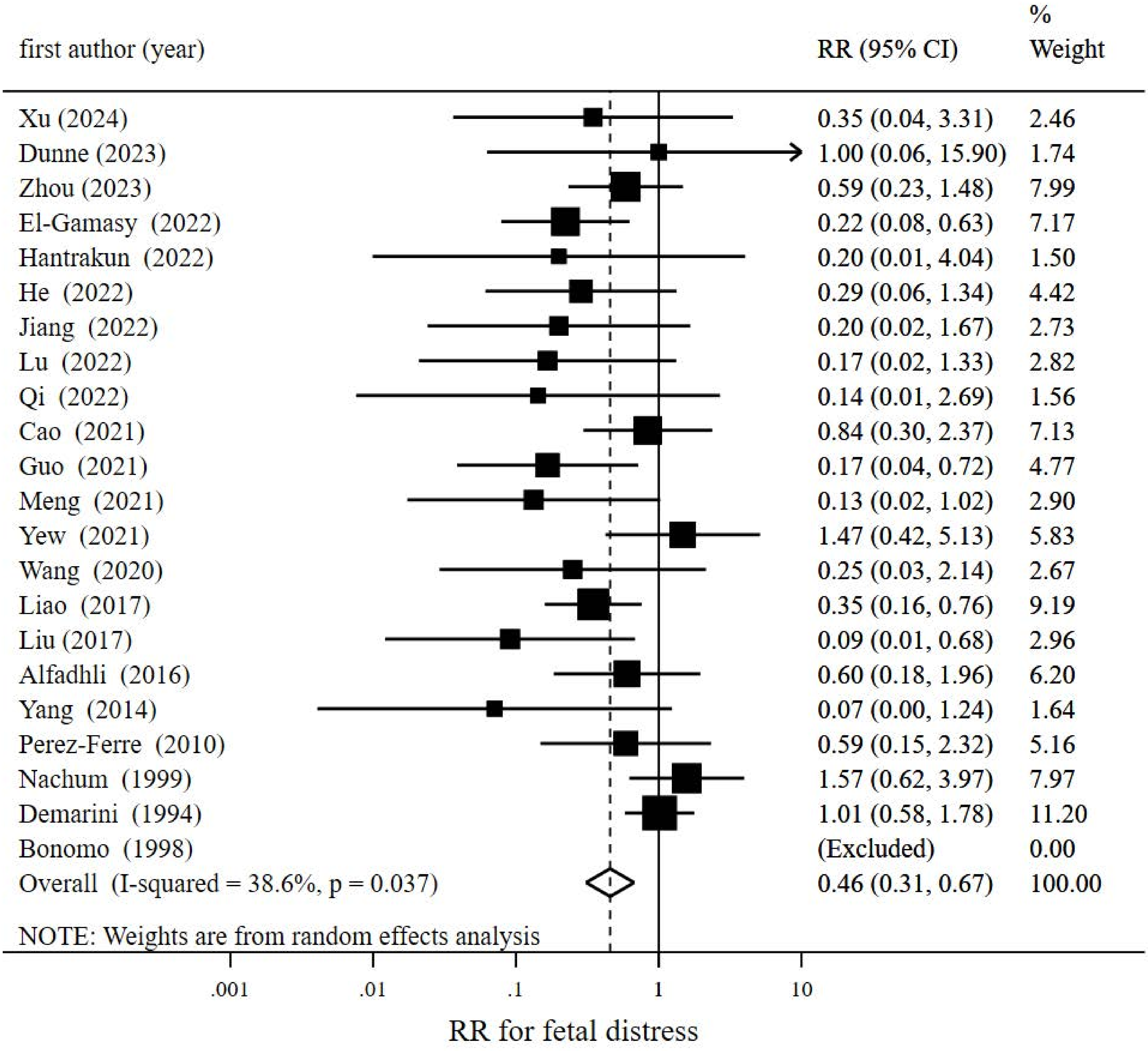
first author (year)
RR (95% CI)
% Weight
Xu (2024) 0.35 (0.04, 3.31) 2.46
Dunne (2023) 1.00 (0.06, 15.90) 1.74
Zhou (2023) 0.59 (0.23, 1.48) 7.99
El-Gamasy (2022) 0.22 (0.08, 0.63) 7.17
Hantrakun (2022) 0.20 (0.01, 4.04) 1.50
He (2022) 0.29 (0.06, 1.34) 4.42
Jiang (2022) 0.20 (0.02, 1.67) 2.73
Lu (2022) 0.17 (0.02, 1.33) 2.82
Qi (2022) 0.14 (0.01, 2.69) 1.56
Cao (2021) 0.84 (0.30, 2.37) 7.13
Guo (2021) 0.17 (0.04, 0.72) 4.77
Meng (2021) 0.13 (0.02, 1.02) 2.90
Yew (2021) 1.47 (0.42, 5.13) 5.83
Wang (2020) 0.25 (0.03, 2.14) 2.67
Liao (2017) 0.35 (0.16, 0.76) 9.19
Liu (2017) 0.09 (0.01, 0.68) 2.96
Alfadhli (2016) 0.60 (0.18, 1.96) 6.20
Yang (2014) 0.07 (0.00, 1.24) 1.64
Perez-Ferre (2010) 0.59 (0.15, 2.32) 5.16
Nachum (1999) 1.57 (0.62, 3.97) 7.97
Demarini (1994) 1.01 (0.58, 1.78) 11.20
Bonomo (1998) (Excluded) 0.00
Overall (I-squared = 38.6%, p = 0.037) 0.46 (0.31, 0.67) 100.00
NOTE: Weights are from random effects analysis
.001
.01
.1
1
10
RR for fetal distress

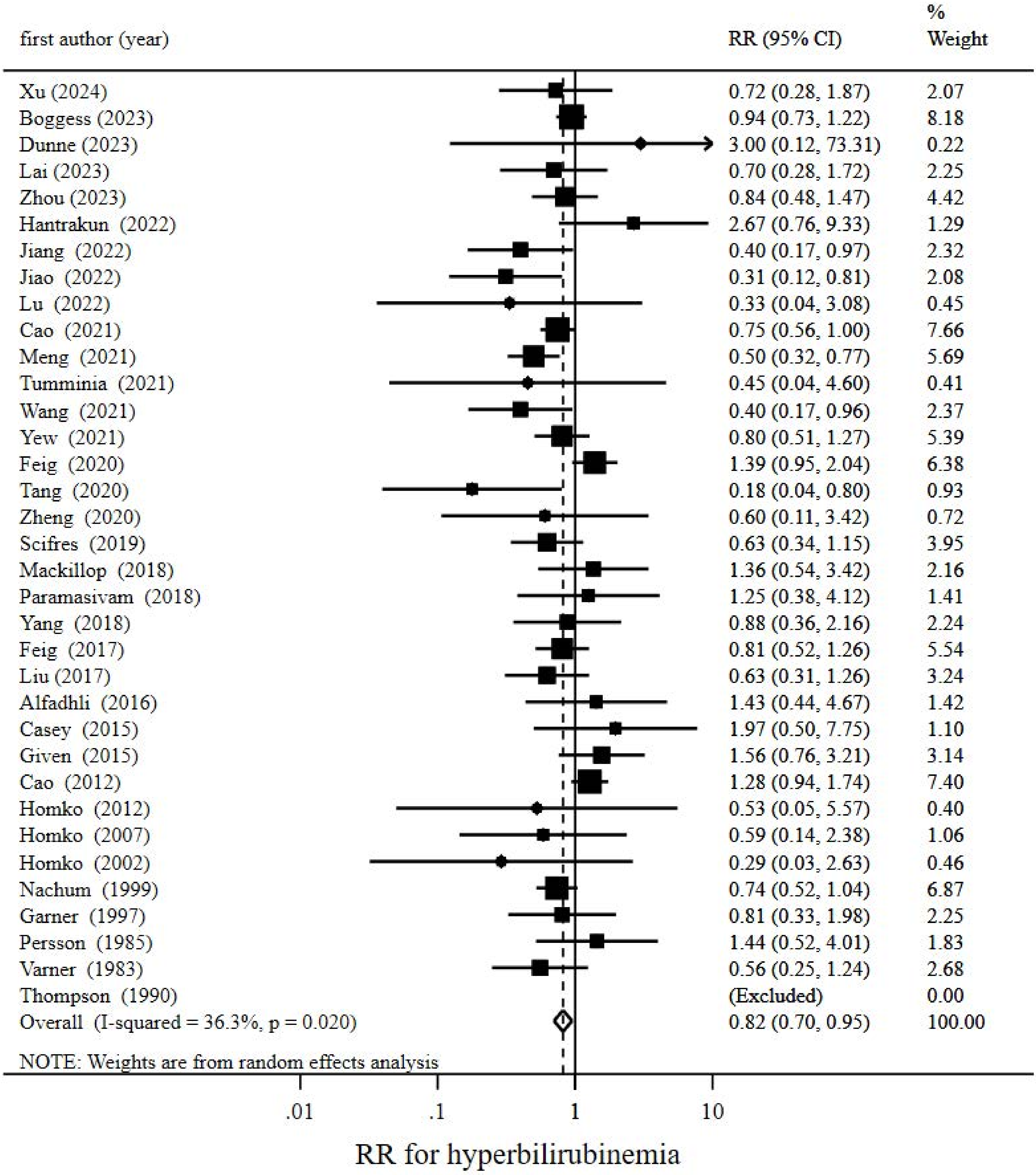
first author (year)
RR (95% CI)
% Weight
Xu (2024) 0.72 (0.28, 1.87) 2.07
Boggess (2023) 0.94 (0.73, 1.22) 8.18
Dunne (2023) 3.00 (0.12, 73.31) 0.22
Lai (2023) 0.70 (0.28, 1.72) 2.25
Zhou (2023) 0.84 (0.48, 1.47) 4.42
Hantrakun (2022) 2.67 (0.76, 9.33) 1.29
Jiang (2022) 0.40 (0.17, 0.97) 2.32
Jiao (2022) 0.31 (0.12, 0.81) 2.08
Lu (2022) 0.33 (0.04, 3.08) 0.45
Cao (2021) 0.75 (0.56, 1.00) 7.66
Meng (2021) 0.50 (0.32, 0.77) 5.69
Tumminia (2021) 0.45 (0.04, 4.60) 0.41
Wang (2021) 0.40 (0.17, 0.96) 2.37
Yew (2021) 0.80 (0.51, 1.27) 5.39
Feig (2020) 1.39 (0.95, 2.04) 6.38
Tang (2020) 0.18 (0.04, 0.80) 0.93
Zheng (2020) 0.60 (0.11, 3.42) 0.72
Scifres (2019) 0.63 (0.34, 1.15) 3.95
Mackillop (2018) 1.36 (0.54, 3.42) 2.16
Paramasivam (2018) 1.25 (0.38, 4.12) 1.41
Yang (2018) 0.88 (0.36, 2.16) 2.24
Feig (2017) 0.81 (0.52, 1.26) 5.54
Liu (2017) 0.63 (0.31, 1.26) 3.24
Alfadhli (2016) 1.43 (0.44, 4.67) 1.42
Casey (2015) 1.97 (0.50, 7.75) 1.10
Given (2015) 1.56 (0.76, 3.21) 3.14
Cao (2012) 1.28 (0.94, 1.74) 7.40
Homko (2012) 0.53 (0.05, 5.57) 0.40
Homko (2007) 0.59 (0.14, 2.38) 1.06
Homko (2002) 0.29 (0.03, 2.63) 0.46
Nachum (1999) 0.74 (0.52, 1.04) 6.87
Garner (1997) 0.81 (0.33, 1.98) 2.25
Persson (1985) 1.44 (0.52, 4.01) 1.83
Varner (1983) 0.56 (0.25, 1.24) 2.68
Thompson (1990) (Excluded) 0.00
Overall (I-squared = 36.3%, p = 0.020) 0.82 (0.70, 0.95) 100.00
NOTE: Weights are from random effects analysis
.01
.1
1
10
RR for hyperbilirubinemia

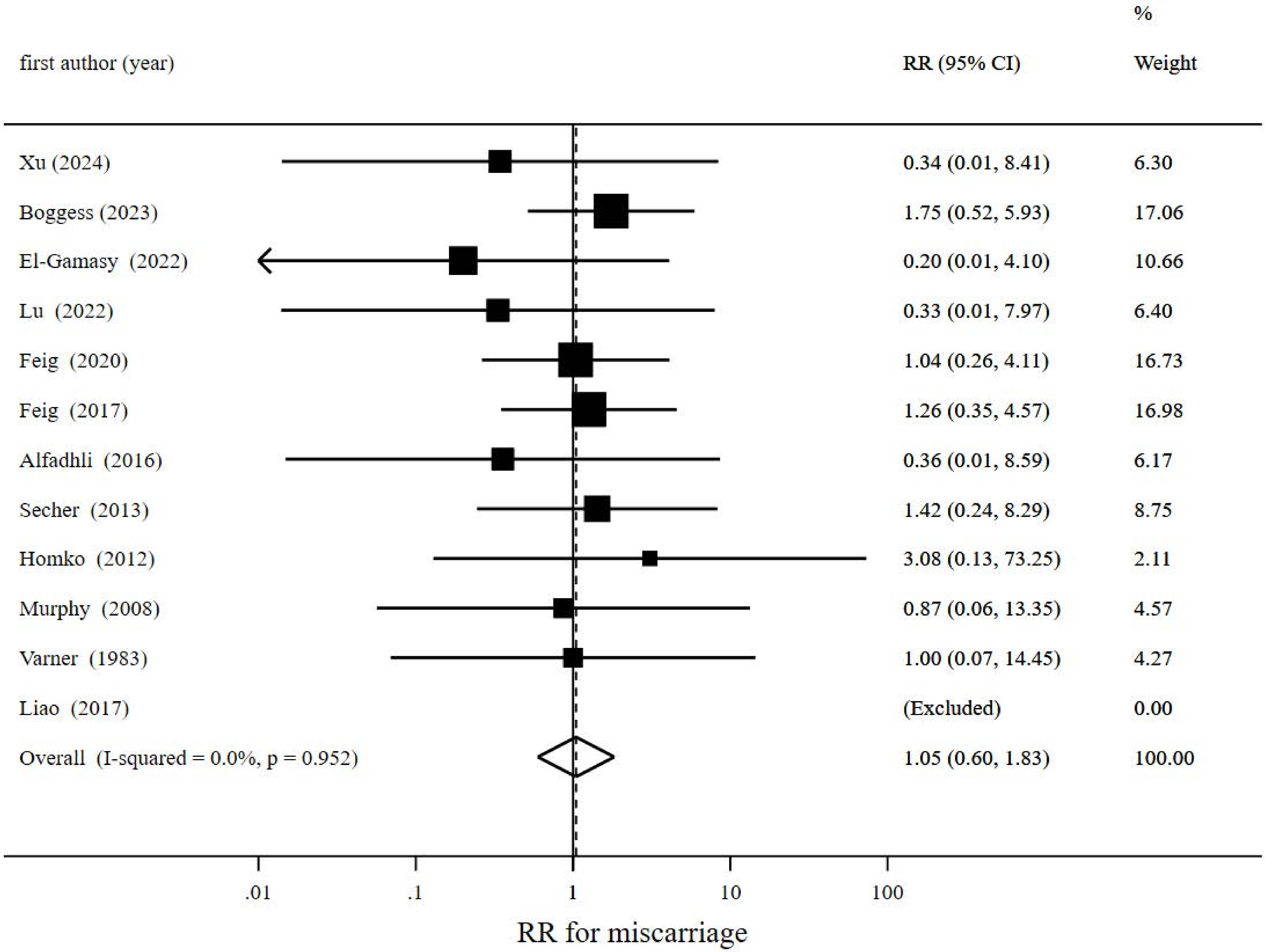

%
first author (year)
RR (95% CI)
Weight
Xu (2024)
0.34 (0.01, 8.41)
6.30
Boggess (2023)
1.75 (0.52, 5.93)
17.06
El-Gamasy (2022)
0.20 (0.01, 4.10)
10.66
Lu (2022)
0.33 (0.01, 7.97)
6.40
Feig (2020)
1.04 (0.26, 4.11)
16.73
Feig (2017)
1.26 (0.35, 4.57)
16.98
Alfadhli (2016)
0.36 (0.01, 8.59)
6.17
Secher (2013)
1.42 (0.24, 8.29)
8.75
Homko (2012)
3.08 (0.13, 73.25)
2.11
Murphy (2008)
0.87 (0.06, 13.35)
4.57
Varner (1983)
1.00 (0.07, 14.45)
4.27
Liao (2017)
(Excluded)
0.00
Overall (I-squared = 0.0%, p = 0.952)
1.05 (0.60, 1.83)
100.00
.01
.1
1
10
100
RR for miscarriage

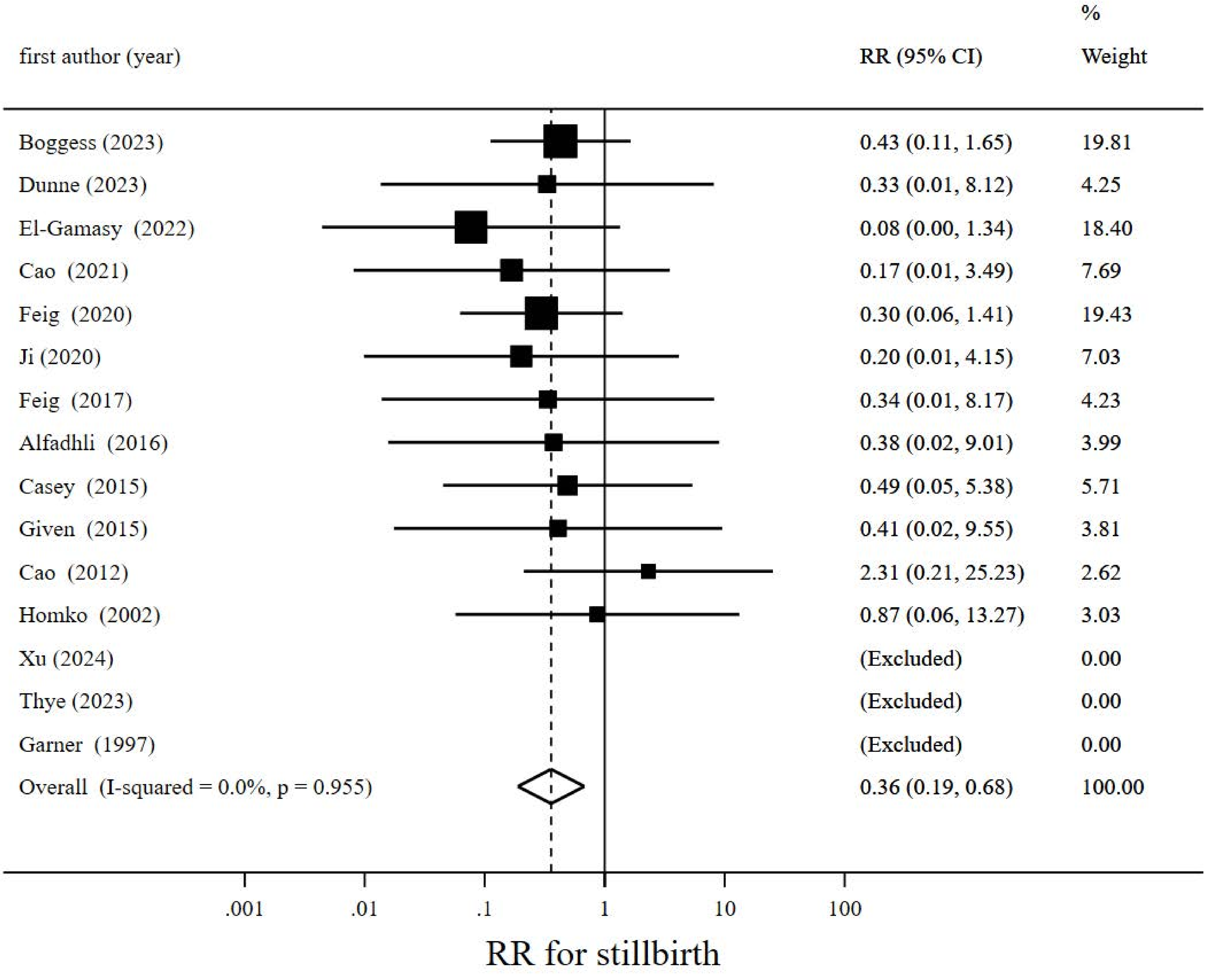
first author (year)
RR (95% CI)
% Weight
Boggess (2023)
0.43 (0.11, 1.65)
19.81
Dunne (2023)
0.33 (0.01, 8.12)
4.25
El-Gamasy (2022)
0.08 (0.00, 1.34)
18.40
Cao (2021)
0.17 (0.01, 3.49)
7.69
Feig (2020)
0.30 (0.06, 1.41)
19.43
Ji (2020)
0.20 (0.01, 4.15)
7.03
Feig (2017)
0.34 (0.01, 8.17)
4.23
Alfadhli (2016)
0.38 (0.02, 9.01)
3.99
Casey (2015)
0.49 (0.05, 5.38)
5.71
Given (2015)
0.41 (0.02, 9.55)
3.81
Cao (2012)
2.31 (0.21, 25.23)
2.62
Homko (2002)
0.87 (0.06, 13.27)
3.03
Xu (2024)
(Excluded)
0.00
Thye (2023)
(Excluded)
0.00
Garner (1997)
(Excluded)
0.00
Overall (I-squared = 0.0%, p = 0.955)
0.36 (0.19, 0.68)
100.00
.001
.01
.1
1
10
100
RR for stillbirth

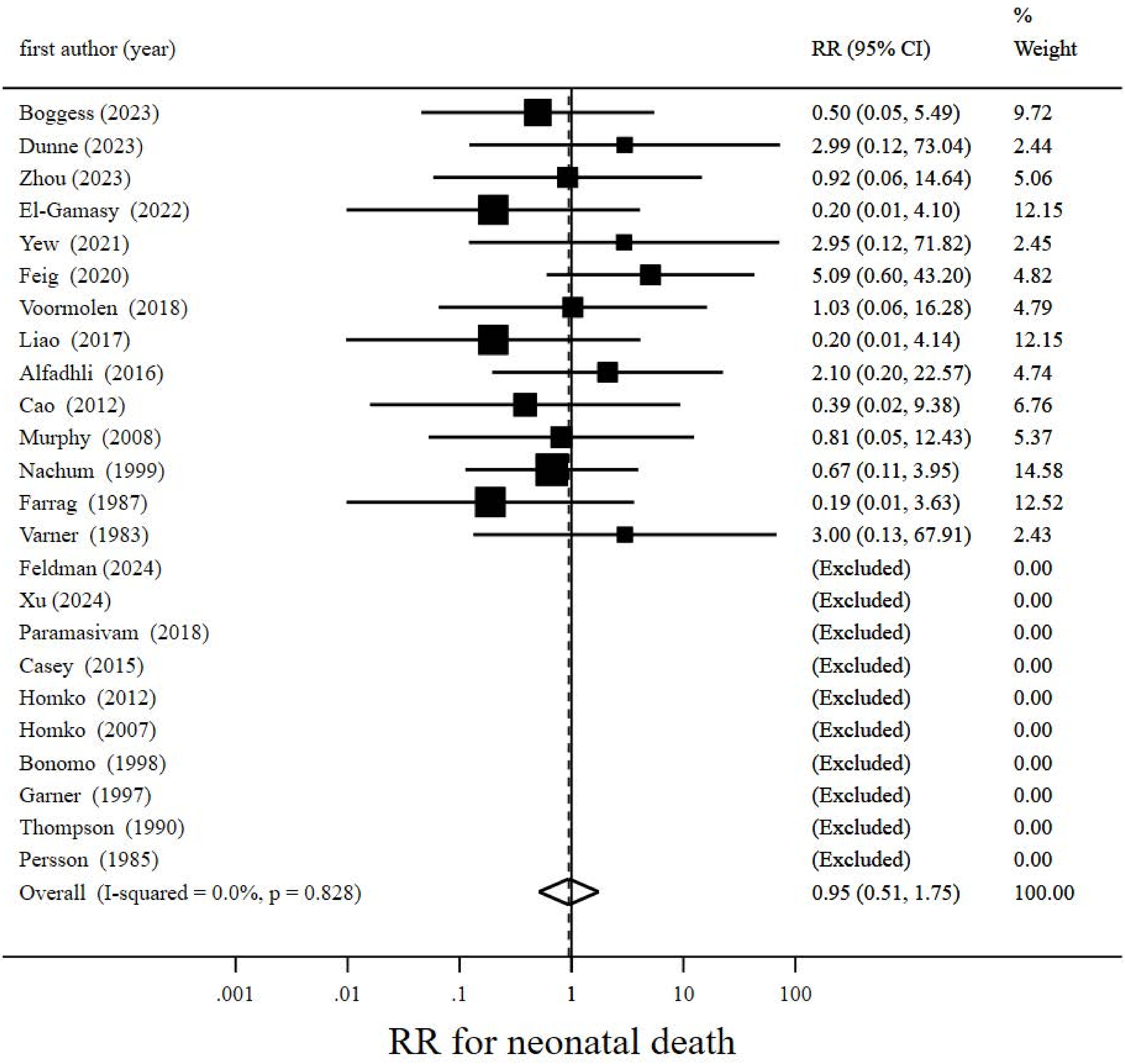

first author (year)
RR (95% CI)
% Weight
Boggess (2023) 0.50 (0.05, 5.49) 9.72
Dunne (2023) 2.99 (0.12, 73.04) 2.44
Zhou (2023) 0.92 (0.06, 14.64) 5.06
El-Gamasy (2022) 0.20 (0.01, 4.10) 12.15
Yew (2021) 2.95 (0.12, 71.82) 2.45
Feig (2020) 5.09 (0.60, 43.20) 4.82
Voormolen (2018) 1.03 (0.06, 16.28) 4.79
Liao (2017) 0.20 (0.01, 4.14) 12.15
Alfadhli (2016) 2.10 (0.20, 22.57) 4.74
Cao (2012) 0.39 (0.02, 9.38) 6.76
Murphy (2008) 0.81 (0.05, 12.43) 5.37
Nachum (1999) 0.67 (0.11, 3.95) 14.58
Farrag (1987) 0.19 (0.01, 3.63) 12.52
Varner (1983) 3.00 (0.13, 67.91) 2.43
Feldman (2024) (Excluded) 0.00
Xu (2024) (Excluded) 0.00
Paramasivam (2018) (Excluded) 0.00
Casey (2015) (Excluded) 0.00
Homko (2012) (Excluded) 0.00
Homko (2007) (Excluded) 0.00
Bonomo (1998) (Excluded) 0.00
Garner (1997) (Excluded) 0.00
Thompson (1990) (Excluded) 0.00
Persson (1985) (Excluded) 0.00
Overall (I-squared = 0.0%, p = 0.828) 0.95 (0.51, 1.75) 100.00
.001
.01
.1
1
10
100
RR for neonatal death

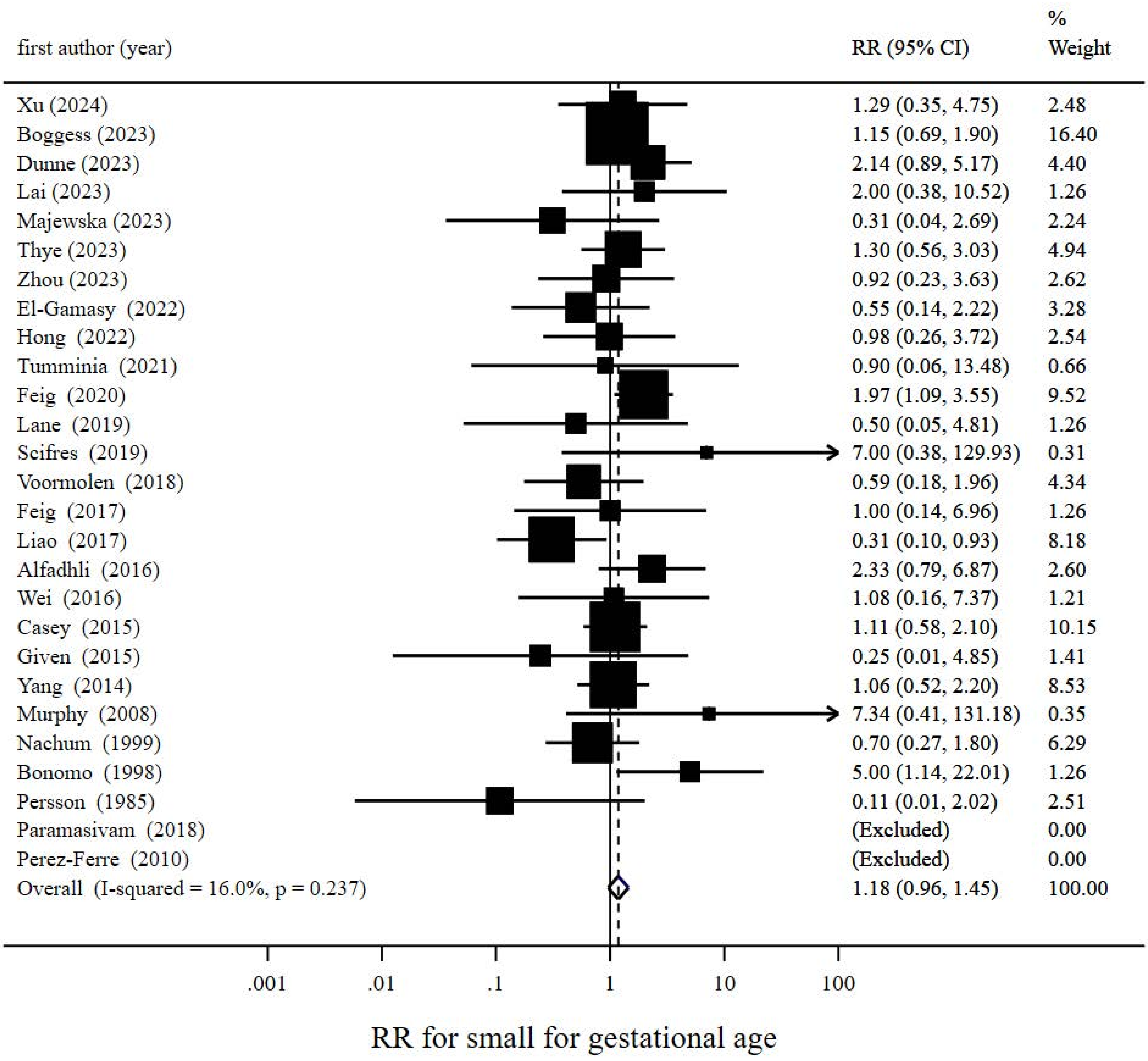
first author (year)
RR (95% CI)
% Weight
Xu (2024) 1.29 (0.35, 4.75) 2.48
Boggess (2023) 1.15 (0.69, 1.90) 16.40
Dunne (2023) 2.14 (0.89, 5.17) 4.40
Lai (2023) 2.00 (0.38, 10.52) 1.26
Majewska (2023) 0.31 (0.04, 2.69) 2.24
Thye (2023) 1.30 (0.56, 3.03) 4.94
Zhou (2023) 0.92 (0.23, 3.63) 2.62
El-Gamasy (2022) 0.55 (0.14, 2.22) 3.28
Hong (2022) 0.98 (0.26, 3.72) 2.54
Tumminia (2021) 0.90 (0.06, 13.48) 0.66
Feig (2020) 1.97 (1.09, 3.55) 9.52
Lane (2019) 0.50 (0.05, 4.81) 1.26
Scifres (2019) 7.00 (0.38, 129.93) 0.31
Voormolen (2018) 0.59 (0.18, 1.96) 4.34
Feig (2017) 1.00 (0.14, 6.96) 1.26
Liao (2017) 0.31 (0.10, 0.93) 8.18
Alfadhli (2016) 2.33 (0.79, 6.87) 2.60
Wei (2016) 1.08 (0.16, 7.37) 1.21
Casey (2015) 1.11 (0.58, 2.10) 10.15
Given (2015) 0.25 (0.01, 4.85) 1.41
Yang (2014) 1.06 (0.52, 2.20) 8.53
Murphy (2008) 7.34 (0.41, 131.18) 0.35
Nachum (1999) 0.70 (0.27, 1.80) 6.29
Bonomo (1998) 5.00 (1.14, 22.01) 1.26
Persson (1985) 0.11 (0.01, 2.02) 2.51
Paramasivam (2018) (Excluded) 0.00
Perez-Ferre (2010) (Excluded) 0.00
Overall (I-squared = 16.0%, p = 0.237) 1.18 (0.96, 1.45) 100.00
.001
.01
.1
1
10
100
RR for small for gestational age

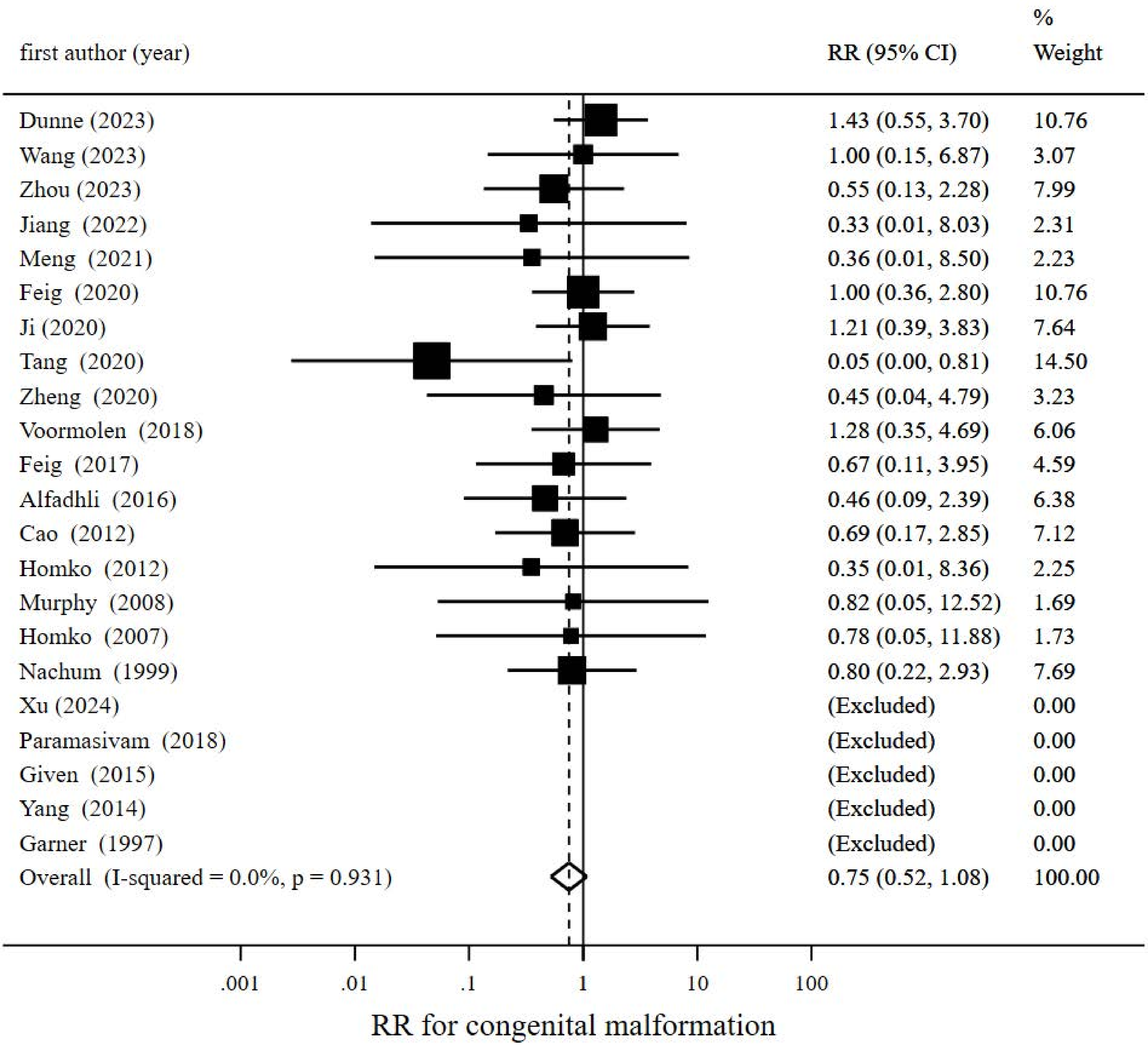
first author (year)
RR (95% CI)
% Weight
Dunne (2023) 1.43 (0.55, 3.70) 10.76
Wang (2023) 1.00 (0.15, 6.87) 3.07
Zhou (2023) 0.55 (0.13, 2.28) 7.99
Jiang (2022) 0.33 (0.01, 8.03) 2.31
Meng (2021) 0.36 (0.01, 8.50) 2.23
Feig (2020) 1.00 (0.36, 2.80) 10.76
Ji (2020) 1.21 (0.39, 3.83) 7.64
Tang (2020) 0.05 (0.00, 0.81) 14.50
Zheng (2020) 0.45 (0.04, 4.79) 3.23
Voormolen (2018) 1.28 (0.35, 4.69) 6.06
Feig (2017) 0.67 (0.11, 3.95) 4.59
Alfadhli (2016) 0.46 (0.09, 2.39) 6.38
Cao (2012) 0.69 (0.17, 2.85) 7.12
Homko (2012) 0.35 (0.01, 8.36) 2.25
Murphy (2008) 0.82 (0.05, 12.52) 1.69
Homko (2007) 0.78 (0.05, 11.88) 1.73
Nachum (1999) 0.80 (0.22, 2.93) 7.69
Xu (2024) (Excluded) 0.00
Paramasivam (2018) (Excluded) 0.00
Given (2015) (Excluded) 0.00
Yang (2014) (Excluded) 0.00
Garner (1997) (Excluded) 0.00
Overall (I-squared = 0.0%, p = 0.931) 0.75 (0.52, 1.08) 100.00
.001
.01
.1
1
10
100
RR for congenital malformation

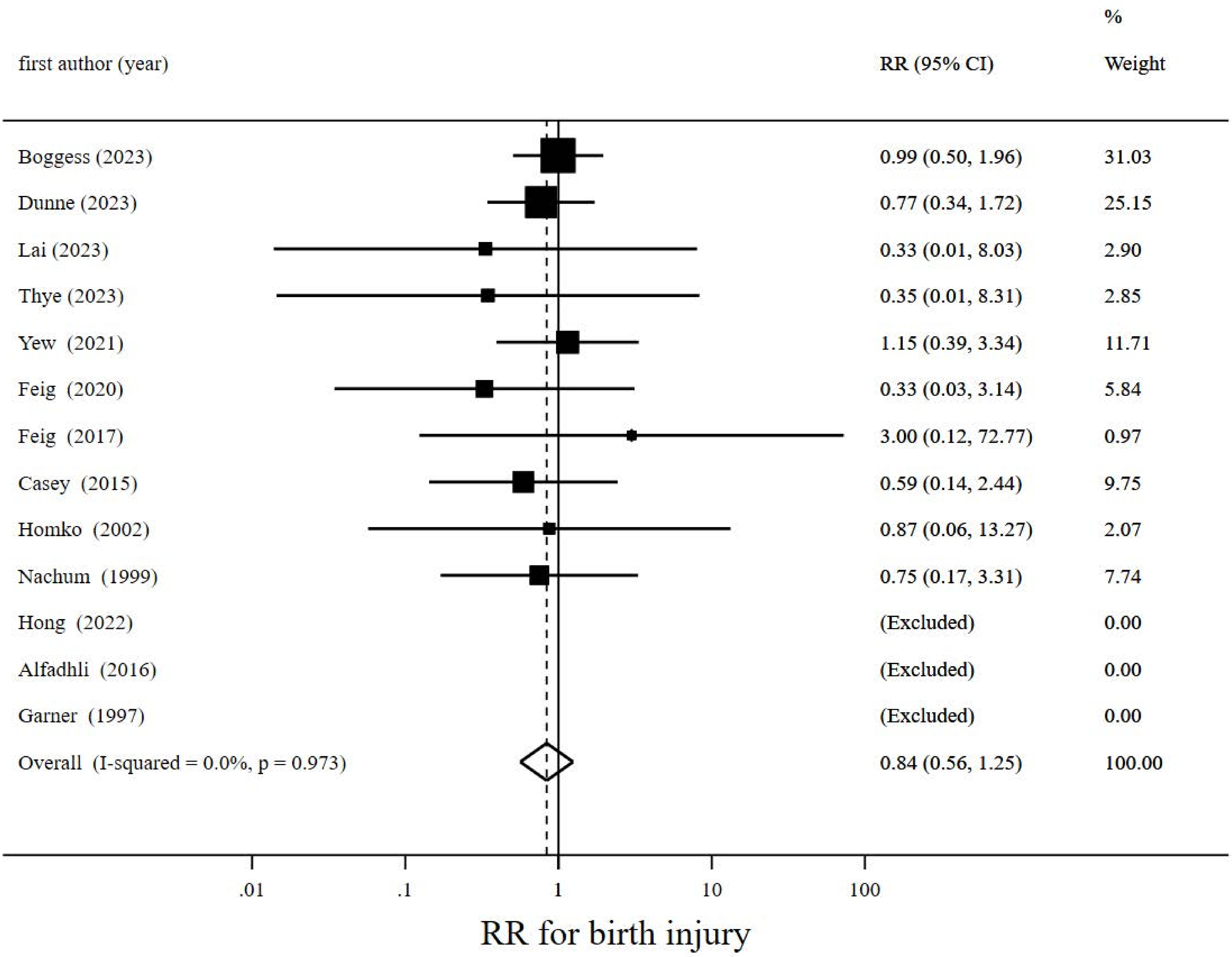

first author (year)
RR (95% CI)
% Weight
Boggess (2023) 0.99 (0.50, 1.96) 31.03
Dunne (2023) 0.77 (0.34, 1.72) 25.15
Lai (2023) 0.33 (0.01, 8.03) 2.90
Thye (2023) 0.35 (0.01, 8.31) 2.85
Yew (2021) 1.15 (0.39, 3.34) 11.71
Feig (2020) 0.33 (0.03, 3.14) 5.84
Feig (2017) 3.00 (0.12, 72.77) 0.97
Casey (2015) 0.59 (0.14, 2.44) 9.75
Homko (2002) 0.87 (0.06, 13.27) 2.07
Nachum (1999) 0.75 (0.17, 3.31) 7.74
Hong (2022) (Excluded) 0.00
Alfadhli (2016) (Excluded) 0.00
Garner (1997) (Excluded) 0.00
Overall (I-squared = 0.0%, p = 0.973) 0.84 (0.56, 1.25) 100.00
.01
.1
1
10
100
RR for birth injury

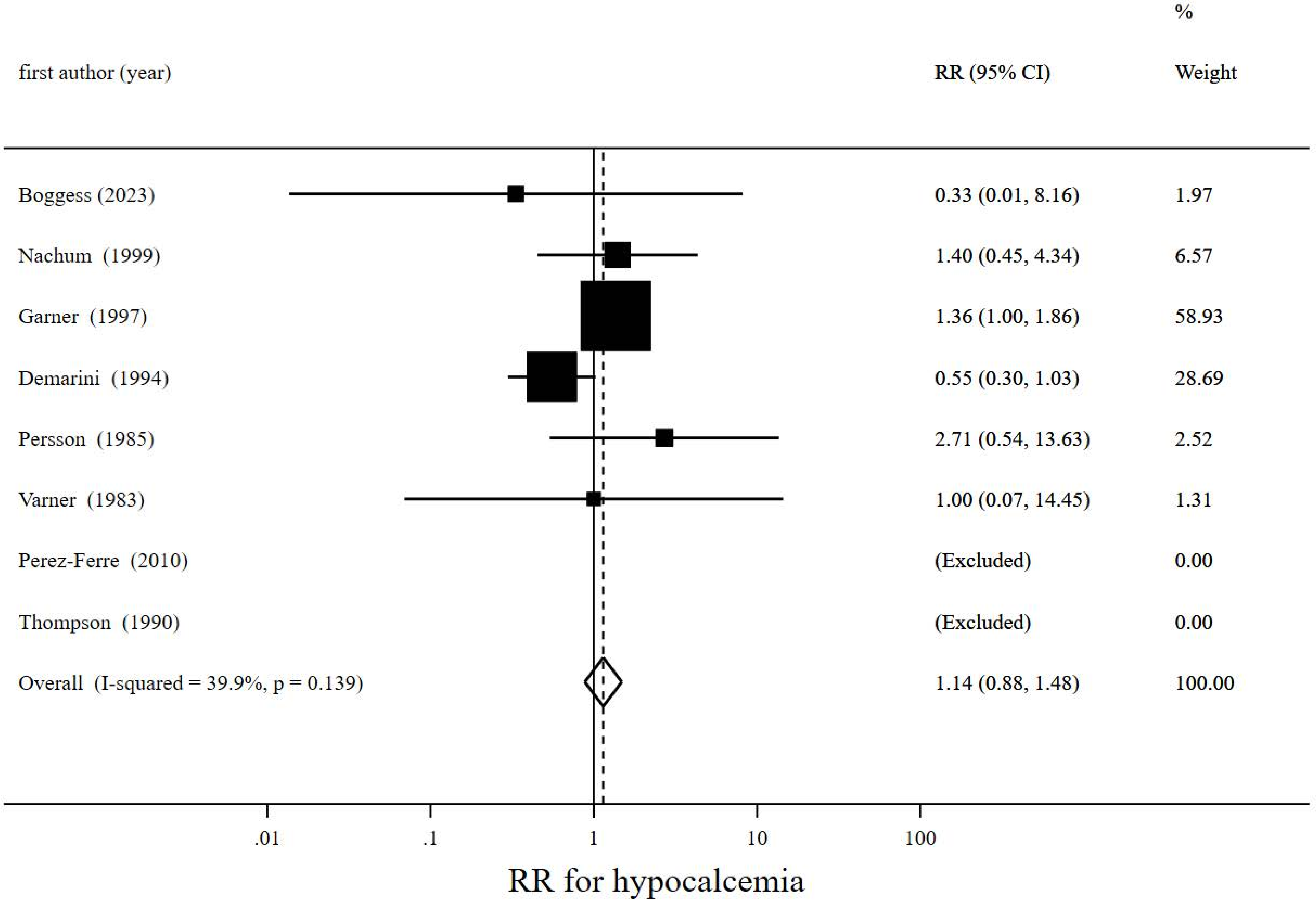
first author (year)
RR (95% CI)
%
Weight
Boggess (2023)
0.33 (0.01, 8.16)
1.97
Nachum (1999)
1.40 (0.45, 4.34)
6.57
Garner (1997)
1.36 (1.00, 1.86)
58.93
Demarini (1994)
0.55 (0.30, 1.03)
28.69
Persson (1985)
2.71 (0.54, 13.63)
2.52
Varner (1983)
1.00 (0.07, 14.45)
1.31
Perez-Ferre (2010)
(Excluded)
0.00
Thompson (1990)
(Excluded)
0.00
Overall (I-squared = 39.9%, p = 0.139)
1.14 (0.88, 1.48)
100.00
.01
.1
1
10
100
RR for hypocalcemia

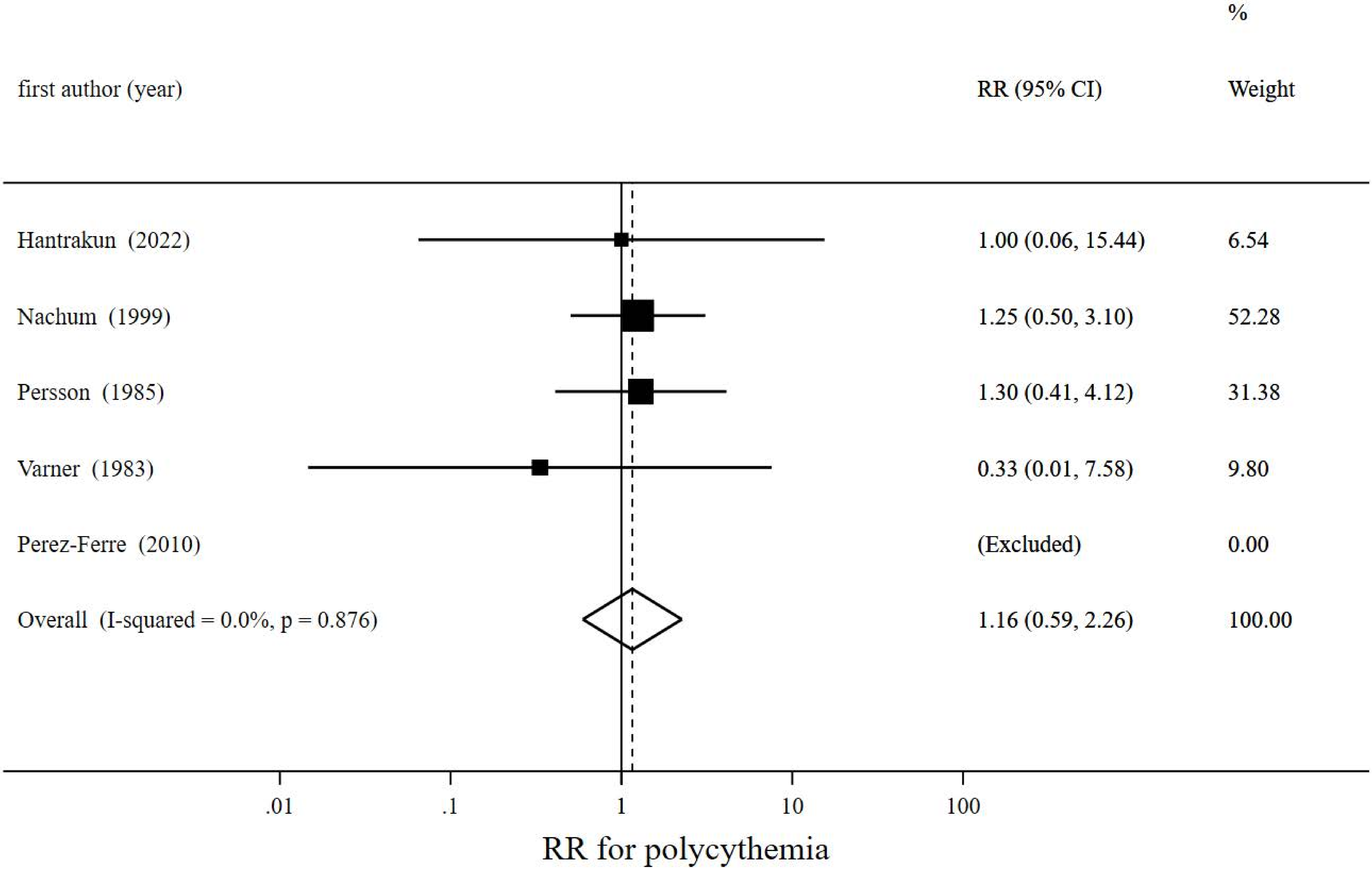
%
first author (year)
RR (95% CI)
Weight
Hantrakun (2022)
1.00 (0.06, 15.44)
6.54
Nachum (1999)
1.25 (0.50, 3.10)
52.28
Persson (1985)
1.30 (0.41, 4.12)
31.38
Varner (1983)
0.33 (0.01, 7.58)
9.80
Perez-Ferre (2010)
(Excluded)
0.00
Overall (I-squared = 0.0%, p = 0.876)
1.16 (0.59, 2.26)
100.00
.01
.1
1
10
100
RR for polycythemia

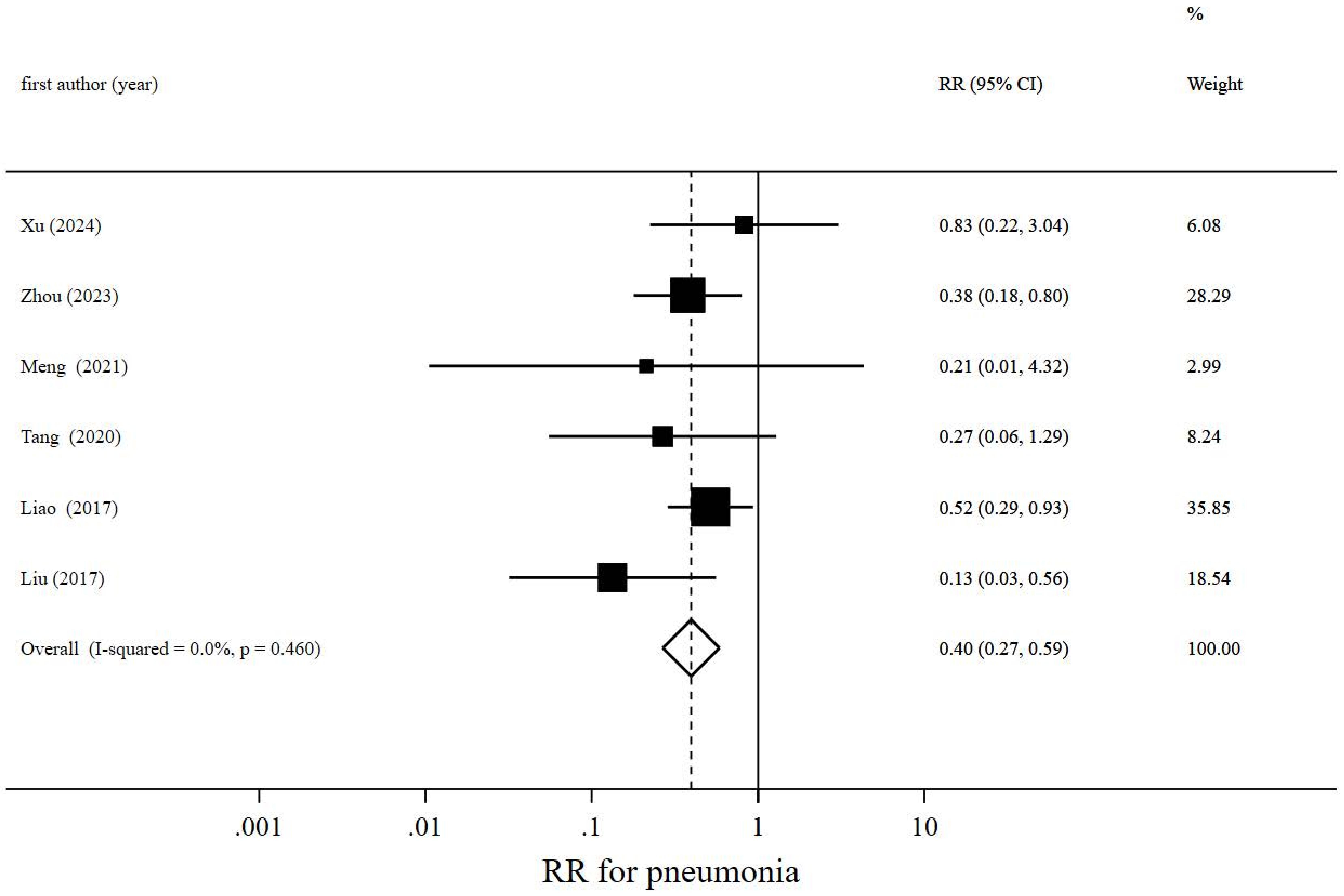
%
first author (year)
RR (95% CI)
Weight
Xu (2024)
0.83 (0.22, 3.04)
6.08
Zhou (2023)
0.38 (0.18, 0.80)
28.29
Meng (2021)
0.21 (0.01, 4.32)
2.99
Tang (2020)
0.27 (0.06, 1.29)
8.24
Liao (2017)
0.52 (0.29, 0.93)
35.85
Liu (2017)
0.13 (0.03, 0.56)
18.54
Overall (I-squared = 0.0%, p = 0.460)
0.40 (0.27, 0.59)
100.00
.001
.01
.1
1
10
RR for pneumonia

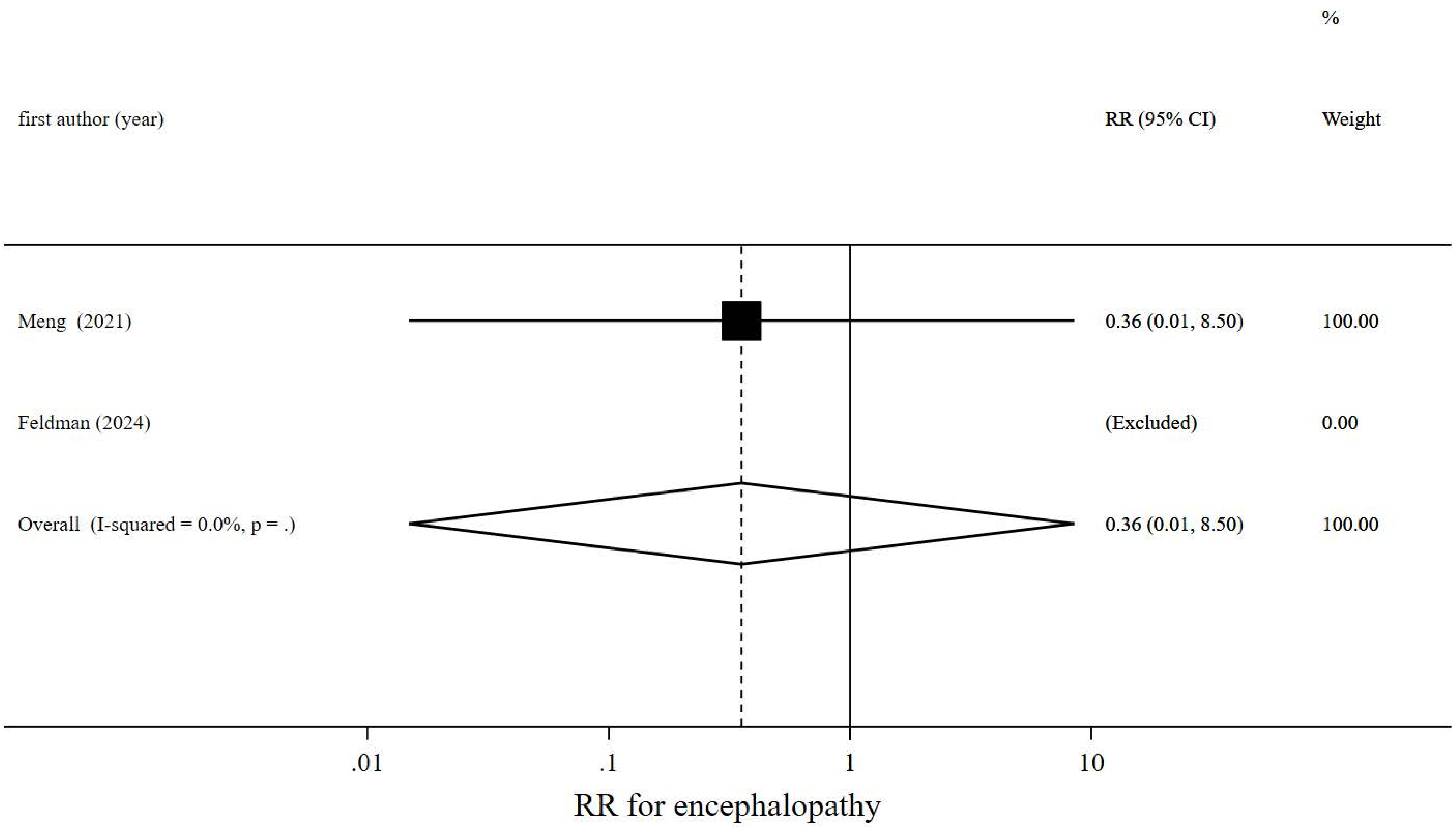
%
first author (year)
RR (95% CI)
Weight
Meng (2021)
0.36 (0.01, 8.50)
100.00
Feldman (2024)
(Excluded)
0.00
Overall (I-squared = 0.0%, p = .)
0.36 (0.01, 8.50)
100.00
.01
.1
1
10
RR for encephalopathy

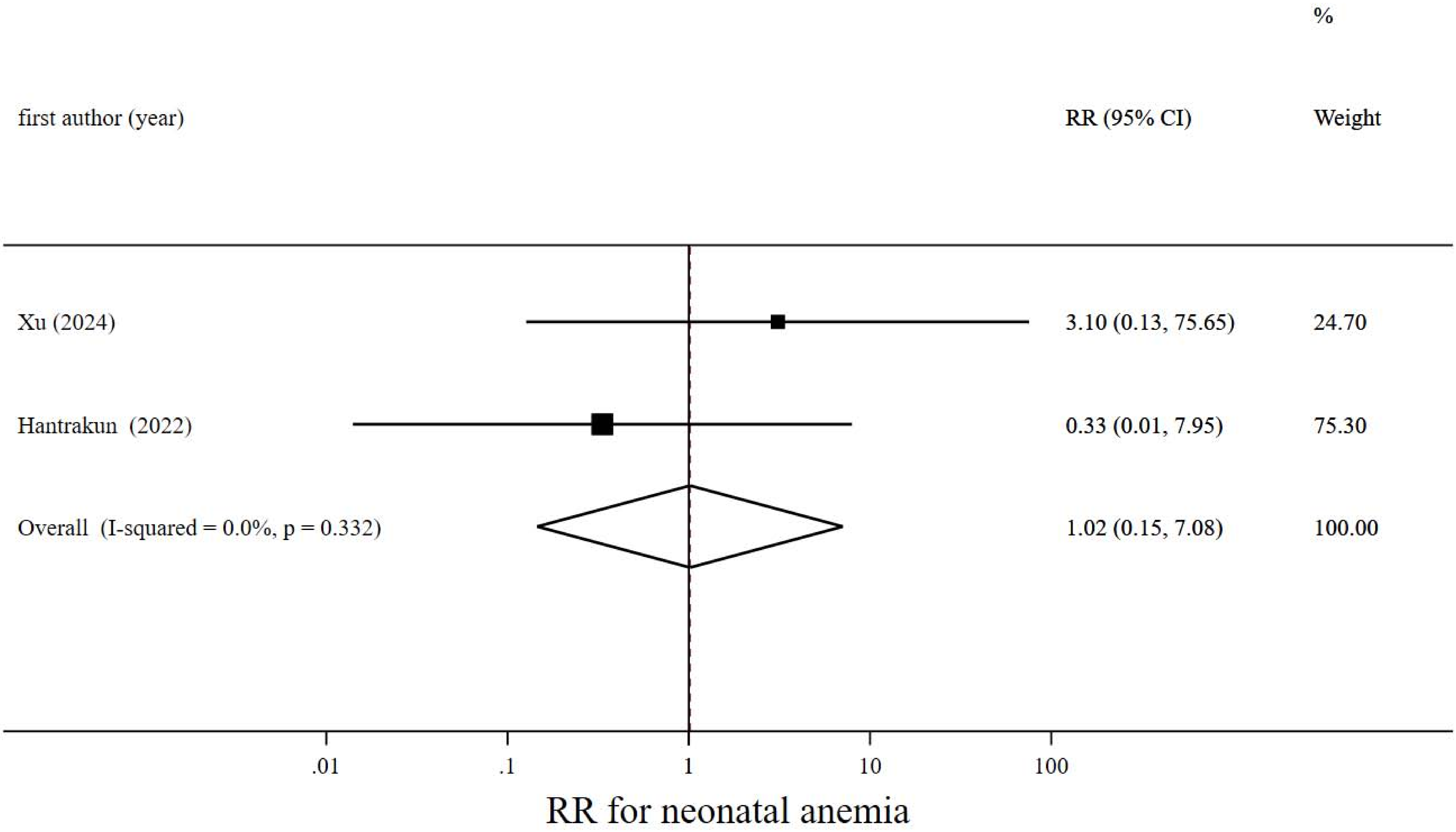
%
first author (year)
RR (95% CI)
Weight
Xu (2024)
3.10 (0.13, 75.65)
24.70
Hantrakun (2022)
0.33 (0.01, 7.95)
75.30
Overall (I-squared = 0.0%, p = 0.332)
1.02 (0.15, 7.08)
100.00
.01
.1
1
10
100
RR for neonatal anemia
