# Supplementary material for: Relationship Between Improvements in Glycemic Control and Risk of Pregnancy Complications in Patients With Diabetes Mellitus: Metaregression Analysis of Randomized Controlled Trials of Intensive Glucose Management
Source: J Diabetes Res. 2025 Jun 23;2025:3490884. doi: 10.1155/jdr/3490884 (PMC12208766; doi:10.1155/jdr/3490884)
Supplement: Supporting Information 2 — Funnel plots of the logarithm of relative risk (RR) and its corresponding standard error (se) for each adverse pregnancy outcome. Visually, asymmetry of the funnel plot suggests bias which was statistically detected (see Table 7). [file 3490884.f2.pdf]

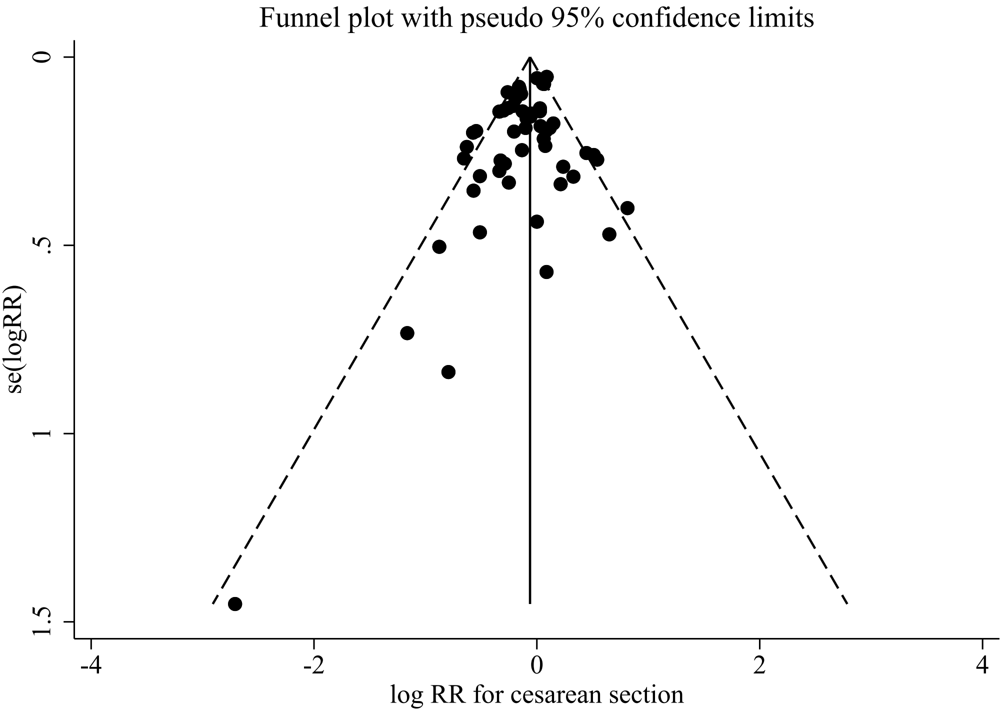

Funnel plot with pseudo 95% confidence limits
se(logRR)
0
.5
1
1.5
-4
-2
0
2
4
log RR for cesarean section

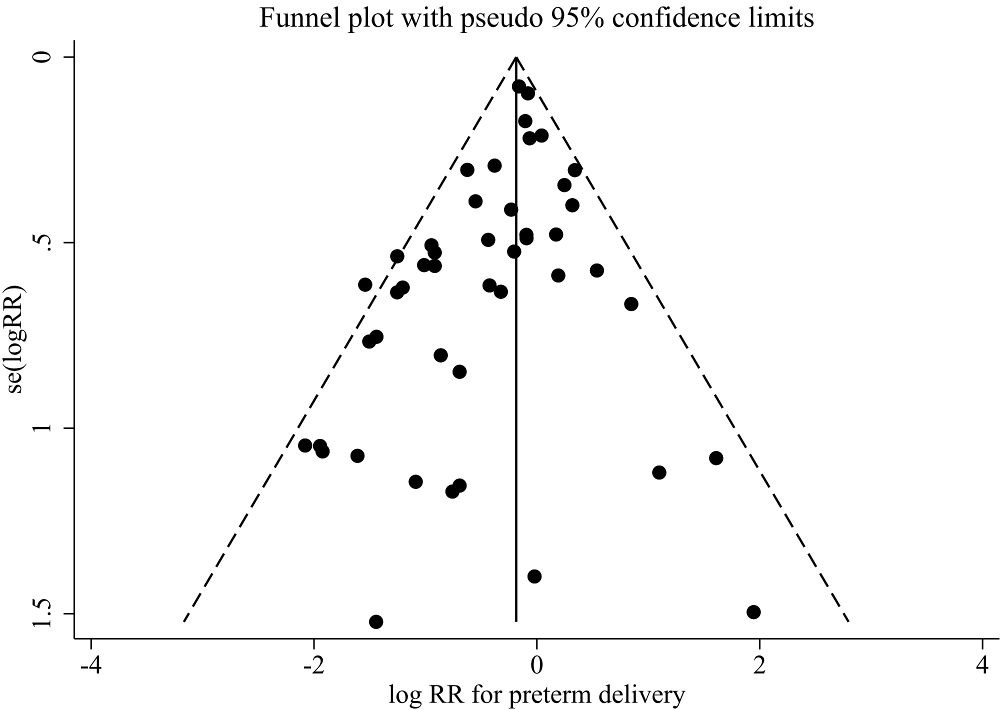
Funnel plot with pseudo 95% confidence limits
0
.5
1
1.5
se(logRR)
-4
-2
0
2
4
log RR for preterm delivery

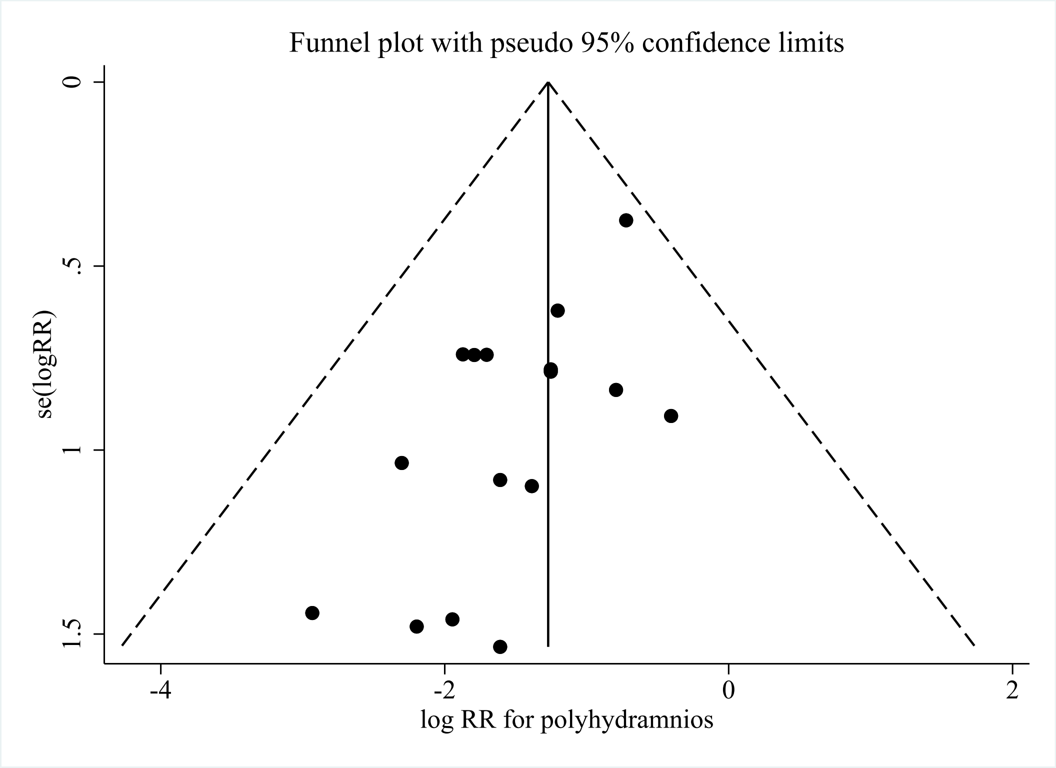
Funnel plot with pseudo 95% confidence limits
0
.5
1
1.5
se(logRR)
-4
-2
0
2
log RR for polyhydramnios

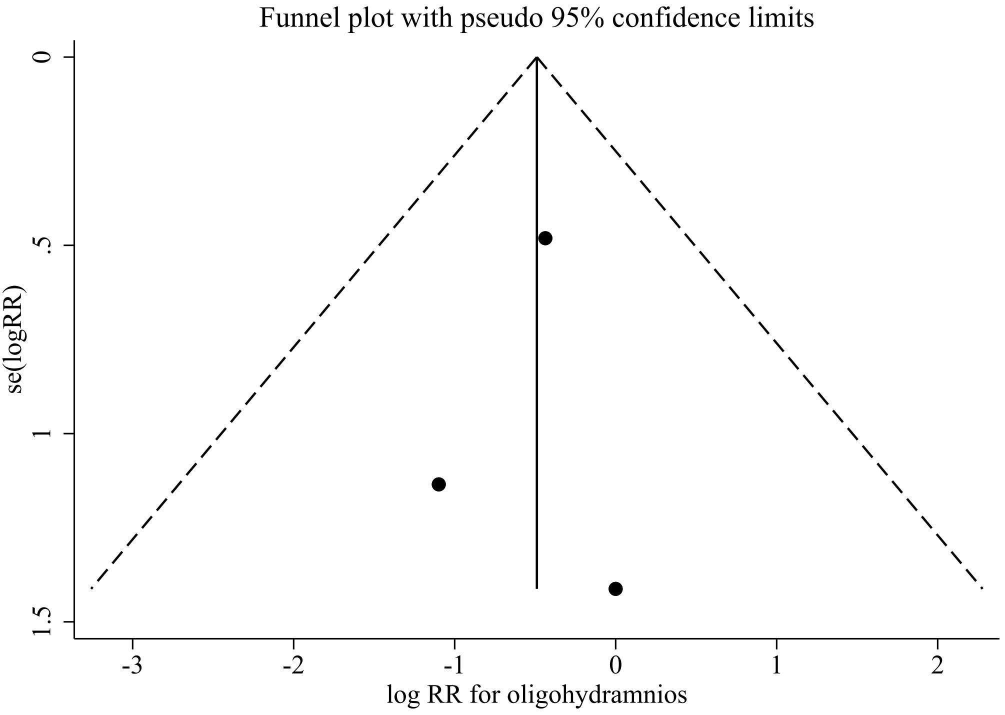
Funnel plot with pseudo 95% confidence limits
0
.5
1
1.5
se(logRR)
-3
-2
-1
0
1
2
log RR for oligohydramnios

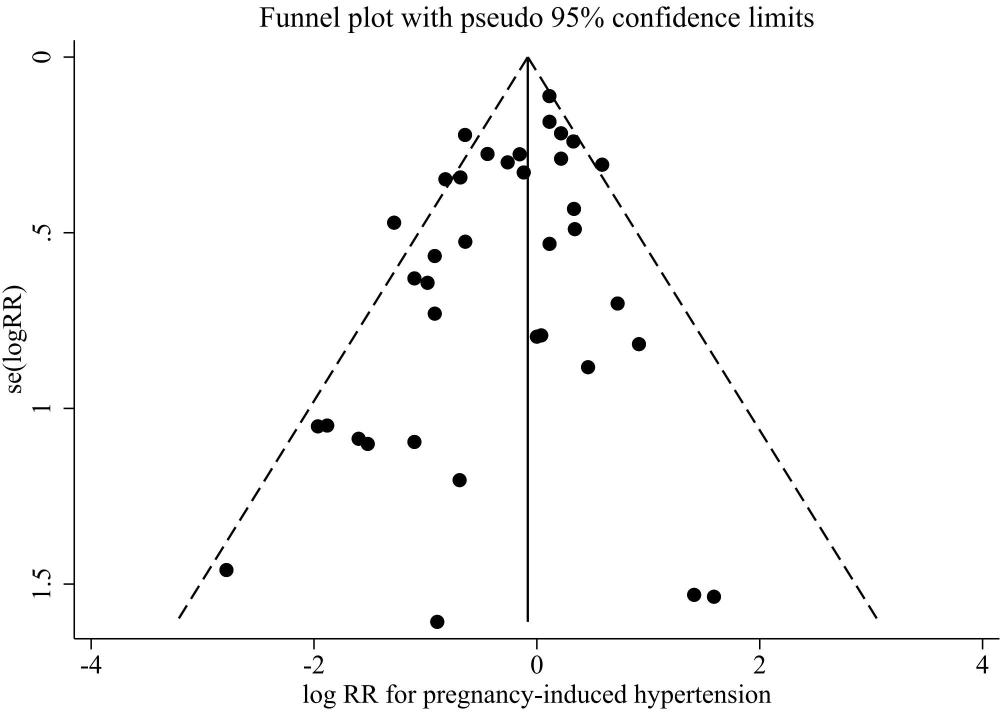
Funnel plot with pseudo 95% confidence limits
se(logRR)
0
.5
1
1.5
-4
-2
0
2
4
log RR for pregnancy-induced hypertension

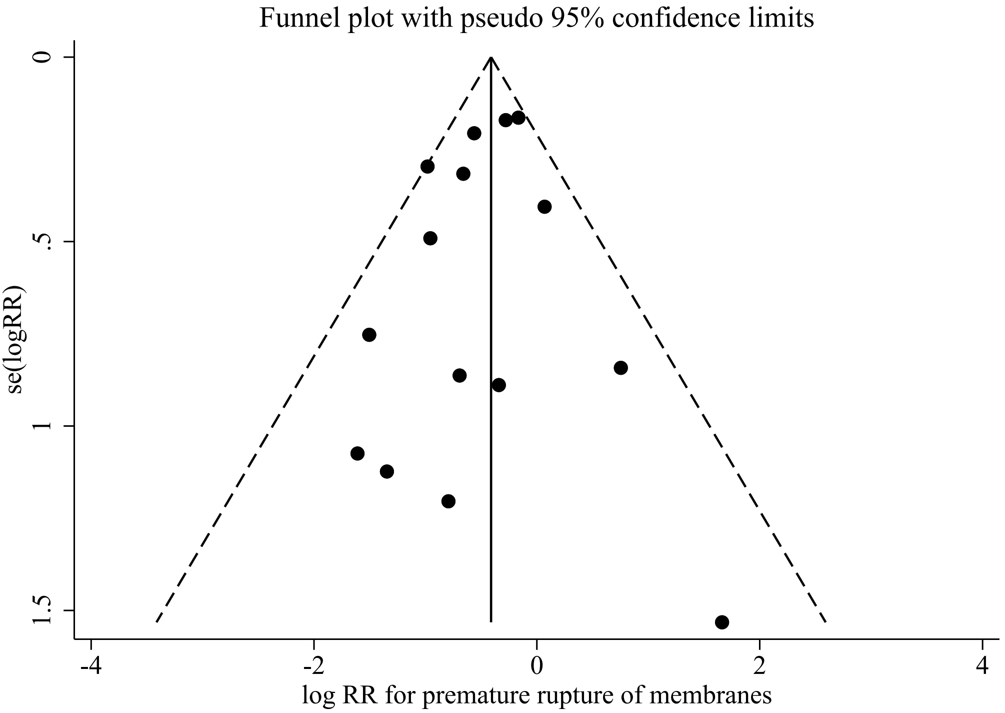
Funnel plot with pseudo 95% confidence limits
se(logRR)
0
.5
1
1.5
-4
-2
0
2
4
log RR for premature rupture of membranes

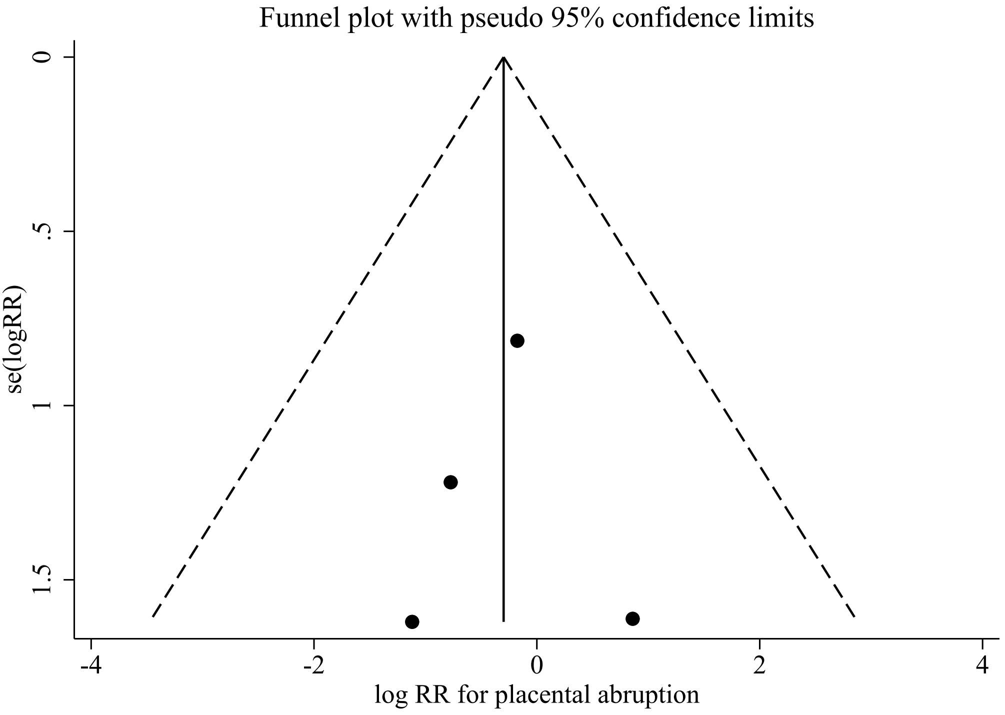

Funnel plot with pseudo 95% confidence limits
0
.5
1
1.5
se(logRR)
-4
-2
0
2
4
log RR for placental abruption

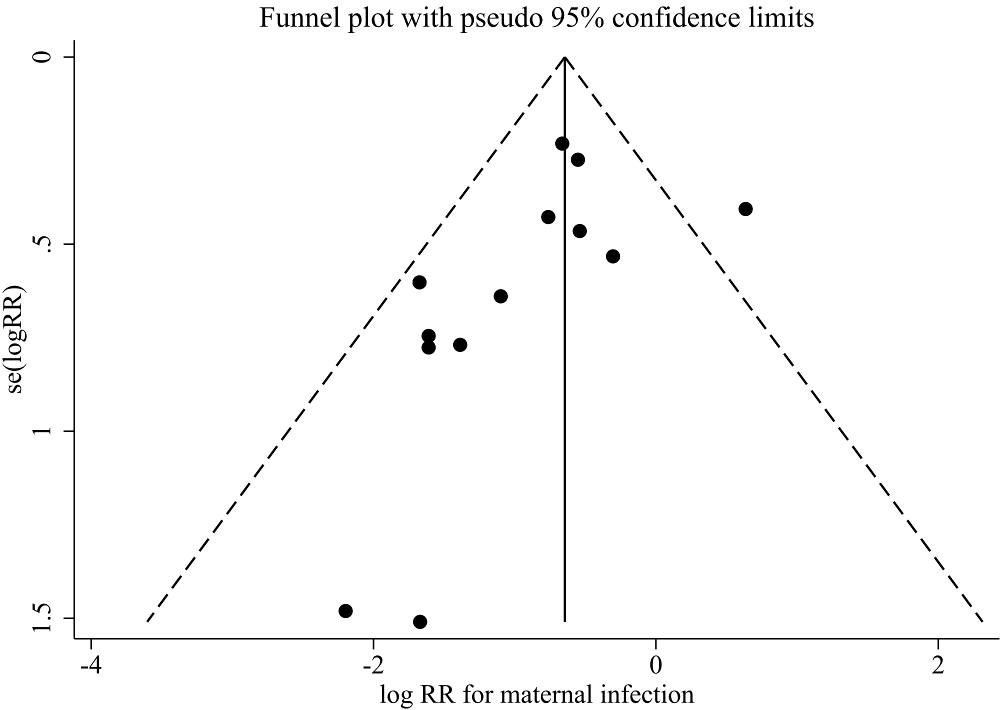
Funnel plot with pseudo 95% confidence limits
se(logRR)
0
.5
1
1.5
-4
-2
0
2
log RR for maternal infection

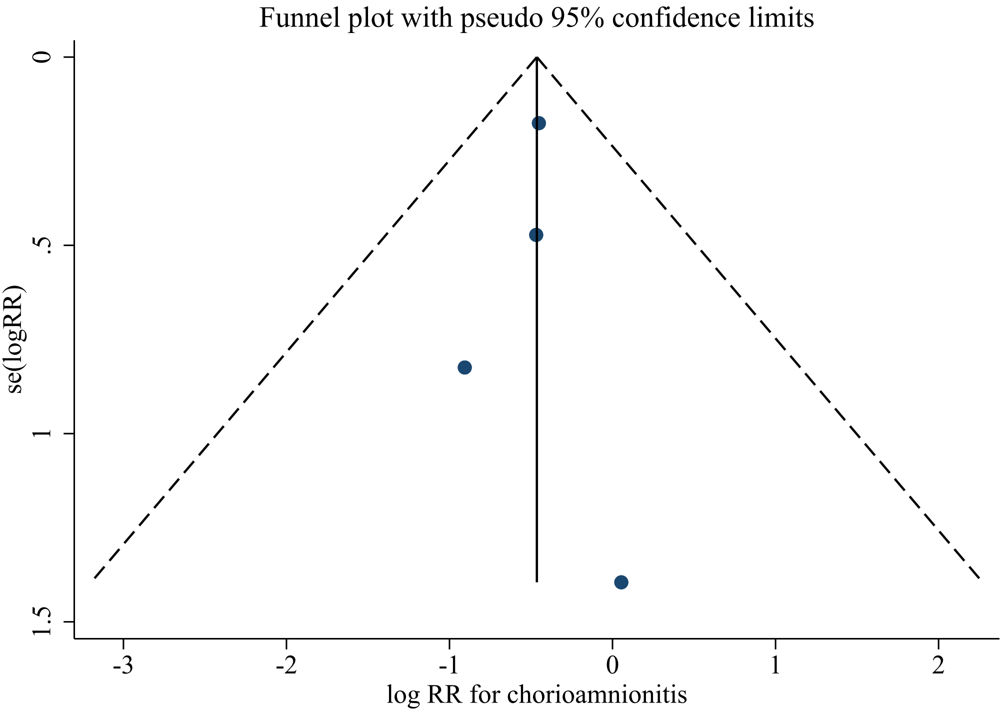

Funnel plot with pseudo 95% confidence limits
0
.5
1
1.5
se(logRR)
-3
-2
-1
0
1
2
log RR for chorioamnionitis

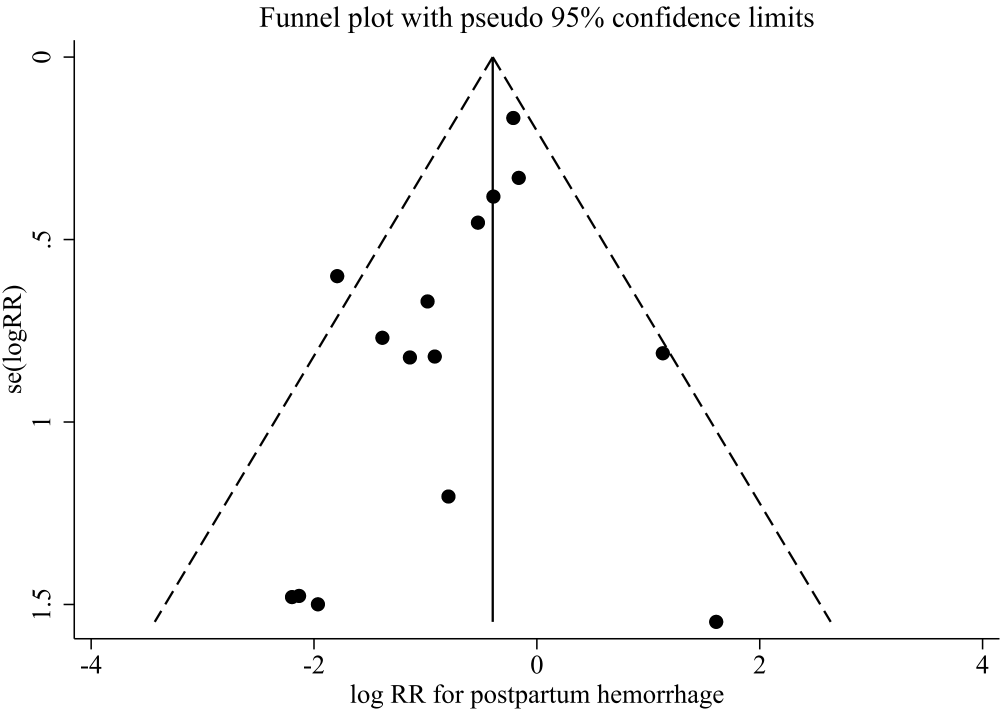
Funnel plot with pseudo 95% confidence limits
se(logRR)
0
.5
1
1.5
-4
-2
0
2
4
log RR for postpartum hemorrhage

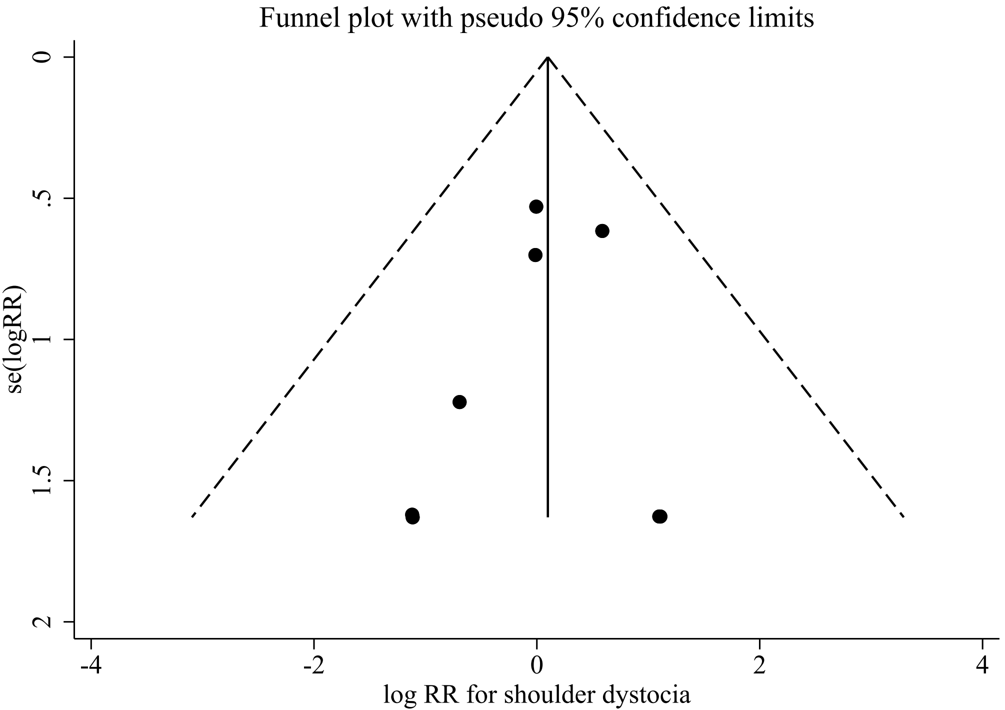

Funnel plot with pseudo 95% confidence limits
0
.5
1
1.5
2
se(logRR)
-4
-2
0
2
4
log RR for shoulder dystocia

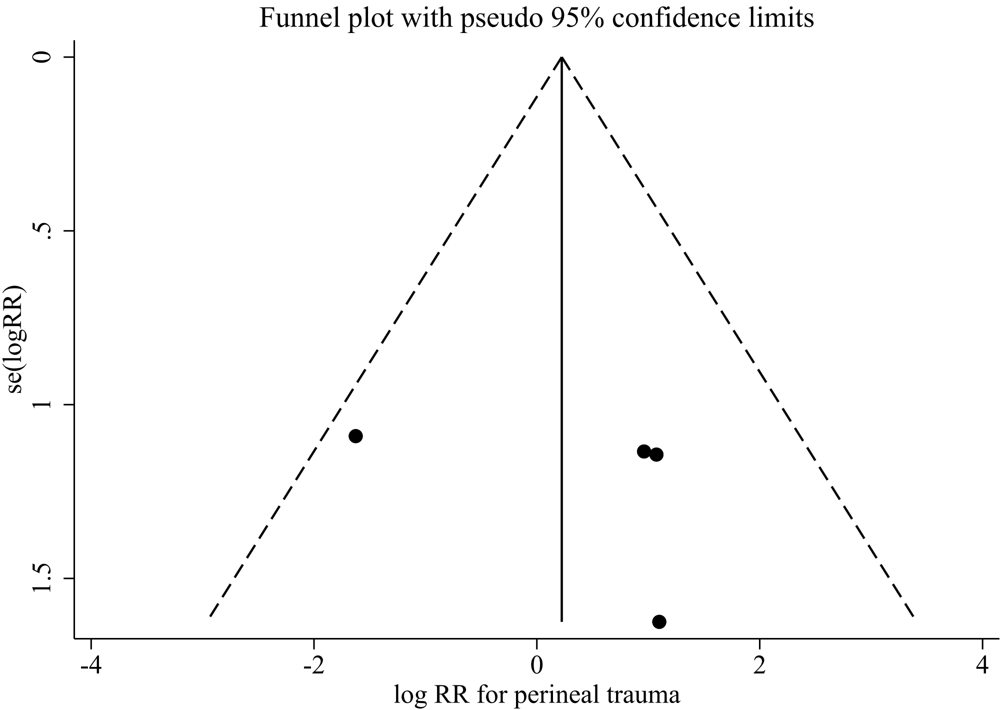

Funnel plot with pseudo 95% confidence limits
0
.5
1
1.5
se(logRR)
-4
-2
0
2
4
log RR for perineal trauma

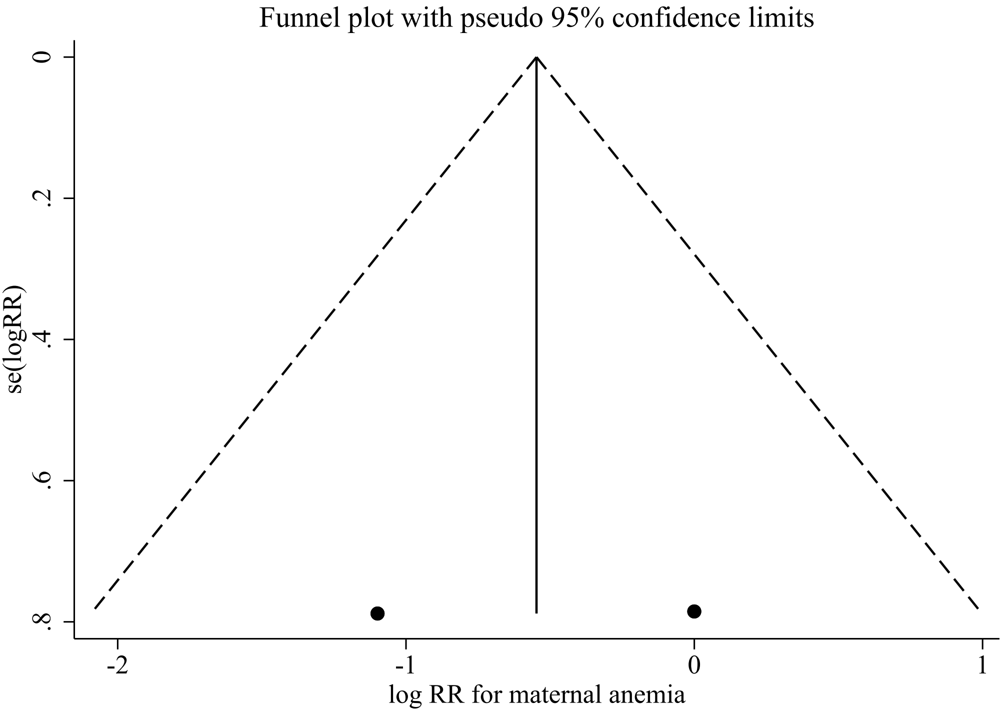
Funnel plot with pseudo 95% confidence limits
se(logRR)
0
.2
.4
.6
.8
-2
-1
0
1
log RR for maternal anemia

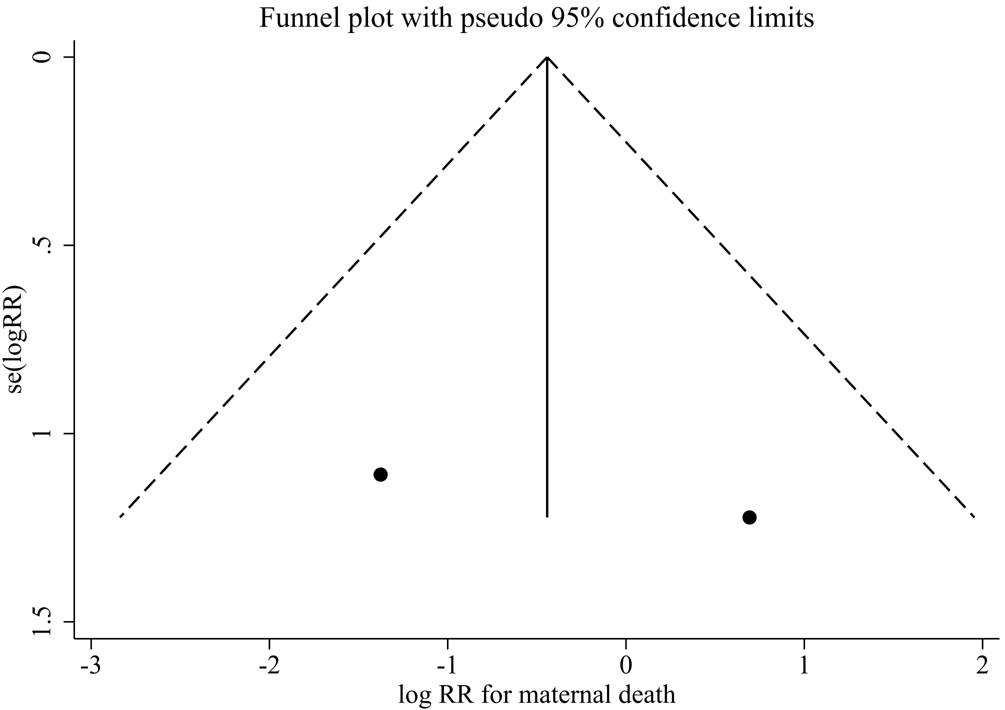

Funnel plot with pseudo 95% confidence limits
0
.5
1
1.5
se(logRR)
-3
-2
-1
0
1
2
log RR for maternal death

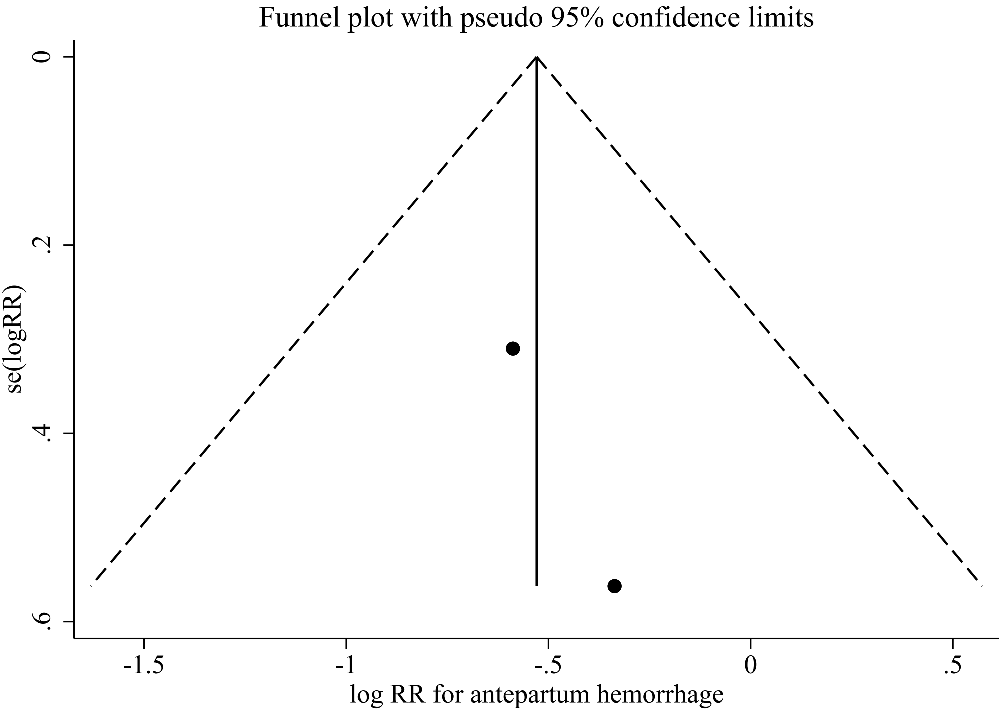

Funnel plot with pseudo 95% confidence limits
0
.2
.4
.6
se(logRR)
-1.5
-1
-.5
0
.5
log RR for antepartum hemorrhage

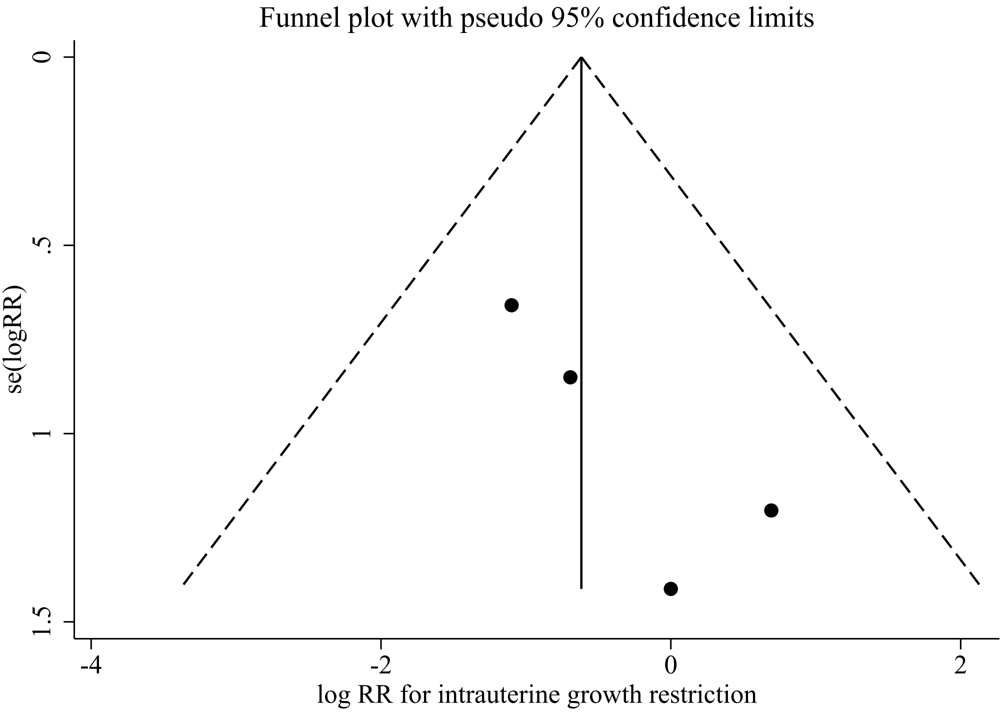

Funnel plot with pseudo 95% confidence limits
0
.5
1
1.5
se(logRR)
-4
-2
0
2
log RR for intrauterine growth restriction

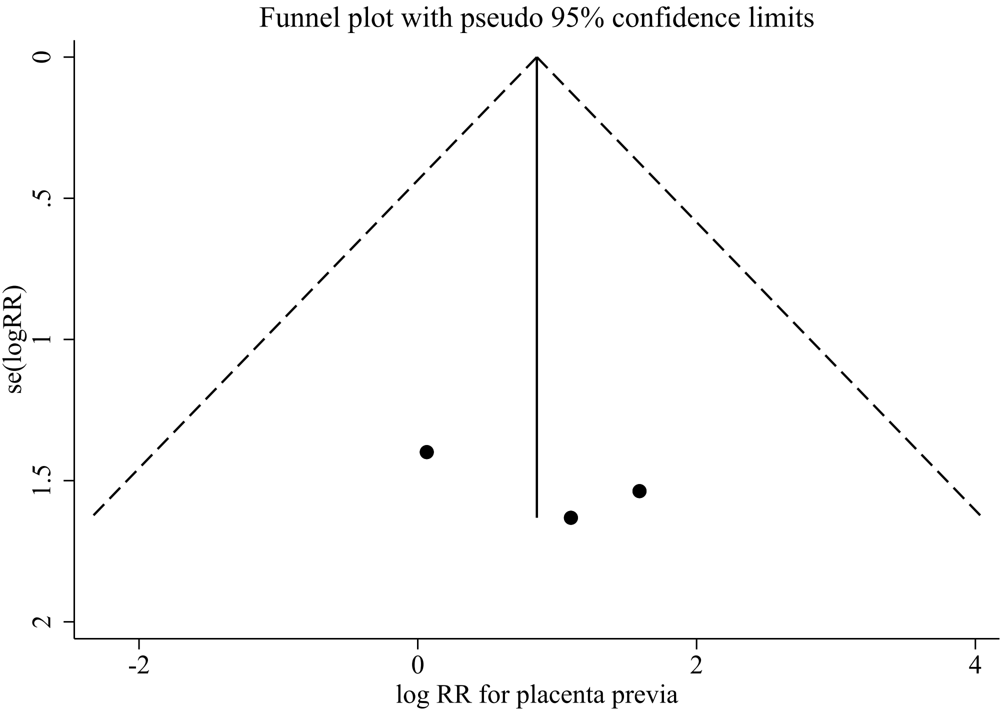
Funnel plot with pseudo 95% confidence limits
se(logRR)
0
.5
1
1.5
2
-2
0
2
4
log RR for placenta previa

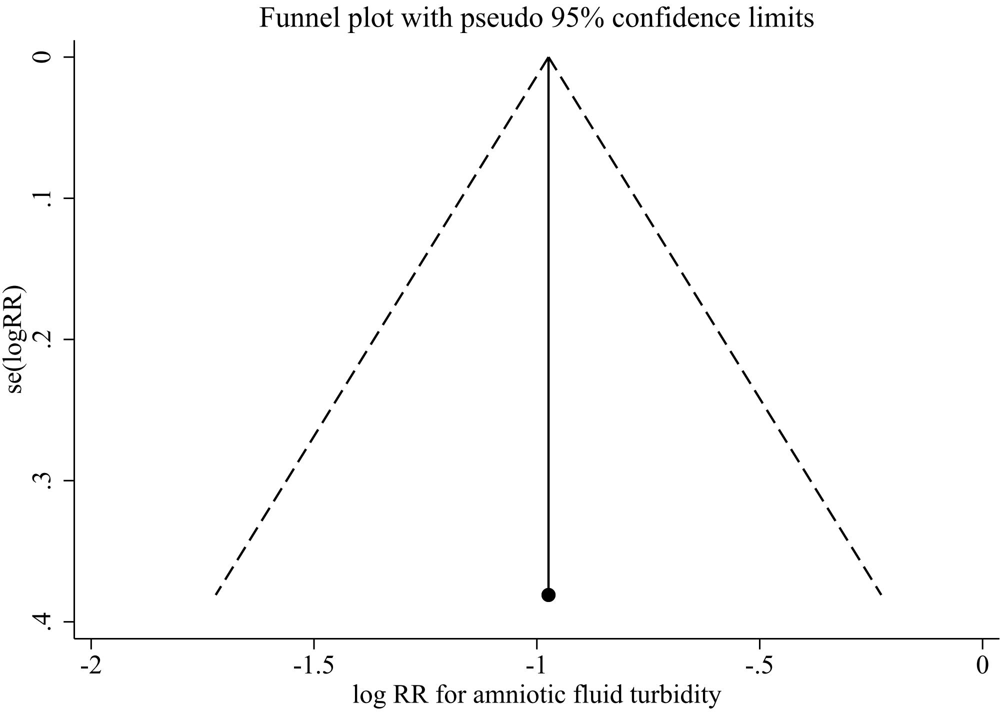

Funnel plot with pseudo 95% confidence limits
0
.1
.2
.3
.4
se(logRR)
-2
-1.5
-1
-.5
0
log RR for amniotic fluid turbidity

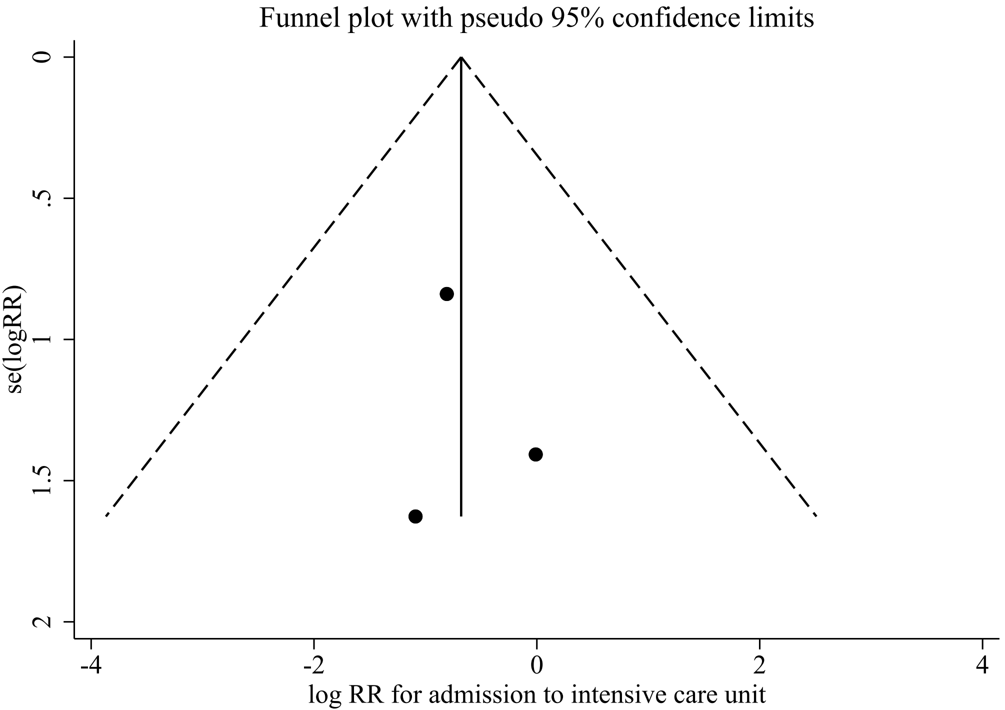
Funnel plot with pseudo 95% confidence limits
0
.5
1
1.5
2
se(logRR)
-4
-2
0
2
4
log RR for admission to intensive care unit

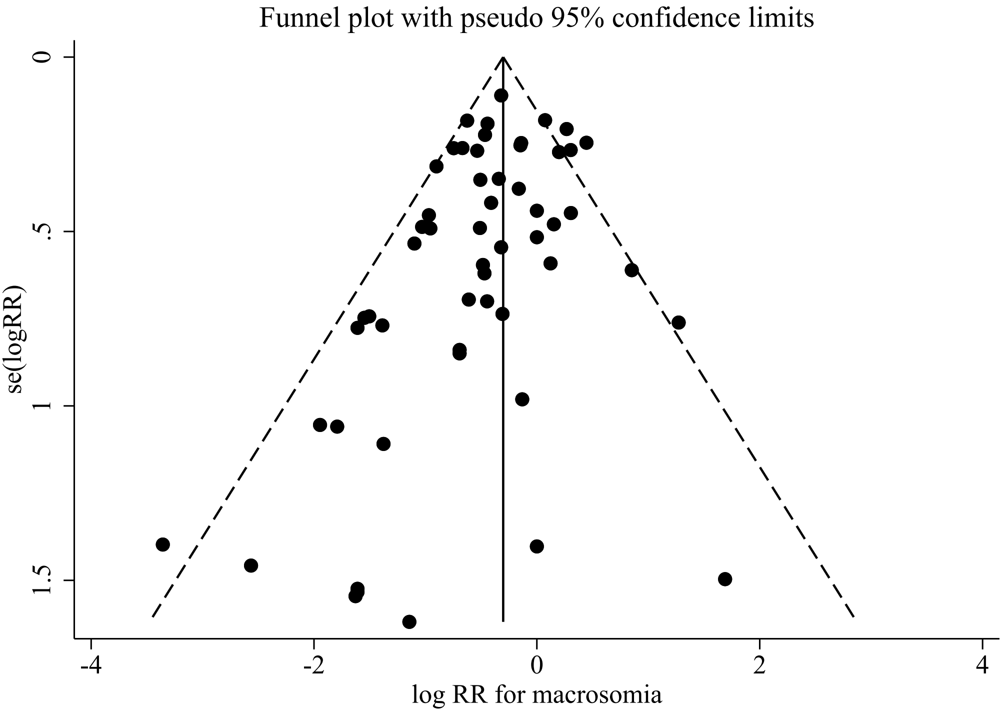

Funnel plot with pseudo 95% confidence limits
0
.5
1
1.5
se(logRR)
-4
-2
0
2
4
log RR for macrosomia

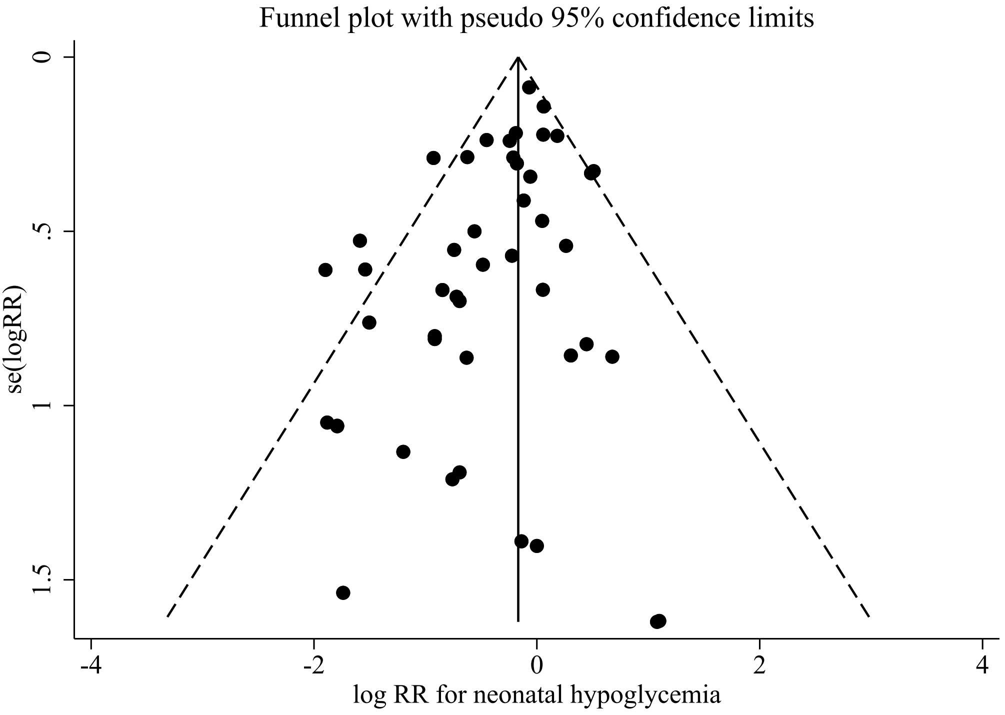

Funnel plot with pseudo 95% confidence limits
0
.5
1
1.5
se(logRR)
-4
-2
0
2
4
log RR for neonatal hypoglycemia

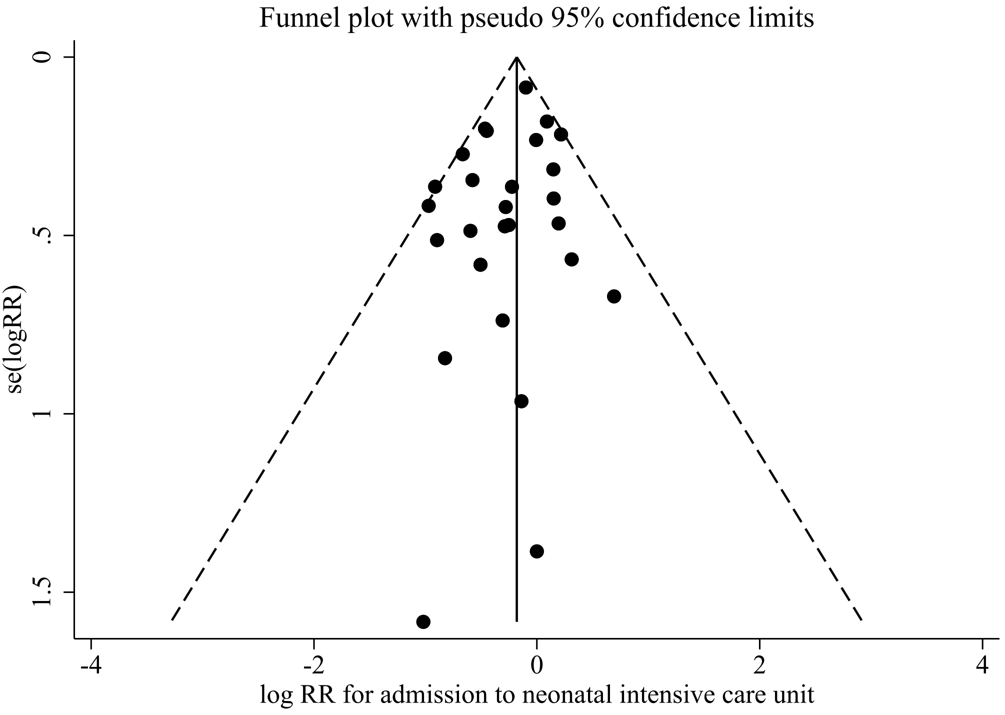
Funnel plot with pseudo 95% confidence limits
se(logRR)
0
.5
1
1.5
-4
-2
0
2
4
log RR for admission to neonatal intensive care unit

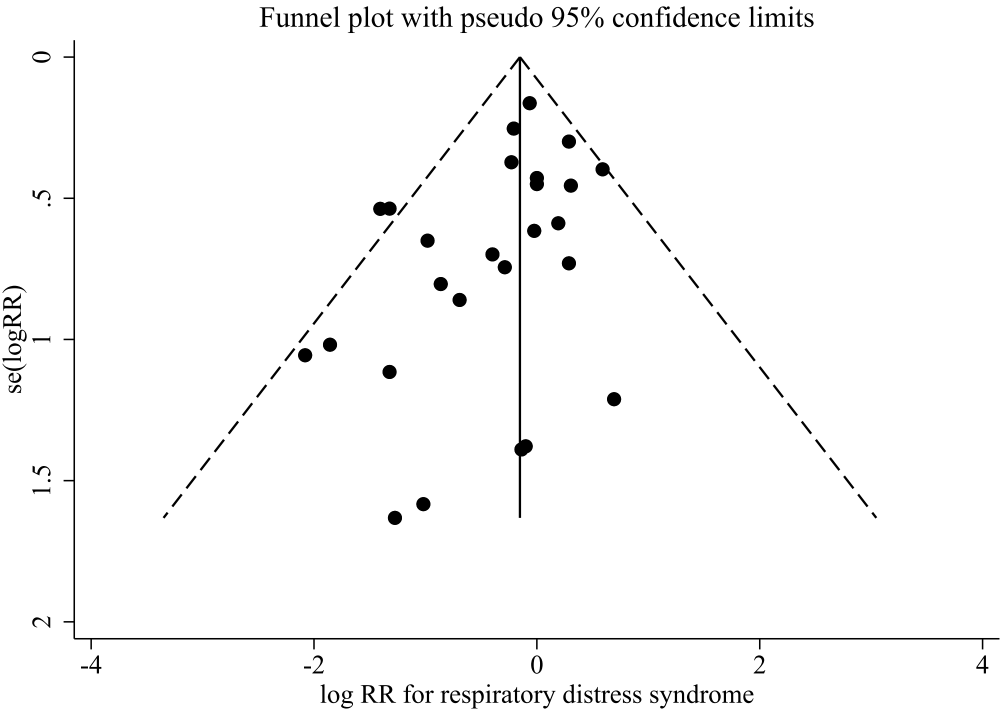
Funnel plot with pseudo 95% confidence limits
0
.5
1
1.5
2
se(logRR)
-4
-2
0
2
4
log RR for respiratory distress syndrome

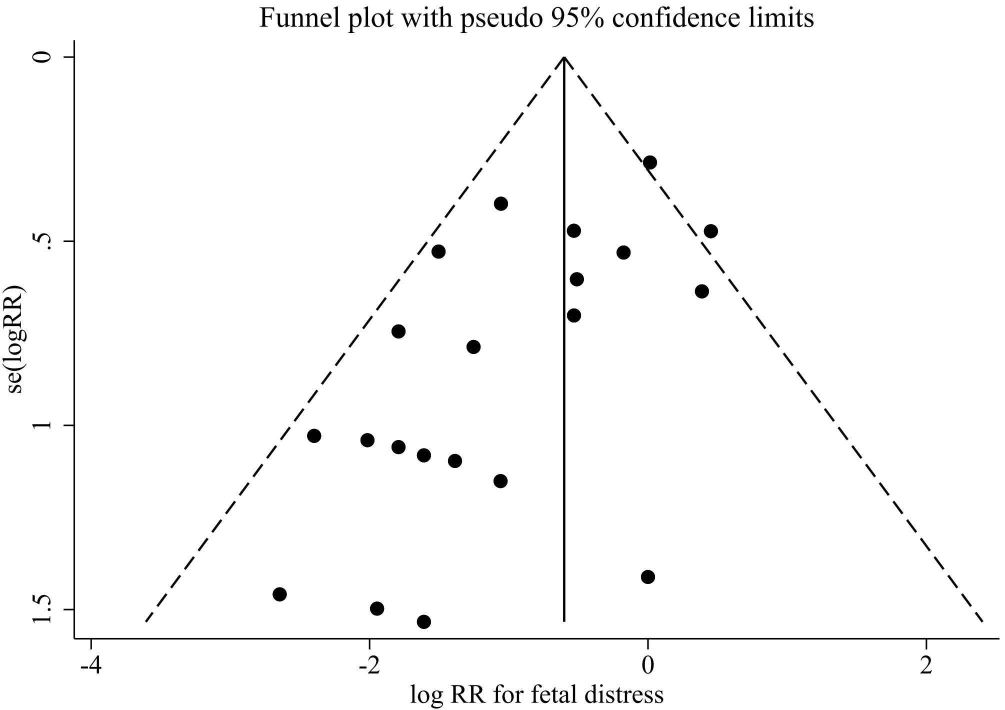
Funnel plot with pseudo 95% confidence limits
se(logRR)
0
.5
1
1.5
-4
-2
0
2
log RR for fetal distress

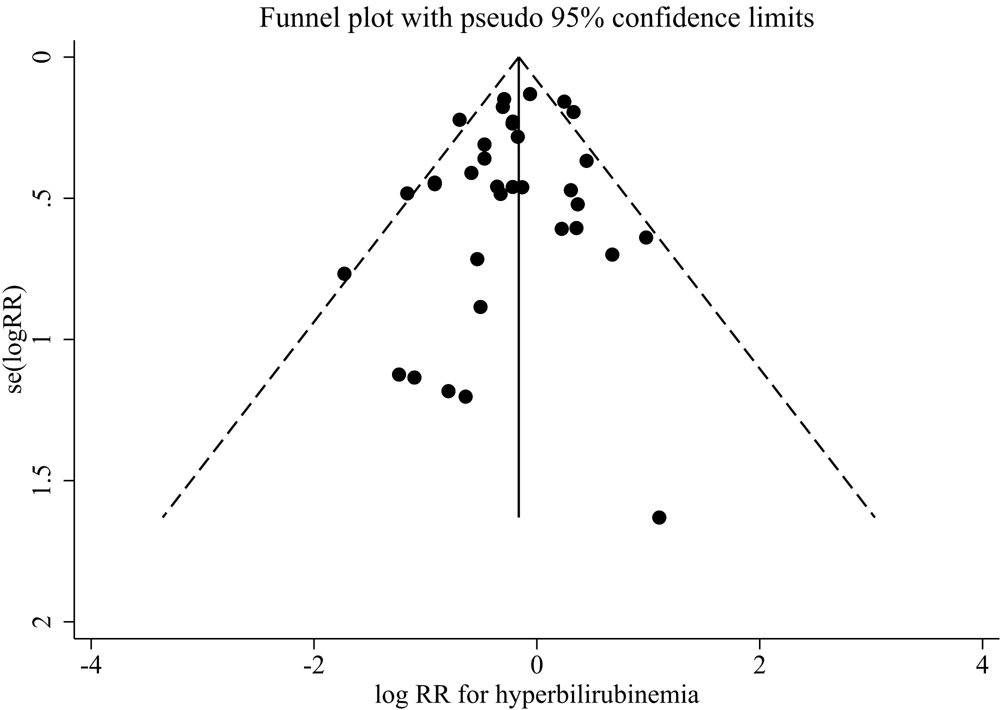

Funnel plot with pseudo 95% confidence limits
0
.5
1
1.5
2
se(logRR)
-4
-2
0
2
4
log RR for hyperbilirubinemia

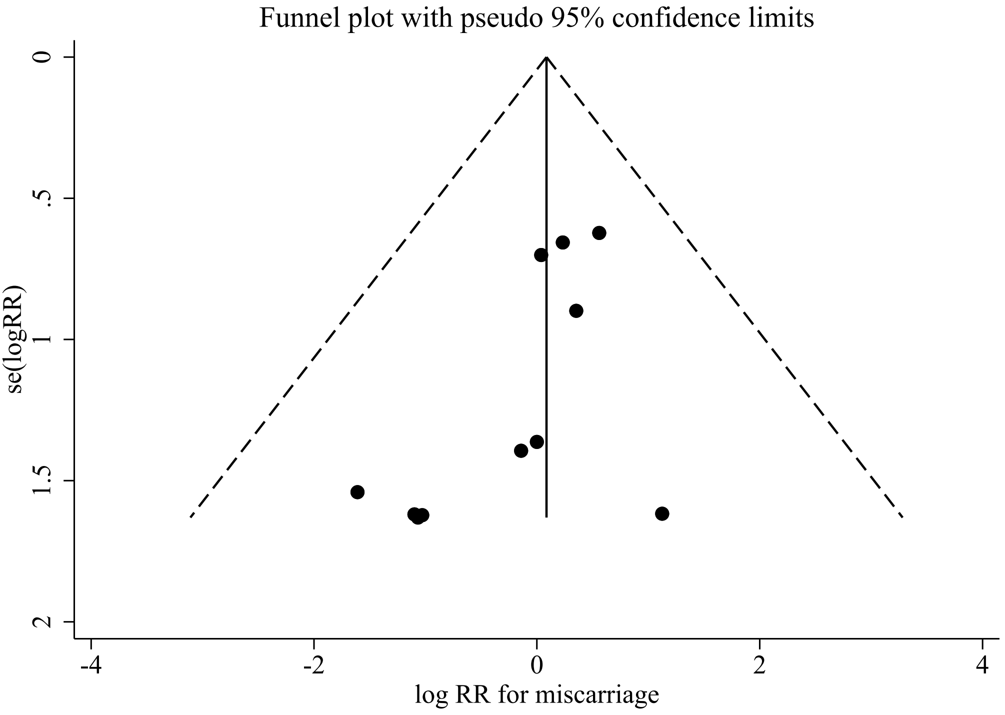
Funnel plot with pseudo 95% confidence limits
0
.5
1
1.5
2
se(logRR)
-4
-2
0
2
4
log RR for miscarriage

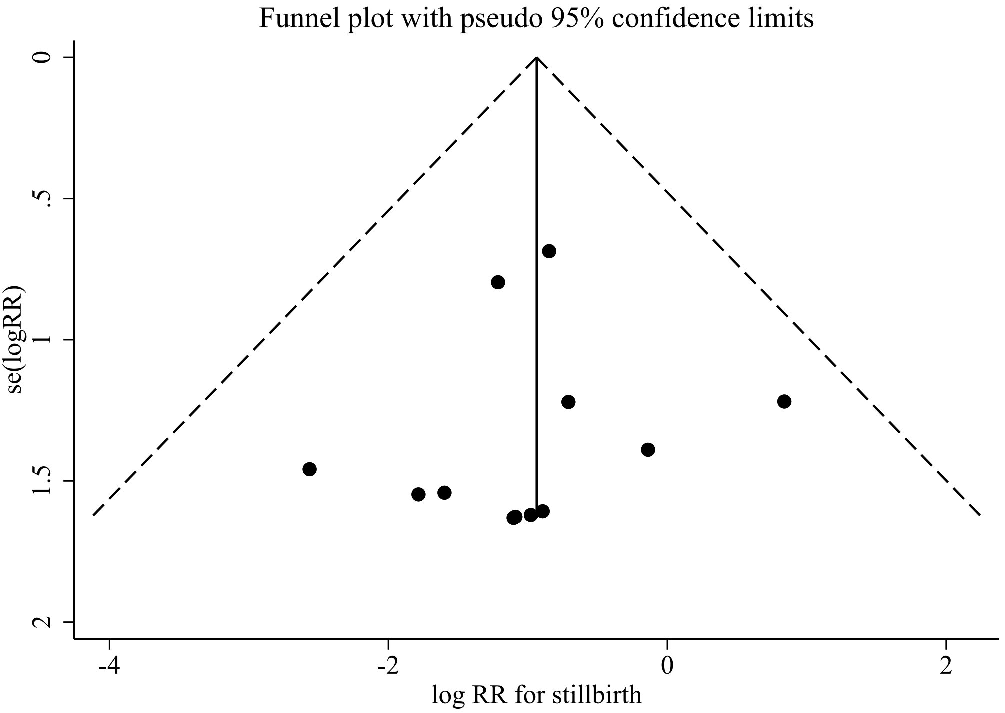

Funnel plot with pseudo 95% confidence limits
0
.5
1
1.5
2
se(logRR)
-4
-2
0
2
log RR for stillbirth

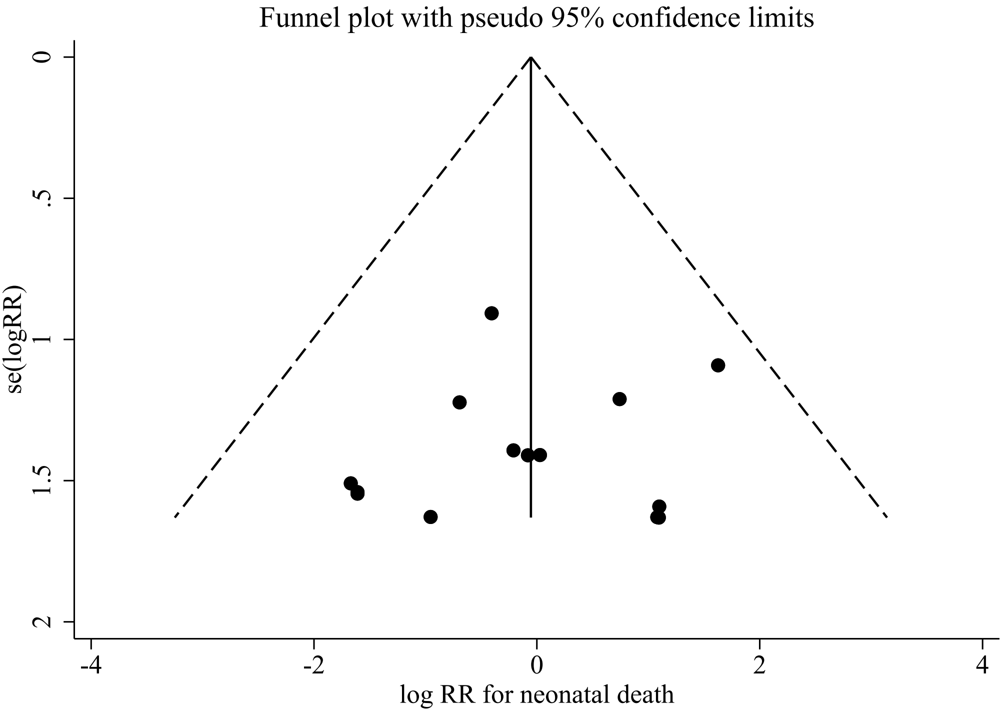
Funnel plot with pseudo 95% confidence limits
se(logRR)
0
.5
1
1.5
2
-4
-2
0
2
4
log RR for neonatal death

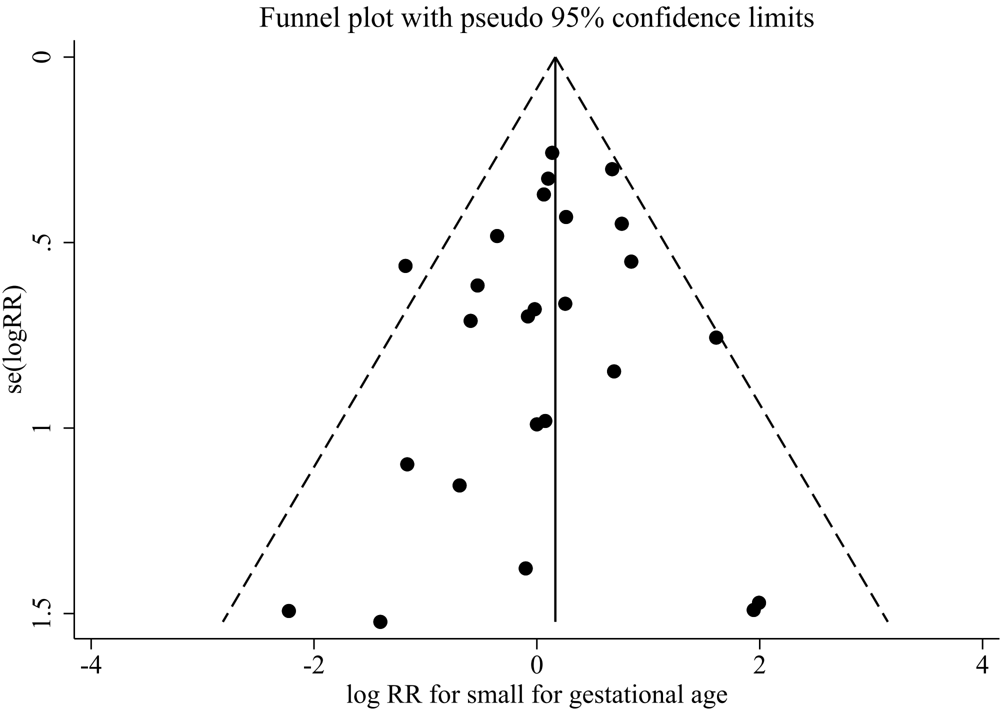
Funnel plot with pseudo 95% confidence limits
0
.5
1
1.5
se(logRR)
-4
-2
0
2
4
log RR for small for gestational age

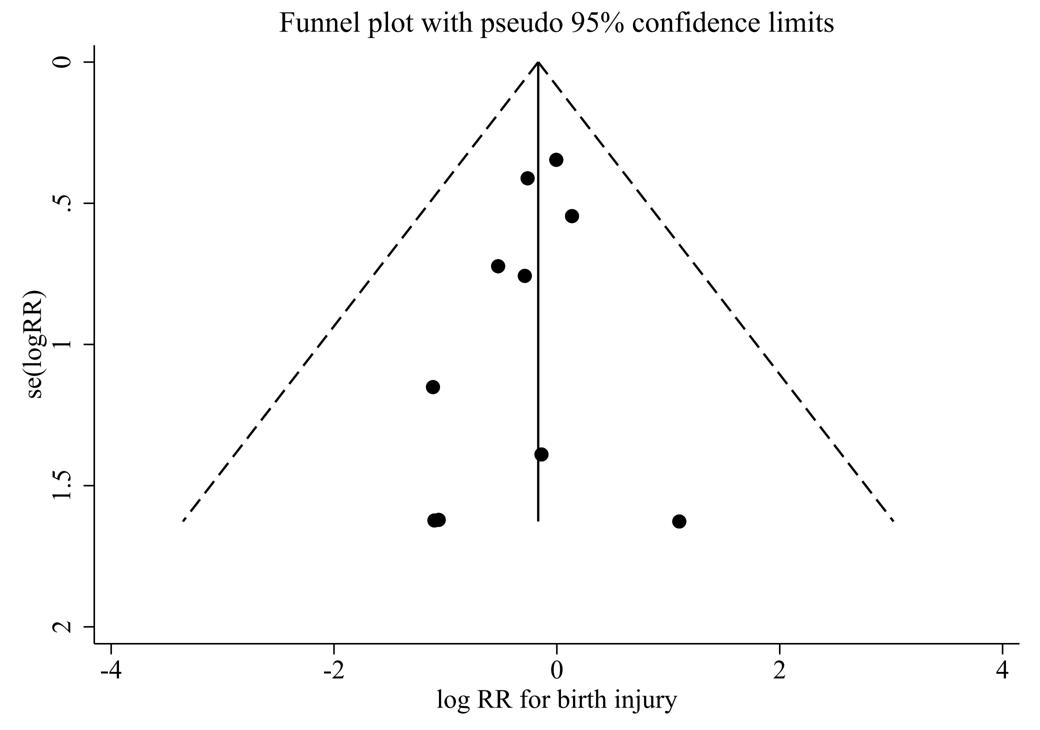

Funnel plot with pseudo 95% confidence limits
0
.5
1
1.5
2
se(logRR)
-4
-2
0
2
4
log RR for birth injury

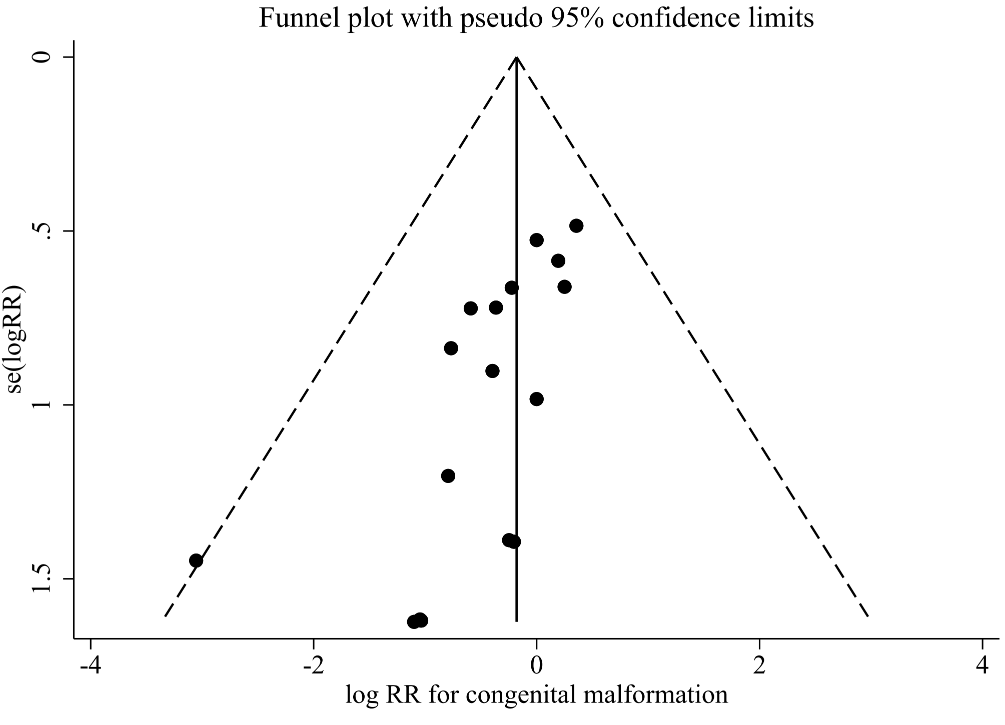
Funnel plot with pseudo 95% confidence limits
0
.5
1
1.5
se(logRR)
-4
-2
0
2
4
log RR for congenital malformation

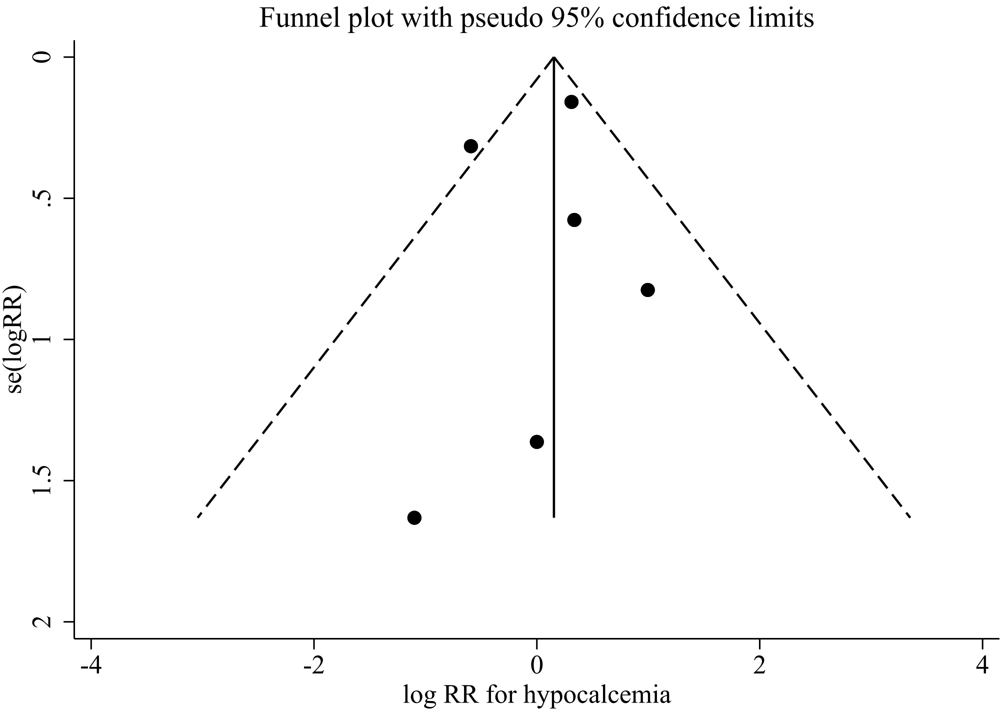

Funnel plot with pseudo 95% confidence limits
0
.5
1
1.5
2
se(logRR)
-4
-2
0
2
4
log RR for hypocalcemia

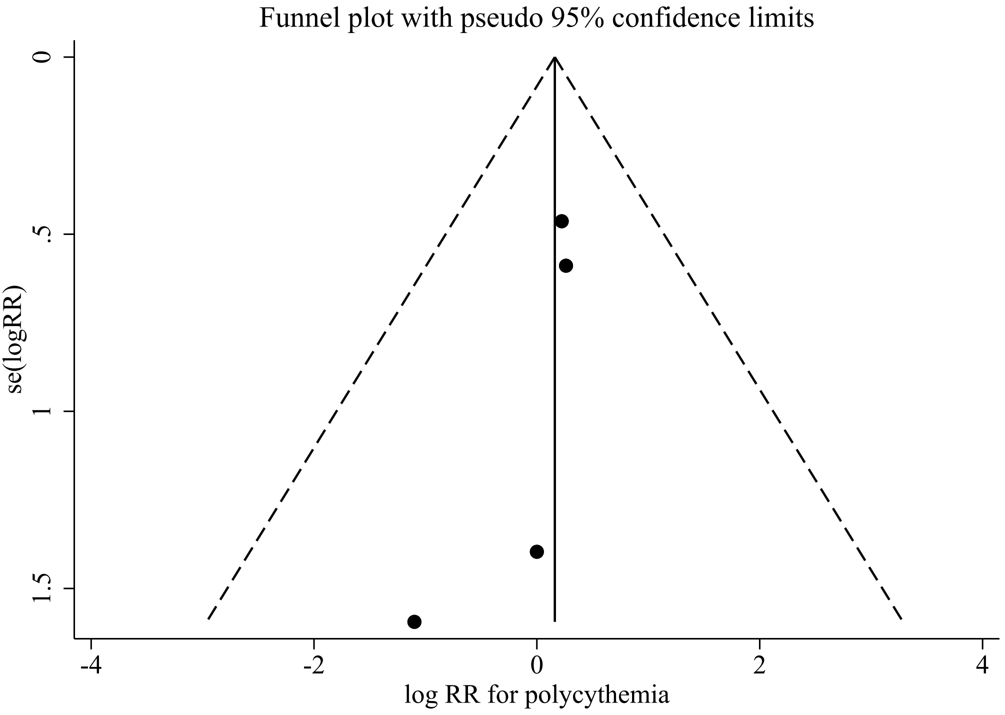

Funnel plot with pseudo 95% confidence limits
0
.5
1
1.5
se(logRR)
-4
-2
0
2
4
log RR for polycythemia

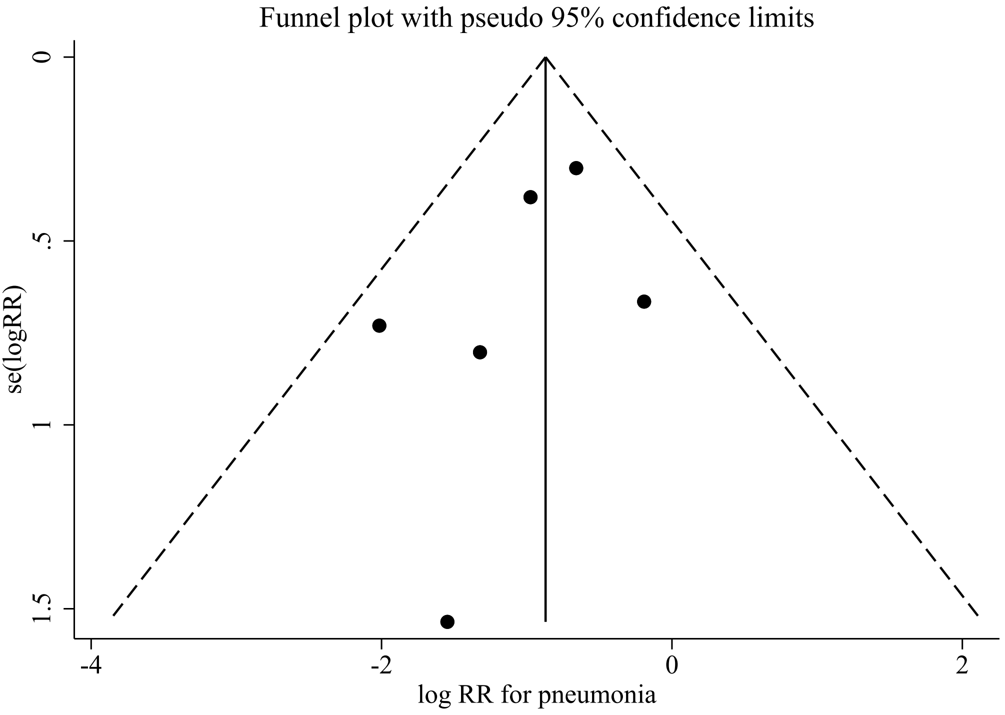
Funnel plot with pseudo 95% confidence limits
se(logRR)
0
.5
1
1.5
-4
-2
0
2
log RR for pneumonia

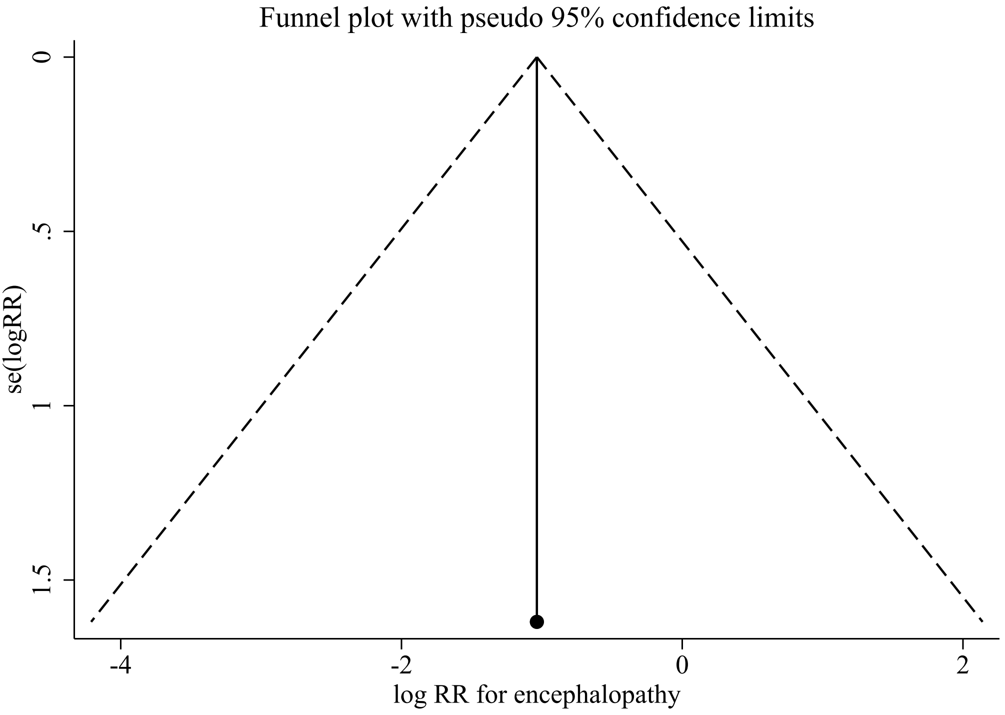
Funnel plot with pseudo 95% confidence limits
0
.5
1
1.5
se(logRR)
-4
-2
0
2
log RR for encephalopathy

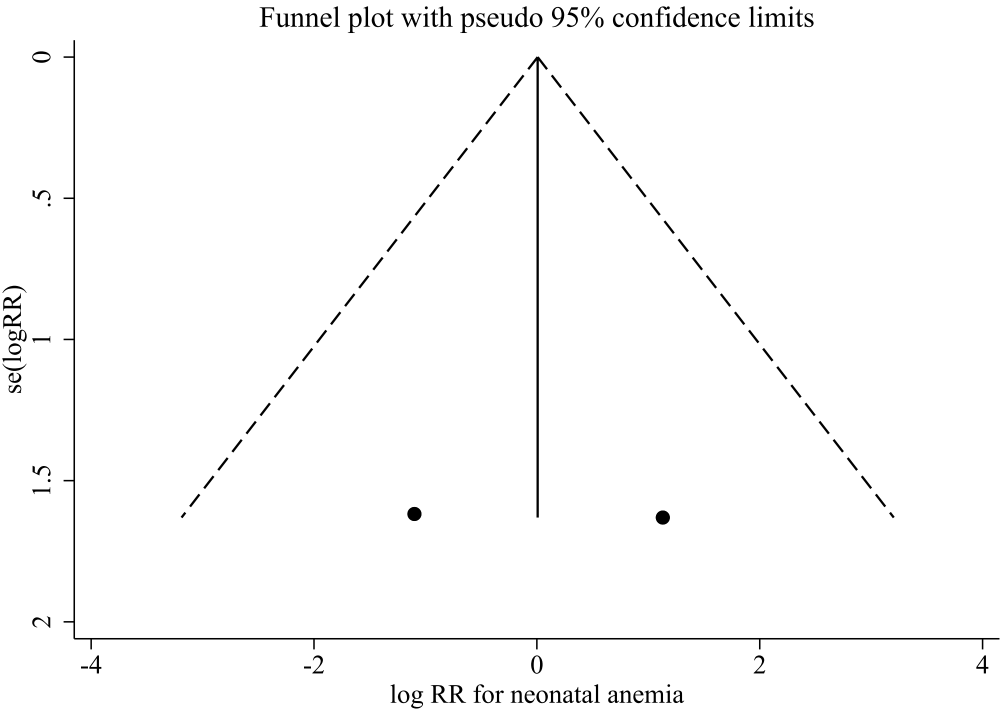
Funnel plot with pseudo 95% confidence limits
0
.5
1
1.5
2
se(logRR)
-4
-2
0
2
4
log RR for neonatal anemia
