# Supplementary material for: Relationship Between Improvements in Glycemic Control and Risk of Pregnancy Complications in Patients With Diabetes Mellitus: Metaregression Analysis of Randomized Controlled Trials of Intensive Glucose Management
Source: J Diabetes Res. 2025 Jun 23;2025:3490884. doi: 10.1155/jdr/3490884 (PMC12208766; doi:10.1155/jdr/3490884)
Supplement: Supporting Information 3 — Scatterplots of reductions in each glycemic indicator and relative risks (RRs) for 14 main adverse pregnancy outcomes. Solid regression lines correspond to all scatterplots and dashed lines correspond to scatterplots from trials of patients with gestational diabetes. The size of circles is proportional to study weight (i.e., inverse of variance of logarithm of RR). Gray circles show trials for gestational diabetes, and white circles show trials for other type of diabetes. The bold regression line indicates that the association was statistically significant, and the thin regression line indicates that the association was not significant. The upper regression formula was determined by analyzing all trials; the lower regression formula was determined limiting the metaregression to trials for gestational diabetes. Panels showing only one regression line and formula indicate that all trials targeted only patients with gestational diabetes. Abbreviations: 2hPG, 2-h postprandial glucose; A1C, hemoglobin A1c; FPG, fasting plasma glucose; MBG, mean blood glucose. [file 3490884.f3.pdf]

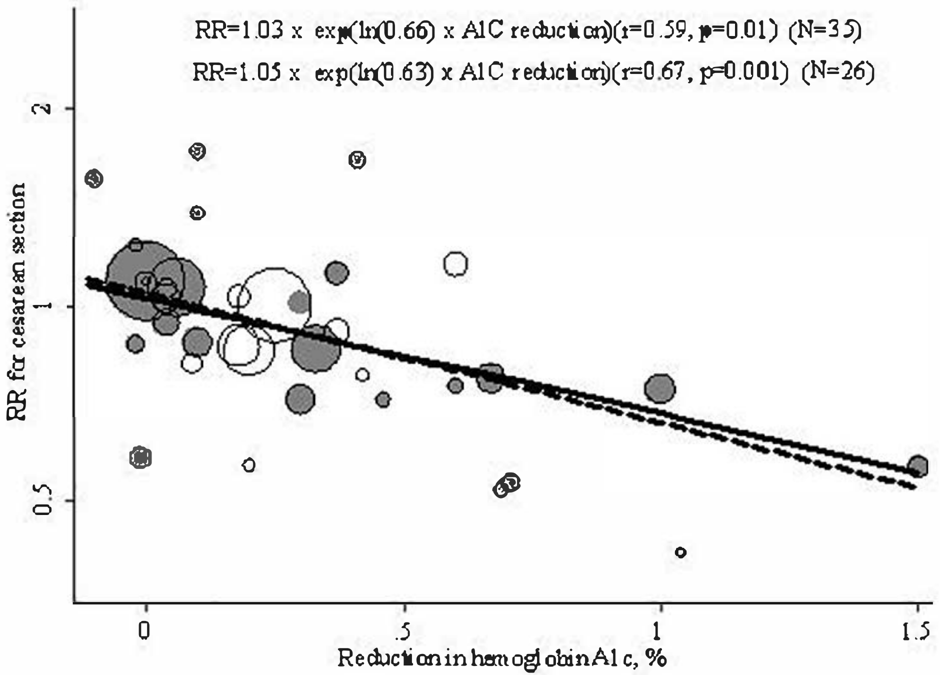
RR=1.03 x exp(ln(0.66) x A1C reduction)(r=0.59, p=0.01) (N=35)
RR=1.05 x exp(ln(0.63) x A1C reduction)(r=0.67, p=0.001) (N=26)
RR for cesarean section
2
1
0.5
0
.5
1
1.5
Reduction in hemoglobin A1c, %

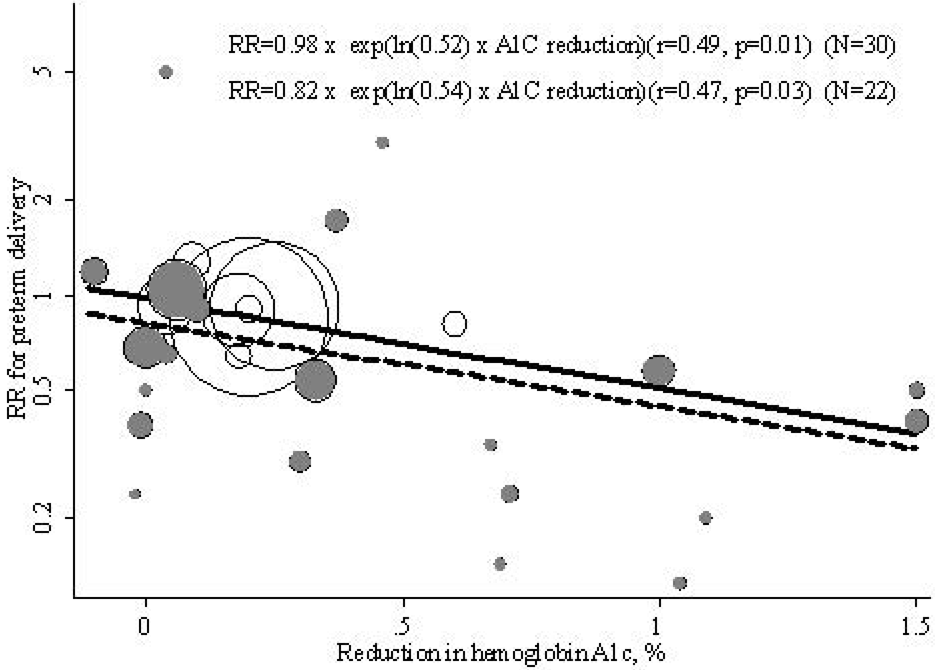
RR=0.98 x exp(ln(0.52) x A1C reduction)(r=0.49, p=0.01) (N=30)
RR=0.82 x exp(ln(0.54) x A1C reduction)(r=0.47, p=0.03) (N=22)
RR for preterm delivery
5
2
1
0.5
0.2
0
.5
1
1.5
Reduction in hemoglobin A1c, %

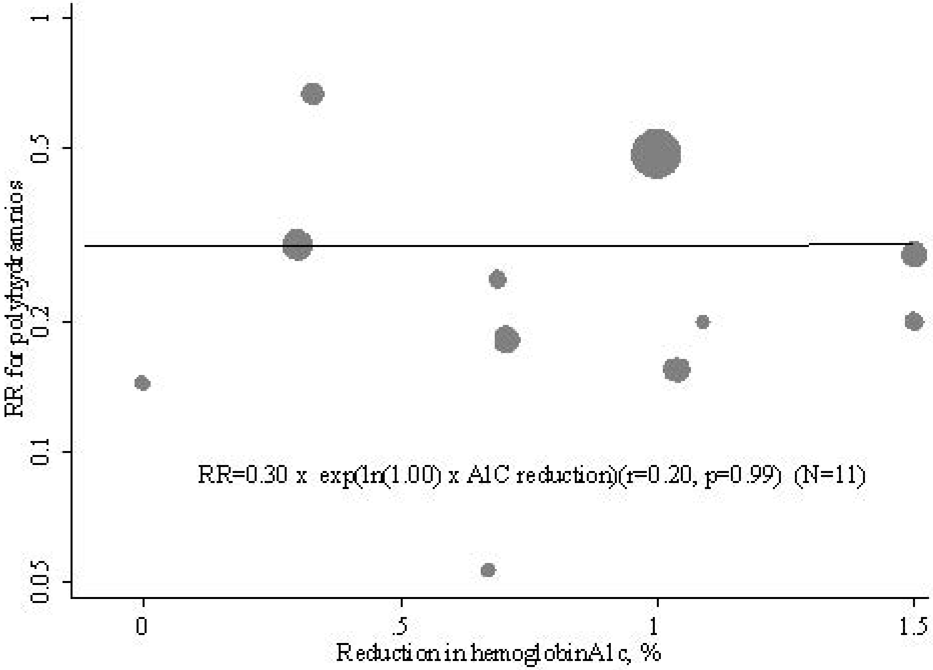
RR for polyhydramnios
1
0.5
0.2
0.1
0.05
0
.5
1
1.5
Reduction in hemoglobinA1c, %
RR=0.30 x exp(ln(1.00) x A1C reduction)(r=0.20, p=0.99) (N=11)

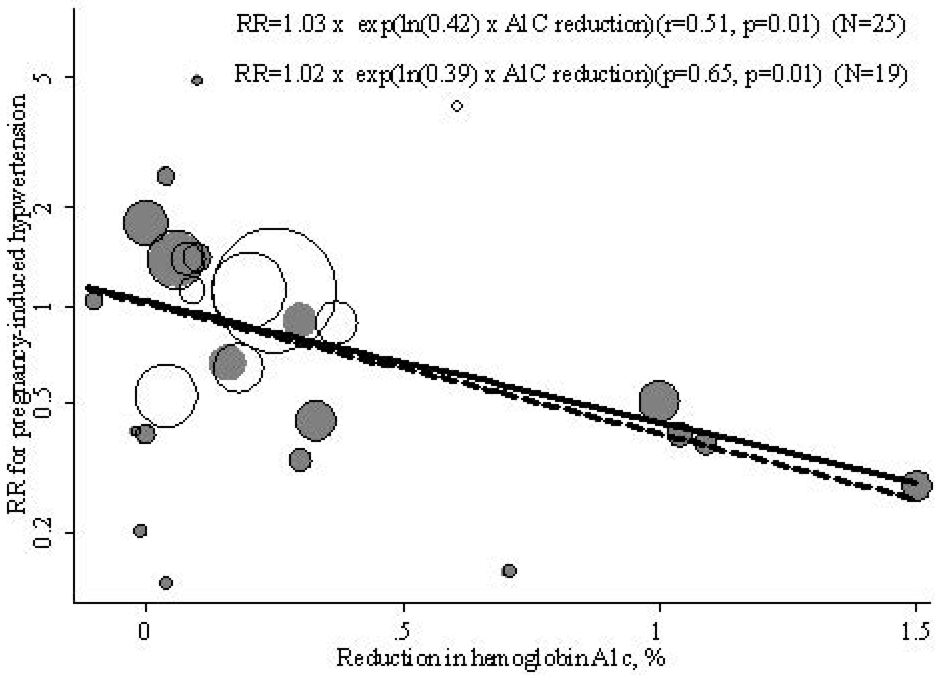

RR=1.03 x exp(ln(0.42) x A1C reduction)(r=0.51, p=0.01) (N=25)
RR=1.02 x exp(ln(0.39) x A1C reduction)(p=0.65, p=0.01) (N=19)
RR for pregnancy-induced hypertension
5
2
1
0.5
0.2
0
.5
1
1.5
Reduction in hemoglobin A1c, %

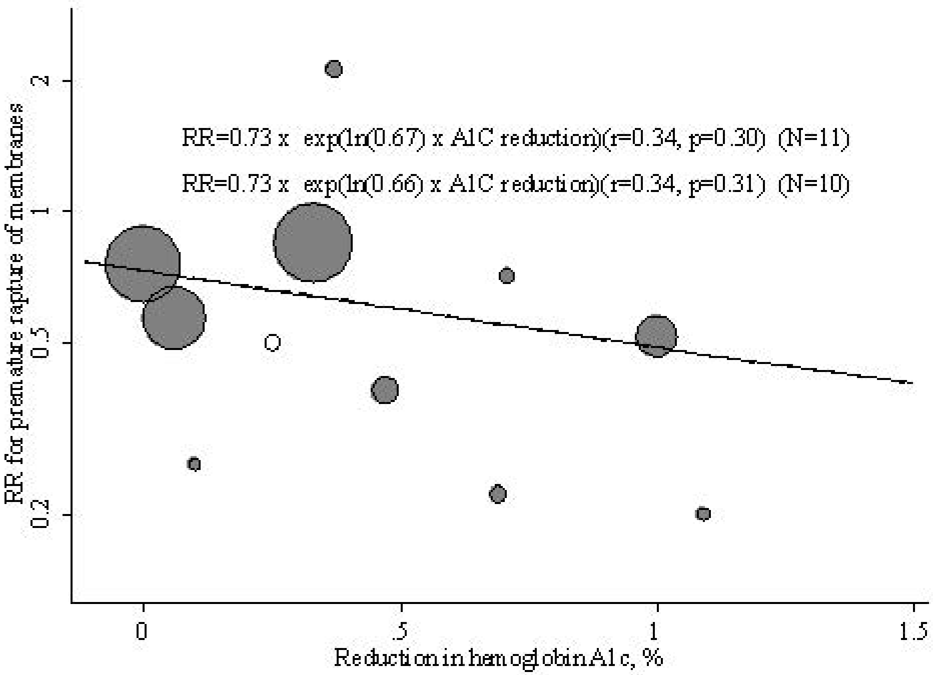

RR=0.73 x exp(ln(0.67) x A1C reduction)(r=0.34, p=0.30) (N=11)
RR=0.73 x exp(ln(0.66) x A1C reduction)(r=0.34, p=0.31) (N=10)
RR for premature rupture of membranes
2
1
0.5
0.2
0
.5
1
1.5
Reduction in hemoglobin A1c, %

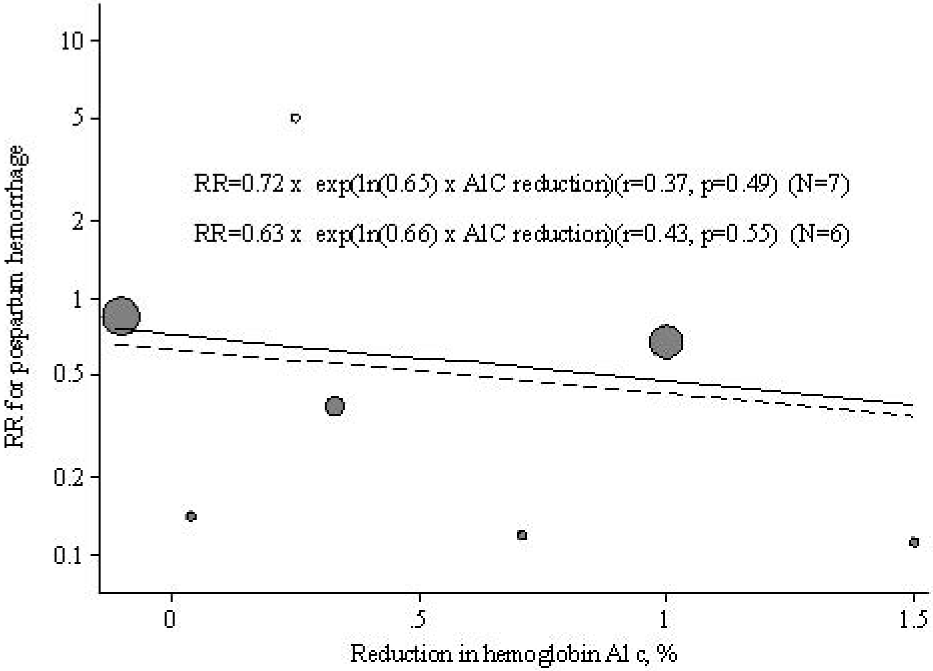
RR=0.72 x exp(ln(0.65) x A1C reduction)(r=0.37, p=0.49) (N=7)
RR=0.63 x exp(ln(0.66) x A1C reduction)(r=0.43, p=0.55) (N=6)
RR for postpartum hemorrhage
10
5
2
1
0.5
0.2
0.1
0
.5
1
1.5
Reduction in hemoglobin A1c, %

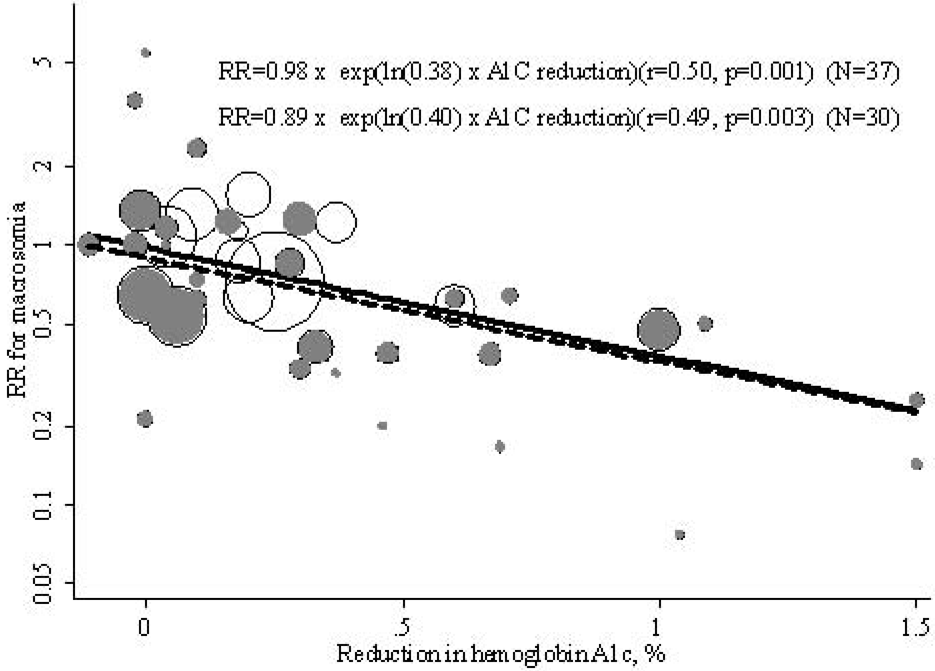
RR=0.98 x exp(ln(0.38) x A1C reduction)(r=0.50, p=0.001) (N=37)
RR=0.89 x exp(ln(0.40) x A1C reduction)(r=0.49, p=0.003) (N=30)
RR for macrosomia
5
2
1
0.5
0.2
0.1
0.05
0
.5
1
1.5
Reduction in hemoglobin A1c, %

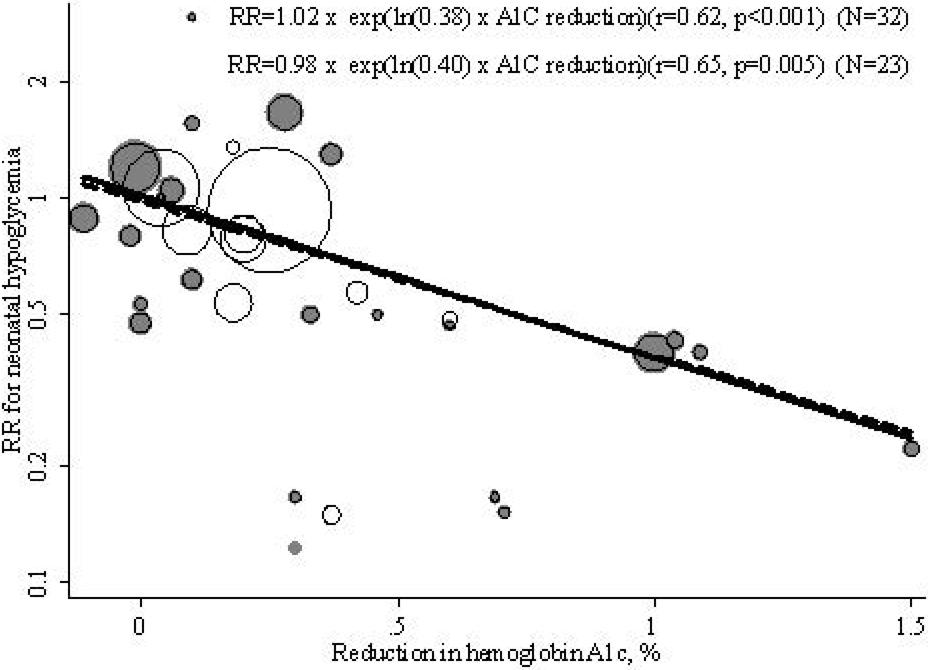
RR=1.02 x exp(ln(0.38) x A1C reduction)(r=0.62, p<0.001) (N=32)
RR=0.98 x exp(ln(0.40) x A1C reduction)(r=0.65, p=0.005) (N=23)
RR for neonatal hypoglycemia
0.1
0.2
0.5
1
2
0
.5
1
1.5
Reduction in hemoglobin A1c, %

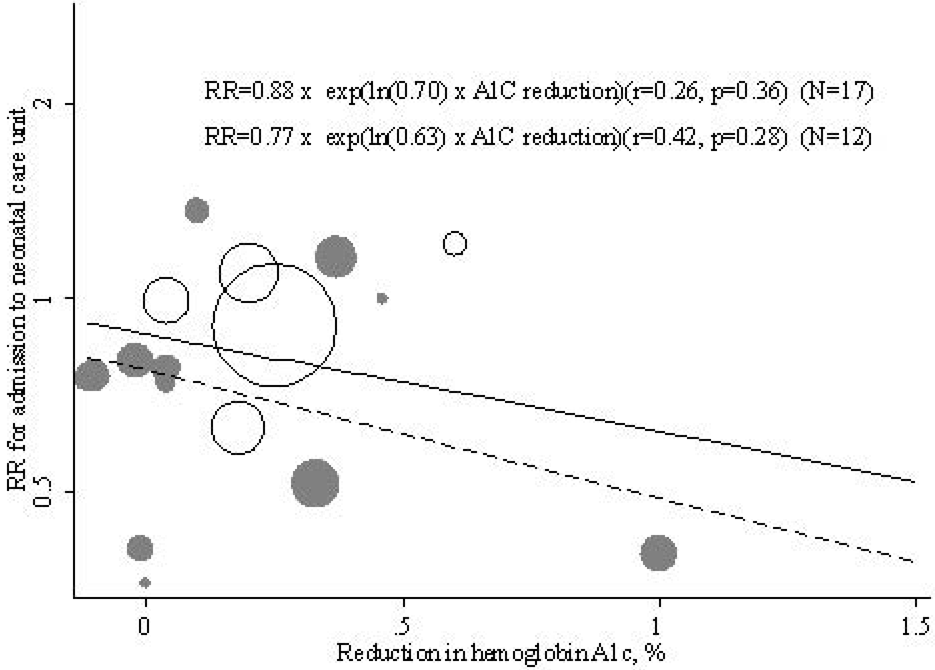
RR=0.88 x exp(ln(0.70) x A1C reduction)(r=0.26, p=0.36) (N=17)
RR=0.77 x exp(ln(0.63) x A1C reduction)(r=0.42, p=0.28) (N=12)
RR for admission to neonatal care unit
2
1
0.5
0
.5
1
1.5
Reduction in hemoglobin A1c, %

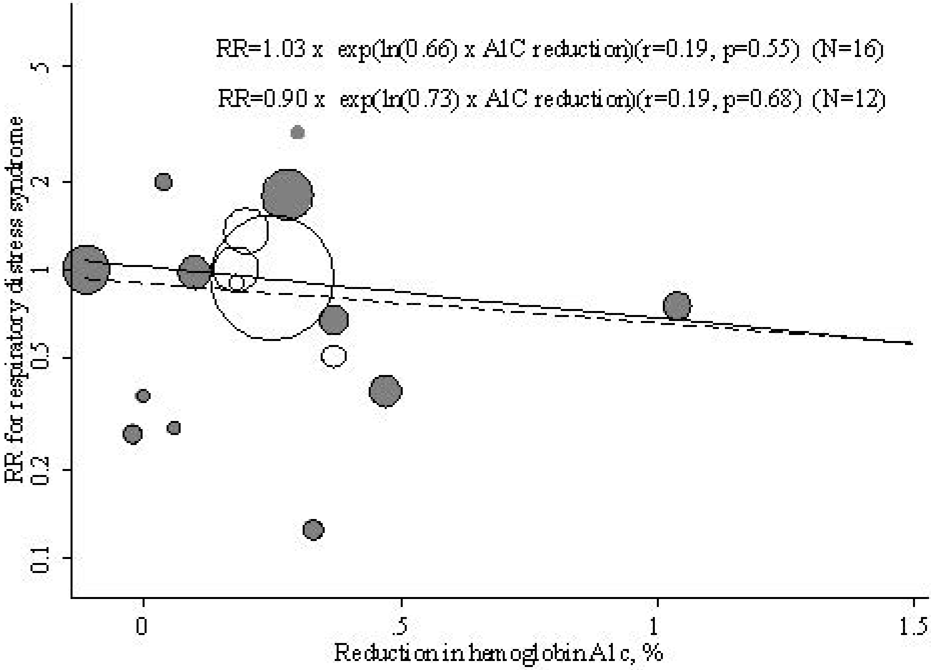
RR=1.03 x exp(ln(0.66) x A1C reduction)(r=0.19, p=0.55) (N=16)
RR=0.90 x exp(ln(0.73) x A1C reduction)(r=0.19, p=0.68) (N=12)
RR for respiratory distress syndrome
5
2
1
0.5
0.2
0.1
0
.5
1
1.5
Reduction in hemoglobin A1c, %

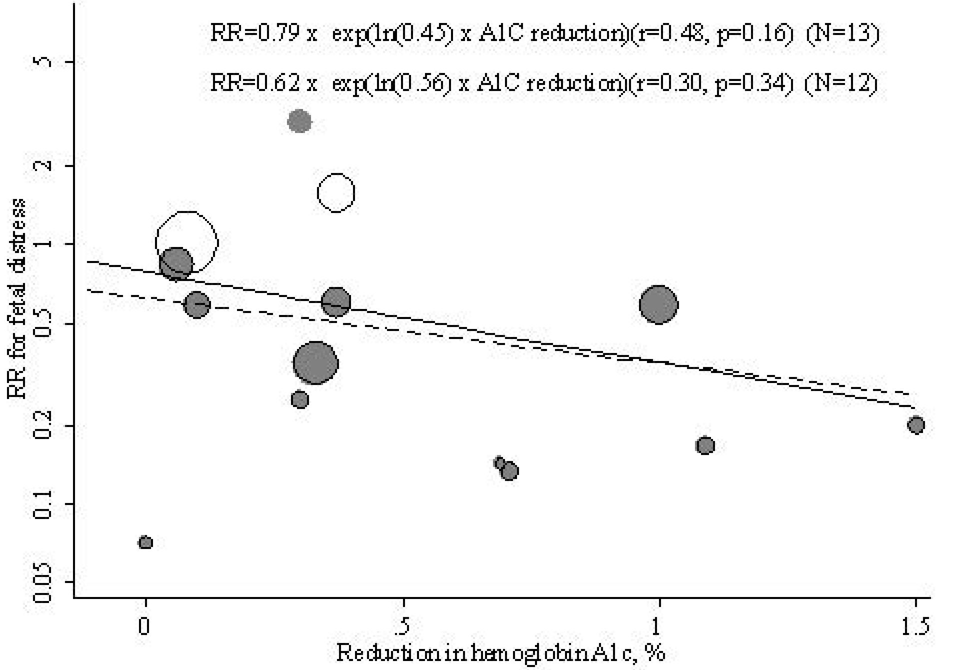
RR=0.79 x exp(ln(0.45) x A1C reduction)(r=0.48, p=0.16) (N=13)
RR=0.62 x exp(ln(0.56) x A1C reduction)(r=0.30, p=0.34) (N=12)
RR for fetal distress
5
2
1
0.5
0.2
0.1
0.05
0
.5
1
1.5
Reduction in hemoglobin A1c, %

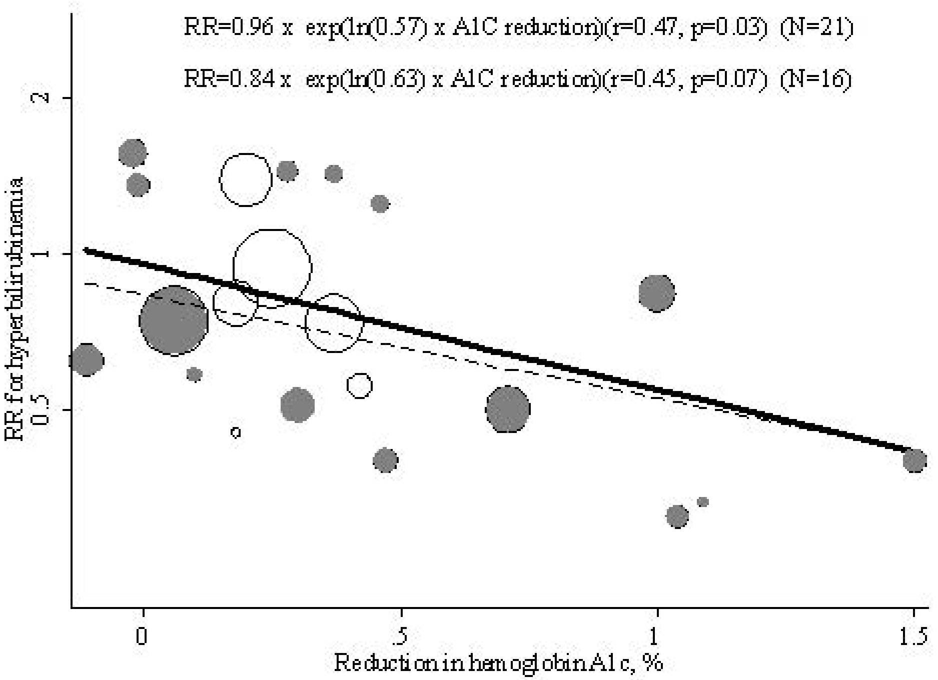
RR=0.96 x exp(ln(0.57) x A1C reduction)(r=0.47, p=0.03) (N=21)
RR=0.84 x exp(ln(0.63) x A1C reduction)(r=0.45, p=0.07) (N=16)
RR for hyperbilirubinemia
2
1
0.5
0
.5
1
1.5
Reduction in hemoglobin A1c, %

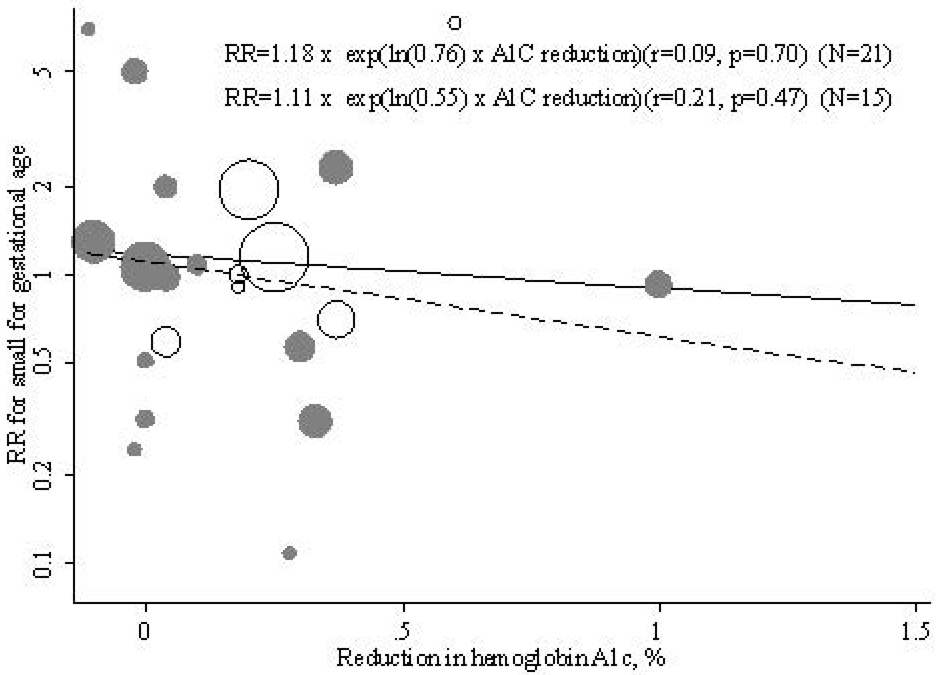
RR=1.18 x exp(ln(0.76) x A1C reduction)(r=0.09, p=0.70) (N=21)
RR=1.11 x exp(ln(0.55) x A1C reduction)(r=0.21, p=0.47) (N=15)
RR for small for gestational age
5
2
1
0.5
0.2
0.1
0
.5
1
1.5
Reduction in hemoglobin A1c, %

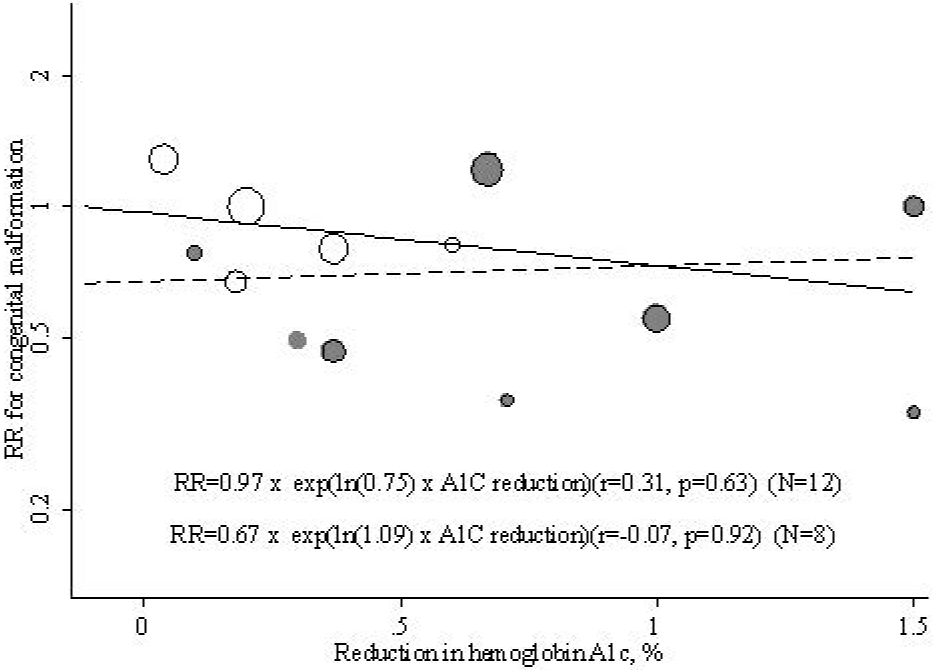

RR for congenital malformation
2
1
0.5
0.2
0
.5
1
1.5
Reduction in hemoglobin A1c, %
RR=0.97 x exp(ln(0.75) x A1C reduction)(r=0.31, p=0.63) (N=12)
RR=0.67 x exp(ln(1.09) x A1C reduction)(r=-0.07, p=0.92) (N=8)

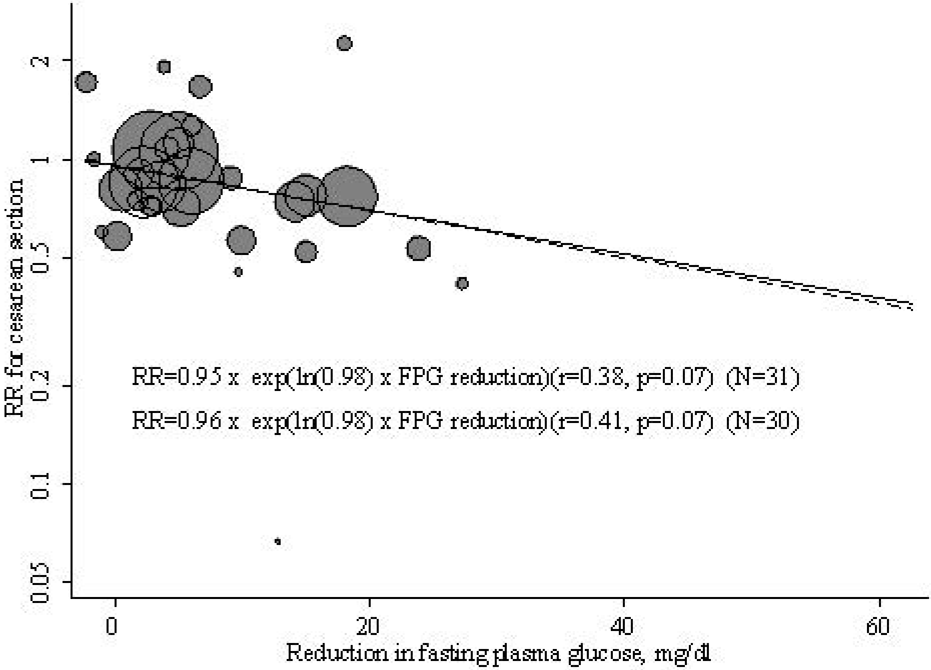

RR for cesarean section
2
1
0.5
0.2
0.1
0.05
0
20
40
60
Reduction in fasting plasma glucose, mg/dl
RR=0.95 x exp(ln(0.98) x FPG reduction)(r=0.38, p=0.07) (N=31)
RR=0.96 x exp(ln(0.98) x FPG reduction)(r=0.41, p=0.07) (N=30)

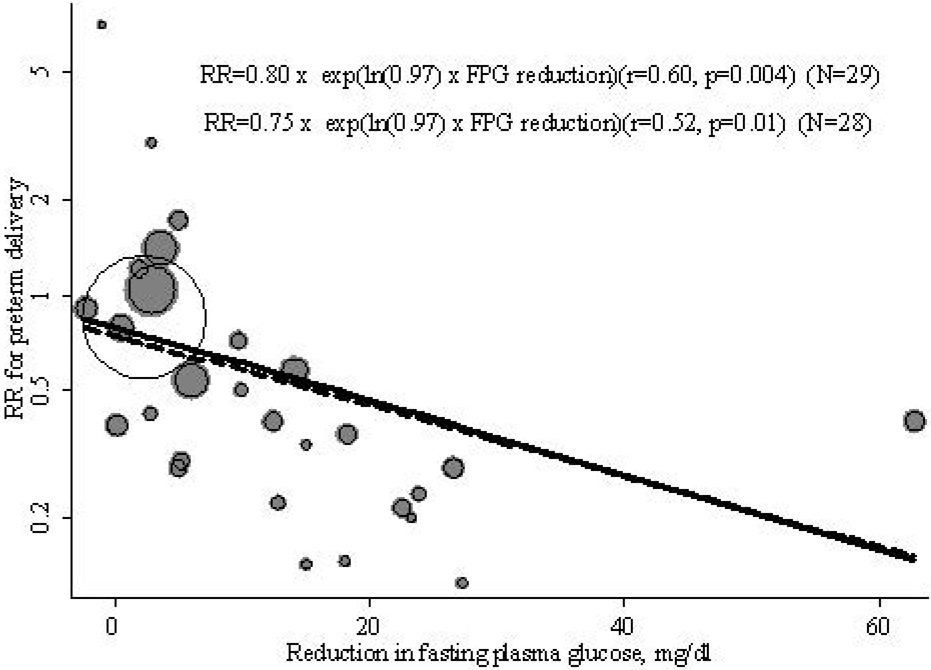
RR=0.80 x exp(ln(0.97) x FPG reduction)(r=0.60, p=0.004) (N=29)
RR=0.75 x exp(ln(0.97) x FPG reduction)(r=0.52, p=0.01) (N=28)
RR for preterm delivery
5
2
1
0.5
0.2
0
20
40
60
Reduction in fasting plasma glucose, mg/dl

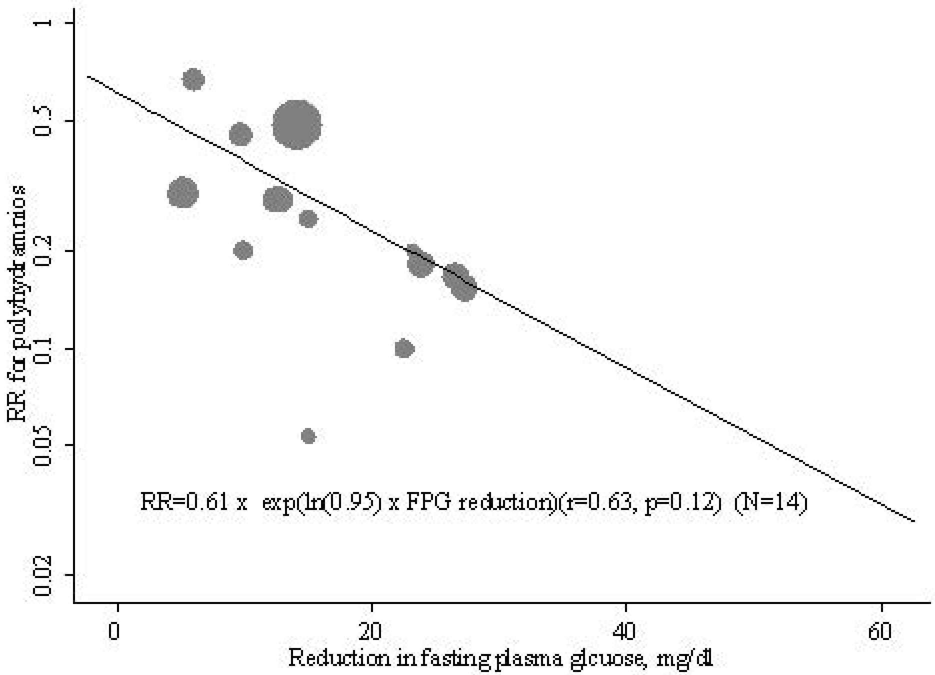
RR for polyhydramnios
1
0.5
0.2
0.1
0.05
0.02
RR=0.61 x exp(ln(0.95) x FPG reduction)(r=0.63, p=0.12) (N=14)
0
20
40
60
Reduction in fasting plasma glcuose, mg/dl

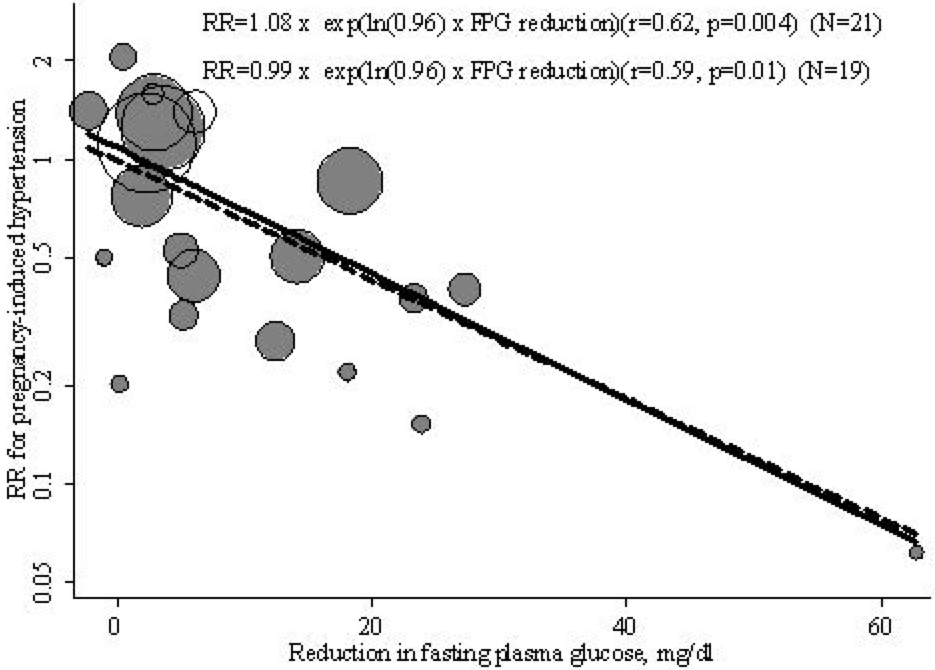
RR=1.08 x exp(ln(0.96) x FPG reduction)(r=0.62, p=0.004) (N=21)
RR=0.99 x exp(ln(0.96) x FPG reduction)(r=0.59, p=0.01) (N=19)
RR for pregnancy-induced hypertension
2
1
0.5
0.2
0.1
0.05
0
20
40
60
Reduction in fasting plasma glucose, mg/dl

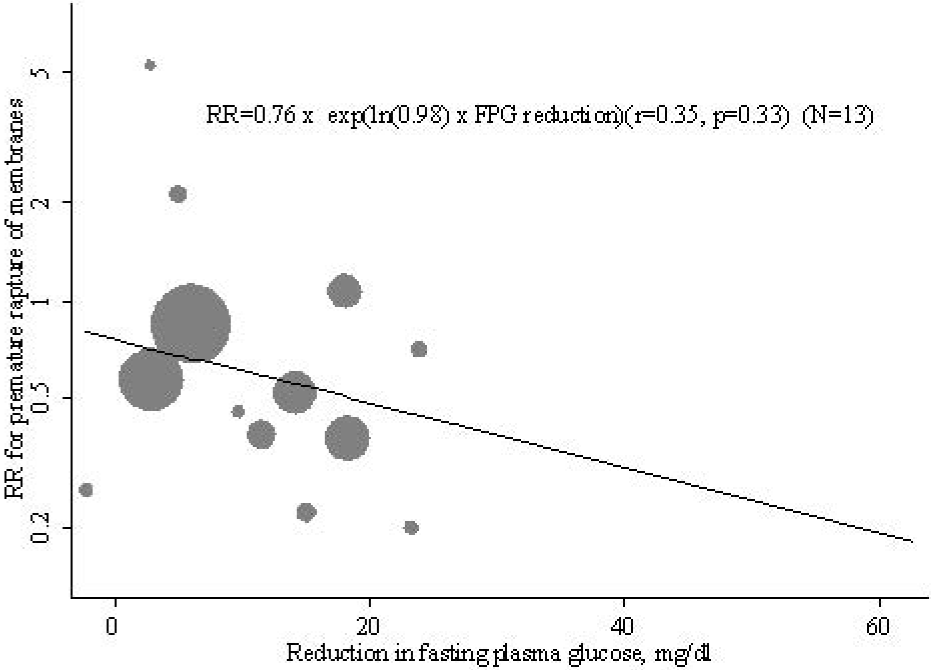
RR=0.76 x exp(ln(0.98) x FPG reduction)(r=0.35, p=0.33) (N=13)
RR for premature rapture of membranes
5
2
1
0.5
0.2
0
20
40
60
Reduction in fasting plasma glucose, mg/dl

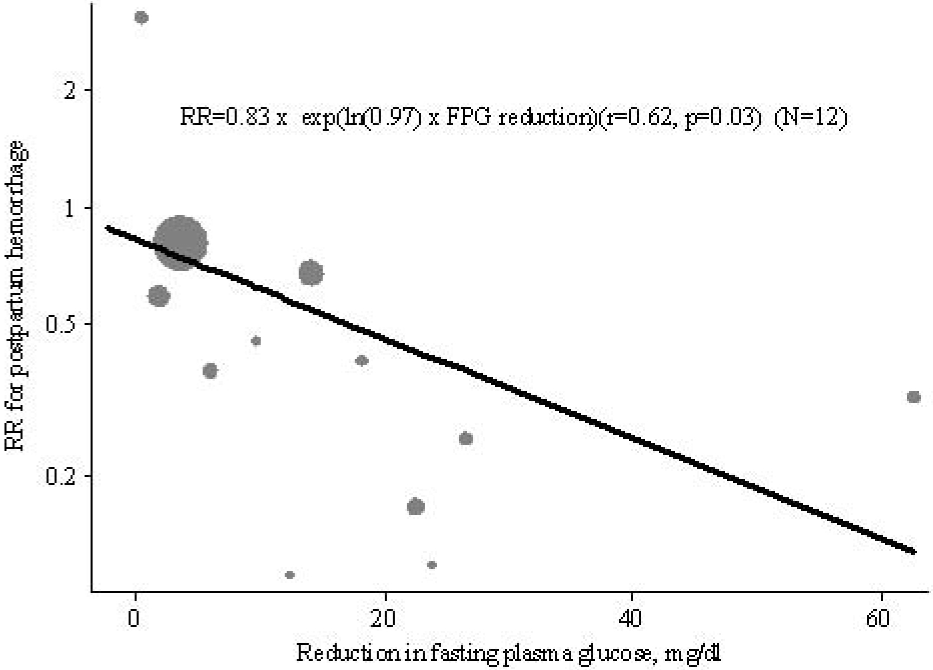

RR=0.83 x exp(ln(0.97) x FPG reduction)(r=0.62, p=0.03) (N=12)
RR for postpartum hemorrhage
2
1
0.5
0.2
0
20
40
60
Reduction in fasting plasma glucose, mg/dl

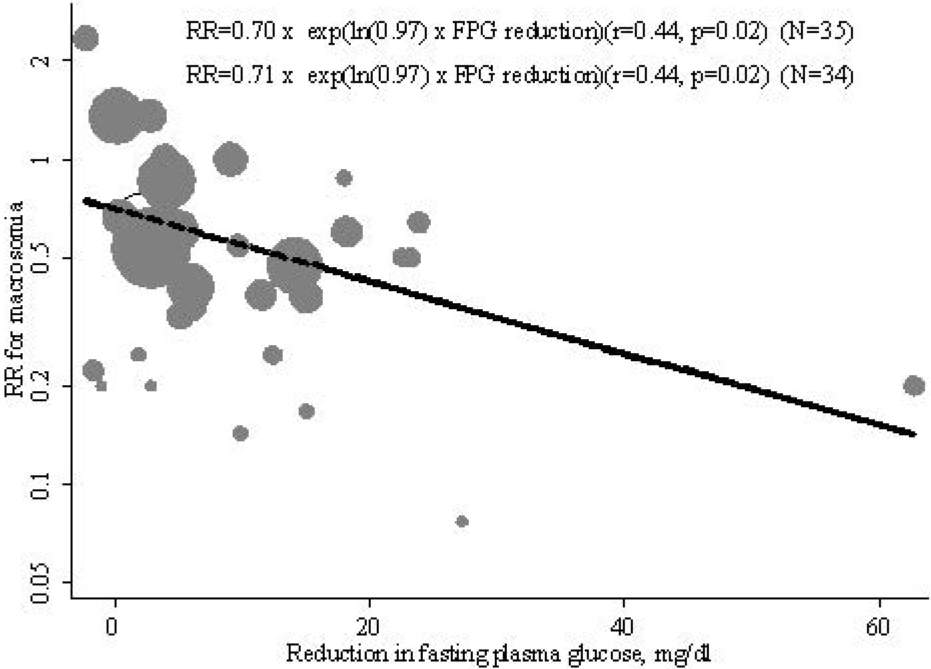
RR=0.70 x exp(ln(0.97) x FPG reduction)(r=0.44, p=0.02) (N=35)
RR=0.71 x exp(ln(0.97) x FPG reduction)(r=0.44, p=0.02) (N=34)
RR for macrosomia
2
1
0.5
0.2
0.1
0.05
0
20
40
60
Reduction in fasting plasma glucose, mg/dl

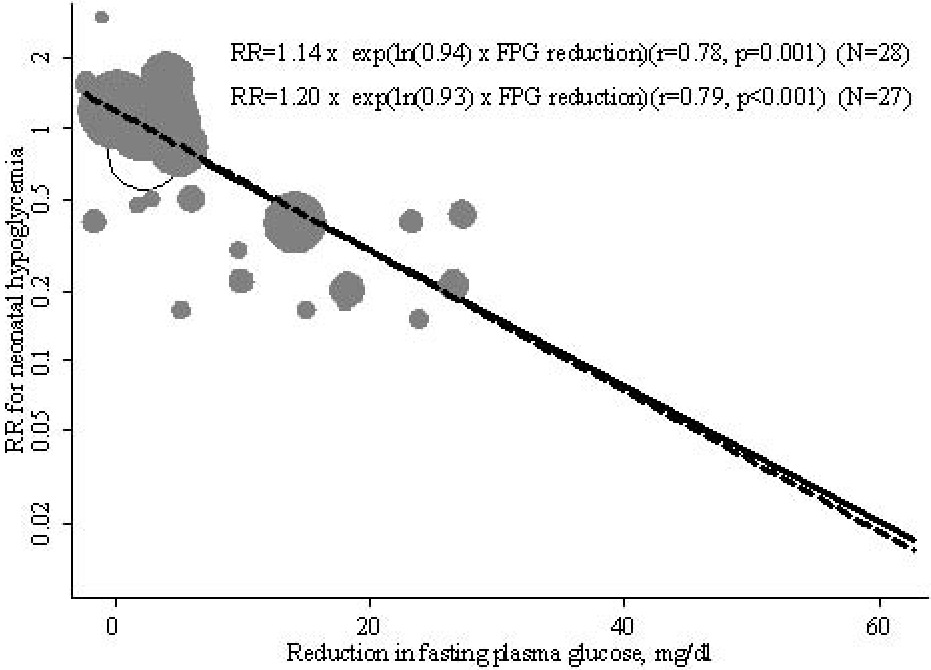

RR=1.14 x exp(ln(0.94) x FPG reduction)(r=0.78, p=0.001) (N=28)
RR=1.20 x exp(ln(0.93) x FPG reduction)(r=0.79, p<0.001) (N=27)
RR for neonatal hypoglycemia
2
1
0.5
0.2
0.1
0.05
0.02
0
20
40
60
Reduction in fasting plasma glucose, mg/dl

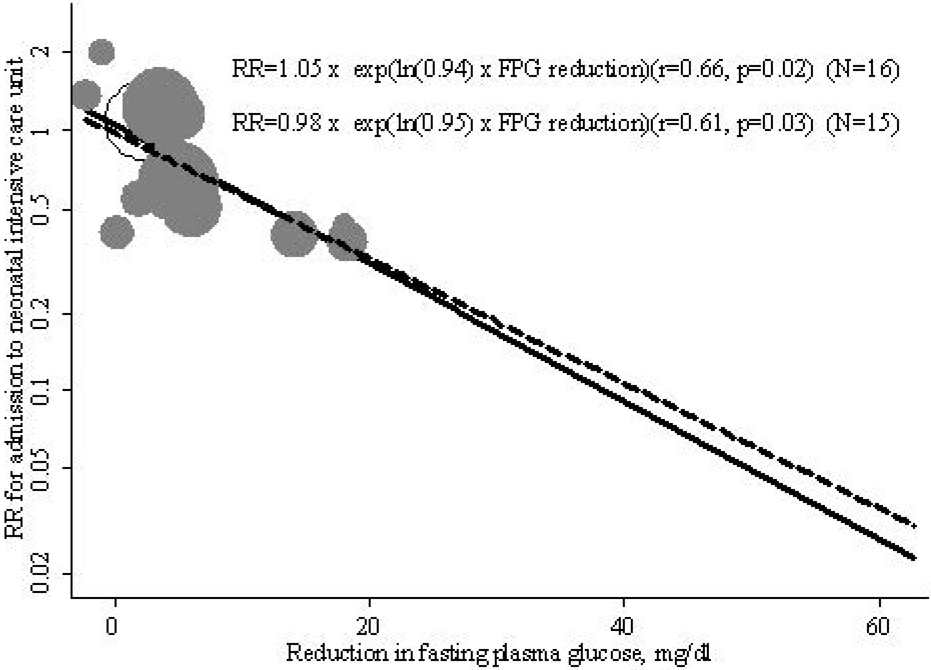
RR=1.05 x exp(ln(0.94) x FPG reduction)(r=0.66, p=0.02) (N=16)
RR=0.98 x exp(ln(0.95) x FPG reduction)(r=0.61, p=0.03) (N=15)
RR for admission to neonatal intensive care unit
2
1
0.5
0.2
0.1
0.05
0.02
0
20
40
60
Reduction in fasting plasma glucose, mg/dl

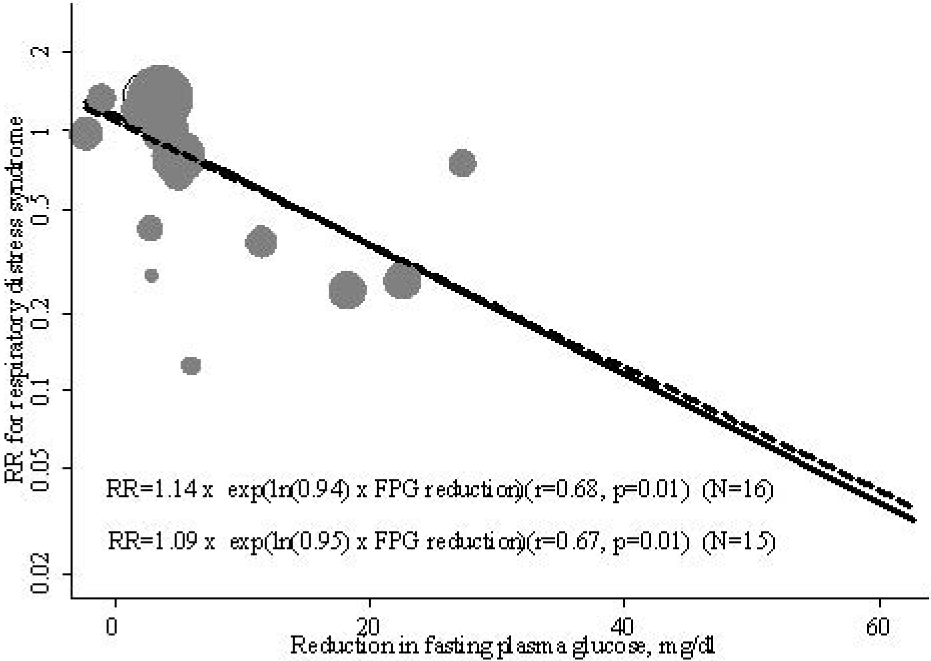

RR for respiratory distress syndrome
2
1
0.5
0.2
0.1
0.05
0.02
0
20
40
60
Reduction in fasting plasma glucose, mg/dl
RR=1.14 x exp(ln(0.94) x FPG reduction)(r=0.68, p=0.01) (N=16)
RR=1.09 x exp(ln(0.95) x FPG reduction)(r=0.67, p=0.01) (N=15)

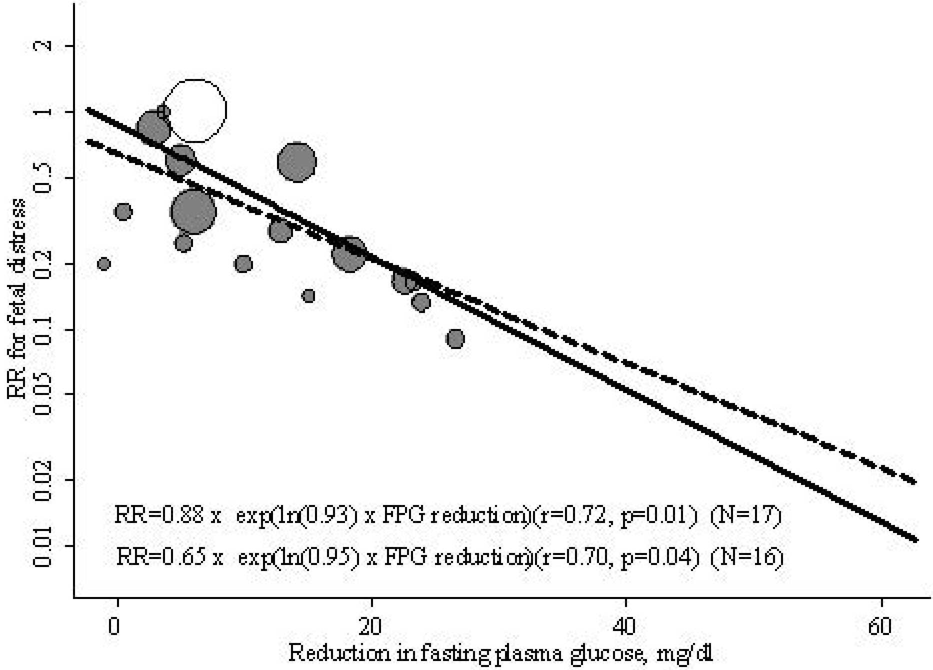

RR for fetal distress
2
1
0.5
0.2
0.1
0.05
0.02
0.01
RR=0.88 x exp(ln(0.93) x FPG reduction)(r=0.72, p=0.01) (N=17)
RR=0.65 x exp(ln(0.95) x FPG reduction)(r=0.70, p=0.04) (N=16)
0
20
40
60
Reduction in fasting plasma glucose, mg/dl

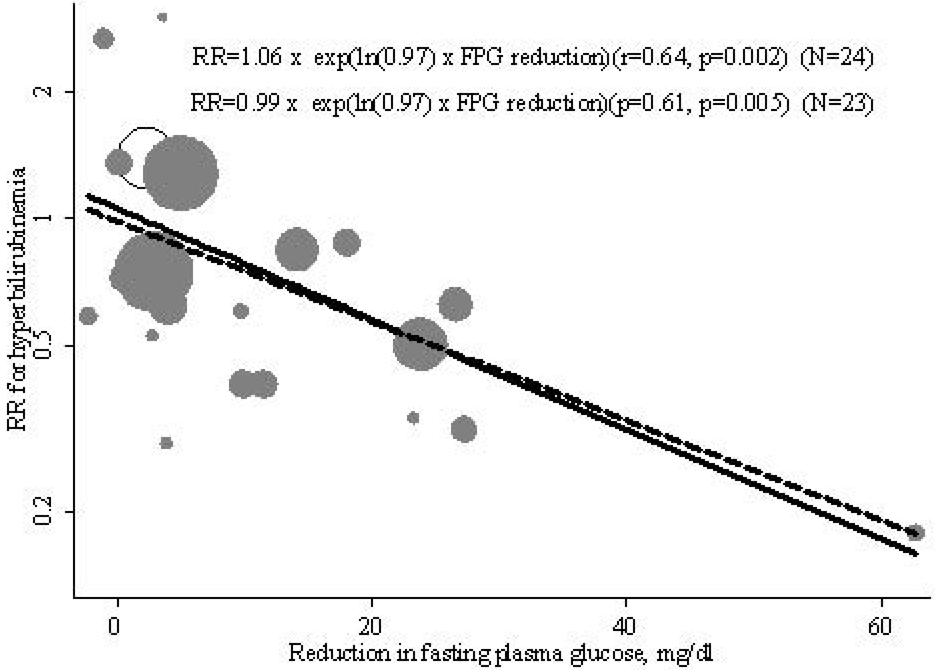
RR=1.06 x exp(ln(0.97) x FPG reduction)(r=0.64, p=0.002) (N=24)
RR=0.99 x exp(ln(0.97) x FPG reduction)(p=0.61, p=0.005) (N=23)
RR for hyperbilirubinemia
2
1
0.5
0.2
0
20
40
60
Reduction in fasting plasma glucose, mg/dl

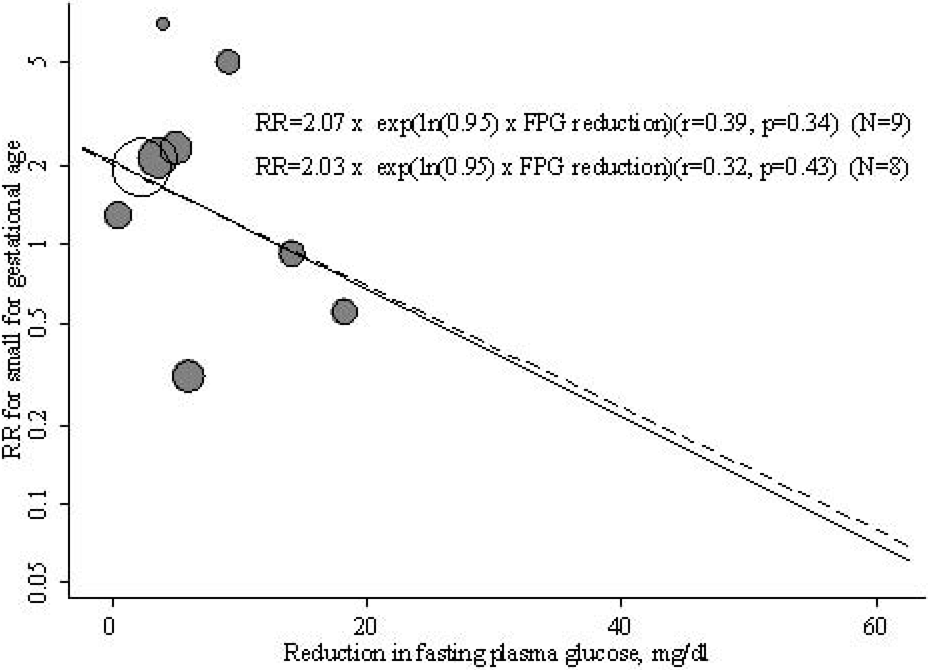
RR=2.07 x exp(ln(0.95) x FPG reduction)(r=0.39, p=0.34) (N=9)
RR=2.03 x exp(ln(0.95) x FPG reduction)(r=0.32, p=0.43) (N=8)
RR for small for gestational age
5
2
1
0.5
0.2
0.1
0.05
0
20
40
60
Reduction in fasting plasma glucose, mg/dl

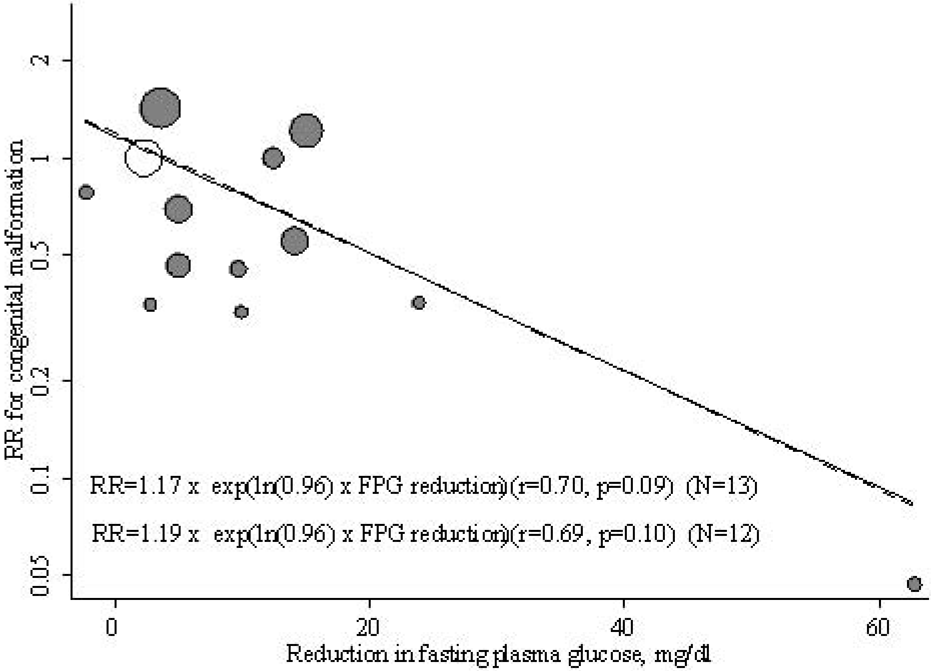

RR for congenital malformation
2
1
0.5
0.2
0.1
0.05
0
20
40
60
Reduction in fasting plasma glucose, mg/dl
RR=1.17 x exp(ln(0.96) x FPG reduction)(r=0.70, p=0.09) (N=13)
RR=1.19 x exp(ln(0.96) x FPG reduction)(r=0.69, p=0.10) (N=12)

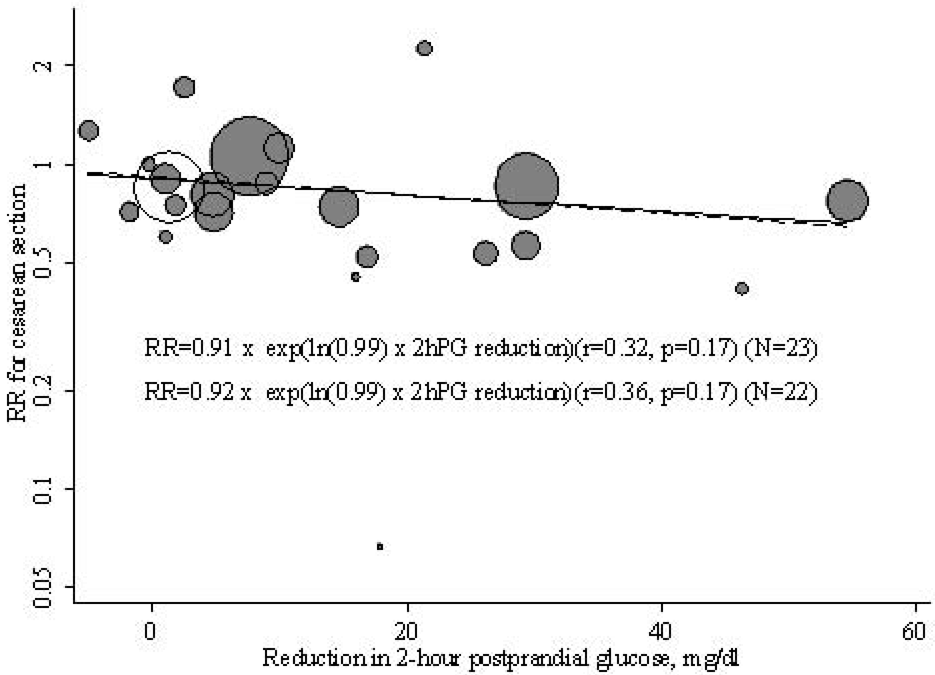

2
1
0.5
0.2
0.1
0.05
RR for cesarean section
RR=0.91 x exp(ln(0.99) x 2hPG reduction)(r=0.32, p=0.17) (N=23)
RR=0.92 x exp(ln(0.99) x 2hPG reduction)(r=0.36, p=0.17) (N=22)
0
20
40
60
Reduction in 2-hour postprandial glucose, mg/dl

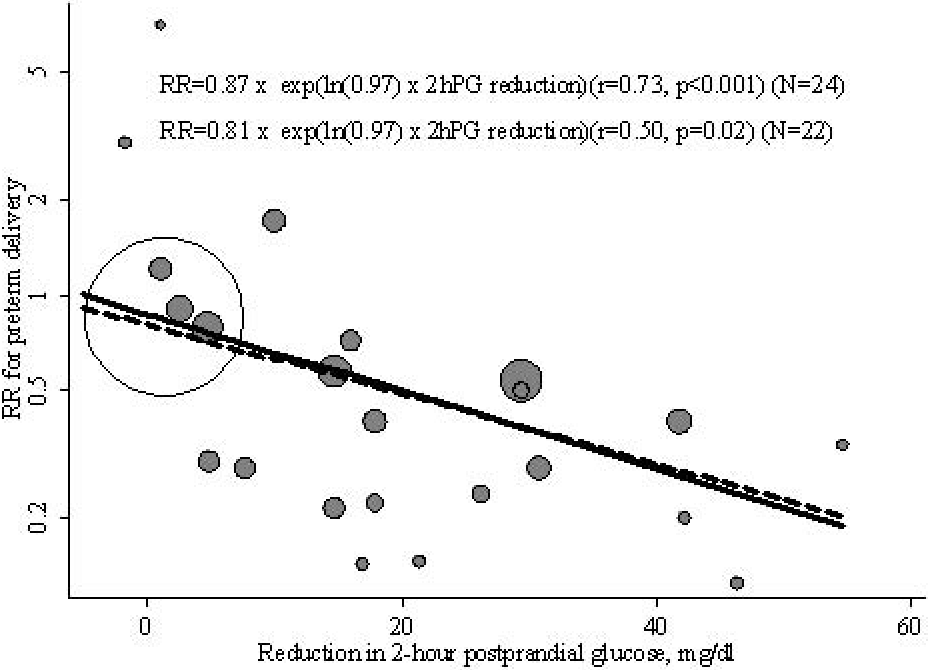
RR=0.87 x exp(ln(0.97) x 2hPG reduction)(r=0.73, p<0.001) (N=24)
RR=0.81 x exp(ln(0.97) x 2hPG reduction)(r=0.50, p=0.02) (N=22)
RR for preterm delivery
5
2
1
0.5
0.2
0
20
40
60
Reduction in 2-hour postprandial glucose, mg/dl

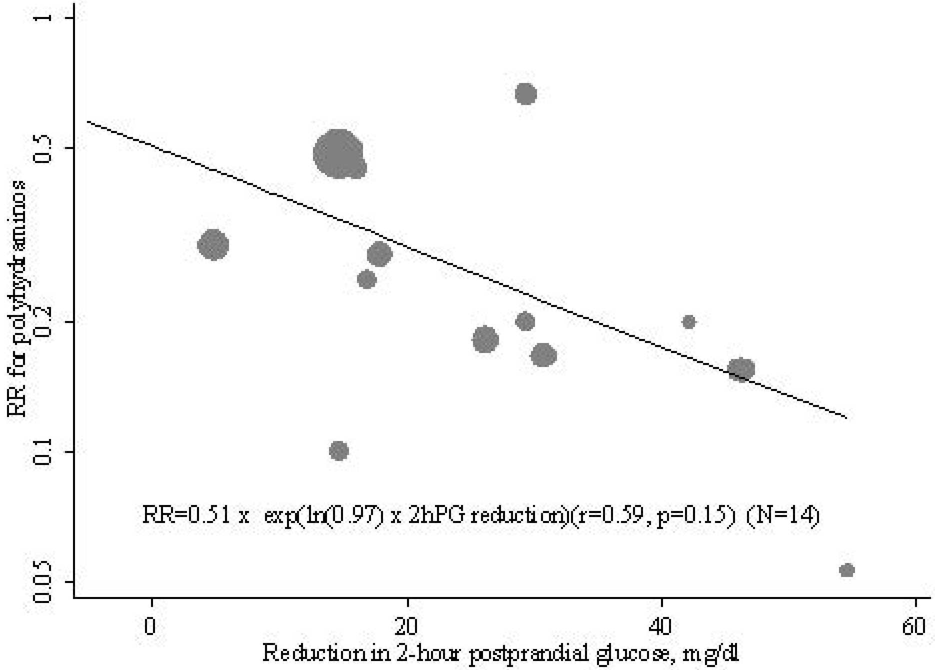

RR for polyhydramnios
1
0.5
0.2
0.1
0.05
RR=0.51 x exp(ln(0.97) x 2hPG reduction)(r=0.59, p=0.15) (N=14)
0
20
40
60
Reduction in 2-hour postprandial glucose, mg/dl

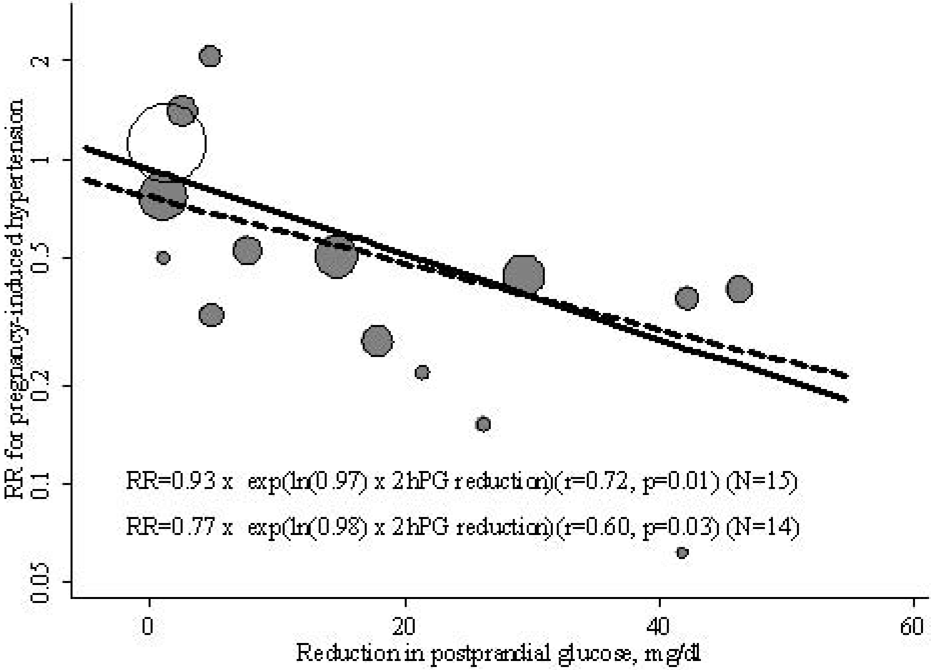

RR for pregnancy-induced hypertension
2
1
0.5
0.2
0.1
0.05
0
20
40
60
Reduction in postprandial glucose, mg/dl
RR=0.93 x exp(ln(0.97) x 2hPG reduction)(r=0.72, p=0.01) (N=15)
RR=0.77 x exp(ln(0.98) x 2hPG reduction)(r=0.60, p=0.03) (N=14)

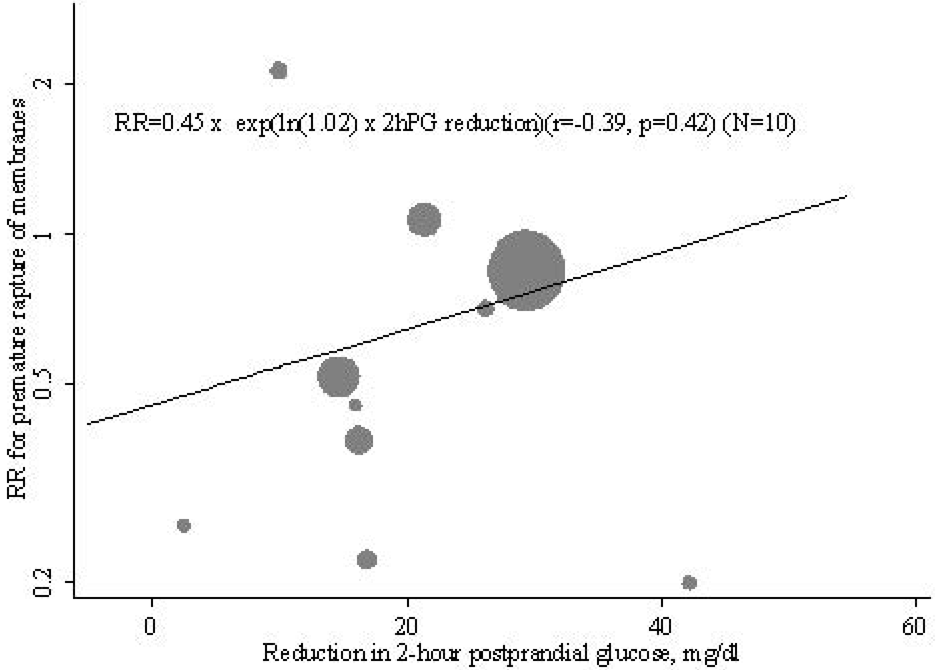

RR=0.45 x exp(ln(1.02) x 2hPG reduction)(r=-0.39, p=0.42) (N=10)
RR for premature rupture of membranes
2
1
0.5
0.2
0
20
40
60
Reduction in 2-hour postprandial glucose, mg/dl

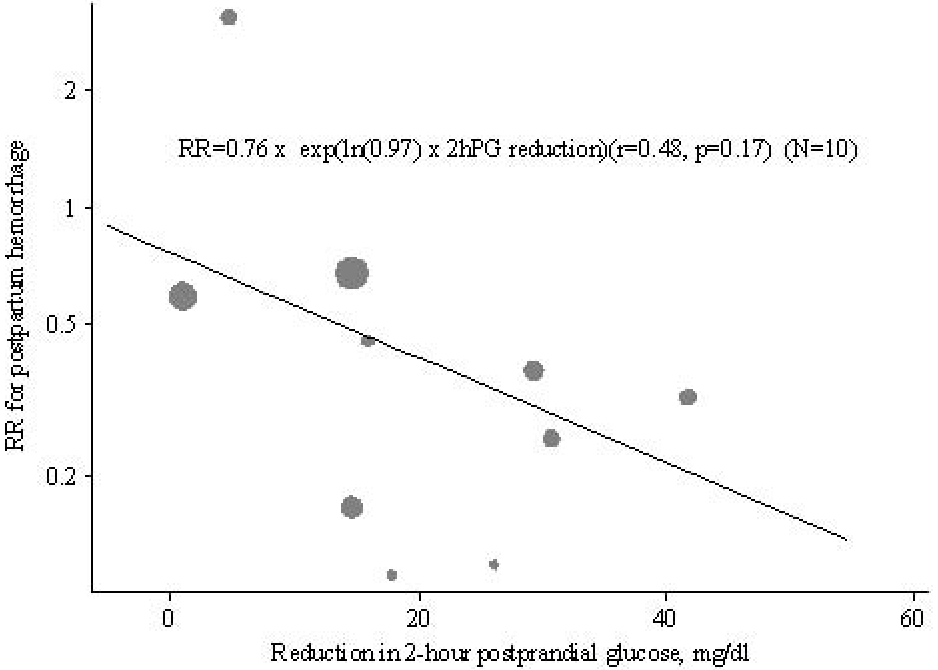

RR=0.76 x exp(ln(0.97) x 2hPG reduction)(r=0.48, p=0.17) (N=10)
RR for postpartum hemorrhage
2
1
0.5
0.2
0
20
40
60
Reduction in 2-hour postprandial glucose, mg/dl

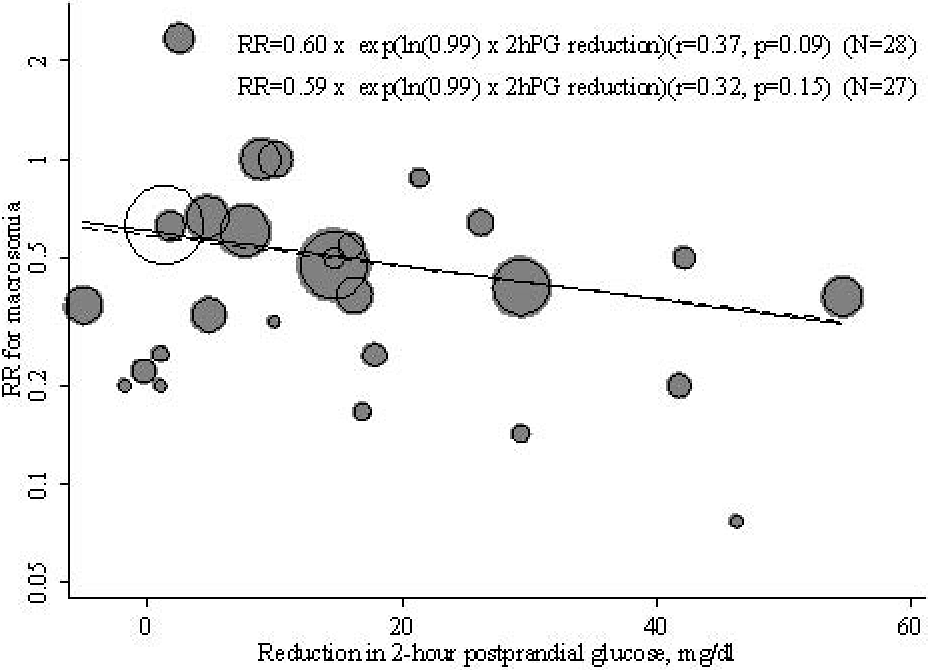

RR=0.60 x exp(ln(0.99) x 2hPG reduction)(r=0.37, p=0.09) (N=28)
RR=0.59 x exp(ln(0.99) x 2hPG reduction)(r=0.32, p=0.15) (N=27)
RR for macrosomia
2
1
0.5
0.2
0.1
0.05
0
20
40
60
Reduction in 2-hour postprandial glucose, mg/dl

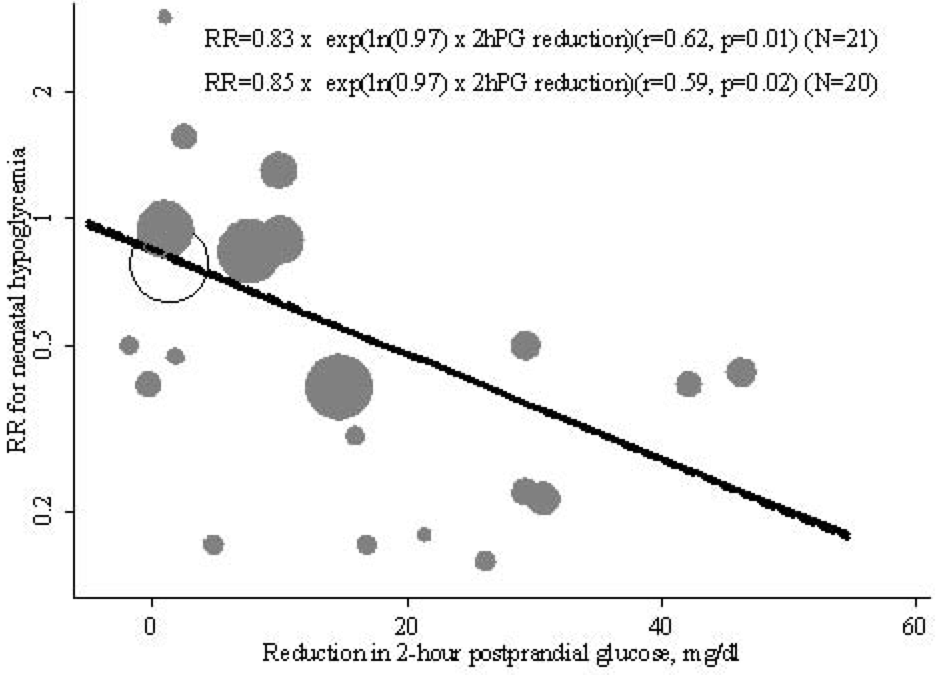
RR=0.83 x exp(ln(0.97) x 2hPG reduction)(r=0.62, p=0.01) (N=21)
RR=0.85 x exp(ln(0.97) x 2hPG reduction)(r=0.59, p=0.02) (N=20)
RR for neonatal hypoglycemia
2
1
0.5
0.2
0
20
40
60
Reduction in 2-hour postprandial glucose, mg/dl

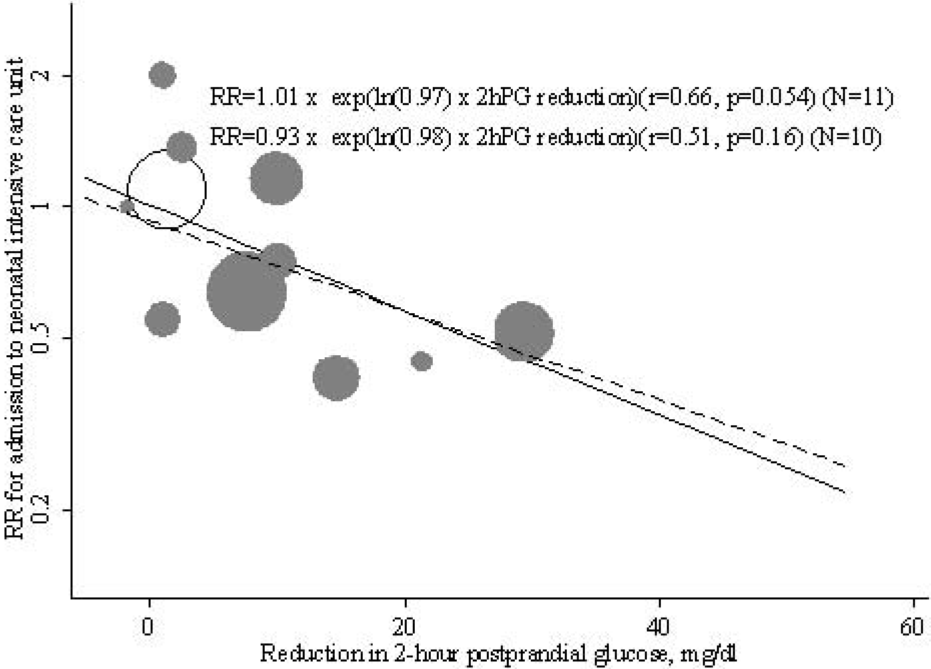

RR=1.01 x exp(ln(0.97) x 2hPG reduction)(r=0.66, p=0.054) (N=11)
RR=0.93 x exp(ln(0.98) x 2hPG reduction)(r=0.51, p=0.16) (N=10)
RR for admission to neonatal intensive care unit
2
1
0.5
0.2
0
20
40
60
Reduction in 2-hour postprandial glucose, mg/dl

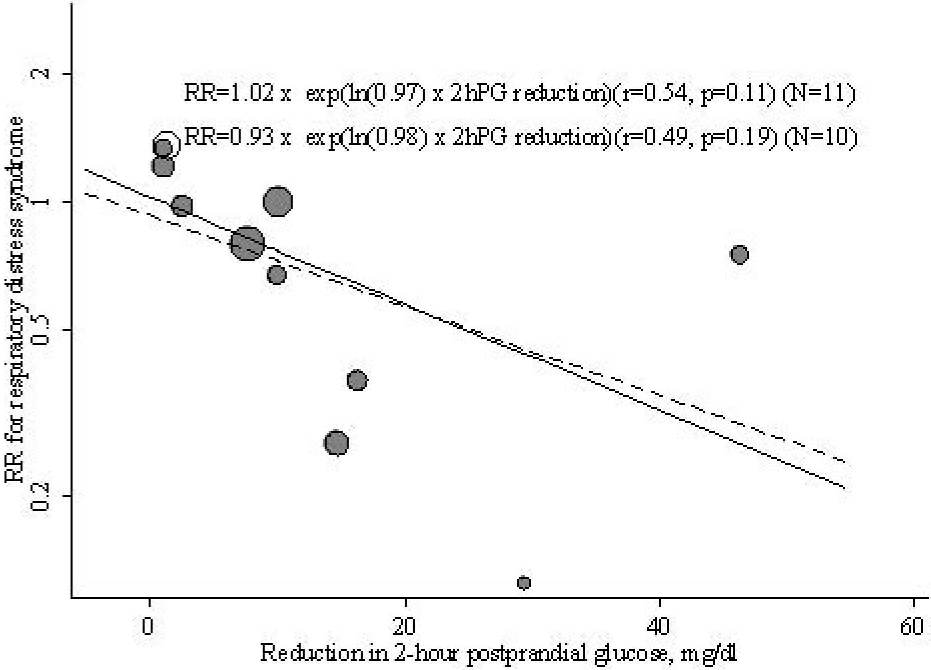
RR=1.02 x exp(ln(0.97) x 2hPG reduction)(r=0.54, p=0.11) (N=11)
RR=0.93 x exp(ln(0.98) x 2hPG reduction)(r=0.49, p=0.19) (N=10)
RR for respiratory distress syndrome
2
1
0.5
0.2
0
20
40
60
Reduction in 2-hour postprandial glucose, mg/dl

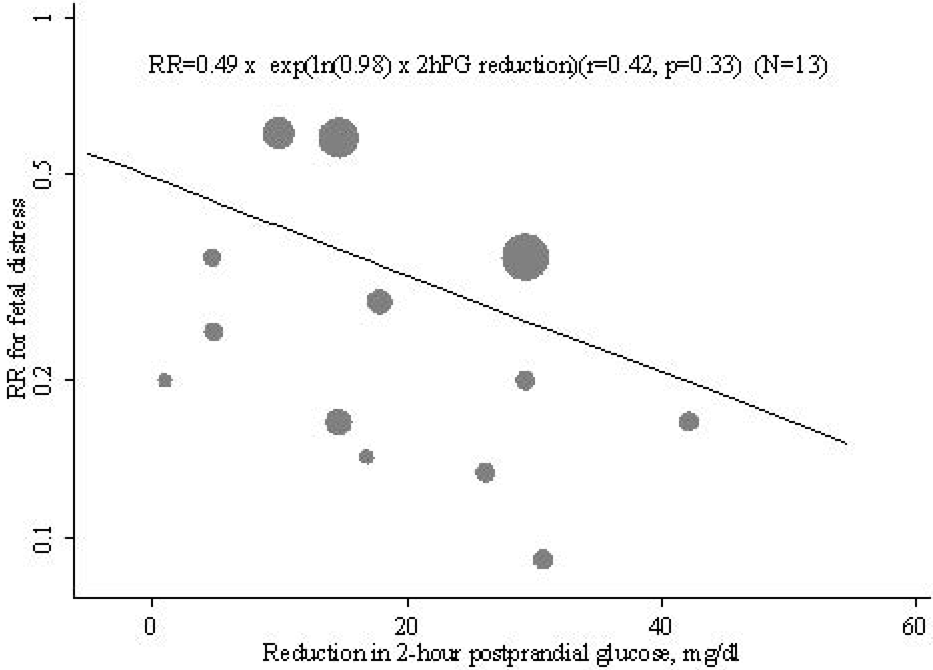
RR=0.49 x exp(ln(0.98) x 2hPG reduction)(r=0.42, p=0.33) (N=13)
RR for fetal distress
1
0.5
0.2
0.1
0
20
40
60
Reduction in 2-hour postprandial glucose, mg/dl

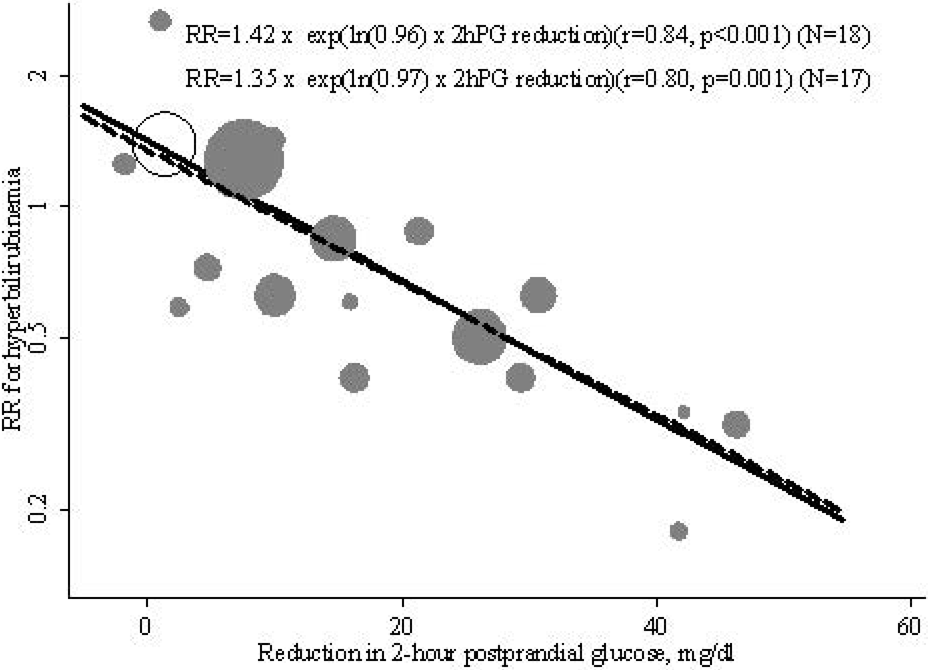
RR=1.42 x exp(ln(0.96) x 2hPG reduction)(r=0.84, p<0.001) (N=18)
RR=1.35 x exp(ln(0.97) x 2hPG reduction)(r=0.80, p=0.001) (N=17)
RR for hyperbilirubinemia
2
1
0.5
0.2
0
20
40
60
Reduction in 2-hour postprandial glucose, mg/dl

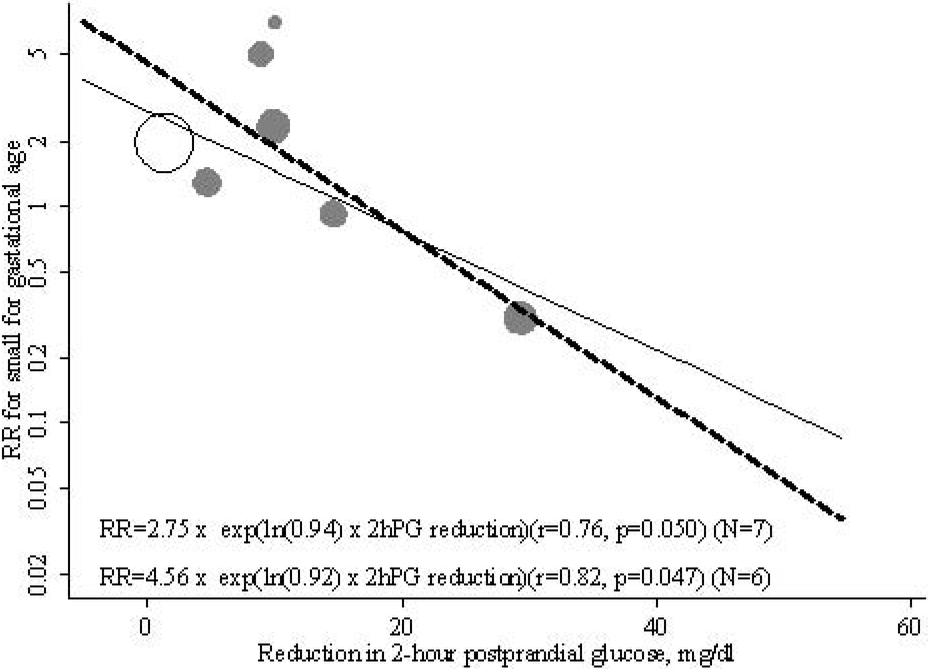
RR for small for gestational age
5
2
1
0.5
0.2
0.1
0.05
0.02
RR=2.75 x exp(ln(0.94) x 2hPG reduction)(r=0.76, p=0.050) (N=7)
RR=4.56 x exp(ln(0.92) x 2hPG reduction)(r=0.82, p=0.047) (N=6)
0
20
40
60
Reduction in 2-hour postprandial glucose, mg/dl

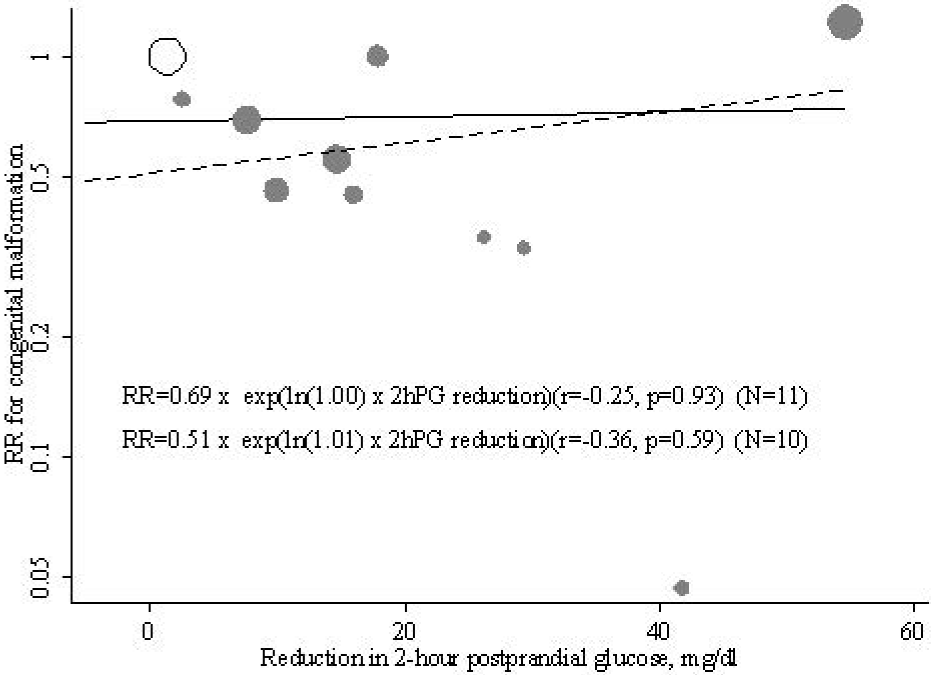
RR for congenital malformation
1
0.5
0.2
0.1
0.05
0
20
40
60
Reduction in 2-hour postprandial glucose, mg/dl
RR=0.69 x exp(ln(1.00) x 2hPG reduction)(r=-0.25, p=0.93) (N=11)
RR=0.51 x exp(ln(1.01) x 2hPG reduction)(r=-0.36, p=0.59) (N=10)

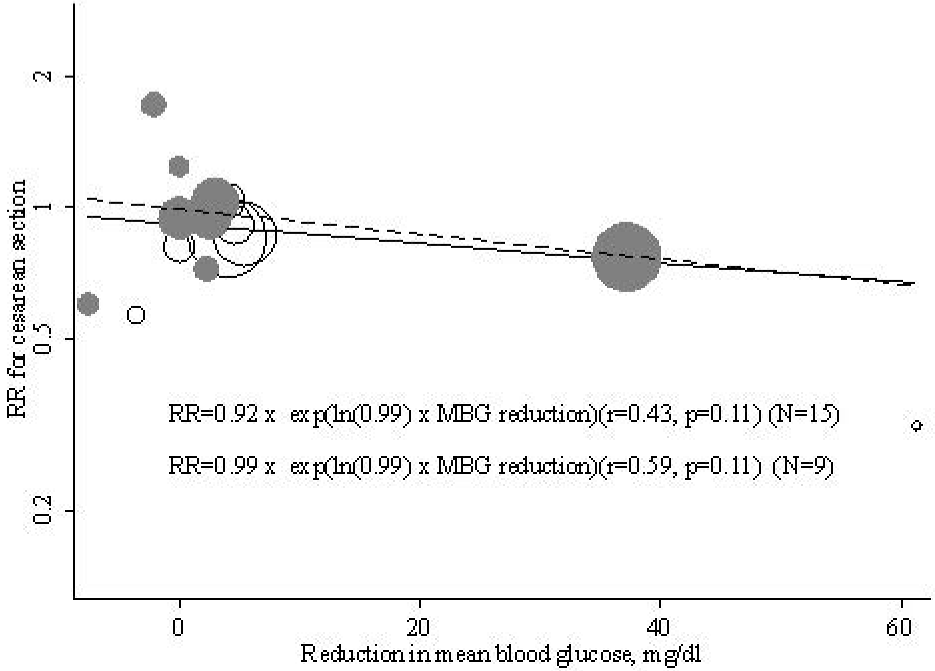

RR for cesarean section
2
1
0.5
0.2
RR=0.92 x exp(ln(0.99) x MBG reduction)(r=0.43, p=0.11) (N=15)
RR=0.99 x exp(ln(0.99) x MBG reduction)(r=0.59, p=0.11) (N=9)
0
20
40
60
Reduction in mean blood glucose, mg/dl

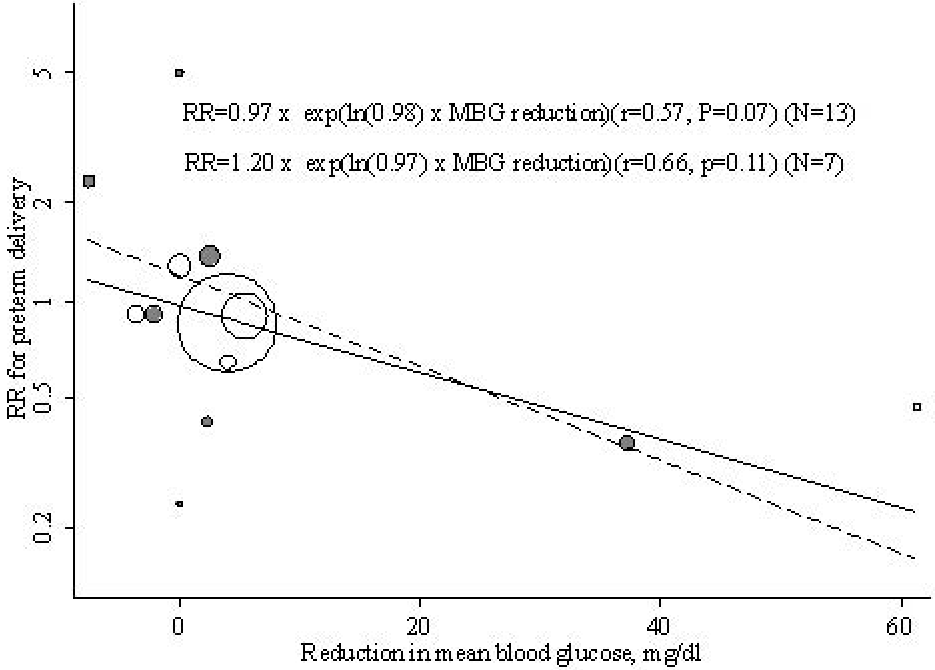
RR=0.97 x exp(ln(0.98) x MBG reduction)(r=0.57, P=0.07) (N=13)
RR=1.20 x exp(ln(0.97) x MBG reduction)(r=0.66, p=0.11) (N=7)
RR for preterm delivery
5
2
1
0.5
0.2
0
20
40
60
Reduction in mean blood glucose, mg/dl

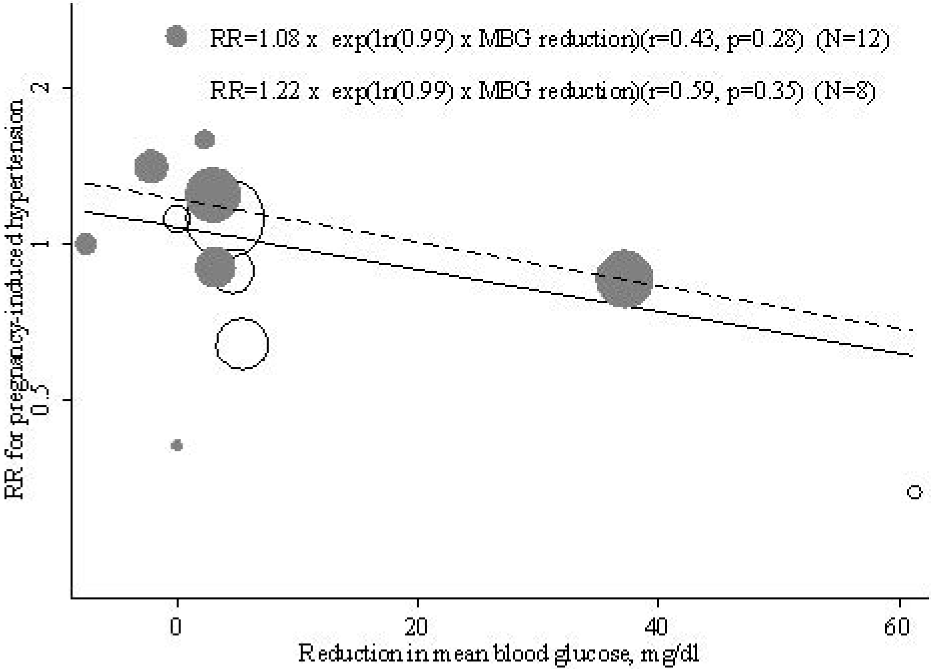
RR=1.08 x exp(ln(0.99) x MBG reduction)(r=0.43, p=0.28) (N=12)
RR=1.22 x exp(ln(0.99) x MBG reduction)(r=0.59, p=0.35) (N=8)
RR for pregnancy-induced hypertension
2
1
0.5
0
20
40
60
Reduction in mean blood glucose, mg/dl

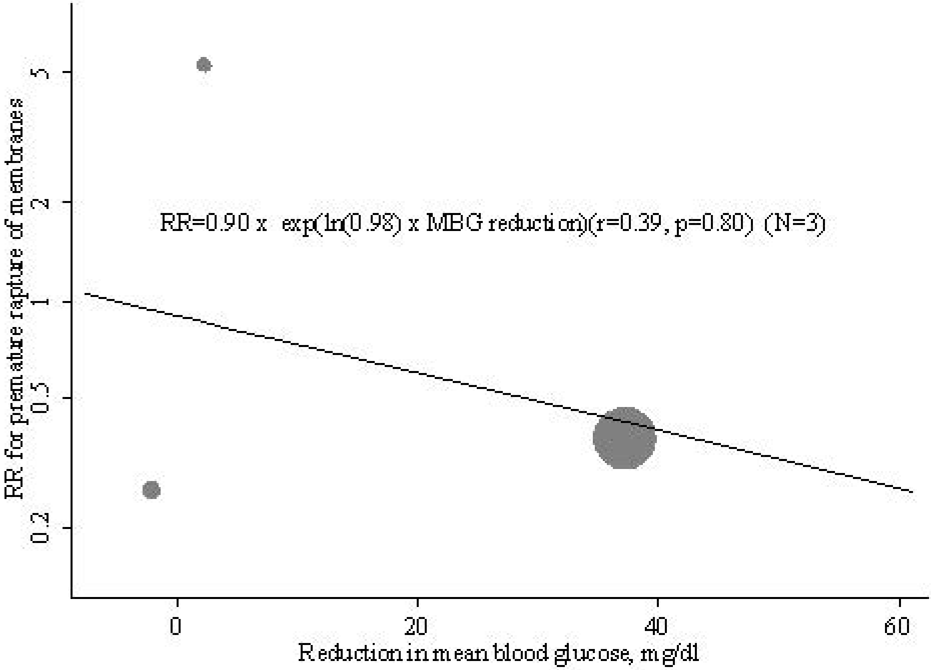

RR=0.90 x exp(ln(0.98) x MBG reduction)(r=0.39, p=0.80) (N=3)
RR for premature rapture of membranes
0.2
0.5
1
2
5
0
20
40
60
Reduction in mean blood glucose, mg/dl

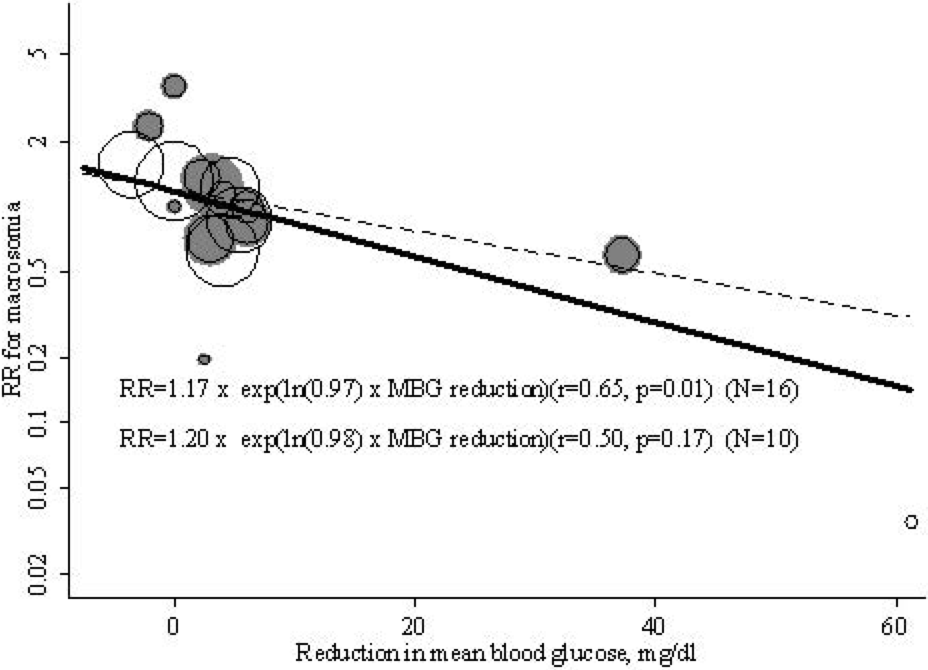
RR for macrosomia
5
2
0.5
0.2
0.1
0.05
0.02
RR=1.17 x exp(ln(0.97) x MBG reduction)(r=0.65, p=0.01) (N=16)
RR=1.20 x exp(ln(0.98) x MBG reduction)(r=0.50, p=0.17) (N=10)
0
20
40
60
Reduction in mean blood glucose, mg/dl

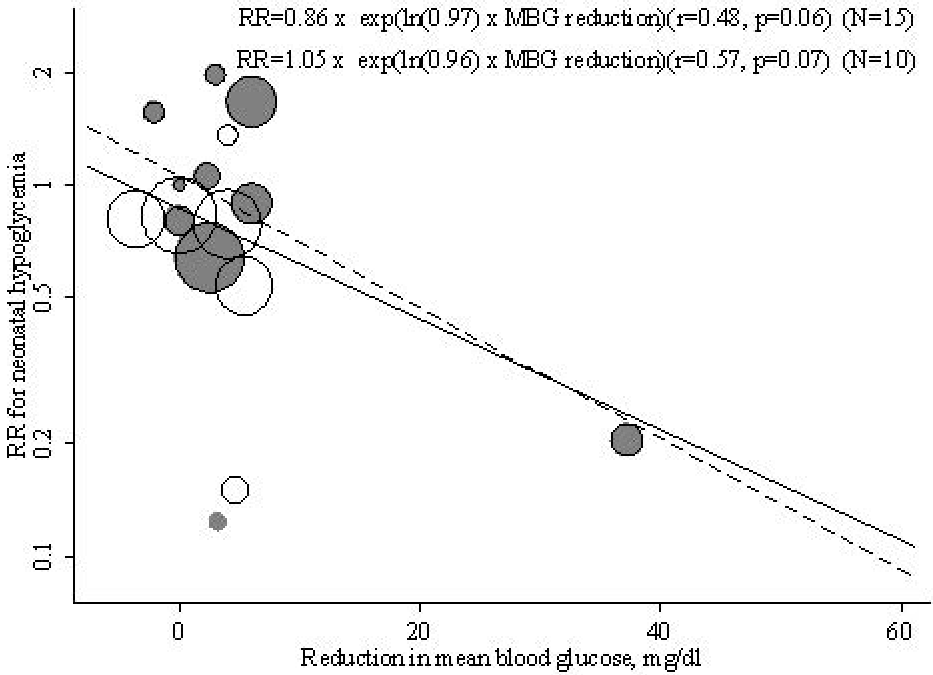

RR=0.86 x exp(ln(0.97) x MBG reduction)(r=0.48, p=0.06) (N=15)
RR=1.05 x exp(ln(0.96) x MBG reduction)(r=0.57, p=0.07) (N=10)
RR for neonatal hypoglycemia
2
1
0.5
0.2
0.1
0
20
40
60
Reduction in mean blood glucose, mg/dl

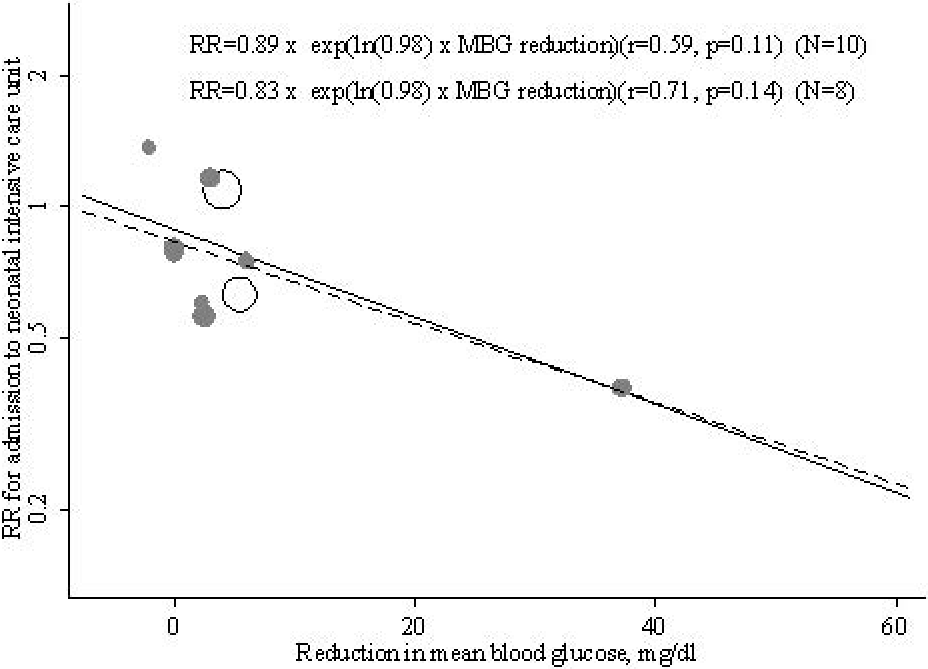

RR=0.89 x exp(ln(0.98) x MBG reduction)(r=0.59, p=0.11) (N=10)
RR=0.83 x exp(ln(0.98) x MBG reduction)(r=0.71, p=0.14) (N=8)
RR for admission to neonatal intensive care unit
2
1
0.5
0.2
0
20
40
60
Reduction in mean blood glucose, mg/dl

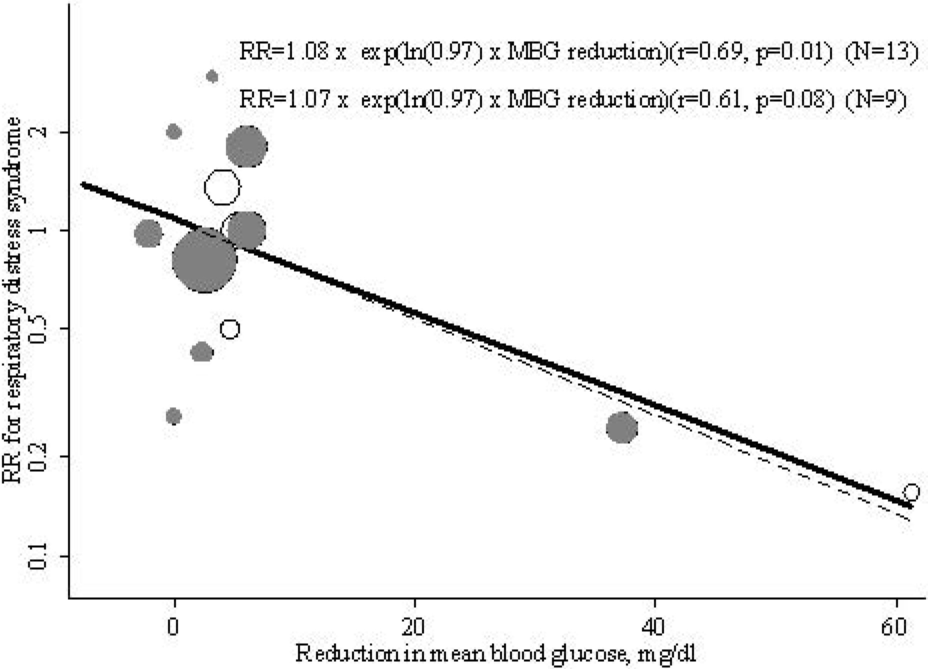
RR=1.08 x exp(ln(0.97) x MBG reduction)(r=0.69, p=0.01) (N=13)
RR=1.07 x exp(ln(0.97) x MBG reduction)(r=0.61, p=0.08) (N=9)
RR for respiratory distress syndrome
2
1
0.5
0.2
0.1
0
20
40
60
Reduction in mean blood glucose, mg/dl

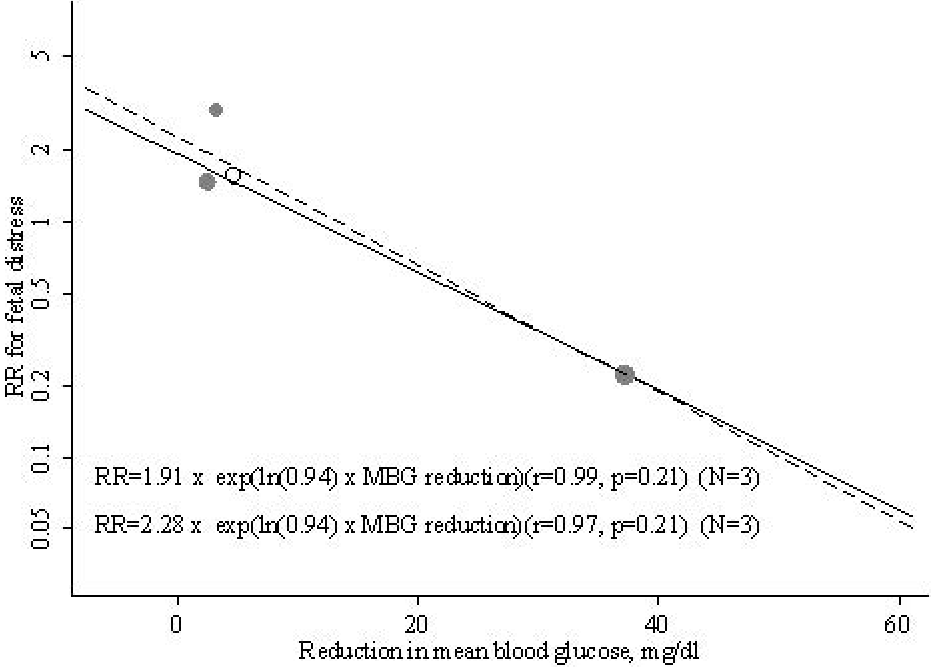

RR for fetal distress
5
2
1
0.5
0.2
0.1
0.05
RR=1.91 x exp(ln(0.94) x MBG reduction)(r=0.99, p=0.21) (N=3)
RR=2.28 x exp(ln(0.94) x MBG reduction)(r=0.97, p=0.21) (N=3)
0
20
40
60
Reduction in mean blood glucose, mg/dl

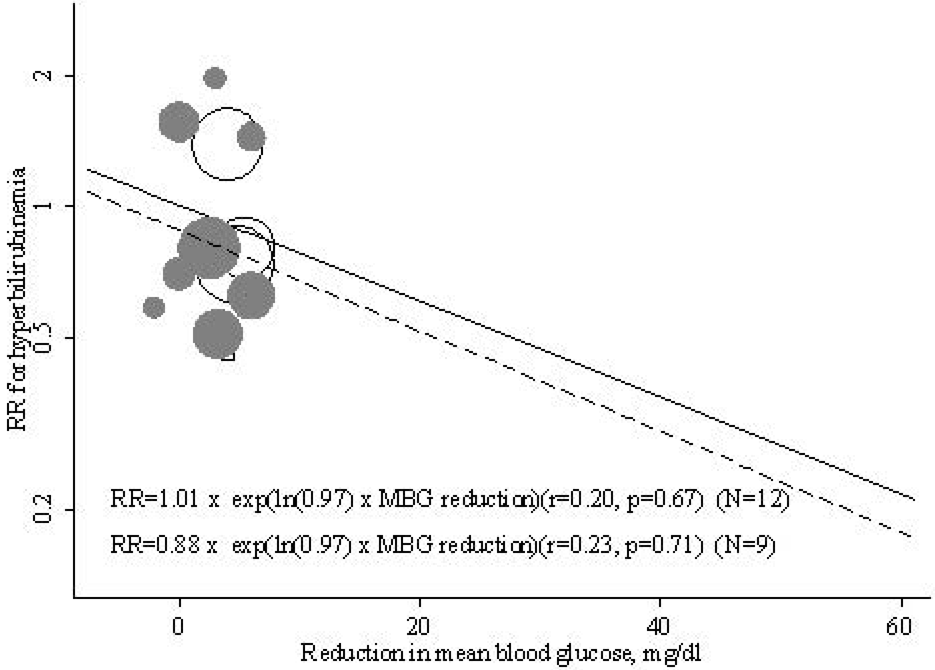
RR for hyperbilirubinemia
2
1
0.5
0.2
0
20
40
60
Reduction in mean blood glucose, mg/dl
RR=1.01 x exp(ln(0.97) x MBG reduction)(r=0.20, p=0.67) (N=12)
RR=0.88 x exp(ln(0.97) x MBG reduction)(r=0.23, p=0.71) (N=9)

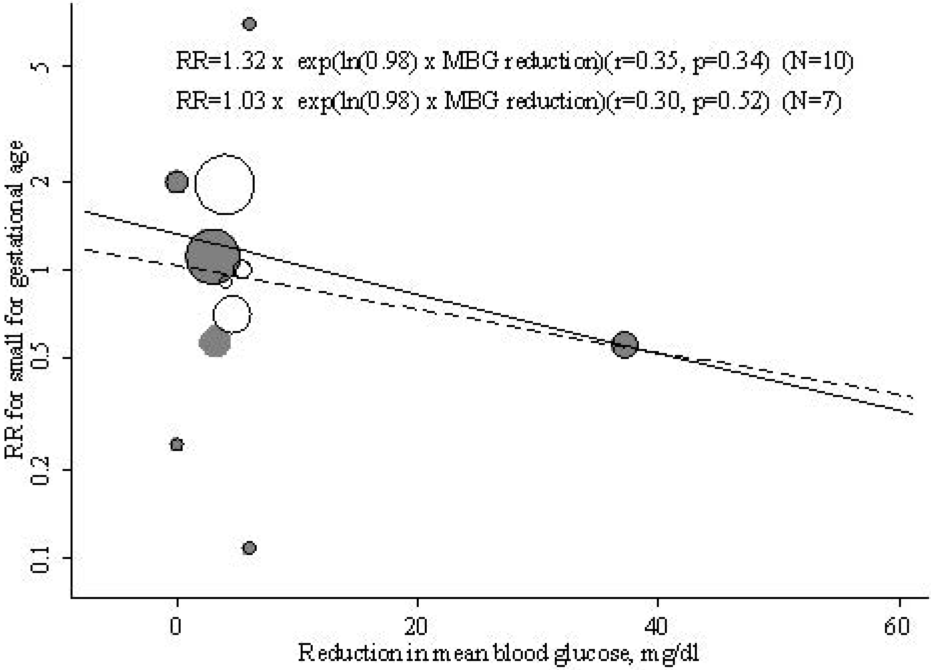
RR=1.32 x exp(ln(0.98) x MBG reduction)(r=0.35, p=0.34) (N=10)
RR=1.03 x exp(ln(0.98) x MBG reduction)(r=0.30, p=0.52) (N=7)
RR for small for gestational age
5
2
1
0.5
0.2
0.1
0
20
40
60
Reduction in mean blood glucose, mg/dl

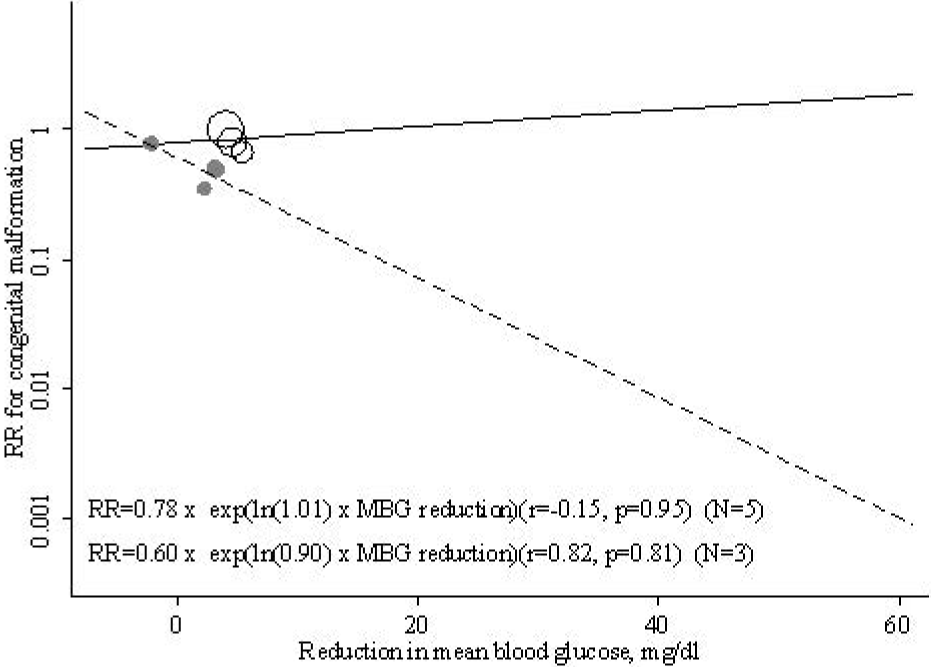

RR for congenital malformation
1
0.1
0.01
0.001
RR=0.78 x exp(ln(1.01) x MBG reduction)(r=-0.15, p=0.95) (N=5)
RR=0.60 x exp(ln(0.90) x MBG reduction)(r=0.82, p=0.81) (N=3)
0
20
40
60
Reduction in mean blood glucose, mg/dl
