# Supplementary material for: Relationship Between Improvements in Glycemic Control and Risk of Pregnancy Complications in Patients With Diabetes Mellitus: Metaregression Analysis of Randomized Controlled Trials of Intensive Glucose Management
Source: J Diabetes Res. 2025 Jun 23;2025:3490884. doi: 10.1155/jdr/3490884 (PMC12208766; doi:10.1155/jdr/3490884)
Supplement: Supporting Information 4 — Scatterplots of reductions in each glycemic indicator and relative risks (RRs) for six adverse pregnancy outcomes. Solid regression lines correspond to scatterplots for trials conducted in Asia or the Middle East, and dashed regression lines correspond to scatterplots for trials conducted in countries other than in Asia and the Middle East. Size of circles is proportional to study weight (i.e., inverse of variance of logarithm of RR). White circles indicate trials performed in Asia or the Middle East, and gray circles indicate trials performed in countries other than in Asia and the Middle East. Upper and lower regression formulas are determined when trials conducted for Asian and non-Asian countries, respectively, were analyzed. Panels that show only one regression line and formula are those for which regression lines could not be drawn for trials conducted in non-Asian countries because of an insufficient number of data (at least three data are necessary). Abbreviations: 2hPG, 2-h postprandial glucose; A1C, hemoglobin A1c; FPG, fasting plasma glucose; MBG, mean blood glucose. [file 3490884.f4.pdf]

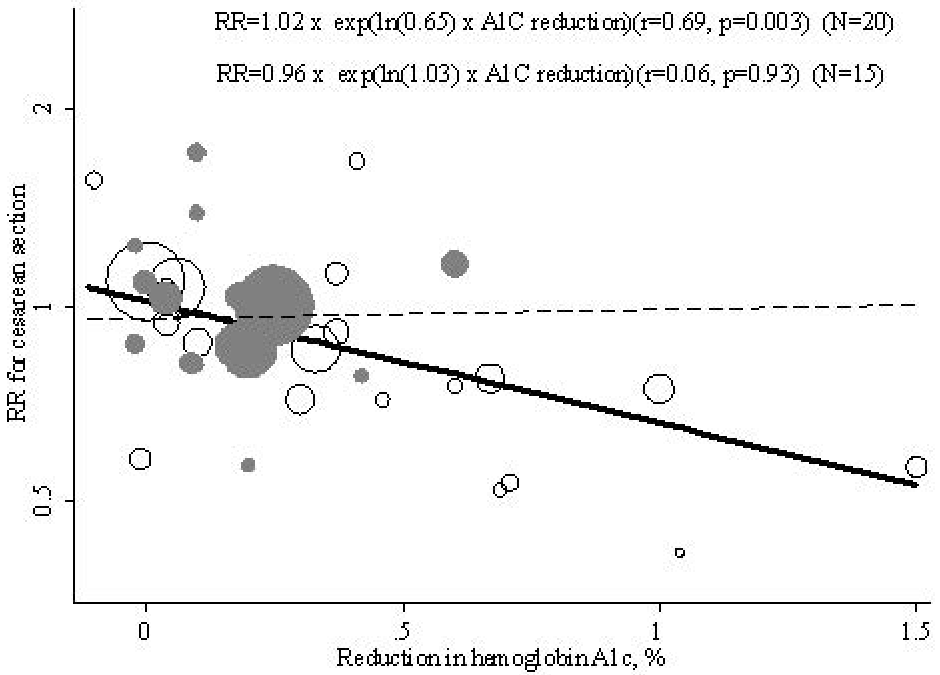
RR=1.02 x exp(ln(0.65) x A1C reduction)(r=0.69, p=0.003) (N=20)
RR=0.96 x exp(ln(1.03) x A1C reduction)(r=0.06, p=0.93) (N=15)
RR for cesarean section
2
1
0.5
0
.5
1
1.5
Reduction in hemoglobin A1c, %

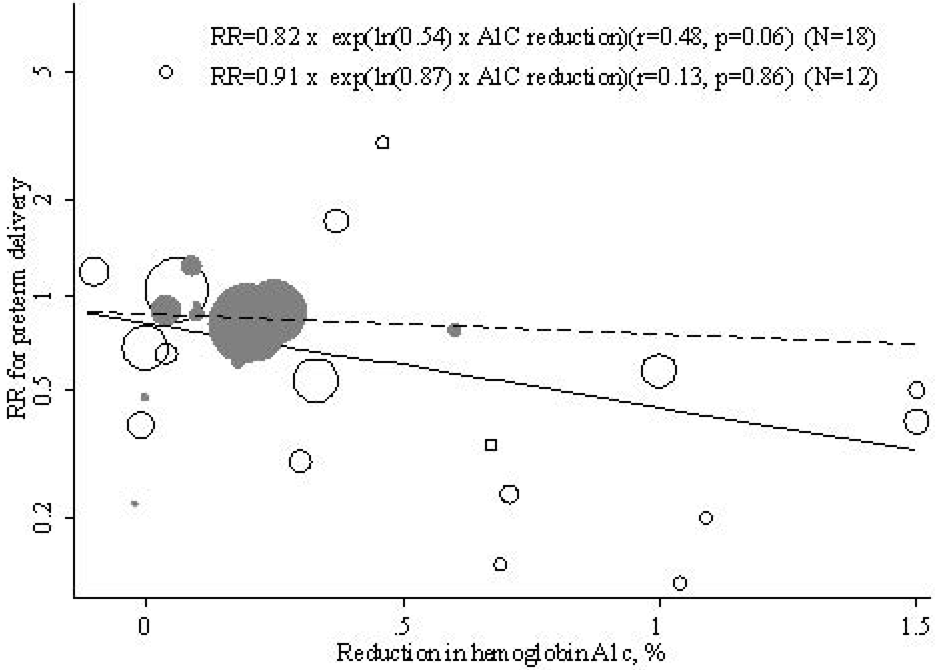

RR=0.82 x exp(ln(0.54) x A1C reduction)(r=0.48, p=0.06) (N=18)
RR=0.91 x exp(ln(0.87) x A1C reduction)(r=0.13, p=0.86) (N=12)
RR for preterm delivery
5
2
1
0.5
0.2
0
.5
1
1.5
Reduction in hemoglobin A1c, %

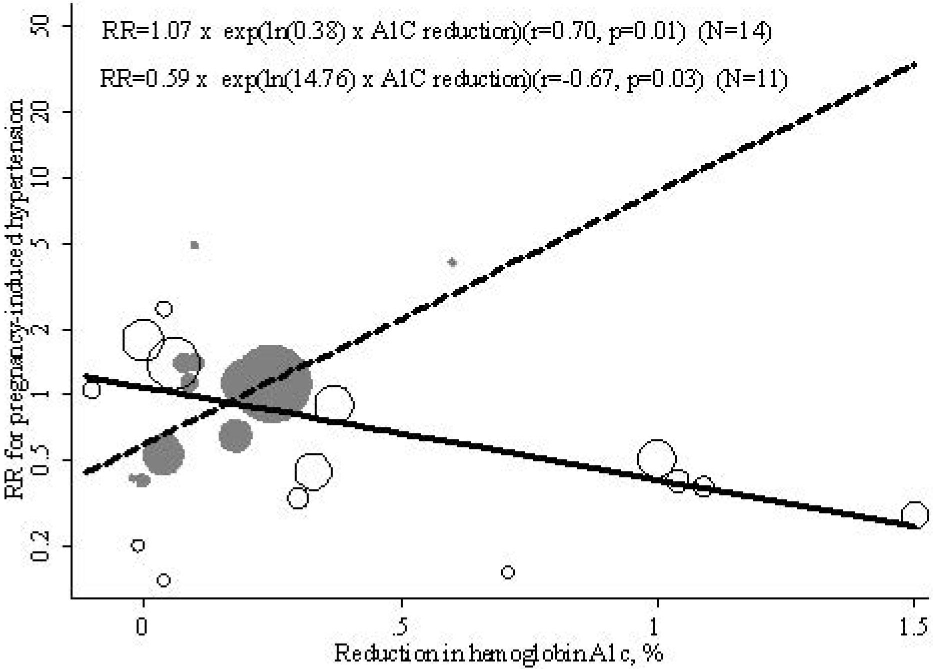
RR=1.07 x exp(ln(0.38) x A1C reduction)(r=0.70, p=0.01) (N=14)
RR=0.59 x exp(ln(14.76) x A1C reduction)(r=-0.67, p=0.03) (N=11)
RR for pregnancy-induced hypertension
50
20
10
5
2
1
0.5
0.2
0
.5
1
1.5
Reduction in hemoglobin A1c, %

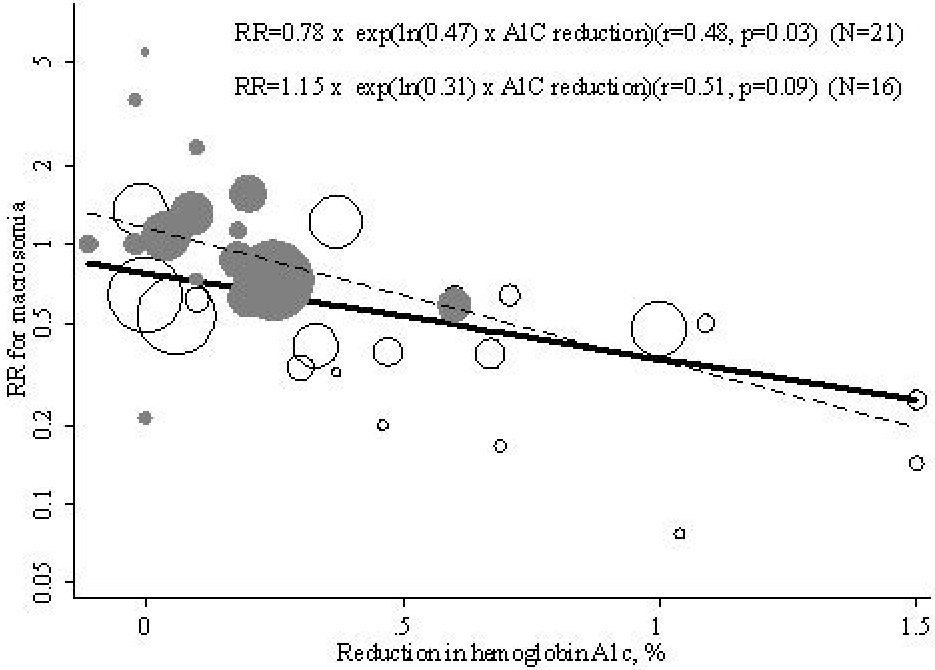

RR=0.78 x exp(ln(0.47) x A1C reduction)(r=0.48, p=0.03) (N=21)
RR=1.15 x exp(ln(0.31) x A1C reduction)(r=0.51, p=0.09) (N=16)
RR for macrosomia
5
2
1
0.5
0.2
0.1
0.05
0
.5
1
1.5
Reduction in hemoglobin A1c, %

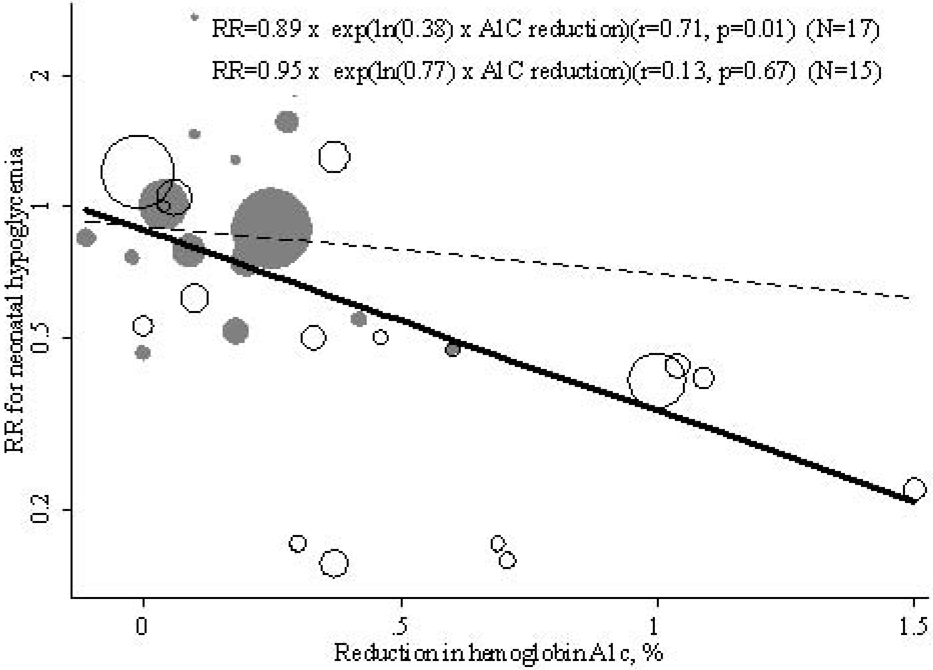
RR=0.89 x exp(ln(0.38) x A1C reduction)(r=0.71, p=0.01) (N=17)
RR=0.95 x exp(ln(0.77) x A1C reduction)(r=0.13, p=0.67) (N=15)
RR for neonatal hypoglycemia
2
1
0.5
0.2
0
.5
1
1.5
Reduction in hemoglobin A1c, %

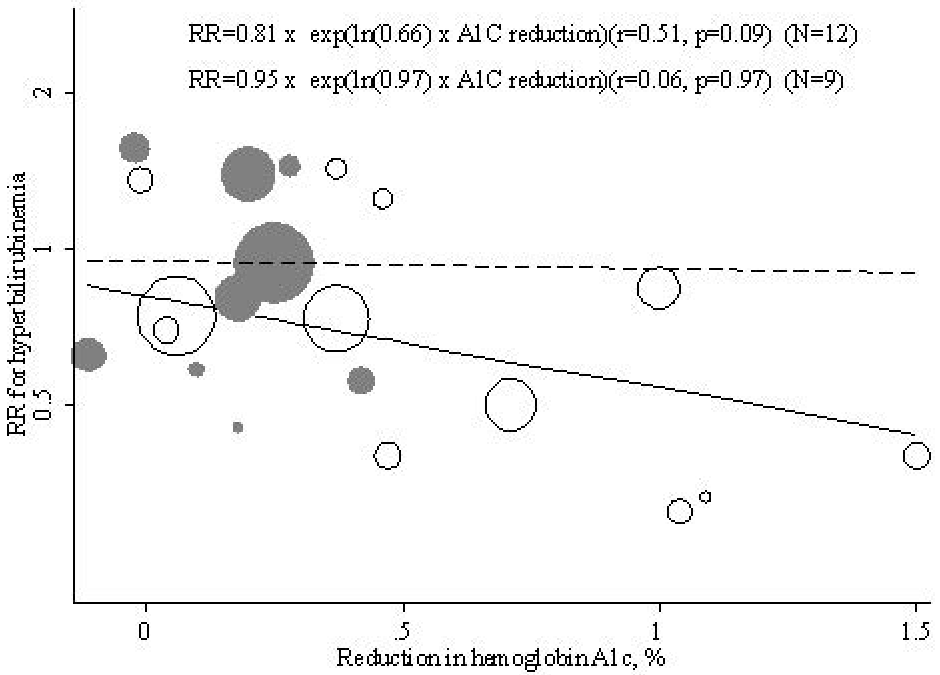
RR=0.81 x exp(ln(0.66) x A1C reduction)(r=0.51, p=0.09) (N=12)
RR=0.95 x exp(ln(0.97) x A1C reduction)(r=0.06, p=0.97) (N=9)
RR for hyperbilirubinemia
2
1
0.5
0
.5
1
1.5
Reduction in hemoglobin A1c, %

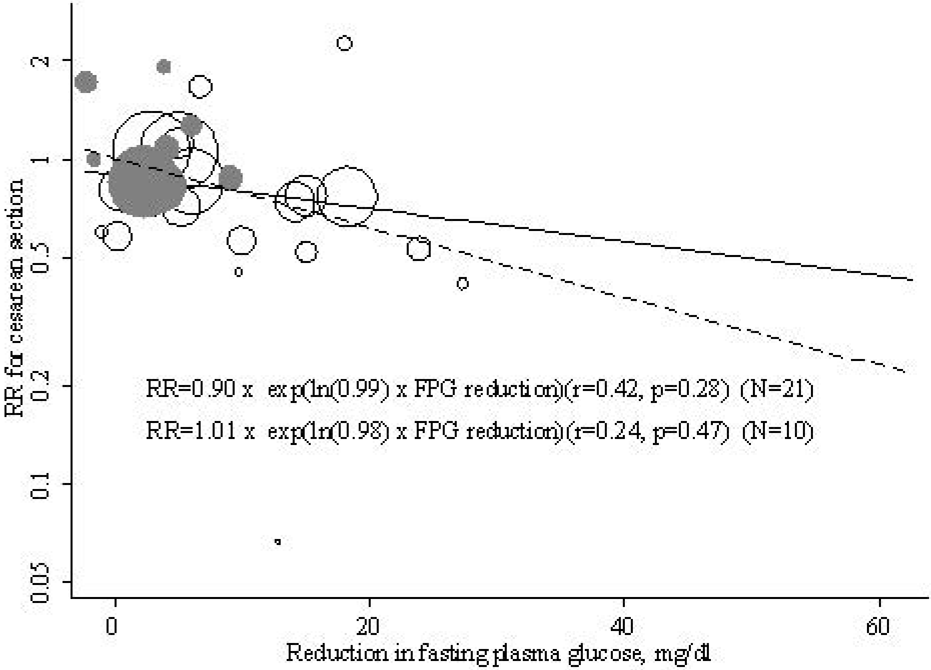

RR for cesarean section
2
1
0.5
0.2
0.1
0.05
RR=0.90 x exp(ln(0.99) x FPG reduction)(r=0.42, p=0.28) (N=21)
RR=1.01 x exp(ln(0.98) x FPG reduction)(r=0.24, p=0.47) (N=10)
0
20
40
60
Reduction in fasting plasma glucose, mg/dl

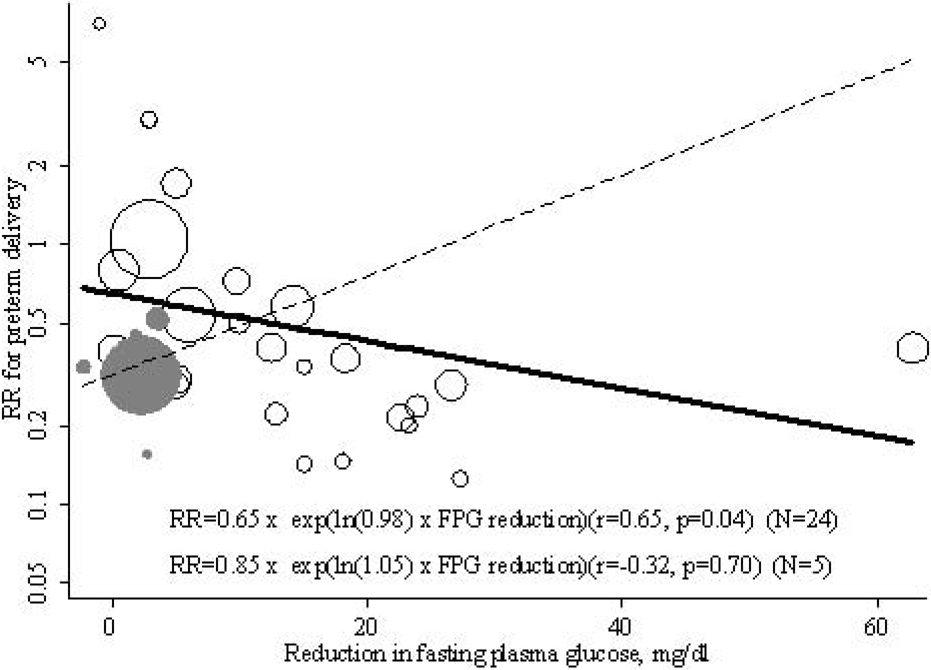
RR for preterm delivery
0.05
0.1
0.2
0.5
1
2
5
0
20
40
60
Reduction in fasting plasma glucose, mg/dl
RR=0.65 x exp(ln(0.98) x FPG reduction)(r=0.65, p=0.04) (N=24)
RR=0.85 x exp(ln(1.05) x FPG reduction)(r=-0.32, p=0.70) (N=5)

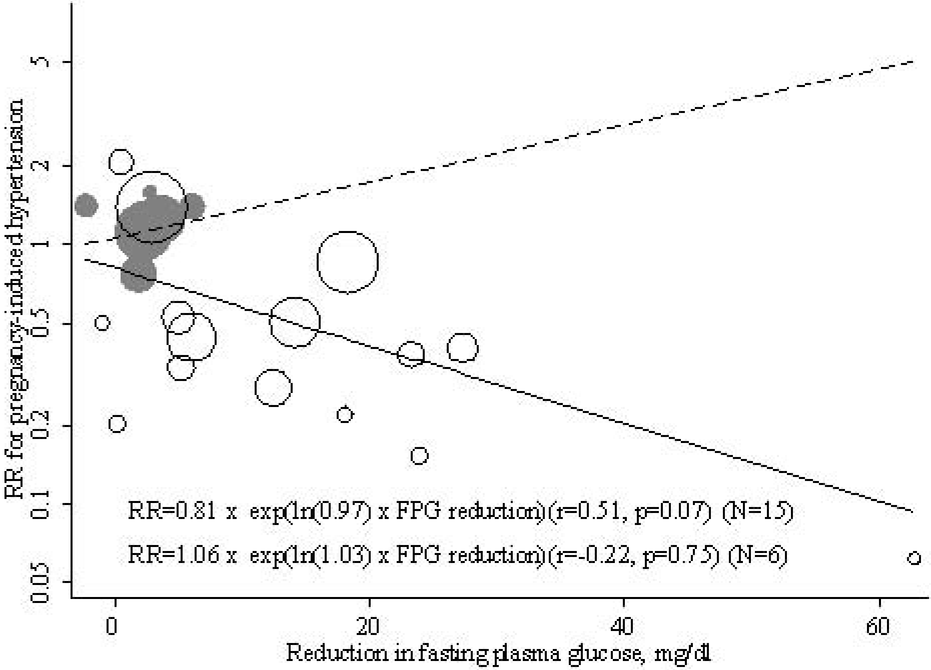

RR for pregnancy-induced hypertension
5
2
1
0.5
0.2
0.1
0.05
RR=0.81 x exp(ln(0.97) x FPG reduction)(r=0.51, p=0.07) (N=15)
RR=1.06 x exp(ln(1.03) x FPG reduction)(r=-0.22, p=0.75) (N=6)
0
20
40
60
Reduction in fasting plasma glucose, mg/dl

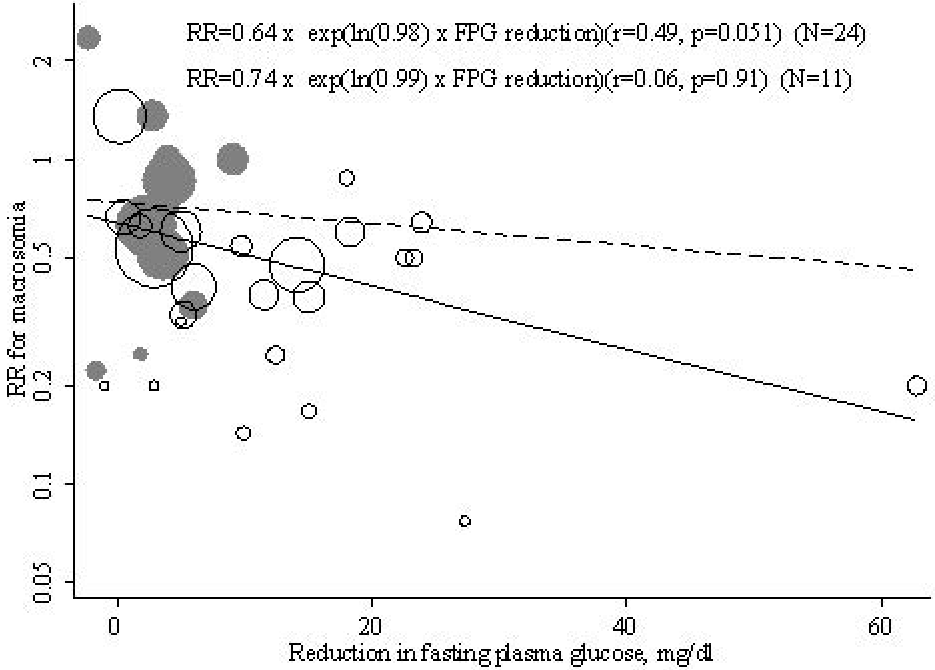
RR=0.64 x exp(ln(0.98) x FPG reduction)(r=0.49, p=0.051) (N=24)
RR=0.74 x exp(ln(0.99) x FPG reduction)(r=0.06, p=0.91) (N=11)
RR for macrosomia
2
1
0.5
0.2
0.1
0.05
0
20
40
60
Reduction in fasting plasma glucose, mg/dl

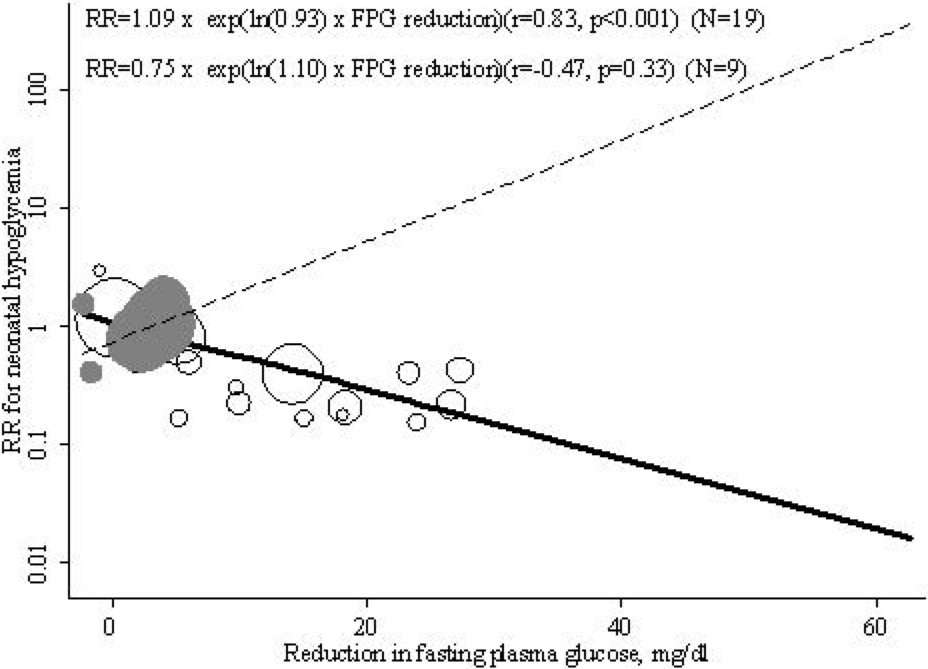

RR=1.09 x exp(ln(0.93) x FPG reduction)(r=0.83, p<0.001) (N=19)
RR=0.75 x exp(ln(1.10) x FPG reduction)(r=-0.47, p=0.33) (N=9)
RR for neonatal hypoglycemia
100
10
1
0.1
0.01
0
20
40
60
Reduction in fasting plasma glucose, mg/dl

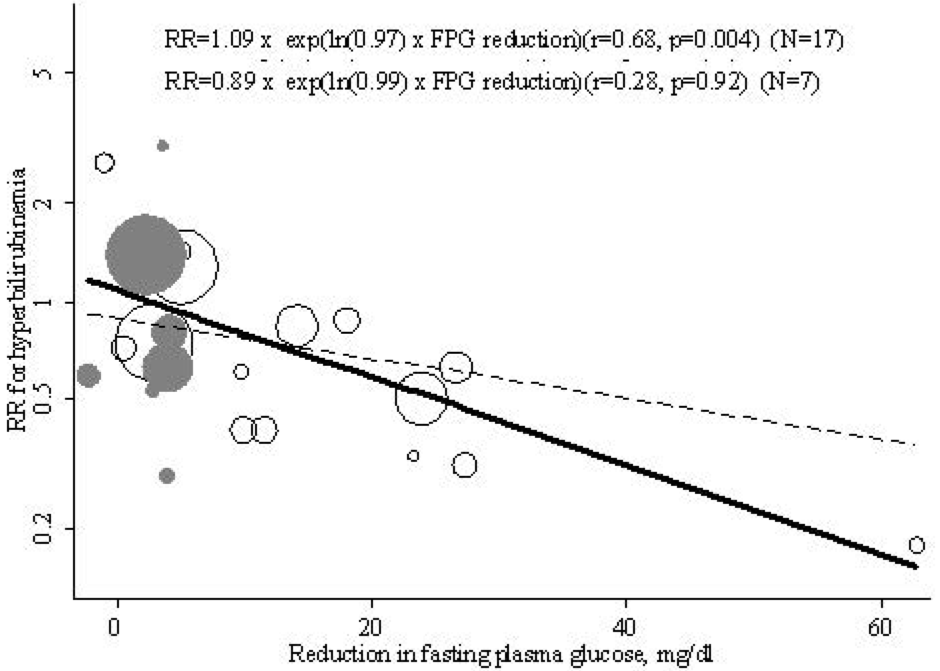
RR=1.09 x exp(ln(0.97) x FPG reduction)(r=0.68, p=0.004) (N=17)
RR=0.89 x exp(ln(0.99) x FPG reduction)(r=0.28, p=0.92) (N=7)
RR for hyperbilirubinemia
5
2
1
0.5
0.2
0
20
40
60
Reduction in fasting plasma glucose, mg/dl

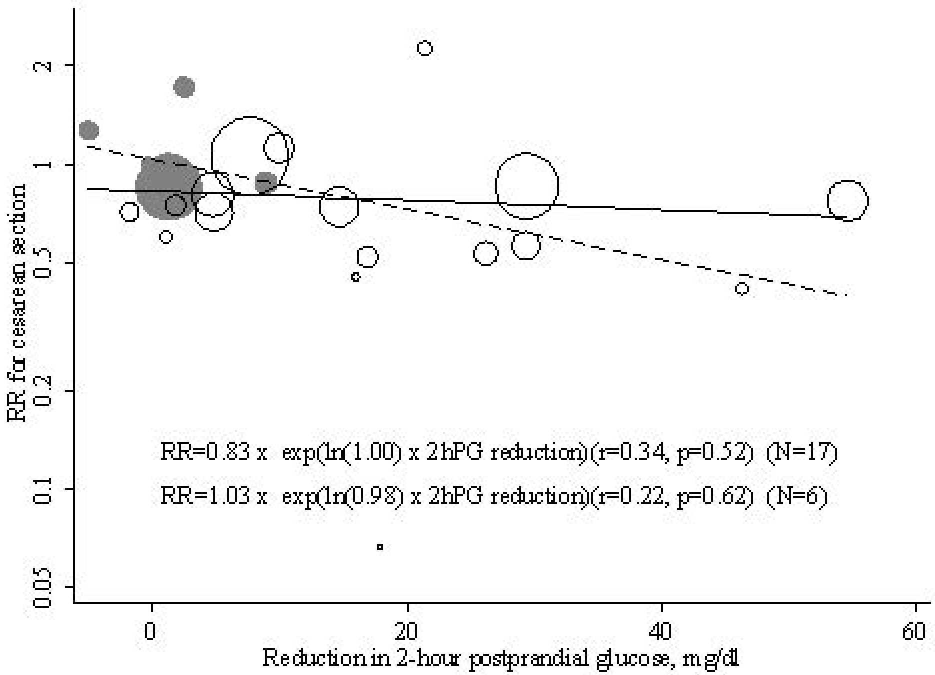

RR for cesarean section
2
1
0.5
0.2
0.1
0.05
0
20
40
60
Reduction in 2-hour postprandial glucose, mg/dl
RR=0.83 x exp(ln(1.00) x 2hPG reduction)(r=0.34, p=0.52) (N=17)
RR=1.03 x exp(ln(0.98) x 2hPG reduction)(r=0.22, p=0.62) (N=6)

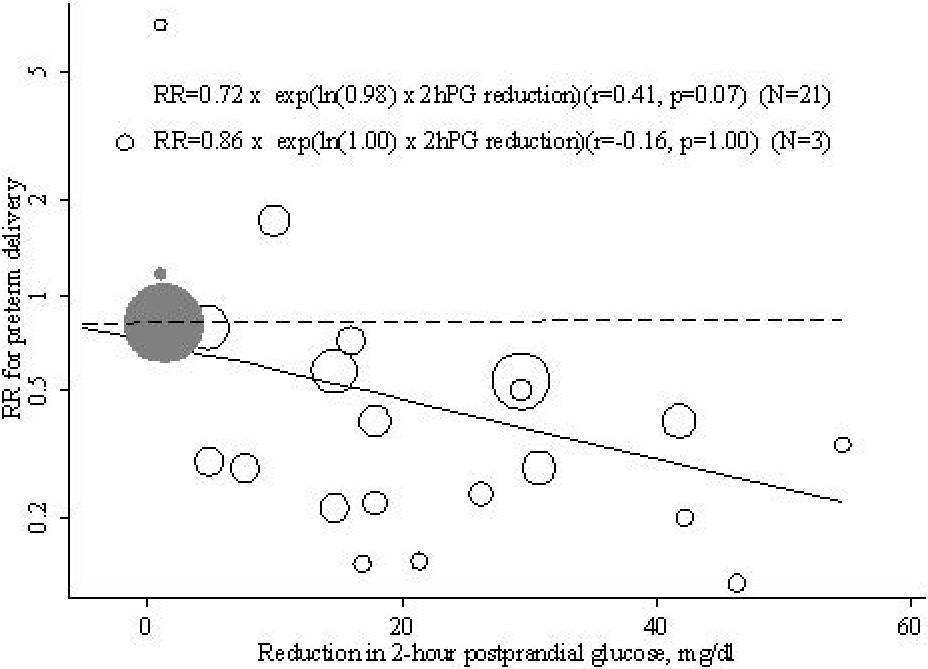

RR=0.72 x exp(ln(0.98) x 2hPG reduction)(r=0.41, p=0.07) (N=21)
RR=0.86 x exp(ln(1.00) x 2hPG reduction)(r=-0.16, p=1.00) (N=3)
RR for preterm delivery
5
2
1
0.5
0.2
0
20
40
60
Reduction in 2-hour postprandial glucose, mg/dl

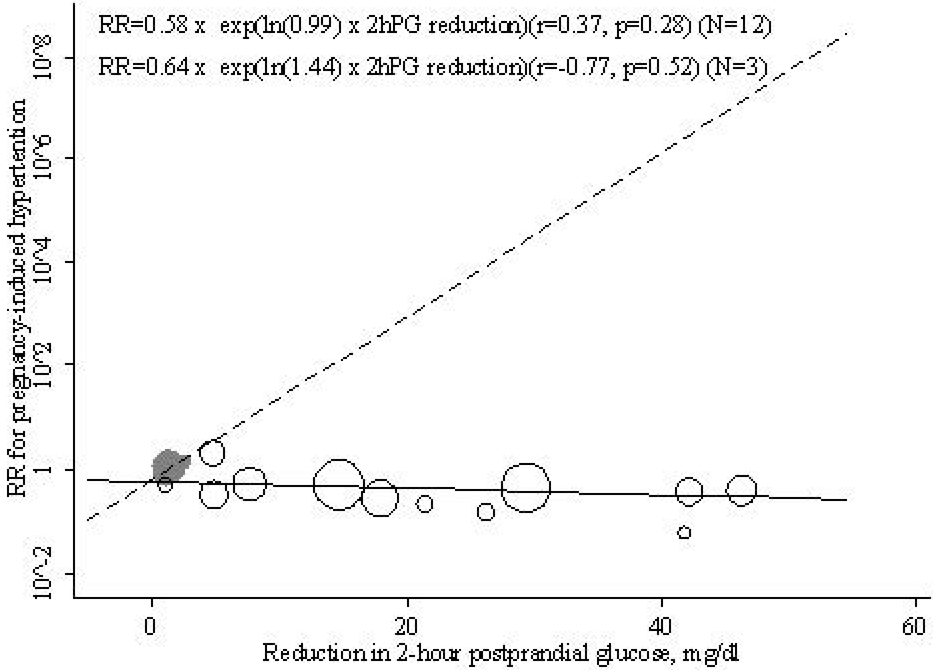
RR=0.58 x exp(ln(0.99) x 2hPG reduction)(r=0.37, p=0.28) (N=12)
RR=0.64 x exp(ln(1.44) x 2hPG reduction)(r=-0.77, p=0.52) (N=3)
RR for pregnancy-induced hypertention
10^8
10^6
10^4
10^2
1
10^-2
0
20
40
60
Reduction in 2-hour postprandial glucose, mg/dl

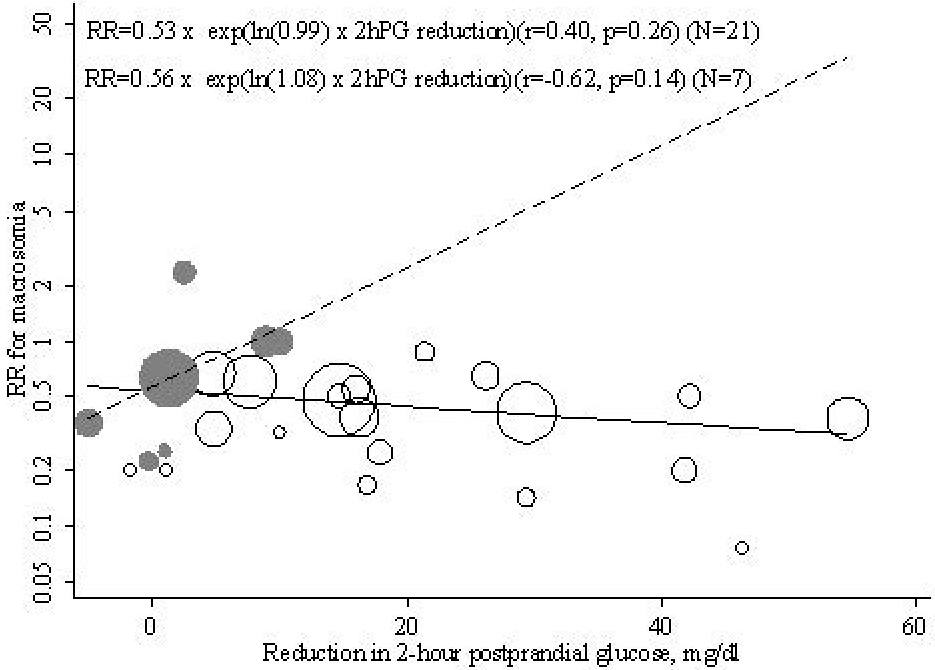

RR=0.53 x exp(ln(0.99) x 2hPG reduction)(r=0.40, p=0.26) (N=21)
RR=0.56 x exp(ln(1.08) x 2hPG reduction)(r=-0.62, p=0.14) (N=7)
RR for macrosomia
50
20
10
5
2
1
0.5
0.2
0.1
0.05
0
20
40
60
Reduction in 2-hour postprandial glucose, mg/dl

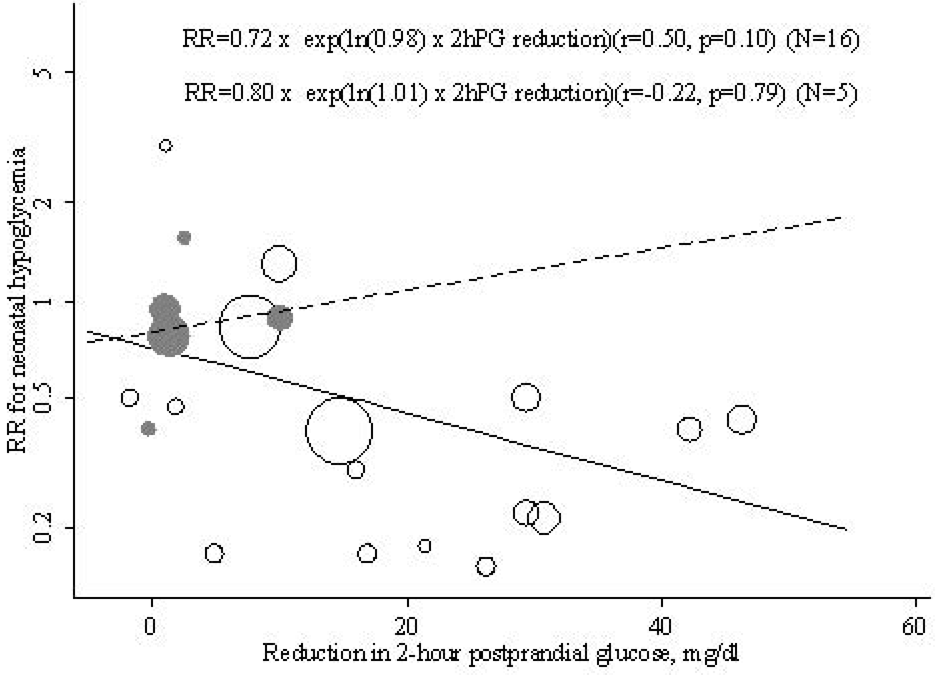
RR=0.72 x exp(ln(0.98) x 2hPG reduction)(r=0.50, p=0.10) (N=16)
RR=0.80 x exp(ln(1.01) x 2hPG reduction)(r=-0.22, p=0.79) (N=5)
RR for neonatal hypoglycemia
5
2
1
0.5
0.2
0
20
40
60
Reduction in 2-hour postprandial glucose, mg/dl

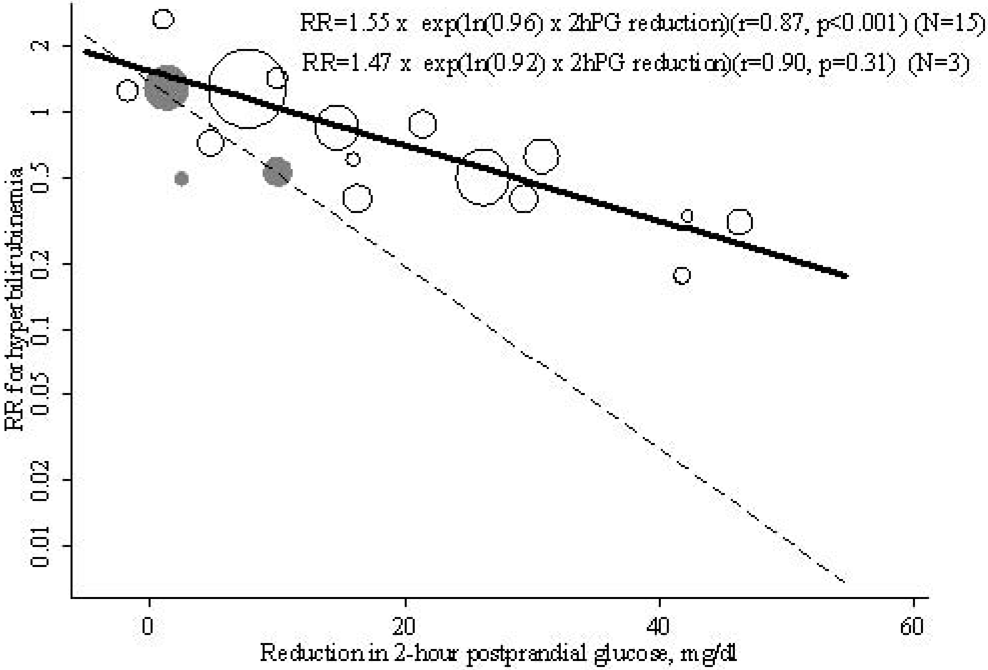

RR=1.55 x exp(ln(0.96) x 2hPG reduction)(r=0.87, p<0.001) (N=15)
RR=1.47 x exp(ln(0.92) x 2hPG reduction)(r=0.90, p=0.31) (N=3)
RR for hyperbilirubinemia
2
1
0.5
0.2
0.1
0.05
0.02
0.01
0
20
40
60
Reduction in 2-hour postprandial glucose, mg/dl
